# Vascular Endothelial Growth Factor A and Leptin Expression Associated with Ectopic Proliferation and Retinal Dysplasia in Zebrafish Optic Pathway Tumors

**DOI:** 10.1089/zeb.2016.1366

**Published:** 2017-08-01

**Authors:** Laura E. Schultz, Staci L. Solin, Wesley A. Wierson, Janna M. Lovan, Judith Syrkin-Nikolau, Deborah E. Lincow, Andrew J. Severin, Donald S. Sakaguchi, Maura McGrail

**Affiliations:** ^1^ Department of Genetics, Development and Cell Biology, Iowa State University, Ames, Iowa.; ^2^ Genome Informatics Facility, Office of Biotechnology, Iowa State University, Ames, Iowa.

**Keywords:** retina, dysplasia, progenitor, proliferation, VEGFA, leptin

## Abstract

In the central nervous system injury induces cellular reprogramming and progenitor proliferation, but the molecular mechanisms that limit regeneration and prevent tumorigenesis are not completely understood. We previously described a zebrafish optic pathway tumor model in which transgenic *Tg(flk1:RFP)is18/+* adults develop nonmalignant retinal tumors. Key pathways driving injury-induced glial reprogramming and regeneration contributed to tumor formation. In this study, we examine a time course of proliferation and present new analyses of the *Tg(flk1:RFP)is18/+* dysplastic retina and tumor transcriptomes. Retinal dysplasia was first detected in 3-month-old adults, but was not limited to a specific stem cell or progenitor niche. Pathway analyses suggested a decrease in cellular respiration and increased expression of components of Hif1-α, VEGF, mTOR, NFκβ, and multiple interleukin pathways are associated with early retinal dysplasia. Hif-α targets VEGFA (*vegfab*) and Leptin (*lepb*) were both highly upregulated in dysplastic retina; however, each showed distinct expression patterns in neurons and glia, respectively. Phospho-S6 immunolabeling indicated that mTOR signaling is activated in multiple cell populations in wild-type retina and in the dysplastic retina and advanced tumor. Our results suggest that multiple pathways may contribute to the continuous proliferation of retinal progenitors and tumor growth in this optic pathway tumor model. Further investigation of these signaling pathways may yield insight into potential mechanisms to control the proliferative response during regeneration in the nervous system.

## Background

Elucidating the molecular mechanisms controlling cellular reprogramming and regeneration is important for improved treatment of nervous system injury and disease. Proper regulation of regenerative programs is necessary to limit proliferation and prevent cellular transformation and neoplasia. In injured tissue dying cells and damaged vasculature lead to inflammation and a hypoxic environment.^1,2^ The pro-inflammatory cytokine and hypoxia inducible factor Hif-α pathways are critical for mediating the cellular response to cell death and hypoxia.^3,4^ How activation of hypoxia and inflammatory pathways contribute to proliferation and regeneration in the nervous system is still not completely understood.

Hypoxia has been shown to activate regeneration gene programs in injured peripheral sensory neurons by Hif1-α induced expression of its direct target, the vascular endothelial growth factor (VEGF).^5^ VEGF receptor tyrosine kinase signaling is essential to embryonic blood vessel development, adult angiogenesis, tumor vascularization, and disease-related vascular retinopathies.^6^ In addition, multiple nonvascular functions have been described for VEGF in the developing and adult central nervous system (CNS).^7,8^ VEGF is expressed by glial progenitors and astrocytes in response to hypoxia in the neonatal subventricular zone and stimulates proliferation of glial progenitors.^9^ In the subgranular zone/neurogenic niche of the adult mouse hippocampus, secretion of VEGFA and VEGFB by neural stem and progenitor cells is thought to influence the microenvironment and maintain the stem cell pool.^10^ Treatment of embryonic cortical neural precursors *in vitro* with VEGF induces expression of E2F family members and cell cycle regulators cyclin D1, cyclin E, and cdc25, supporting the above observations that VEGF may have a direct role in promoting progenitor proliferation *in vivo*.^11^ In the retina, VEGF has been shown to act directly on chick retinal progenitors to promote proliferation;^12^ however, whether VEGF mediates the proliferative response to hypoxia in the retina has not previously been shown.

A second transcriptional target of Hif-α, the cytokine Leptin, is expressed in response to hypoxia^13^ and has been shown to stimulate proliferation of neural progenitors^14^ and regulate angiogenesis^15–17^ and wound healing.^18^ The presence of Leptin in the eye vitreous is associated with inflammation and vision loss in patients with diabetic retinopathy.^19^ Experimentally, Leptin expression is increased as part of the inflammatory response in a guinea pig model of uveitis^20^ and induces accumulation of reactive oxygen species in human endothelial cells.^21^ This suggests that Leptin and VEGF together might integrate hypoxia and inflammatory signaling pathways, which are both implicated in solid tumor initiation and cancer progression.^22–24^ Most recently it has been shown that extended exposure of normal bronchial epithelial cells to hypoxia and pro-inflammatory cytokines TNF-α and IL1-β induces cancer-like phenotypic changes, providing a link among hypoxia, inflammation, and cellular transformation *in vitro*.^25^ How sustained signaling due to a combination of hypoxia and chronic inflammation contributes to transformation in solid tumor initiation *in vivo* remains an open question. Moreover, the connection between hypoxic induction of Hif-α targets, such as VEGF and Leptin, to neoplastic transformation and tumor induction is less clear than the well-documented role of these factors in vascularization supporting tumor growth.^26^

Zebrafish has been used extensively as a model system for studying activation of latent progenitor populations and the molecular pathways controlling injury induced regeneration in the vertebrate nervous system.^27,28^ Two populations of progenitor cells contribute to the growth of the zebrafish retina; latent progenitors derived from Müller glia in the inner nuclear layer and neural progenitors from neuroepithelial stem cells present in the ciliary marginal zone at the retina periphery.^28^ Chemical, mechanical, genetic, and light-induced injury of the zebrafish retina has revealed multiple growth factor, and cytokine signaling pathways stimulate latent progenitor proliferation and Müller glia dedifferentiation and reprogramming, both of which contribute to regeneration.^29–31^ Together these studies reveal the importance of coordinated activation of multiple pathways, including heparin-binding EGF-like growth factor, Wnt, Leptin, Interleukin-6, and Jak/Stat signaling, in progenitor proliferation and subsequent fate specification and differentiation.^31,32^

The initial response to injury in the zebrafish retina involves transient expression of the pro-inflammatory cytokine TNF-α, which prevents gliosis and stimulates induction of inner nuclear layer progenitor proliferation and regeneration.^33^ The limited regenerative response indicates that control mechanisms are enacted to prevent unregulated growth and dysplasia. Hif-α and mTOR signaling have been reported to work together in regulating ciliary marginal zone progenitor proliferation in Xenopus retina after nutrient starvation,^34,35^ and mTOR has been shown to be required for Müller glia-derived progenitor proliferation in injured chick retina.^36^ A role for Hif-α or mTOR signaling in zebrafish retinal development and regeneration has not previously been reported.

We previously reported the characterization of a zebrafish optic pathway tumor model in which transgenic *Tg(flk1:RFP)is18/+* adults develop nonmalignant retinal tumors at ∼80% penetrance.^37^ The molecular basis for tumor induction is not known; however, our analyses showed that the retinal tumors may originate, in part, from Müller glia-derived progenitors, and activated signaling pathways in the tumor transcriptome are similar to injury induced regeneration pathways driving Müller glia reprogramming and progenitor proliferation. In contrast to injury induced regeneration models, our retinal tumor model is distinct in that TNF-α expression is not upregulated and proliferation is not transient. Once initiated, proliferation is sustained and contributes continuously to the growth of dysplastic tissue and the development of nonmalignant glial-like tumors. These observations indicate that other molecular pathways can stimulate retinal progenitor proliferation and override the normal controls that limit regeneration.

To identify pathways that might be associated with proliferation and the responsive retinal cell populations, we performed additional immunocytochemical and differential gene expression analyses on adult *Tg(flk1:RFP)is18/+* retina. Early proliferation and dysplasia were not restricted to the ciliary marginal zone or to Müller glia-derived progenitors in the inner nuclear layer, the normal stem cell/progenitor niches of the teleost retina.^28,38^ Ingenuity pathway analysis (IPA) of the early dysplastic retina identified Hif-α signaling targets VEGF and Leptin, components of inflammation pathways NFκβ, IL-1, IL-6, and IL-8, and pathways required for Müller glia-derived progenitor proliferation in injured retina, including HBEGF^32,39^ and mTOR.^36^ Novel pathways that act in tissue repair and regeneration,^40^ but not previously implicated in retinal regeneration, were represented, such as GADD45 growth arrest and DNA damage response, endothelin-1 signaling, and caveolin-1-mediated endocytosis. Our results suggest that multiple signaling pathways might trigger progenitor proliferation and contribute to tumor growth. The *Tg(flk1:RFP)is18* tumor model may provide insight into the molecular mechanisms that prevent neoplastic transformation during regeneration in the nervous system.

## Materials and Methods

### Zebrafish

The *Tg(flk1:RFP)is18* transgenic zebrafish line predisposed to optic pathway tumors was previously described.^37^ Zebrafish were housed in an Aquatic Habitat system (Aquatic Ecosystems, Inc.) and maintained on a 14-h light/14-h dark cycle at 27°C. Transgenic fish predisposed to tumor formation were raised side by side with nontransgenic siblings. Heterozygous and homozygous transgenic fish and sibling fish were monitored daily during routine feeding for viability and morbidity, and monitored bi-weekly for gross presentation of ocular tumors. Juvenile and adult fish were anesthetized and euthanized in MS-222 Tricaine Methanesulfonate according to experimental protocols approved by the Iowa State University Institutional Animal Care and Use Committee (Log # 11-06-6252-I) in compliance with the American Veterinary Medical Association and the National Institutes of Health guidelines for the humane use of laboratory animals in research. Adult fish were anesthetized and euthanized in MS-222 Tricaine Methanesulfonate before sacrifice and tissue dissection for RNA isolation, histopathology, and immunolabeling.

### Histopathology and immunocytochemistry

One month old intact juvenile fish or whole heads dissected from adult zebrafish were fixed in Davidson's fixative (2:3:1:3 Formalin:Ethanol:Glacial Acetic Acid:Water) for 16 h at 4°C, decalcified in Cal-Ex (Fisher) for 2 days at 4°C, and processed and embedded in paraffin blocks at the Clinical Histopathology Laboratory at Iowa State University. Paraffin blocks were serial sectioned at 6 μm on a Shandon Finesse 325 microtome. Slides were stained with Hematoxylin 7211 Richard-Allan Scientific (Fisher) and 3% Eosin Y (Argos Organics) and mounted in Permount (Fisher). To aid antigen retrieval for PCNA labeling, slides with sectioned tissue were pretreated with 10 mM Sodium Citrate. Mouse monoclonal anti-PCNA (Sigma P8825) was used at 1:1000; rabbit anti-Sox2 polyclonal (Sigma-Aldrich/Millipore AB5603) was used at 1:200; rabbit anti-phospho-S6 ribosomal protein (Ser235/236; Cell Signaling 2211) was used at 1:300; and Alexa-488 and Alexa-594 conjugated secondary antibodies (Invitrogen) were used at a dilution of 1:500. Antibody-labeled slides were stained with 4′,6-diamidino-2-phenylindole (DAPI) and mounted in Fluorogel (EMS). Hematoxylin and Eosin (H&E) stained slides were imaged on a Zeiss Axioskop II using a Nikon Rebel camera. Immunofluorescence was imaged on a Zeiss LSM700 laser scanning confocal microscope. Images of histological staining and antibody labeling from the same fish were captured from tissue sections separated by ∼100 μm.

### Quantification and statistics

For analysis of proliferation in early dysplastic regions of the retina, sections of retinal tissue adjacent to a section stained with H&E (Fig. 2) were labeled with PCNA and Sox2 antibodies. The total number of PCNA and Sox2 labeled cells was counted in three retinal sections each from three wild-type and three *Tg(flk1:RFP)is18/+* age-matched siblings (18 sections total). The total number of positively labeled cells was counted separately in two regions of the retina: at the ciliary marginal zone and in a 300 μm length of the inner retina. To quantify phospho-S6 labeling, three individual wild-type and three individual *Tg(flk1:RFP)is18/+* retinal tumors were examined. From each individual, three serial sections were imaged at 63 × for a total of 18 images. The total number of DAPI positive cells and phospho-S6 positive cells were counted in a 100 μm × 100 μm region of wild-type retina and *Tg(flk1:RFP)is18/+* tumor tissue in each section and used to calculate the percentage of phospho-S6 positive cells. Statistical comparison of quantification in wild type and *Tg(flk1:RFP)is18/+* was performed using student's *t*-test in GraphPad Prism software.

### *In situ* hybridization

cDNA was amplified by reverse transcription-polymerase chain reaction out of total RNA isolated from wild-type 5 days post-fertilization embryos and was cloned into the pCR4-TOPO vector (Invitrogen). Primers for amplification were as follows: *apoe* forward 5′ TAGCGGCCGCGAATTCGCCC 3′, *apoe* reverse 5′ TAGTCCTGCAGGTTTAAACGA 3′; *lepb* forward 5′ TGCTTGTTAATATCATCCCTGGT 3′, *lepb* reverse 5′ GAGAATGAATGTCTCAGCCACA 3′; *lepr* forward 5′ CGCTGTAAAGACGTGAACGA 3′, *lepr* reverse 5′ TCTGCCTGAAGTCCATTCCT 3′; *flk1* forward 5′ AAGTGGCTAAAGGCATGGAGTTC 3′, *flk1* reverse 5′ GACACTCCATCTCCGAGTCAAGG 3′; *vegfab* forward 5′ CGCGTGCTCCAGTTATTTATTGTG 3′, *vegfab* reverse 5′ CACCTCCTTGGTTTGTCACATCTG 3′; *vegfaa* forward 5′ TGATACAGTTATTTCTCGCGGCTC 3′, *vegfaa* reverse 5′ TTTGCAGGAGCATTTACAGGTGAG 3′. Digoxigenin-labeled probes for *in situ* hybridization were synthesized using a DIG RNA labeling mix (Roche #11277073910) and T3 RNA polymerase (Roche #11031163001) and stored in 50% formamide at −20°C. Adult zebrafish tissues were dissected, fixed in 4% paraformaldehyde, and embedded in optimal cutting temperature medium. *in situ* hybridization on 12–16 μm cryosections of head and eye tissue was performed as described.^41^ Tissues were photographed on a Zeiss Axioskop II microscope using a Nikon Rebel camera.

### RNA-Seq and transcriptomic analyses

The transcriptome of retinal and tumor tissue from age-matched wild-type sibling and *Tg(flk1:RFP)is18*/+ 6-month-old adults was reported previously^37^ and the data deposited at the ArrayExpress database (www.ebi.ac.uk/arrayexpress) under accession number E-MTAB-2886. Briefly, total RNA from dissected retinas or tumor tissue from age-matched 6-month-old sibling fish was isolated using an RNeasy RNA Isolation Kit (Qiagen). Three dissected wild-type sibling retina, heterozygous *Tg(flk1:RFP)is18*/+ dysplastic retina, and heterozygous *Tg(flk1:RFP)is18*/+ retinal tumor tissue were pooled and used for total RNA isolation. *Tg(flk1:RFP)is18*/+ retinas were classified as dysplastic or tumor based on the gross appearance of the tissue. Dissected *Tg(flk1:RFP)is18/+* retinas that were thicker than wild type but did not have obvious lesions were used for the dysplastic retina sample. Dissected *Tg(flk1:RFP)is18/+* eyes that contained large masses filling the vitreous were used for the retinal tumor tissue sample. A single RNA-Seq library for 100 bp paired end sequencing was prepared for each sample and the three libraries sequenced at the Genome Sequencing and Analysis Core Resource, Duke Institute for Genome Sciences and Policy, Duke University. Wild-type, dysplastic retina, and retinal tumor RNA-Seq libraries contained 436,511,378, 444,838,528, and 422,324,454 reads, respectively.

As previously described,^37^ sequences were mapped to the zebrafish reference genome v9 using GSNAP, counted with HTSeq-count, upper quartile normalization applied, and the Fisher's exact test used to determine differential gene expression. q-value estimation of false discovery rate was performed in R using the open source software qvalue (http://bioconductor.org/biocLite.R). In the present study genes with at least 10 read counts were used for downstream analyses. Gene ontology GO Term^42^ analyses were done using the Princeton GO Term Finder website (http://go.princeton.edu/cgi-bin/GOTermFinder). Cluster frequency was calculated as the percentage of genes from the dataset significantly associated with a particular term, while genome frequency (or background frequency) was calculated as the percentage of all genes in the *Danio rerio* genome associated with a particular term. *p*-value cutoffs were set at 0.01. *p*-values were Bonferroni corrected with an estimated false discovery rate of <0.01.

Transcriptome data were analyzed with QIAGEN's IPA (QIAGEN Redwood City; www.qiagen.com/ingenuity) using genes having a read count of at least 10 in all samples and a three-fold change in expression between wild-type retina and *Tg(flk1:RFP)is18/+* dysplastic retina or retinal tumor samples. Ensembl gene IDs for human homologs were extracted using Biomart^43^ at (http://useast.ensembl.org/biomart/martview/da30878652120cac4e352ddac342a16e).

### Availability of supporting data

Raw transcriptome dataset supporting the conclusions of this article is available in the ArrayExpress repository (E-MTAB-2886 https://ebi.ac.uk/arrayexpress/experiments/E-MTAB-2886/). An excel file containing normalized read counts for all genes was previously published in Solin *et al.*^37^

## Results

### Analysis of cellular proliferation and dysplasia in *Tg(flk1:RFP)is18/+* adult retina

To examine morphological changes and dysplasia in the *Tg(flk1:RFP)is18* retina, we performed histopathology on serial sectioned head tissue from cohorts of age-matched wild-type sibling and *Tg(flk1:RFP)is18/+* zebrafish. Wild-type and transgenic siblings were sacrificed at 4 weeks [*n* = 10 wild type, *n* = 10 *Tg(flk1:RFP)is18/+*], 3 months/11 weeks [*n* = 8 wild type, *n* = 9 *Tg(flk1:RFP)is18/+*], and 4 months [*n* = 5 wild type, *n* = 15 *Tg(flk1:RFP)is18/+*] of age. In all individuals 6 μm sections extending from the anterior to posterior limits of the eyes were examined for the frequency and location of ectopic cell proliferation and abnormal retinal architecture.

**FIG. 1.**
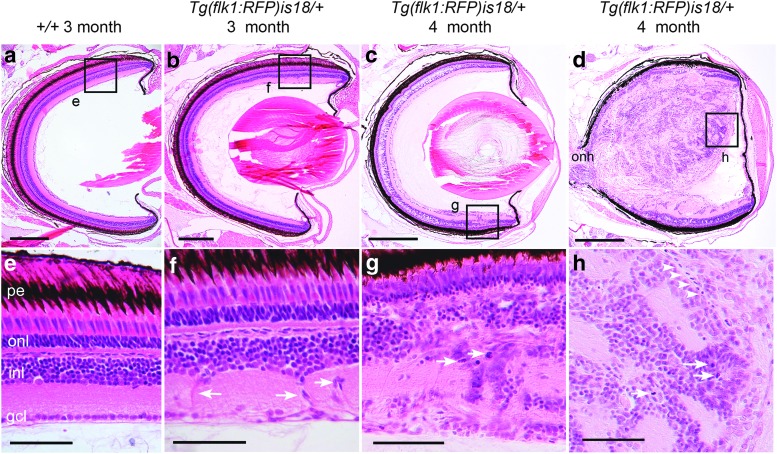
Detection of aberrant cell migration and ectopic proliferation in young adult *Tg(flk1:RFP)is18* dysplastic retina. **(a, e)** Histological staining of retina section from 3-month-old adult wild-type zebrafish showing nuclear layers and organized laminar structure. **(b, f)** Representative image of section through retina of 3-month-old *Tg(flk1:RFP)is18* adult reveals aberrant migration of cells across the inner nuclear layer **(***arrows*, **f)** (*n* = 9). **(c–h)** Representative images of retinal sections from 4-month-old *Tg(flk1:RFP)is18* adults (*n* = 15). **(c, g)** Retina from 4-month-old *Tg(flk1:RFP)is18* adult with dysplasia reveals disruption of inner, outer, and ganglion cell layers with numerous mitotic figures **(***arrows*, **g)**. **(d, h)** Four-month-old *Tg(flk1:RFP)is18* adult with advanced retinal tumor filling the vitreous space. Tumor tissue is composed of fibrous material interspersed with numerous mitotic figures, cells showing heterogeneous nuclear morphology and forming occasional rosettes **(***arrows*, **h)**, and blood vessels **(***arrowheads*, **h)**. pe, pigmented epithelium; onl, outer nuclear layer; inl, inner nuclear layer; gcl, ganglion cell layer. Scale bars **(a, b)** 200 μm; **(c, d)** 500 μm; **(e–h)** 50 μm.

Comparison of 10 four-week-old *Tg(flk1:RFP)is18/+* with 10 age-matched wild-type siblings did not reveal any obvious morphological abnormalities or ectopic proliferation in the retinas, indicating that during juvenile stages in *Tg(flk1:RFP)is18/+* heterozygotes, the growth of the retina and the rate of progenitor proliferation and production of neurons and Müller glia proceed normally. At 3 months of age, in comparison to wild-type retina (Fig. 1a, e), 6/9 *Tg(flk1:RFP)is18/+* fish had regions of ectopic proliferation in the retina in at least one eye that contained clusters of proliferating cells in the inner nuclear layer, inner plexiform layer, or retinal ganglion cell layer (Fig. 1b, f). Cells with processes stretched across the inner plexiform layer were frequent (Fig. 1f, arrows). In contrast, the ciliary marginal zone appeared normal compared to wild type.

A similar frequency of retinal abnormalities was observed in 4-month-old *Tg(flk1:RFP)is18/+* fish, with 10 out of 15 individuals presenting with ectopic proliferation in one or both retina. However, phenotypes ranged from retina with a normal appearance to retina containing multiple regions of proliferation and disorganization with gaps in the inner nuclear layer or large lesions distending the nuclear layers (Fig. 1c, g) and retina completely replaced by neoplastic tumors that filled the vitreal cavity (Fig. 1d, h), which was not observed in the 3-month-old retinas. Regions of proliferation and disorganization were expanded across the retina layers and included the outer nuclear layer (Fig. 1c, g). Mitotic figures, which are rarely detected in mature regions of wild-type adult retina, were numerous in these regions (Fig. 1g, arrows). Larger tumors contained rosettes with mitotic figures (Fig. 1h, arrows) and blood vessels (Fig. 1h, arrowheads) embedded in a fibrous matrix, similar to the advanced retinal tumors previously reported in older adult *Tg(flk1:RFP)is18/+* fish.^37^

**FIG. 2.**
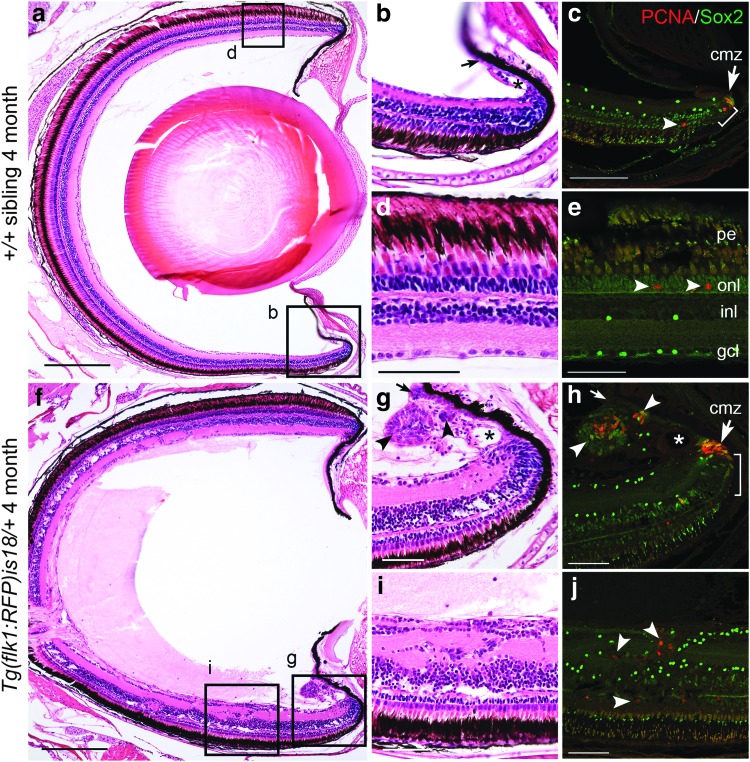
Ectopic proliferation and Sox2 expressing cells in young adult *Tg(flk1:RFP)is18* dysplastic retina. **(a–e)** Wild-type 4-month-old adult (*n* = 3) shows that PCNA positive cells are restricted to stem/progenitor cells at the ciliary marginal zone **(**cmz, *arrow*, **c)** and rod precursor cells in the photoreceptor outer nuclear layer **(***arrowheads*, **e)**. Sox2-expressing cells overlap with PCNA positive stem cells at the ciliary marginal zone **(***bracket*, **c)** and are present in amacrine/displaced amacrine cells in the inner nuclear and ganglion cell layers **(c, e)**. **(f–j)** Retina from 4-month-old *Tg(flk1:RFP)is18* adult (*n* = 3) shows numerous regions of proliferation distributed throughout the neural retina **(f)**. The ciliary marginal zone **(g, h)** and ventral ciliary circumferential artery **(***asterisk*, **g)** are expanded. Small masses of PCNA and Sox2 positive cells **(***arrowheads*, **g, h)** are present in the normally single-cell layered nonpigmented epithelium extending from the ciliary marginal zone **(**small *arrow*, **g, h**–compare to small *arrow* in wild type **b)**. Ectopic regions of proliferation in the neural retina contain disorganized cells expressing PCNA and Sox2 **(***arrowheads*, **j)**. cmz, ciliary marginal zone; gcl, ganglion cell layer; inl, inner nuclear layer; onl, outer nuclear layer; pe, pigmented epithelium. Scale bars **(a, f)** 200 μm; all other panels 50 μm.

To examine proliferation and progenitor cells in early dysplastic retina, serial sections from three wild-type and three *Tg(flk1:RFP)is18/+* 4-month-old adults were histologically stained with H&E and adjacent sections labeled with PCNA and Sox2 antibodies (Fig. 2). In wild type (Fig. 2a–e) a small number of PCNA and Sox2-labeled cells were present at the ciliary marginal zone (Fig. 2c, arrow) and in the region just adjacent to the ciliary marginal zone (Fig. 2c, bracket). Few PCNA positive cells were detected in the outer nuclear layer at the periphery (Fig. 2c, arrowhead) or central regions of the retina (Fig. 2e, arrowheads). The Sox2 expressing cells detected in the inner nuclear layer and ganglion cell layer (Fig. 2c, e) have previously been described as Müller glia, amacrine, and displaced amacrine cells.^44^

**FIG. 3.**
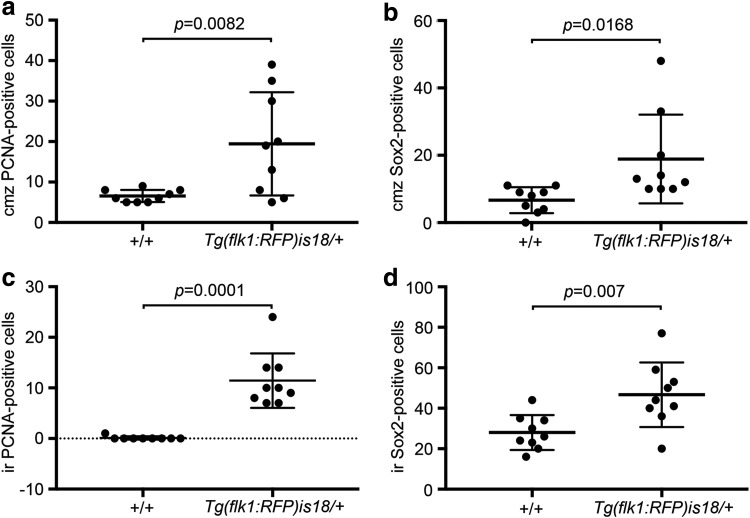
The ciliary marginal zone and dysplastic regions of *Tg(flk1:RFP)is18/+* retina in 4-month-old adults contain increased numbers of PCNA and Sox2 positive cells. A total number of PCNA and Sox2 labeled cells were counted at the ciliary marginal zone and in a 300 μm long section of the internal retina in three wild-type and three age-matched 4-month-old *Tg(flk1:RFP)is18/+* siblings containing a region of dysplasia identified by Hematoxylin and Eosin staining. **(a)** Ciliary marginal zone PCNA positive cells 19 ± 4 versus 7 ± 1, *p* = 0.0082. **(b)** Ciliary marginal zone Sox2 positive cells 19 ± 4 versus 7 ± 1, *p* = 0.0168. **(c)** Internal retina PCNA positive cells: 11 ± 2 versus <1, *p* = 0.0001. **(d)** Internal retina Sox2 positive cells: 47 ± 5 versus 28 ± 3, *p* = 0.007. *n* = 9 retinal sections, three each from three individuals of each genotype. Error bars represent standard error of the mean. cmz, ciliary marginal zone; ir, internal retina.

In contrast to wild type, 3/3 four-month-old *Tg(flk1:RFP)is18/+* adults had regions of ectopic proliferation in multiple serial sections (Figs. 2 and 3). Groups of cells in the inner plexiform layer and gaps in the inner nuclear layer could be detected at multiple locations in a single retina (Fig. 2f). The ciliary marginal zone (Fig. 2g, h, arrow), and adjacent progenitor region (Fig. 2g, h, bracket), appeared expanded in the *Tg(flk1:RFP)is18/+* retina compared to wild type. The diameter of the ventral ciliary circumferential arteries was increased (Fig. 2g, h, asterisk), and the nonpigmented epithelium that lines the vitreal face of the iris was expanded (Fig. 2g, h, small arrow) and contained small masses with PCNA and Sox2 expressing cells (Fig. 2g, h, arrowheads). In the internal retina, PCNA was detected in cells within each nuclear layer and in disorganized masses in the inner plexiform layer (Fig. 2i, j, arrowheads). Sox2 expressing cells were present scattered throughout the masses and inner nuclear layer and ganglion cell layer. Quantification of Sox2 and PCNA positive cells at the ciliary marginal zone and regions of dysplasia in the inner retina showed that the increase in *Tg(flk1:RFP)is18/+* compared to age-matched wild-type siblings was significant (Fig. 3).

In summary, the histological analysis of tumor onset indicates that in the *Tg(flk1:RFP)is18/+* retina, neurogenesis proceeds normally through juvenile stages of development. Abnormal proliferation begins in early adulthood and occurs in the inner nuclear layer and ganglion cell layers of the retina (Fig. 1). The nonpigmented epithelium that extends away from the ciliary marginal zone along the vitreal surface of the iris is also affected (Fig. 2g). In contrast, the ciliary marginal zone appears expanded but relatively normal overall, even in fish with advanced tumors (Figs. 1d and 2g). The data suggest that multiple cell populations in the *Tg(flk1:RFP)is18/+* retina are stimulated to proliferate and may include cells in the pigmented epithelium or ganglion cell layer, in addition to the known progenitor populations in the inner nuclear layer and at the ciliary marginal zone.^38^

### Transcriptomics revealed elevated expression of VEGF, leptin, and mTOR signaling pathways correlates with *Tg(flk1:RFP)is18/+* retinal dysplasia

**FIG. 4.**
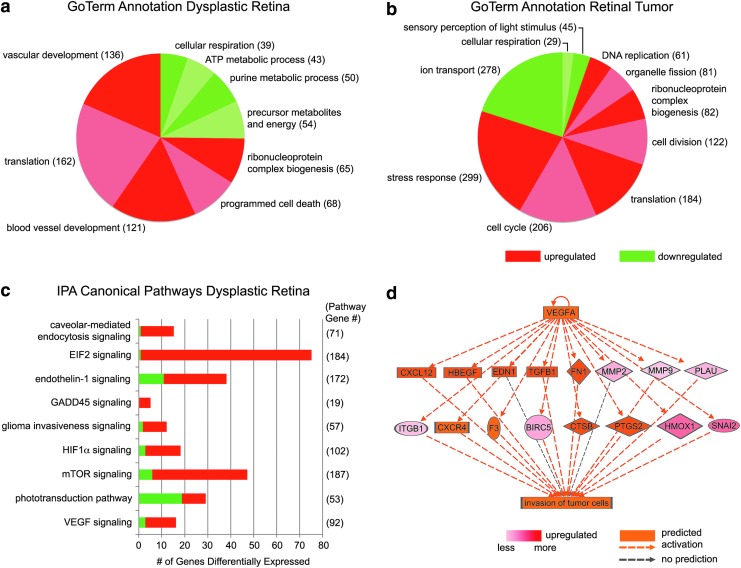
*Tg(flk1:RFP)is18/+* dysplastic retina associated GoTerm and IPA canonical pathways. **(a)** GoTerm analysis of DGE of dysplastic retinal tissue suggests decreased oxidative metabolism and increased vasculogenesis pathways. **(b)** GoTerm analysis of DGE of retinal tumor tissue is consistent with loss of phototransduction and increased proliferation. **(c)** IPA canonical pathways to which the *Tg(flk1:RFP)is18/+* dysplastic retina transcriptome is associated. **(d)** IPA regulator pathway analysis of VEGF targets involved in cytokine signaling, transcriptional response, and extracellular matrix remodeling underlying invasion of tumor cells. IPA, ingenuity pathway analysis; VEGF, vascular endothelial growth factor.

We previously reported transcriptome analysis of age-matched, adult wild-type retina; *Tg(flk1:RFP)is18/+* dysplastic retina (formerly designated Pretumor) and *Tg(flk1:RFP)is18/+* retinal tumor tissue were consistent with activation of signal transduction pathways known to be required for Müller glia reprogramming during injury induced regeneration.^37^ GO Term analysis of the RNASeq dataset using genes with an fragments per kilobase of transcript per million mapped reads (FPKM) of ≥1 and a significant change in expression level between samples (*p* ≤ 0.01) revealed translation; vascular development and blood vessel development processes were upregulated in *Tg(flk1:RFP)is18/+* dysplastic retina (Fig. 4a and Supplementary Tables S1–S4; Supplementary Data are available online at www.liebertpub.com/zeb). In retinal tumor the analysis revealed a significant decrease in phototransduction and ion transport and an increase in cellular processes necessary for proliferation, including DNA replication, cell division, and ribonucleoprotein complex biogenesis (Fig. 4b and Supplementary Tables S1–S4).

We used IPA analysis to further examine the altered molecular pathways that might initiate transformation and proliferation of progenitor cells leading to tumor formation. To increase the stringency of the analysis, the original RNA-Seq data were first filtered to remove genes with a read count of <10 in wild-type retina, dysplastic, or tumor tissues. Next, genes with less than three-fold change in expression level between wild-type retina and dysplastic retina, or between wild-type retina and tumor tissue, were removed, leaving a set of 2587 genes. Human homologs of the 2587 gene set were recovered at BioMart.^43^ Individual zebrafish genes of interest absent from the BioMart conversion (i.e., ENSDARG00000045548_*lepb*, ENSDARG00000076972_si:dkey-208k22.3_*plvap*) were identified using BLAST, leaving 2570 genes for IPA analysis (Supplementary Table S5).

Pathways with the most significant overlap in the dysplastic tissue were EIF2 Signaling (*p* = 4.65E-26), Phototransduction Pathway (*p* = 4.65E-15), mTOR signaling (*p* = 2.28E-08), and Cell Cycle Control of Chromosomal Replication (1.46E-07). General categories of disease, disorders, and molecular and cellular function were consistent with injury and tumorigenesis and included cancer, organismal injury and abnormalities, and neurological disease and predicted an increase in cell movement, growth, and proliferation and a decrease in cell death and apoptosis. Specific canonical signaling pathways represented in the dysplastic retina included caveolin-mediated endocytosis, EIF2 translation, Hif1-α, endothelin-1, VEGF, mTOR, and GADD45B (Fig. 4c and Supplementary Table S6). NFκβ and multiple interleukin pathways (IL1, IL6, and IL8) were present (Supplementary Table S6). A glioma invasiveness pathway was activated (Fig. 4c and Supplementary Table S6) as expected given the involvement of glial reprogramming pathways and the glioma-like cellular and molecular features of the tumors. Together the data suggest that activation of multiple signaling pathways is associated with dysplasia in the *Tg(flk1:RFP)is18/+* retina.

### VEGFA and leptin are induced in distinct cell populations in the early dysplastic *Tg(flk1:RFP)is18/+* retina

A number of genes with the greatest change in expression between wild-type and *Tg(flk1:RFP)is18/+* dysplastic retina are direct targets of hypoxia-inducible factor Hif1-α^45–48^ or components of the Hif1-α (*egln1a*, *egln3*, *lepb*, *vegfab*, *stc2a*), VEGF (*hmox1*, *ptgs2b*), or JAK/Stat signaling pathways (*socs3a*) (Supplementary Table S5). Other hypoxia-inducible targets whose expression was significantly increased included *cxcr4b*, *cxcl12*, *ddit4*, *and gadd45ba.* Hypoxia regulates the level of Hif1-α through protein stabilization (reviewed in Corcoran and O'Connor^2^). The level of *hif1aa* and *hi1ab* gene expression was not significantly changed between wild type, *Tg(flk1:RFP)is18/+* dysplastic retina, and tumor tissue. Together these observations suggest that a hypoxic environment is one possible trigger associated with ectopic proliferation in the *Tg(flk1:RFP)is18/+* retina. Because both VEGF and Leptin are direct targets of Hif1-α and have been shown to promote neural progenitor proliferation,^49,50^ we examined these two signaling molecules in more detail.

In dysplastic *Tg(flk1:RFP)is18/+* retina tissue, the level of *vegfaa* increased 2.9-fold and *vegfab* increased 11-fold. IPA analysis showed increased expression or activation of many components in the VEGF regulatory pathway that promotes tumor cell invasion (Fig. 4d and Supplementary Table S5), including multiple growth factors (*tgfβ*, *hbegf*), cytokines (*endothelin-1*, *cxcl12*), matrix metalloproteases, and peptidases (*mmp2*, *mmp9*, *plau*), and enzymes involved in prostaglandin synthesis (*cox2*, *ptgs2*). In the retina, VEGF promotes proliferation of retinal neural progenitors through activation of E2F using the MEK-ERK signal pathway.^12,51^ The E2F family of transcription factors has been shown to activate expression of gene networks regulating cell cycle progression, DNA damage and mitotic checkpoints, chromosome maintenance, and chromatin assembly.^52^ Many direct targets of E2F were significantly increased in the dysplastic retina transcriptome, including cyclin dependent kinase *cdk1* and *cdk2*, minichromosome maintenance deficit family members *mcm3*, *mcm5*, *mcm6*, and mitotic kinases aurora kinase *aurkb* and polo-like kinase *plk1* (Supplementary Table S5). Together, the data are consistent with VEGF's role as a mitogen that induces cell migration and stimulates cytokine signaling and angiogenesis.

A second target of Hif1-α signaling, the cytokine Leptin,^53^ was dramatically increased in the dysplastic retina transcriptome (∼500-fold) and remained elevated in tumor tissue (Supplementary Table S5). IPA analysis did not indicate a significant overlap of genes in the canonical pathways of which Leptin is a component, including Leptin signaling in obesity, insulin, or Jak/Stat. However, Leptin can mediate activation of the Jak/Stat pathway through induction of Socs3, one of the genes most highly altered in the dysplastic retina transcriptome (*socs3a*; Supplementary Table S5). Leptin has also been shown to synergize with interleukin IL-6 in Müller glia reprogramming in injury-induced regeneration.^32^ Together, this indicates that like VEGF, Leptin may play an important role in mediating the response to metabolic changes in the *Tg(flk1:RFP)is18/+* retina, either contributing to dysplasia and tumorigenesis or as a result of increased proliferation.

**FIG. 5.**
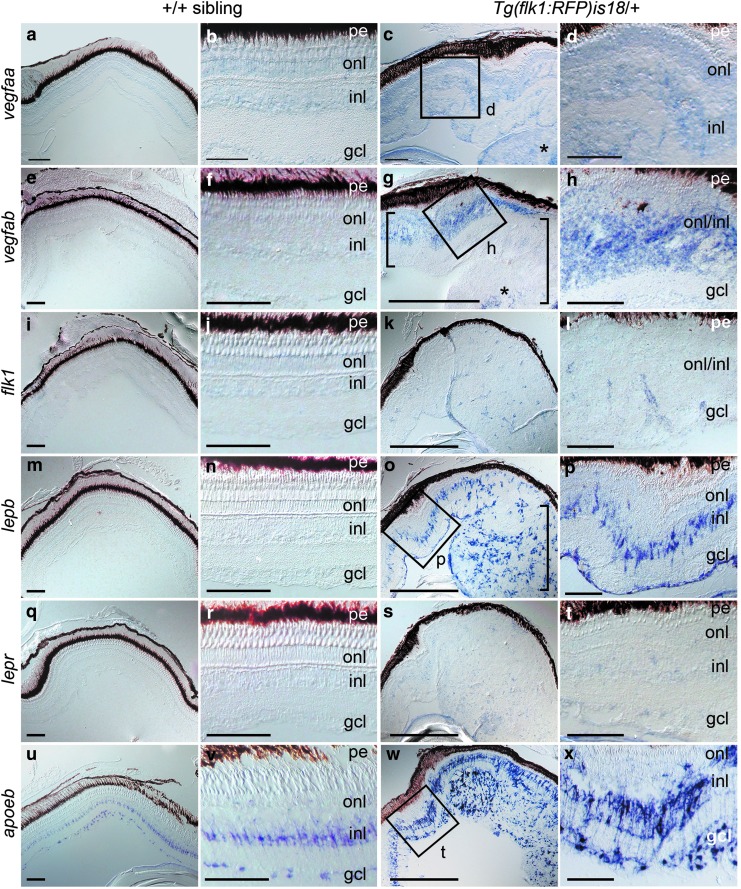
Induction of *vegfab* and *lepb* expression in distinct cell populations in *Tg(flk1:RFP)is18* dysplastic retina and retinal tumor. *In situ* hybridization of retina cryosections from +/+ **(a, b, e, f, i, j, m, n, q, r, u, v)** and *Tg(flk1:RFP)is18* **(c, d, g, h, k, l, o, p, s, t, w, x)** adult siblings. Weak expression of *vegfaa* is detected in the three nuclear layers of the retina **(a, b)** and in early dysplastic **(c** [*box*]**, d)** and retinal tumor tissue **(c,** *asterisk***)**. Expression of *vegfab* **(e, f)**, the VEGF receptor *flk1* **(i, j)**, *lepb* **(m, n)**, or the Leptin receptor *lepr* **(q, r)** is not detected in wild-type *+/+* retina. **(g, h)** In early dysplastic retina *vegfab* is detected throughout the inner and outer nuclear layers **(g** [*small bracket*]**, h).** In the tumor containing region (*large bracket*) *vegfab* is detected in a central region of the lesion (*asterisk*). **(k, l)** The VEGF receptor *flk1* was present in small groups of cells scattered throughout the tissue that may overlap with microvessels. **(o, p)** *lepb* is highly expressed in a subset of cells in the inner nuclear layer and cells in the ganglion cell layer in dysplastic retina and present in many cells evenly distributed in the early tumor (*bracket*). **(s, t)** The *lepr* receptor expression is faint and diffuse in dysplastic retina and tumor tissue. **(u, v)** In wild-type retina the glial marker *apoeb* is strongly expressed in inner nuclear layer Müller glia and astrocytes in the ganglion cell layer/nerve fiber layer. **(w, x)** *apoeb* expression is highly elevated in dysplastic retina and is detected in projections crossing the inner plexiform layer. Numerous cells throughout the tumor tissue labeled intensely with *apoeb*. gcl, ganglion cell layer; inl, inner nuclear layer; onl, outer nuclear layer; pe, pigmented epithelium. All scale bars, 100 μm, except panels **(g, k, o, s, t)** scale bars, 500 μm.

To examine which cell types in the retina express *vegfaa*, *vegfab*, and *lepb*, *in situ* hybridization was performed on retinal sections from wild type and *Tg(flk1:RFP)is18/+* adults (*n* = 3–5 biological replicates). The panel of *in situ* hybridization results shown in Figure 5 is composed of images from the same *Tg(flk1:RFP)is18/+* retina, to illustrate expression for multiple genes in a retina with dysplasia, as well as an advanced tumor. As shown in Figure 5, dysplastic retina was defined by a thickening and distortion of the neural retina layers compared to wild type, whereas tumor represents a mass that completely distorts the retinal layers and extends into the vitreous.

In wild-type retina the *vegfaa* signal was weak in all three nuclear layers (Fig. 5a, b) and was only slightly elevated in dysplastic retina and tumor (Fig. 5c [box]; d [asterisk]). *vegfab*, the VEGF receptor *flk1*, *lepb*, and the Leptin receptor *lepr* were not detected in any layer of the wild-type neural retina (Fig. 5e, f, i, j, m, n, q, r), as expected given the low number of reads in the wild-type retina transcriptome. The glial marker *apoeb* was readily detected in the inner nuclear layer and ganglion cell layer (Fig. 5u, v), presumably expressed in Müller glia and nerve fiber layer astrocytes, respectively. In regions of the *Tg(flk1:RFP)is18/+* retina with dysplasia, a high level of *vegfab* labeling was present and appeared to be expressed in the inner and outer nuclear layers, but was absent from the ganglion cell layer (Fig. 5g [small bracket], h). The retina also contained a region with a tumorous lesion that distended the nuclear layers (Fig. 5g, large bracket). *vegfab* was detected in a central region in the lesion (Fig. 5g, asterisk). The VEGF receptor *flk1* showed a low level of expression in cells scattered throughout the dysplastic region and larger lesion in the retina (Fig. 5k, l). In contrast to *vegfab*, in dysplastic tissue *lepb* expression appeared to localize specifically to Müller glia in the inner nuclear layer and in cells in the nerve fiber layer, presumably astrocytes (Fig. 5o, p). In addition, high levels of *lepb* were detected in clusters throughout the tumorous lesion (Fig. 5o, bracket), whereas little *vegfab* was present in a similar section (Fig. 5g, large bracket). The *lepr* receptor showed diffuse expression throughout both regions in the dysplastic retina (Fig. 5s, t).

Apolipoprotein ApoE is expressed by activated Müller glia and astrocytes in the injured retina and promotes neurite outgrowth of various neuronal cell types.^54^ *lepb* expression was similar to *apoeb*, which showed a substantial increase throughout the inner nuclear layer and ganglion cell layer and widespread expression in the retinal lesion, but appeared absent from neuronal cells (Fig. 5w, x), further supporting the restricted expression of *lepb* in glia within the *Tg(flk1:RFP)is18* dysplastic retina. Together these results indicate that in the *Tg(flk1:RFP)is18* retina, neurons and glia respond by expression of distinct growth factors and cytokines. *vegfab* expression is highly induced in neurons, and possibly Müller glia, in the inner nuclear layer. In contrast, *lebp* expression overlapped with *apoeb* and appeared restricted to the Müller glia and astrocytes in the nerve fiber layer.

**FIG. 6.**
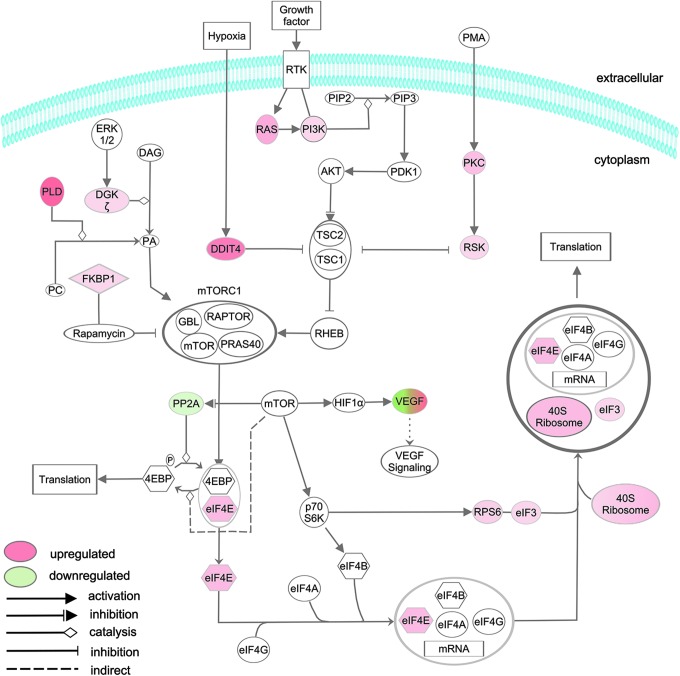
*Tg(flk1:RFP)is18/+* dysplastic retina genes associated with IPA canonical pathway mTOR signaling. Upstream components PLD, DDIT, RAS, and PI3K predicted to activate mTOR signaling are upregulated. Downstream effectors of mTOR signaling, such as translation initiation factors eIF4E and eIF3 and ribosomal proteins, including RPS6, are also upregulated. Mixed *red* and *green* color in VEGF symbol reflects ∼11-fold *vegfab* upregulation and *vegfb* three-fold downregulation. PLD, phospholipase D; DDIT, DNA damage induced transcript; PI3K, phosphoinositol 3-kinase.

A third canonical pathway identified by IPA analysis of the *Tg(flk1:RFP)is18/+* transcriptome was mTOR signaling (Fig. 4c), which regulates cell growth and metabolism.^55^ Activation of mTOR in response to hypoxia or growth factor signaling stimulates translation through phosphorylation of eukaryotic initiation factor 4E-binding protein and ribosomal protein S6-kinase, which stimulates phosphorylation of ribosomal protein S6 (Fig. 6). mTOR can also act upstream of HIF1-α to activate VEGF signaling (Fig. 6) indicating positive feedback between the hypoxia and mTOR pathways. Components of mTOR signaling located upstream and downstream of the mTORC1 complex that were upregulated in the dysplastic retina transcriptome included phospholipase D, DNA damage inducible transcript (DDIT4), RAS, phosphoinositol 3-kinase, eukaryotic translation initiation factors eIF4E and eIF3, ribosomal protein S6, and many ribosomal proteins (Fig. 6 and Supplementary Table S5).

**FIG. 7.**
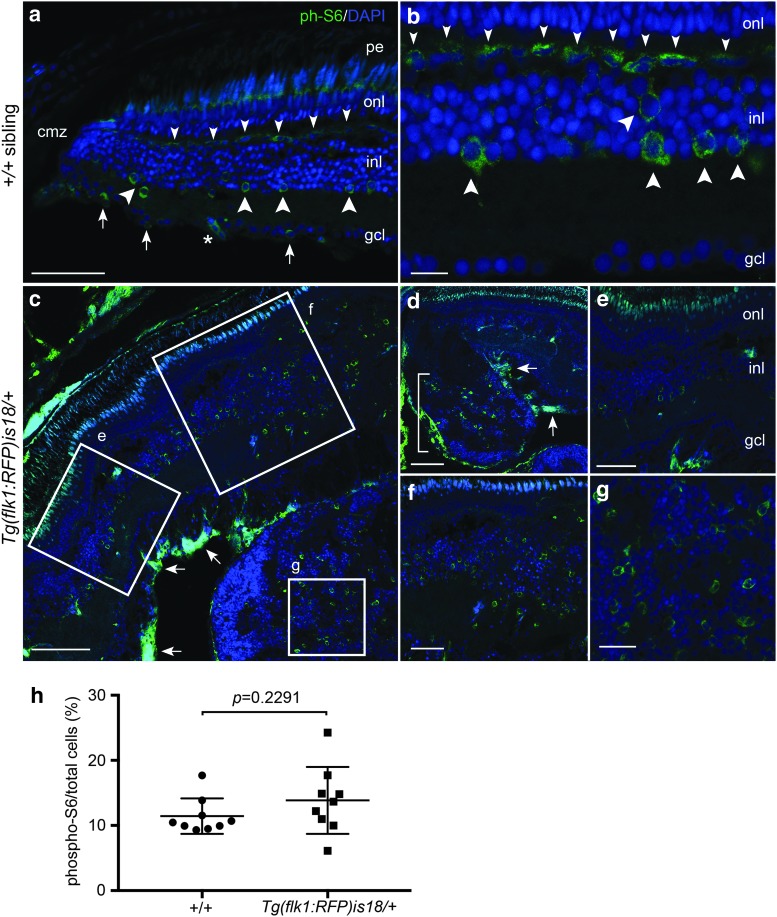
Activated mTOR signaling marker phosphorylated-S6 in adult zebrafish wild-type retina, *Tg(flk1:RFP)is18/+* dysplastic retina, and retinal tumor. **(a, b)** phospho-S6 (*green*) is detected in putative horizontal cells (*small arrowheads*), amacrine and/or Müller glia (*large arrowheads*), and retinal ganglion cells and/or displaced amacrine cells (*small arrows*) in the mature region of the retina. Nuclei are labeled with 4′,6-diamidino-2-phenylindole. *Asterisk* marks a blood vessel. **(c–g)** In *Tg(flk1:RFP)is18/+* dysplastic retina phospho-S6 labeling is present in a subset of cells throughout regions of the retina with increased cell number and disorganized retinal layers **(c–f)**. Intense labeling of phospho-S6 was present in the ganglion cell and nerve fiber layers **(c, d,** *arrows***)**. Large lesions contained phospho-S6 positive cells distributed throughout the tumor mass **(d** [*bracket*], **g)**. **(h)** Percentage of phospho-S6 cells/total cells in wild-type retina and *Tg(flk1:RFP)is18/+* tumor. +/+ 11.4% ± 0.9 vs. *Tg(flk1:RFP)is18/+* 13.9% ± 1.7, *p* = 0.2291. *n* = 9 sections, three sections each from three individuals of each genotype. gcl, ganglion cell layer; inl, inner nuclear layer; onl, outer nuclear layer; pe, pigmented epithelium. Scale bars, **(a, g, f)** 50 μm; **(b, c)** 20 μm; **(d, e)** 100 μm; **(h)** 25 μm.

To examine mTOR signaling in the *Tg(flk1:RFP)is18/+* retina tissues were labeled with an antibody specific for phosphorylated ribosomal protein S6 (phospho-S6) [*n* = 7 wild type and *n* = 8 *Tg(flk1:RFP)is18/+* biological replicates]. In wild-type adult zebrafish retina phospo-S6 labeling was detected in numerous cell types. In the inner nuclear layer of the mature retina phospho-S6 labeled putative horizontal cells (Fig. 7a, b, small arrowheads) and amacrine cells and/or Müller glia (Fig. 7a, b, large arrowheads). Phospho-S6 labeling was also detected in the retinal ganglion cell layer, present in retinal ganglion cells and/or displaced amacrine cells (Fig. 7a, arrows).^56^ Regions of the *Tg(flk1:RFP)is18/+* retina with dysplasia (Fig. 7c, d [bracket], e, f) or advanced tumor (Fig. 7g) showed phospho-S6 labeled cells distributed throughout the lesions. However, the percentage of phospho-S6 positive cells in *Tg(flk1:RFP)is18/+* tumor tissue was not significantly different than in wild-type retina (Fig. 7h, 11.4% ± 0.9 vs. 13.9% ± 1.7, *p* = 0.2291, *n* = 9 sections, three sections each from three individuals of each genotype). In the *Tg(flk1:RFP)is18/+* retina phospho-S6 was also detected in the nerve fiber layer on the vitreal side of the retina (Fig. 7d, e, arrows). This labeling may represent active mTOR signaling in retinal ganglion cells, displaced amacrine cells, astrocytes in the nerve fiber layer, or blood vessels of the capillary plexus that extend across the vitreal surface of the retina. Together the data indicate that mTOR signaling may contribute to the sustained growth of tumors in *Tg(flk1:RFP)is18/+* retina.

## Discussion

In this study we examined the onset of ectopic proliferation in the retina of adult *Tg(flk1:RFP)is18/+* zebrafish predisposed to nonmalignant retinal tumors and analyzed activation of signaling pathways that correlate with retinal dysplasia and tumor growth. A major finding from our time course analysis of proliferation is that multiple cell populations proliferate in the early dysplastic *Tg(flk1:RFP)is18/+* retina, including cells in the pigmented epithelium, ganglion cell layer, and inner nuclear layer, as well as at the ciliary marginal zone. We also observed aberrant cell migration across the inner nuclear layer in the very early dysplastic retina, which could contribute to dysplasia and tumor growth. These cells may originate from angiogenic sprouting of blood vessels in the nerve fiber layer in response to release of VEGF by inner nuclear layer neurons. Together, our data indicate that cells outside of the zebrafish retina ciliary marginal zone, in addition to Müller glia-derived progenitors, have the potential to proliferate.

A second major finding of our study is that expression of VEGFA and Leptin is significantly increased in early dysplastic retina, before formation of large lesions or tumors. Within the dysplastic retina neural and glial cell populations appear to respond differently, with *vegfab* expression upregulated in neurons and *lepb* expression overlapping with the glial marker *apoeb*. How this contributes to dysplasia in the retina, and separating and evaluating the distinct roles VEGF plays in neurogenesis directly versus indirectly through enhanced angiogenesis, will require additional investigation. Leptin has previously been reported to function in Müller glia reprogramming,^32^ and our results showing increased expression of *lepb* in *Tg(flk1:RFP)is18/+* retinal glia support a role for leptin signaling in glial-derived progenitor proliferation. Recent studies have identified a tissue regeneration enhancer element in the zebrafish *lepb* promoter that mediates high levels of *leptin* expression during fin and heart regeneration,^57^ suggesting that the elevated expression of *lepb* in *Tg(flk1:RFP)is18/+* retina may simply be due to enhancer activation. It is well documented that Hif1-α activates the VEGF and Leptin promoters,^5,53^ consistent with elevation of the Hif1-α pathway in the dysplastic retina transcriptome. However, it is equally likely that additional upstream triggers may activate VEGF and Leptin expression in the *Tg(flk1:RFP)is18/+* retina, leading to proliferation and dysplasia.

Our study also provides evidence that in wild-type adult retina mTOR signaling is active in numerous cells in the inner nuclear layer and retinal ganglion cell layer, which has not previously been reported in zebrafish. mTOR activation was detected in putative horizontal cells, amacrine cells, and retinal ganglion cells, but was absent from the ciliary marginal zone. In *Tg(flk1:RFP)is18/+* retina phospho-S6 positive cells were present in dysplastic regions with high cellularity and large tumor lesions. Compared to VEGF and Leptin signaling, mTOR activation may not be a factor in initiating inappropriate proliferation in the early dysplastic retina, but could contribute to the sustained growth of retinal tumors. mTOR has been shown to be required for proliferation of Müller glia progenitors in response to injury in chick retina.^36^ While multiple signaling pathways most likely drive proliferation and tumor growth in the *Tg(flk1:RFP)is18/+* model, our data are consistent with our previous analyses which suggest that mTOR signaling could influence activation of Müller glia regeneration pathways in the *Tg(flk1:RFP)is18/+* retina.

In summary, differential gene expression and cytological analyses of retinal dysplasia in adult *Tg(flk1:RFP)is18/+* zebrafish predisposed to optic pathway tumors reveal that ectopic proliferation is associated with elevated expression of markers in the VEGF, Leptin, and mTOR pathways. The causative molecular mechanism inducing retinal dysplasia and tumor formation in the *Tg(flk1:RFP)is18* line is not known. Our previous molecular characterization of the *Tg(flk1:RFP)is18* line revealed a 500-copy number <flk1:RFP> transgene inserted into a lncRNA. Isolation of a 147 kb deletion allele that removed the majority of the lncRNA gene did not cause tumor formation in heterozygous or homozygous adults.^37^ It is possible that the nature of this particular high copy number transgene is linked to deregulation of multiple signaling pathways, and this underlies proliferation and dysplasia. One possibility is that hundreds of copies of the *flk1* promoter alter global gene expression or a subset of vascular genes, including VEGF. Elevated levels of VEGF can cause vascular permeability and leakage,^7^ which would disrupt exchange of nutrients and metabolic waste. Alternatively, high levels of membrane targeted RFP expressed from the transgene in vascular endothelial cells may compromise vascular integrity, leading to defective retina function and metabolism. One of the most highly overexpressed genes in the *Tg(flk1:RFP)is18* dysplastic retina transcriptome is homologous to Plasmalemma Vesicle Associated Protein (*plvap*), a marker of vascular permeability. This supports the idea that defective vasculature and metabolic recycling could contribute to persistent stimulation of neural progenitor proliferation, leading to retinal dysplasia and nonmalignant tumor formation. Determining whether similar mechanisms regulate stem and progenitor cells throughout the nervous system will be important for a better understanding of CNS injury and repair.

## Supplementary Material

Supplemental data
