# Supplementary material for: Vascular Endothelial Growth Factor A and Leptin Expression Associated with Ectopic Proliferation and Retinal Dysplasia in Zebrafish Optic Pathway Tumors
Source: Zebrafish. 2017 Aug 1;14(4):343–56. doi: 10.1089/zeb.2016.1366 (PMC5549800; doi:10.1089/zeb.2016.1366)
Supplement: Supplemental data [file Supp_Data.zip › Supp_Table6.pdf]

© 2000-2016 QIAGEN. All rights reserved.

Supplementary Table S6. IPA *Tg(flk1:RFP)is18* Dysplastic retina Transcriptome

Ingenuity Canonical Pathways	-log (p-value)	Ratio	z-score	Down-regulated	No change	Up-regulated	No overlap with dataset	Molecules
Caveolar-mediated Endocytosis Signaling	2.09E00	2.11E-01	NaN	1/71 (1%)	0/71 (0%)	14/71 (20%)	56/71 (79%)	B2M,ITGB1,ACTB,ITGA8,ITGA6,EGF,ITGB7,ITGAE,ITGB2,ACTA2,FLNC,FLNA,INS,PTRF,ACTC1
EIF2 Signaling	2.53E01	4.08E-01	6.172	1/184 (1%)	0/184 (0%)	74/184 (40%)	109/184 (59%)	RPL11,RPL22,RPS23,RPLP2,RPL39,RPL35A,RPS11,RPS7,EIF3B,RPS13,RPL23A,INS,RPL19,RPL21,RPS2,PABPC1,RPL32,RRAS,RPL3,RPL29,EIF2S3,RPL12,EIF3E,RPL28,RPL9,EIF3M,RPL10A,RPS6,RPL15,RPL8,PPP1R15A,RPL10,RPL6,RPS15A,RPS25,RPL13A,RPSA,RPLP1,RPS3A,RPL36A,RPL22L1,RPL7A,EIF2S1,RPL7,EIF4E,RPS4X,RPS28,RPL35,RPL18A,RPS9,RPS5,RPS17,RPL18,RPL31,RPS24,RPS19,RPL4,RPL34,RPL17,RPS10,RPL30,RPS21,RPL23,RPS29,RPL27,RPS15,RPS16,RPS26,EIF2B1,RPS27A,RPL5,RPL37,EIF2AK2,RPS14,RPLP0
Endothelin-1 Signaling	4.87E00	2.21E-01	2.694	11/172 (6%)	0/172 (0%)	27/172 (16%)	134/172 (78%)	GUCY2C,CASP4,PLCH2,BRAF,MYC,HMOX1,JUN,CASP9,GNAT1,EDN1,GNA15,CASP1,ECE1,NOS2,CASP8,PRKCQ,CASP3,GUCY1A3,GUCY2D,RRAS,GNA12,ADCY3,PLA2G4C,GNAQ,MAPK9,ITPR1,PLA2G4A,PLA2G4D,PLCG2,ADCY1,GUCY2F,RARRES3,GNAT2,EDNRA,PTGER2,PTGS2,OPN1SW,CASP7
GADD45 Signaling	1.33E00	2.63E-01	NaN	0/19 (0%)	0/19 (0%)	5/19 (26%)	14/19 (74%)	GADD45B,GADD45G,CDK1,CDK2,CCNB1
Glioma Invasiveness Signaling	1.76E00	2.11E-01	1.732	2/57 (4%)	0/57 (0%)	10/57 (18%)	45/57 (79%)	RHOG,RHOQ,F2R,RRAS,RHOC,HMMR,CD44,RHOU,MMP2,PLAU,MMP9,TIMP2
HIF1α Signaling	1.61E00	1.76E-01	NaN	3/102 (3%)	0/102 (0%)	15/102 (15%)	84/102 (82%)	SLC2A1,RRAS,MMP14,MMP15,VEGFB,MAPK9,MMP13,MMP2,SLC2A3,PGF,VEGFA,JUN,EDN1,PROK1,EGLN3,NOS2,APEX1,MMP9
mTOR Signaling	7.64E00	2.51E-01	2.840	6/187 (3%)	0/187 (0%)	41/187 (22%)	140/187 (75%)	RPS3A,RPS23,FKBP1A,VEGFB,PRR5L,RPS11,EIF4E,RPS4X,PGF,VEGFA,RPS7,RPS28,HMOX1,RHOG,EIF3B,RPS13,PROK1,RPS9,INS,RHOU,RPS2,RPS6KA2,RPS17,RPS5,RPS24,RPS19,PRKCQ,DDIT4,RRAS,RHOC,RPS10,RPS21,EIF3E,RPS29,PPP2R5A,EIF3M,RPS6,DGKZ,RPS15,RPS16,RHOQ,RPS26,RPS27A,RPS15A,RPS25,RPSA,RPS14
Phototransduction Pathway	1.43E01	5.47E-01	NaN	19/53 (36%)	0/53 (0%)	10/53 (19%)	24/53 (45%)	RGS9,PDE6G,GUCY2C,GNB5,GUCA1B,GRK1,ARR3,GNB3,PDE6A,GNAT1,PDC,SAG,GNGT2,PDE6B,GUCY1A3,GUCY2D,GUCA1C,OPN1LW,PRKAR2A,CNGA1,CNGA3,CNGB1,RGR,GUCY2F,RGS9BP,GNAT2,OPN1SW,PDE6D,RCVRN
VEGF Signaling	1.43E00	1.74E-01	1.387	3/92 (3%)	0/92 (0%)	13/92 (14%)	76/92 (83%)	SH2D2A,PTPN6,RRAS,ACTB,ACTN2,VEGFB,EIF2S3,EIF2S1,PGF,VEGFA,ACTA2,PROK1,PLCG2,EIF2B1,KDR,ACTC1
