# Supplementary material for: Vascular Endothelial Growth Factor A and Leptin Expression Associated with Ectopic Proliferation and Retinal Dysplasia in Zebrafish Optic Pathway Tumors
Source: Zebrafish. 2017 Aug 1;14(4):343–56. doi: 10.1089/zeb.2016.1366 (PMC5549800; doi:10.1089/zeb.2016.1366)
Supplement: Supplemental data [file Supp_Data.zip › Supp_Data.pdf]

Supplementary Data

Supplementary Table S1. Princeton Gene Ontology Term Analysis and Annotated Genes in Each Term Category Downregulated in *Tg(flk1:RFP)is18* Dysplastic Retina

Supplementary Table S2. Princeton Gene Ontology Term Analysis and Annotated Genes in Each Term Category Upregulated in *Tg(flk1:RFP)is18* Dysplastic Retina

Supplementary Table S3. Princeton Gene Ontology Term Analysis and Annotated Genes in Each Term Category Downregulated in *Tg(flk1:RFP)is18* Tumor Tissue

Supplementary Table S4. Princeton Gene Ontology Term Analysis and Annotated Genes in Each Term Category Upregulated in *Tg(flk1:RFP)is18* Tumor Tissue

Supplementary Table S5. Zebrafish *Tg(flk1:RFP)is18* Dysplastic Retina and Retinal Tumor Three-Fold DGE Gene List with Human Homologs

Zebrafish genes with human homologs identified using BioMart and BLAST at Ensembl (http://useast.ensembl.org/Danio_rerio/Info/Index).

Supplementary Table S6. Ingenuity Pathway Analysis Canonical Pathways in *Tg(flk1:RFP)is18* Dysplastic Retina Transcriptome
