# Supplementary material for: Vascular Endothelial Growth Factor A and Leptin Expression Associated with Ectopic Proliferation and Retinal Dysplasia in Zebrafish Optic Pathway Tumors
Source: Zebrafish. 2017 Aug 1;14(4):343–56. doi: 10.1089/zeb.2016.1366 (PMC5549800; doi:10.1089/zeb.2016.1366)
Supplement: Supplemental data [file Supp_Data.zip › Supp_Table1.pdf]

Supplementary Table S1. A. Down *Tg(flk1:RFP)is18* Dysplastic retina GO terms and genes annotated to the term

Terms from the Process Ontology of gene_association.zfin with p-value <= 0.01   http://go.princeton.edu/tmp/1508523//query_results.html

Gene Ontology term	Cluster frequency	Genome frequency	Corrected P-value	FDR	FALSE Positives	Genes annotated to the term
cellular respiration	32 of 6538 genes, 0.5%	42 of 22409 genes, 0.2%	9.38E-07	0.00%	0	sdhdb, ndufb8, sdhb, uqcrb, idh3g, dlst, fh, mdh1b, park2, sfxn4, ndufv2, IDH2, idh3a, ndufa7, aco2, etfdh, ogdhb, ogdha, sdhc, dhtkd1, mt-co3, mt-nd2, ndufa5, ndufs1, idh3b, mt-nd4l, mt-co1, MDH2, sdhaf2, uqcrh, mt-nd5, mt-nd4
energy derivation by oxidation of organic compounds	39 of 6538 genes, 0.6%	61 of 22409 genes, 0.3%	4.53E-05	0.00%	0	sdhdb, zgc:77112, ndufb8, sdhb, aglb, uqcrb, idh3g, dlst, fh, mdh1b, mc4r, park2, sfxn4, ndufv2, IDH2, ppp1r3ca, idh3a, ndufa7, PHKB, phkg1b, aco2, etfdh, phkg1a, ogdhb, sdhc, ogdha, dhtkd1, mt-co3, mt-nd2, ndufa5, ndufs1, idh3b, uqcrh, mt-co1, MDH2, mt-nd4l, sdhaf2, mt-nd5, mt-nd4
ATP metabolic process	43 of 6538 genes, 0.7%	71 of 22409 genes, 0.3%	9.01E-05	0.00%	0	atp5h, ndufb8, atp5g3a, uqcrb, atp1a1a.2, atp1a1a.4, atp5d, atp1a1b, atp6v1ab, atp5c1, park2, atp6v1ba, atp5f1, ndufv2, atp5ib, ATP5B, msh2, atp5o, atp1a1a.3, atp5a1, tefm, ndufa7, mt-atp6, atp5g3b, abcb8, atp1a2a, atp1a3a, atp5j, atp6v1b2, supv3l1, mt-nd2, ndufs1, atp1a3b, atp6v1aa, atp5ia, uqcrh, sdhaf2, mt-co1, mt-nd4l, atp5l, mt-nd5, mt-atp8, mt-nd4
purine nucleoside monophosphate metabolic process	50 of 6538 genes, 0.8%	93 of 22409 genes, 0.4%	1.40E-03	0.00%	0	atp5h, ndufb8, atp5g3a, uqcrb, atp1a1a.2, atp1a1a.4, atp5d, gmps, ampd3a, atp1a1b, atp6v1ab, atp5c1, park2, atp6v1ba, adal, atp5f1, ndufv2, atp5ib, ATP5B, msh2, atp5o, atp1a1a.3, atp5a1, tefm, ndufa7, mt-atp6, impdh1a, ada, atp5g3b, abcb8, atp1a2a, atp1a3a, adss, atp5j, atp6v1b2, supv3l1, mt-nd2, ndufs1, atp1a3b, atp6v1aa, atp5ia, hprt1l, uqcrh, sdhaf2, mt-nd4l, mt-co1, atp5l, mt-nd5, mt-atp8, mt-nd4
purine ribonucleoside monophosphate metabolic process	50 of 6538 genes, 0.8%	93 of 22409 genes, 0.4%	0.0014	0.00%	0	atp5h, ndufb8, atp5g3a, uqcrb, atp1a1a.2, atp1a1a.4, atp5d, gmps, ampd3a, atp1a1b, atp6v1ab, atp5c1, park2, atp6v1ba, adal, atp5f1, ndufv2, atp5ib, ATP5B, msh2, atp5o, atp1a1a.3, atp5a1, tefm, ndufa7, mt-atp6, impdh1a, ada, atp5g3b, abcb8, atp1a2a, atp1a3a, adss, atp5j, atp6v1b2, supv3l1, mt-nd2, ndufs1, atp1a3b, atp6v1aa, atp5ia, hprt1l, uqcrh, sdhaf2, mt-nd4l, mt-co1, atp5l, mt-nd5, mt-atp8, mt-nd4
Golgi vesicle transport	25 of 6538 genes, 0.4%	36 of 22409 genes, 0.2%	0.00162	0.00%	0	blzf1, gosr1, golga1, cope, sec23a, mppe1, arcn1b, vps51, arcn1a, sec22a, trip11, stx6, sec24c, AP1M2, klhl12, sec22c, golga5, trappc4, trappc2, sec22ba, cog6, USO1, sec13, gcc1, cux1b
generation of precursor metabolites and energy	54 of 6538 genes, 0.8%	104 of 22409 genes, 0.5%	0.00219	0.00%	0	sdhdb, zgc:77112, ndufb8, ndufs4, sdhb, eno2, tpi1a, aglb, uqcrb, idh3g, pfkmb, dlst, fh, mdh1b, mc4r, park2, sfxn4, pgam1b, pdha1a, ndufv2, pdha1b, IDH2, ppp1r3ca, idh3a, tefm, PHKB, ldhbb, ndufa7, phkg1b, aco2, phkg1a, etfdh, ogdhb, sdhc, ogdha, bpgm, dhtkd1, mt-co3, ENO4, pgam2, mt-nd2, ndufa5, ndufs1, idh3b, uqcrh, sdhaf2, MDH2, mt-co1, mt-nd4l, mt-nd5, eno1b, gpib, mt-co2, mt-nd4
electron transport chain	17 of 6538 genes, 0.3%	21 of 22409 genes, 0.1%	0.00317	0.00%	0	ndufa7, ndufb8, ndufs4, etfdh, uqcrb, mt-co3, mt-nd2, ndufa5, ndufs1, park2, uqcrh, mt-nd4l, sdhaf2, mt-nd5, ndufv2, mt-nd4, mt-co2
ATP biosynthetic process	22 of 6538 genes, 0.3%	31 of 22409 genes, 0.1%	0.00432	0.00%	0	atp5h, mt-atp6, atp5g3a, atp5g3b, atp1a1a.2, atp1a1a.4, atp5d, atp1a2a, atp1a3a, atp5j, atp1a1b, atp1a3b, atp5c1, atp5ia, atp5l, atp5f1, mt-atp8, atp5ib, ATP5B, atp5o, atp1a1a.3, atp5a1
purine ribonucleotide biosynthetic process	53 of 6538 genes, 0.8%	105 of 22409 genes, 0.5%	0.00841	0.00%	0	gucy1a3, guca1d, atp5h, atp5g3a, adra2b, adsl, nos1, guca1e, atp1a1a.2, atp1a1a.4, atp5d, gmps, ampd3a, pank2, atp1a1b, gc2, nos2a, mc4r, atp5c1, atp5f1, nme8, atp5ib, ATP5B, atp5o, paqr7b, guca1g, gnal, atp1a1a.3, atp5a1, rp2, pank1b, mt-atp6, impdh1a, gc3, dcakd, atp5g3b, nme6, GUCY2C, atp1a2a, coasy, pth2r, nme2a, atp1a3a, GUCA1A, adss, atp5j, atp1a3b, atp5ia, hprt1l, pank1a, atp5l, mt-atp8, gucy1b3
purine nucleoside biosynthetic process	38 of 6538 genes, 0.6%	68 of 22409 genes, 0.3%	0.00906	0.00%	0	atp5h, atp5g3a, atp1a1a.2, atp1a1a.4, atp5d, gmps, pank2, amd1, atp1a1b, atp5c1, atp5f1, nme8, atp5ib, ATP5B, atp5o, atp1a1a.3, atp5a1, rp2, pank1b, mt-atp6, impdh1a, dcakd, ada, atp5g3b, nme6, atp1a2a, coasy, nme2a, atp1a3a, adss, atp5j, atp1a3b, atp5ia, hprt1l, MAT2B, pank1a, atp5l, mt-atp8
purine ribonucleoside biosynthetic process	38 of 6538 genes, 0.6%	68 of 22409 genes, 0.3%	0.00906	0.00%	0	atp5h, atp5g3a, atp1a1a.2, atp1a1a.4, atp5d, gmps, pank2, amd1, atp1a1b, atp5c1, atp5f1, nme8, atp5ib, ATP5B, atp5o, atp1a1a.3, atp5a1, rp2, pank1b, mt-atp6, impdh1a, dcakd, ada, atp5g3b, nme6, atp1a2a, coasy, nme2a, atp1a3a, adss, atp5j, atp1a3b, atp5ia, hprt1l, MAT2B, pank1a, atp5l, mt-atp8
