# Supplementary material for: Vascular Endothelial Growth Factor A and Leptin Expression Associated with Ectopic Proliferation and Retinal Dysplasia in Zebrafish Optic Pathway Tumors
Source: Zebrafish. 2017 Aug 1;14(4):343–56. doi: 10.1089/zeb.2016.1366 (PMC5549800; doi:10.1089/zeb.2016.1366)
Supplement: Supplemental data [file Supp_Data.zip › Supp_Table2.pdf]

Supplementary Table S2. Up *Tg(flk1:RFP)is18* Dysplastic retina GO terms and genes annotated to the term

Terms from the Process Ontology of gene_association.zfin with p-value <= 0.01 http://go.princeton.edu/cgi-bin/JobWatch.pl?id=7417909

Gene Ontology term	Cluster frequency	Genome frequency	Corrected P-value	FDR	FALSE Positives	Genes annotated to the term
translation	162 of 6701 genes, 2.4%	311 of 22409 genes, 1.4%	4.50E-13	0.00%	0	eif3i, tcea2, rpl12, pdcd11, eef1b2, etf1, rps15a, RPS11, mrps6, eif3eb, eprs, denr, eif4ebp2, eef1db, rpl10, farsa, sars, rpl19, eif3ba, RPS6, rpl22, rpl18, rpl27, rpl17, rps15, trmt6, nars, aars, qars, rps4x, iars, rpl23a, larsa, rps26, eif4a3, eef1g, tcea3, rpl23, eif3c, rpl37, eif3d, rps16, eef2a.1, eif2b4, eif2s2, mrpl20, rpl5a, rpsa, rpl4, eif5a, eif6, rpl39, rplp0, eif4a1a, eif3s6ip, rpl5b, rpl13a, eef1a1a, RPS20, rps3a, cars, rpl36a, rpl3, eif2s3, gra, dars, mif4gdb, RPL38, larsb, eif3s10, eif4ebp1, eif2b2, eif1axa, rpl36, TNRC6A, rplp2, rpl18a, RPS17, rps27.1, magoh, caprin2, rps29, mettl17, WARS, rps23, farsb, yars, rps8a, eif2ak1, rbm8a, mif4gda, rpl9, EIF3F, eif3ha, eif3m, rplp1, rpl30, rpl10a, rps26l, eif5a2, eef2b, rps7, eif3ea, rps21, EIF3K, uba52, tars, eif2ak3, rpl14, vars, rps14, rps5, ticrr, hars, faua, rpl28, rps2, eif4eb, impact, rpl26, rars, rpp30, rpl8, eef2k, rps13, RPL31, rpl15, rps9, rps27a, ngdn, rpl22l1, kars, gars, eef1a1l1, rpl35a, eif3g, mars, eif2d, rps27.2, eif1axb, rpl13, rpl35, rps28, eif2b1, rps18, ell, rpl32, eif4bb, rpl21, gfm2, EIF1B, eif2b3, brf1a, rps24, rpl34, rps19, qki2, rplp2l, eif4ebp3l, rpl6, rpl11, rps3
ribonucleo-protein complex biogenesis	65 of 6701 genes, 1.0%	107 of 22409 genes, 0.5%	9.59E-08	0.00%	0	eif3i, gnl3l, rps8a, gtpbp4, eif3eb, sdad1, tsr2, dcaf13, EIF3F, nol11, wdr55, eif3ha, eif3m, rrs1, rsl24d1, wdr12, utp15, snrpc, eif3ba, RPS6, rps7, eif3ea, rps21, EIF3K, coil, pwp2h, tbl3, dimt1l, nip7, rrp36, ddx51, dkc1, ticrr, MRTO4, gnl1, icln, LUC7L3, eif3c, HEATR1, nop10, eif3d, pes, tsr1, rpsa, rcl1, eif6, rplp0, eif3g, eif3s6ip, shq1, gar1, nhp2, nop2, fbl, rpl7a, sart3, WBP11, rps28, rps18, nhp2l1b, rps24, ftsj, gnl3, eif3s10, RPS17
regulation of translational initiation	20 of 6701 genes, 0.3%	23 of 22409 genes, 0.1%	5.62E-05	0.00%	0	eif3i, eif3g, eif3s6ip, eif3ba, eif3c, eif3ea, EIF3K, eif2b1, eif3d, gra, eif2b4, trmt6, eif3eb, eif2b2, eif4ebp1, eif3s10, eif4ebp3l, EIF3F, eif3m, eif4ebp2
ribosome biogenesis	45 of 6701 genes, 0.7%	74 of 22409 genes, 0.3%	9.28E-05	0.00%	0	dkc1, MRTO4, gnl1, HEATR1, gnl3l, nop10, pes, rps8a, gtpbp4, tsr1, sdad1, tsr2, dcaf13, rpsa, nol11, wdr55, rcl1, eif6, rplp0, rrs1, wdr12, rsl24d1, gar1, utp15, nhp2, fbl, nop2, rpl7a, RPS6, WBP11, rps28, rps7, rps21, pwp2h, rps18, tbl3, dimt1l, nhp2l1b, ftsj, rps24, gnl3, nip7, rrp36, RPS17, ddx51
translational initiation	38 of 6701 genes, 0.6%	60 of 22409 genes, 0.3%	0.00023	0.00%	0	ticrr, eif3i, eif4eb, eif3c, eif3d, eif2b4, eif2s2, eif2ak1, eif3eb, EIF3F, eif3ha, denr, eif4ebp2, eif3m, eif5a, eif6, eif4a1a, eif3s6ip, eif3g, eif2d, eif1axb, eif3ba, eif3ea, EIF3K, eif2b1, eif2s3, gra, eif4bb, EIF1B, trmt6, eif2ak3, eif2b3, brf1a, eif1axa, eif2b2, eif4ebp1, eif3s10, eif4ebp3l

vasculature development	136 of 6701 genes, 2.0%	324 of 22409 genes, 1.4%	0.00609	0.00%	0	vhl, figf, LAMA1, colec12, fzd6, meis1, tnfrsf19, NTN4, jmjd6, frzb, rasgrp4, ephb4a, bmpr2a, fermt2, alox5ap, erg, notch1b, msna, etv2, ANGPTL1, sars, vegfaa, appb, smad5, foxc1a, jak2a, nus1, fzd8a, lpar1, acvrl1, foxo1b, lgals2a, foxm1, flt4, hmgcrb, dla, ELMO1, rnf213a, sox7, amotl2a, wdr43, wnt2bb, flt1, pak2a, calcrla, dll4, nrp2b, birc5a, ptgs1, amot, sox18, gng2, tbx6, itgav, lpar6a, tie1, cxcl12b, gnb2l1, ctnnb1, rab13, lama4, SHC1, snx5, pdgfrb, egfl7, sat1b, vegfab, ptprb, fzd9b, TAL1, dlc, igfbp7, mb, kdrl, rps29, twsg1b, PLXND1, smo, hexb, fli1a, ppih, loxl2a, trpc1, dzip1, PDGFB, pbx1a, pld1a, eif3ha, ecscr, per2, FOXJ2, utp15, pggt1b, foxc1b, cdh5, fbxw11a, unc5b, lmo2, notch3, fzd2, spry2, loxl2b, smarcal1, twsg1a, s1pr1, sfrp1a, ctsz, tinagl1, fli1b, runx1, kdr, pak2b, angpt1, plxnb2a, itga2b, ephx2, vap, fzd7b, nrp2a, notch2, CFI, s1pr2, rbpjb, fbn2b, mcamb, robo4, f3b, fmnl3, ell, tp53, ptprja, lef1, itgb8, gata2a, ilk, plekhh1
positive regulation of cell death	25 of 6701 genes, 0.4%	37 of 22409 genes, 0.2%	0.00666	0.00%	0	casp9, fas, bnip4, caspa, phlda3, tradd, bbc3, bnip3lb, zgc:73226, dusp6, bmf2, tnfrsfa, baxa, pmaip1, fadd, rela, casp3a, bida, tp53, caspb, rest, scrib, CASP8, bnip3la, baxb
positive regulation of apoptotic process	25 of 6701 genes, 0.4%	37 of 22409 genes, 0.2%	0.00666	0.00%	0	casp9, fas, bnip4, caspa, phlda3, tradd, bbc3, bnip3lb, zgc:73226, dusp6, bmf2, tnfrsfa, baxa, pmaip1, fadd, rela, casp3a, bida, tp53, caspb, rest, scrib, CASP8, bnip3la, baxb
positive regulation of programmed cell death	25 of 6701 genes, 0.4%	37 of 22409 genes, 0.2%	0.00666	0.00%	0	casp9, fas, bnip4, caspa, phlda3, tradd, bbc3, bnip3lb, zgc:73226, dusp6, bmf2, tnfrsfa, baxa, pmaip1, fadd, rela, casp3a, bida, tp53, caspb, rest, scrib, CASP8, bnip3la, baxb
regulation of apoptotic process	85 of 6701 genes, 1.3%	185 of 22409 genes, 0.8%	0.00759	0.00%	0	phlda3, rnf2, tnfsf10l, akt2, traf4a, rela, kdm5c, mcl1b, smo, msxb, si:ch211-218c6.8, bcl2l10, BCL2, tp73, rad21a, casp9, park7, FOXJ2, zgc:158343, bbc3, bnip3lb, ddt, dusp6, foxc1b, bmf2, tnfrsfa, PPM1K, card9, foxc1a, pmaip1, fadd, casp3a, dusp1, caspb, apex1, rest, esco2, foxo1b, pycard, mcl1a, bnip4, si:dkey-10c21.1, prnprs3, tradd, baxa, spast, dusp2, egr1, HEATR1, traf3, nod2, perp, mcm5, nes, bida, apaf1, scrib, pak2a, snx7, pak2b, wt1b, bcor, bnip3la, birc5a, baxb, dlg5a, fas, egr2a, caspa, GRINA, epas1b, zgc:73226, casp8l2, birc2, bcl2l1, EGR3, gbp3, ctnnb1, tp53, lef1, zgc:174906, plrg1, boka, CASP8, aurkb

regulation of programmed cell death	86 of 6701 genes, 1.3%	188 of 22409 genes, 0.8%	0.00829	0.00%	0	phlda3, rnf2, tnfsf10l, akt2, traf4a, rela, kdm5c, mcl1b, smo, msxb, si:ch211-218c6.8, bcl2l10, BCL2, tp73, rad21a, casp9, park7, FOXJ2, zgc:158343, bbc3, bnip3lb, ddt, dusp6, foxc1b, bmf2, tnfrsfa, cct3, PPM1K, card9, foxc1a, pmaip1, fadd, casp3a, dusp1, caspb, apex1, rest, esco2, foxo1b, pycard, mcl1a, bnip4, si:dkey-10c21.1, prnprs3, tradd, baxa, spast, dusp2, egr1, HEATR1, traf3, nod2, perp, mcm5, nes, bida, apaf1, scrib, pak2a, snx7, pak2b, wt1b, bcor, bnip3la, birc5a, baxb, dlg5a, fas, egr2a, caspa, GRINA, epas1b, zgc:73226, casp8l2, birc2, bcl2l1, EGR3, gbp3, ctnnb1, tp53, lef1, zgc:174906, plrg1, boka, CASP8, aurkb
blood vessel development	121 of 6701 genes, 1.8%	284 of 22409 genes, 1.3%	0.00901	0.00%	0	vhl, figf, LAMA1, TAL1, colec12, fzd6, igfbp7, meis1, dlc, mb, tnfrsf19, NTN4, rps29, kdrl, jmjd6, twsg1b, PLXND1, ephb4a, fermt2, smo, alox5ap, hexb, fli1a, ppih, trpc1, loxl2a, erg, PDGFB, dzip1, notch1b, pld1a, msna, etv2, ecscr, per2, FOXJ2, utp15, ANGPTL1, sars, pggt1b, foxc1b, vegfaa, cdh5, smad5, fbxw11a, foxc1a, jak2a, unc5b, lmo2, notch3, nus1, spry2, lpar1, loxl2b, smarcal1, acvrl1, foxo1b, twsg1a, lgals2a, ctsz, foxm1, tinagl1, flt4, hmgcrb, dla, ELMO1, rnf213a, fli1b, runx1, sox7, amotl2a, wdr43, wnt2bb, flt1, pak2a, calcrla, kdr, pak2b, dll4, nrp2b, angpt1, birc5a, ptgs1, amot, itga2b, plxnb2a, ephx2, vap, sox18, gng2, tbx6, itgav, nrp2a, lpar6a, rbpjb, tie1, CFI, cxcl12b, fbn2b, robo4, mcamb, ell, fmnl3, ctnnb1, gnb2l1, tp53, lef1, ptprja, itgb8, rab13, lama4, SHC1, snx5, gata2a, pdgfrb, egfl7, sat1b, vegfab, ilk, plekhh1, ptprb
regulation of cell death	87 of 6701 genes, 1.3%	191 of 22409 genes, 0.9%	0.00902	0.00%	0	phlda3, rnf2, tnfsf10l, akt2, traf4a, rela, kdm5c, mcl1b, smo, msxb, si:ch211-218c6.8, rilpl1, bcl2l10, BCL2, tp73, rad21a, casp9, park7, FOXJ2, zgc:158343, bbc3, bnip3lb, ddt, dusp6, foxc1b, bmf2, tnfrsfa, cct3, PPM1K, card9, foxc1a, pmaip1, fadd, casp3a, dusp1, caspb, apex1, rest, esco2, foxo1b, pycard, mcl1a, bnip4, si:dkey-10c21.1, prnprs3, tradd, baxa, spast, dusp2, egr1, HEATR1, traf3, nod2, perp, mcm5, nes, bida, apaf1, scrib, pak2a, snx7, pak2b, wt1b, bcor, bnip3la, birc5a, baxb, dlg5a, fas, egr2a, caspa, GRINA, epas1b, zgc:73226, casp8l2, birc2, bcl2l1, EGR3, gbp3, ctnnb1, tp53, lef1, zgc:174906, plrg1, boka, CASP8, aurkb
