# Supplementary material for: Vascular Endothelial Growth Factor A and Leptin Expression Associated with Ectopic Proliferation and Retinal Dysplasia in Zebrafish Optic Pathway Tumors
Source: Zebrafish. 2017 Aug 1;14(4):343–56. doi: 10.1089/zeb.2016.1366 (PMC5549800; doi:10.1089/zeb.2016.1366)
Supplement: Supplemental data [file Supp_Data.zip › Supp_Table3.pdf]

Supplementary Table S3. Down *Tg(flk1:RFP)is18* retinal Tumor GO terms and genes annotated to the term

Terms from the Process Ontology of gene_association.zfin with p-value <= 0.01 http://go.princeton.edu/tmp/3167671//query_results.html

Gene Ontology term	Cluster frequency	Genome frequency	Corrected P-value	FDR	FALSE Positives	Genes annotated to the term
sensory perception of light stimulus	45 of 7259 genes, 0.6%	71 of 22409 genes, 0.3%	2.00E-04	0.00%	0	rom1a, prph2a, pde6h, prph2b, pde6d, nyx, rpe65a, rgrb, si:ch211-37p17.5, cx55.5, pcdh15b, rom1b, arr3a, opn4xb, rho, rgra, gucy2f, slc17a6b, pde6g, opn4xa, GPR179, dlat, zgc:112320, tmtopsb, opn1mw2, rhol, opn4b, grk7b, opn1mw1, opn1sw2, prph2l, opn1sw1, valopa, lum, opn4.1, opn1lw2, vsx1, unc119b, opn1mw4, arl6, crx, valopb, nphp3, gnat2, grk7a
visual perception	44 of 7259 genes, 0.6%	70 of 22409 genes, 0.3%	4.00E-04	0.00%	0	rom1a, prph2a, pde6h, prph2b, pde6d, nyx, rpe65a, rgrb, si:ch211-37p17.5, pcdh15b, rom1b, arr3a, opn4xb, rho, rgra, gucy2f, slc17a6b, pde6g, opn4xa, GPR179, dlat, zgc:112320, tmtopsb, opn1mw2, rhol, opn4b, grk7b, opn1mw1, opn1sw2, prph2l, opn1sw1, valopa, lum, opn4.1, opn1lw2, vsx1, unc119b, opn1mw4, arl6, crx, valopb, nphp3, gnat2, grk7a
cellular respiration	29 of 7259 genes, 0.4%	42 of 22409 genes, 0.2%	3.11E-03	0.00%	0	sdhdb, ndufa7, aco2, sdhb, uqcrb, ogdhb, idh3g, sdhc, ogdha, mt-co3, dhtkd1, mt-nd2, ndufs1, ndufa5, idh3b, mdh1b, park2, uqcrh, sdhaf2, MDH2, mt-co1, mt-nd4l, mtfr1, sdha, ndufv2, mt-nd5, IDH2, idh3a, mt-nd4
ion transport	278 of 7259 genes, 3.8%	680 of 22409 genes, 3.0%	3.67E-03	0.00%	0	scn4ba, cacna1ba, atp5h, ap1b1, kcnj14, atp11a, trpc5b, slc7a9, atp1a1a.4, nnt, gria2b, KCNG3, cacng8b, asic4b, atp5ib, asic2, slc16a6a, slc5a12, atp6v0d1, cacna1da, slc34a2a, slc13a2, atp1a2a, slc12a10.3, cftr, cnga3a, KCTD9, SLC31A2, slc25a42, slc25a22, ttyh2l, slc22a4, atp2b2, scn3b, mt-atp8, CNGB3, cacnb4a, grin2ca, cx35b, mcu, cacna1sa, pitpnb, slc26a2, atp1b3b, atp5c1, atp6v1ba, atp5f1, KCNH4, slc26a11, VDAC3, cacna1c, cnga5, atp1b4, gabrb2, cacng7b, nalcn, kcnc1b, glrbb, glra1, atp1a3a, kcnd3, slc8a3, gabrb3, atp6v0cb, gria2a, scn4aa, cacna1aa, slc25a28, slc26a5, surf1, slc4a2b, cox5ab, asic4a, gabra5, slc8a1b, si:dkey-28b4.8, CACNG1, atp2b1a, sco2, ca2, atp2b3a, grin1b, trpc1, cacng2b, slc7a4, gria3a, kcnh5a, atp1a1a.3, grin2db, atp8b2, cacnb3a, GRIN3B, micu1, kcnq5b, kcnk1a, atp6v0b, GLRA3, gria4a, ankha, ATP5J, slc9a6b, CNGA1, slc30a9, slc17a6a, fth1b, kcnq5a, atp2a3, slc39a10, KCNH6, kctd8, slc4a4a, slc38a6, atp2a2a, kcnma1a, mpc2, tpcn1, slc9a3.2, gabrd, kcnh3, tcirg1, atp1b2a, slc17a6b, slc4a11, ATP5B, atp5a1, CACNA1I, atp6v1c1a, kcnma1b, cacna1f, scn8ab, rhcga, atp2c1, p2rx8, sfxn5b, MPC1, si:rp71-39b20.4, SLC26A6, slc1a2b, zgc:171544, atp1a3b, atp5ia, slc40a1, cngb1a, kcnc3b, grin2aa, slc8a4b, cox17, kcnb2, gabra6b, atp6v0a1a, scn8aa, kcnd2, abcc4, trpm1a, gabra3, kcnv2b, abcc9, grin1a, kcnn3, scn12aa, hcn3, kcnd1, slc6a9, atp8a1, scn1ba, SLC39A14, slc8a4a, slc1a2a, cacng7a, slc38a2, slc1a7a, ryr3, atp8a2, SLC16A1, cacna1g, zgc:173594, cacna1ab, cacng3a, trpa1b, hcn4l, atp1a1a.2, slc4a4b, atp9b, gabrr1, TMEM38B, gria3b, aqp9b, KCNG1, slc26a6l, slc9a8, slc9a7, cacnb2b, SLC39A8, slc4a5, slc8a1a, grid2, atp5o, gdnfa, cln3, cnga3b, nr3c1, slc6a4b, slc1a8a, slc30a8, hcn4, atp1a1a.5, SLC9A1, glrba, clcn5, slc12a2, zgc:171453, tmem163b, slc1a8b, p2rx7, itpr2, slc7a2, gltpd1, KCNAB1, kcnk3, zgc:162160, atp1a1b, si:ch211-39a7.1, slc6a6b, si:dkey-106c17.3, slc1a9, slc4a10b, cacnb2a, slc10a1, gabrr2b, vdac1, kcnc3a, trpm3, scn2b, atp1b1b, gabrg2, slc39a5, slc13a1, slc30a5, atp5g3b, si:dkey-162b23.4, scn1a, KCNT1, atp11c, gabrr2a, trpm7, gria1a, gabra1, atp2b1b, slc30a10, atp2b3b, slc9a2, atp1b2b, kcnj11l, slc9a6a, gabra6a, grik1a, atp1b1a, si:ch73-380n15.2, kctd12b, cacnb3b, mt-atp6, slc8a2b, kcnk2b, KCNJ5, kctd12.1, atp1b3a, kcnf1b, kcnv2a, kcnk9, gria4b, slc25a39, KCNQ1, kctd16b, CLCN2, trpm1b, clcn3, SLC16A7, KCNA1
monovalent inorganic cation transport	118 of 7259 genes, 1.6%	256 of 22409 genes, 1.1%	0.00772	0.00%	0	atp5h, ap1b1, surf1, KCNAB1, cox5ab, kcnj14, kcnb2, asic4a, atp1a1a.4, scn8aa, atp6v0a1a, kcnk3, zgc:162160, kcnd2, atp1a1b, slc6a6b, ca2, si:dkey-106c17.3, nnt, slc10a1, kcnc3a, kcnv2b, KCNG3, asic4b, abcc9, atp5ib, kcnh5a, atp1a1a.3, asic2, scn12aa, kcnn3, atp1b1b, kcnd1, hcn3, slc5a12, slc13a1, atp6v0d1, scn1ba, kcnq5b, atp5g3b, slc8a4a, kcnk1a, atp6v0b, slc38a2, slc13a2, atp1a2a, scn1a, KCNT1, ATP5J, slc9a6b, cnga3a, CNGA1, slc17a6a, scn3b, mt-atp8, kcnq5a, CNGB3, KCNH6, slc4a4a, hcn4l, slc9a2, slc38a6, atp1a1a.2, slc9a6a, kcnma1a, atp1b2b, kcnj11l, TMEM38B, slc26a6l, KCNG1, slc9a3.2, atp1b1a, atp1b3b, atp5c1, kcnh3, atp6v1ba, tcirg1, slc9a8, atp5f1, atp1b2a, KCNH4, slc9a7, slc8a1a, slc17a6b, ATP5B, atp5o, cnga5, atp5a1, atp1b4, cnga3b, mt-atp6, atp6v1c1a, kcnma1b, kcnk2b, scn8ab, rhcga, KCNJ5, atp1b3a, hcn4, kcnv2a, kcnf1b, kcnk9, atp1a1a.5, SLC9A1, si:rp71-39b20.4, kcnc1b, atp1a3a, kcnd3, slc8a3, atp1a3b, atp6v0cb, KCNQ1, atp5ia, cngb1a, zgc:171453, scn4aa, kcnc3b, KCNA1
