# Supplementary material for: Vascular Endothelial Growth Factor A and Leptin Expression Associated with Ectopic Proliferation and Retinal Dysplasia in Zebrafish Optic Pathway Tumors
Source: Zebrafish. 2017 Aug 1;14(4):343–56. doi: 10.1089/zeb.2016.1366 (PMC5549800; doi:10.1089/zeb.2016.1366)
Supplement: Supplemental data [file Supp_Data.zip › Supp_Table4.pdf]

Supplementary Table S4. Up *Tg(flk1:RFP)is18* retinal Tumor GO terms and genes annotated to the term

Terms from the Process Ontology of gene_association.zfin with p-value <= 0.01 http://go.princeton.edu/tmp/2366867//query_results.html

Gene Ontology term	Cluster frequency	Genome frequency	Corrected P-value	FDR	FALSE Positives	Genes annotated to the term
ribonucleo-protein complex biogenesis	82 of 8294 genes, 1.0%	107 of 22409 genes, 0.5%	1.99E-13	0.00%	0	eif3i, exosc6, gnl2, gnl3l, rps8a, gtpbp4, eif3eb, tsr2, sdad1, prpf8, dcaf13, wdr36, wdr55, nol11, EIF3F, eif3m, eif3ha, rrnad1, rrs1, wdr12, rsl24d1, utp15, snrpc, RPS6, eif3ba, rps7, mphosph10, rps21, patl1, eif3ea, coil, EIF3K, pwp2h, tbl3, noc4l, dimt1l, rrp36, prpf31, nip7, ticrr, dkc1, MRTO4, eif3jb, icln, eif3c, dicer1, c1d, HEATR1, nop10, eif3d, bop1, pes, tsr1, rpsa, eif6, krr1, rplp0, eif3s6ip, eif3g, shq1, gar1, ybey, nhp2, fbl, nop2, rpl7a, prmt7, sart3, WBP11, rps28, rps18, naf1, nhp2l1b, ftsj, rps24, gnl3, tarbp2, eif3s10, eif3ja, bms1l, lsg1, RPS17
translation	184 of 8294 genes, 2.2%	311 of 22409 genes, 1.4%	3.86E-12	0.00%	0	eif3i, tcea2, mrps15, rpl12, pdcd11, eef1b2, rps15a, etf1, RPS11, mrps6, eif3eb, MRPL32, casc3, eprs, eif4ebp2, denr, eef1db, rpl10, sars, farsa, rpl19, RPS6, eif3ba, rpl22, rpl18, rpl27, rpl17, rps15, trmt6, eif4e2rs1, nars, qars, aars, rps4x, rpl23a, iars, larsa, rps26, eif4a3, eif3jb, mrps24, eef1g, cnot10, rpl23, eif3c, rpl37, eif3d, rps16, eef2a.1, eif2b4, eif2s2, eif4ebp3, mrpl20, eif4a2, rpl5a, rpsa, MTRF1, rpl4, eif5a, eif6, sepsecs, rpl39, rplp0, eif3s6ip, eif4a1a, rpl5b, eif5, rpl13a, RPS20, rps3a, tcea1, cars, rpl36a, rpl3, eif2s3, gra, eef1da, dars, tufm, RPL38, larsb, tarbp2, rpl36, eif2b2, eif3s10, eif4ebp1, eif3ja, rplp2, rpl18a, rps27.1, RPS17, EIF2A, magoh, caprin2, rps29, EIF1AD, WARS, rps23, farsb, yars, rps8a, rqcd1, eif2ak1, rbm8a, rpl9, mif4gda, EIF3F, ncbp2, eif3ha, eif3m, rplp1, rpl30, rpl10a, rps26l, eif5a2, eef2b, rps7, rps21, eif3ea, EIF3K, uba52, dars2, tars, eif4e1c, vars, eif2ak3, rpl14, rps5, rps14, RNF8, mrrf, ticrr, mrpl37, hars, faua, rpl28, rps2, eif4eb, impact, rars, rpl26, rpp30, rpl8, eef2k, mrps10, rps13, RPL31, rpl15, rps9, rps27a, ngdn, mrpl13, kars, rpl22l1, eef1a1l1, gars, rpl35a, eif3g, mars, mtif2, rps27.2, eif1axb, rpl13, rpl35, rps28, eif4ea, eif2b1, rps18, rpl32, eif4bb, rpl21, EIF1B, eif2b3, rps24, rps19, cnot7, mrps18a, rpl34, rplp2l, eif4ebp3l, qki2, rpl6, rpl11, rps3
mitotic cell cycle	101 of 8294 genes, 1.2%	157 of 22409 genes, 0.7%	9.48E-09	0.00%	0	plk1, mastl, fbxl15, rnf2, ndc80, gnl2, snx33, cdca8, cdc26, tpx2, mad1l1, pafah1b1b, smc2, fam83d, actr8, zwilch, pafah1b1a, ANAPC4, ncaph, bora, tp73, snap25a, katna1, nabp1a, wee1, rad21a, mcmbp, cdc25, smarcb1a, nek8, ccnf, ncapd3, ccnd1, ska3, snx18a, TXNL4B, stil, SETDB2, nusap1, aspm, chek1, kif23, dctn2, prkci, cep63, sass6, klhl21, tfdp1a, cdk5rap3, dtl, esco2, ncapg, chfr, smarcal1, vangl2, ube2i2, anapc10, ttk, ticrr, cdc16, smc3, fbxo5, mad2l1, CDKN1C, cenph, wee2, haus6, ints3, mcm5, seh1l, ppp2r4, setd8a, pold1, txnl4a, dscc1, spc24, arl8ba, rcc2, ube2i, cdk1, usp37, ska1, tipin, antxr2a, kdm8, ccnb3, cenpn, tmpoa, tp53, RBBP8, swap70b, ncapd2, chaf1b, fsd1, wnt11, gnl3, zw10, ercc6l, nuf2, rhoab, aurkb
mitotic cell cycle process	87 of 8294 genes, 1.0%	131 of 22409 genes, 0.6%	2.30E-08	0.00%	0	mastl, fbxl15, ndc80, gnl2, snx33, cdca8, cdc26, tpx2, mad1l1, pafah1b1b, smc2, fam83d, actr8, zwilch, pafah1b1a, ANAPC4, ncaph, bora, tp73, snap25a, katna1, nabp1a, wee1, rad21a, mcmbp, smarcb1a, nek8, ccnf, ncapd3, ccnd1, ska3, snx18a, TXNL4B, stil, SETDB2, nusap1, chek1, kif23, dctn2, prkci, cep63, sass6, klhl21, dtl, ncapg, chfr, vangl2, ube2i2, anapc10, ttk, ticrr, smc3, fbxo5, mad2l1, cenph, wee2, haus6, ints3, seh1l, ppp2r4, setd8a, txnl4a, dscc1, spc24, arl8ba, rcc2, ube2i, usp37, ska1, tipin, antxr2a, kdm8, ccnb3, cenpn, tmpoa, tp53, RBBP8, swap70b, ncapd2, fsd1, wnt11, gnl3, zw10, ercc6l, nuf2, rhoab, aurkb
regulation of translational initiation	23 of 8294 genes, 0.3%	23 of 22409 genes, 0.1%	3.52E-07	0.00%	0	eif3i, eif3s6ip, eif3g, eif3jb, eif3ba, eif3c, eif3ea, EIF3K, eif2b1, eif3d, gra, eif2b4, eif4ebp3, trmt6, eif3eb, eif4ebp1, eif2b2, eif3s10, eif3ja, eif4ebp3l, EIF3F, eif4ebp2, eif3m

ribosome biogenesis	55 of 8294 genes, 0.7%	74 of 22409 genes, 0.3%	2.09E-07	0.00%	0	dkc1, MRTO4, exosc6, gnl2, HEATR1, c1d, gnl3l, nop10, pes, bop1, rps8a, gtpbp4, tsr1, tsr2, sdad1, rpsa, dcaf13, wdr36, wdr55, nol11, eif6, krr1, rplp0, rrnad1, rrs1, wdr12, rsl24d1, utp15, gar1, ybey, nhp2, fbl, nop2, RPS6, rpl7a, WBP11, rps7, rps28, mphosph10, rps21, pwp2h, rps18, naf1, tbl3, dimt1l, nhp2l1b, noc4l, rps24, ftsj, gnl3, bms1l, lsg1, rrp36, nip7, RPS17
cell division	122 of 8294 genes, 1.5%	208 of 22409 genes, 0.9%	5.02E-07	0.00%	0	mastl, ccnb2, prc1b, prc1a, pdgfaa, ndc80, gnl2, cdc73, snx33, cdca8, dhx8, cdc26, tpx2, mad1l1, pafah1b1b, smc2, fam83d, cdc40, actr8, ercc4, zwilch, pafah1b1a, ANAPC4, cdc42l, ccne2, ncaph, bora, hells, snap25a, katna1, wee1, rad21a, CCNA2, maea, mcmbp, nek8, ccnf, ccdc124, zgc:86764, ncapd3, ccnd1, vegfaa, ska3, snx18a, TXNL4B, trip13, SETDB2, nusap1, kif23, dctn2, notch3, cdc42, cep63, sass6, klhl21, zgc:56231, cdc5l, pdgfab, ncapg, chfr, thbs4b, ccne1, ube2i2, anapc10, RNF8, vegfbb, ttk, tdrd9, cdc16, tex15, smc3, cdc45, fbxo5, mad2l1, mre11a, CDC37, fgf3, wee2, spast, KIF20A, top2b, haus6, seh1l, setd8a, apoa2, txnl4a, dscc1, ect2, spc24, pelo, cdc14b, arl8ba, h2afx, rcc2, ube2i, cdk1, mnd1, mns1, usp37, ccnb1, skiv2l2, ska1, pms1, tipin, cdc20, ccnd2a, ccnb3, cenpn, gnb2l1, tp53, RBBP8, swap70b, ncapd2, fsd1, cdc27, gnl3, zw10, ercc6l, vegfab, nuf2, aurkb, top2a
mitotic nuclear division	62 of 8294 genes, 0.7%	88 of 22409 genes, 0.4%	5.60E-07	0.00%	0	mastl, smc3, mad2l1, fbxo5, ndc80, gnl2, wee2, snx33, haus6, cdca8, seh1l, cdc26, tpx2, mad1l1, setd8a, pafah1b1b, smc2, actr8, fam83d, txnl4a, dscc1, zwilch, pafah1b1a, ANAPC4, ncaph, bora, spc24, arl8ba, katna1, rcc2, wee1, rad21a, ube2i, usp37, mcmbp, nek8, ccnf, ncapd3, ska1, tipin, ska3, snx18a, TXNL4B, SETDB2, nusap1, cenpn, dctn2, cep63, RBBP8, klhl21, swap70b, ncapd2, fsd1, chfr, ncapg, gnl3, zw10, ube2i2, ercc6l, anapc10, nuf2, aurkb
translational initiation	46 of 8294 genes, 0.6%	60 of 22409 genes, 0.3%	1.30E-06	0.00%	0	ticrr, eif3i, EIF2A, eif4eb, eif3jb, eif3c, EIF1AD, eif3d, eif2b4, eif2s2, eif4ebp3, eif4a2, eif2ak1, eif3eb, EIF3F, eif6, eif5a, eif4ebp2, eif3m, eif3ha, denr, mtif2, eif3g, eif3s6ip, eif4a1a, eif5, eif1axb, eif3ba, eif4ea, eif3ea, EIF3K, eif2b1, eif2s3, gra, eif4bb, EIF1B, eif4e1c, trmt6, eif2ak3, eif2b3, eif4ebp1, eif4e2rs1, eif2b2, eif3s10, eif3ja, eif4ebp3l
cell cycle process	125 of 8294 genes, 1.5%	218 of 22409 genes, 1.0%	2.26E-06	0.00%	0	mastl, prc1b, fbxl15, prc1a, ndc80, gnl2, snx33, cep72, cdca8, cdc26, tpx2, mad1l1, pafah1b1b, smc2, fam83d, actr8, ercc4, zwilch, pafah1b1a, ANAPC4, ncaph, cdkn1ba, bora, hells, tp73, snap25a, katna1, nabp1a, cdkn1bb, wee1, rad21a, mcmbp, cdc25, smarcb1a, nek8, ccnf, zgc:86764, ncapd3, ccnd1, ska3, plk4, snx18a, TXNL4B, stil, trip13, SETDB2, nusap1, chek1, bre, kif23, dctn2, prkci, cep63, tbl3, sass6, klhl21, zgc:56231, si:dkey-283b15.3, dtl, esco2, ncapg, chfr, sesn2, vangl2, ube2i2, anapc10, ttk, ticrr, tex15, usp33, smc3, cdkn1a, foxm1, fbxo5, mad2l1, CDKN1C, fam175a, haus3, insm1a, sesn1, cenph, wee2, KIF20A, top2b, haus6, ints3, seh1l, ppp2r4, atm, setd8a, pold1, mdm2, txnl4a, dscc1, ect2, haus1, spc24, arl8ba, rcc2, ube2i, usp37, HAUS4, ska1, pms1, tipin, antxr2a, kdm8, haus5, ccnb3, cenpn, tmpoa, tp53, RBBP8, rad1, swap70b, ncapd2, fsd1, wnt11, gnl3, zw10, ercc6l, nuf2, rhoab, aurkb, top2a
ribonucleo-protein complex assembly	32 of 8294 genes, 0.4%	39 of 22409 genes, 0.2%	3.13E-05	0.00%	0	ticrr, eif3i, eif3jb, icln, dicer1, eif3c, eif3d, eif3eb, prpf8, rpsa, EIF3F, eif6, eif3ha, eif3m, eif3g, eif3s6ip, shq1, snrpc, eif3ba, prmt7, sart3, eif3ea, patl1, coil, EIF3K, pwp2h, eif3s10, tarbp2, eif3ja, nip7, prpf31, RPS17
nuclear division	77 of 8294 genes, 0.9%	125 of 22409 genes, 0.6%	6.02E-05	0.00%	0	mastl, ndc80, gnl2, snx33, cdca8, cdc26, tpx2, mad1l1, pafah1b1b, smc2, fam83d, actr8, ercc4, zwilch, pafah1b1a, ANAPC4, ncaph, bora, hells, katna1, wee1, rad21a, mcmbp, nek8, ccnf, ncapd3, ska3, snx18a, TXNL4B, trip13, SETDB2, nusap1, dctn2, cep63, sass6, klhl21, ncapg, chfr, ube2i2, anapc10, ttk, tdrd9, tex15, smc3, fbxo5, mad2l1, mre11a, wee2, top2b, haus6, seh1l, setd8a, apoa2, txnl4a, dscc1, spc24, arl8ba, h2afx, rcc2, ube2i, mns1, mnd1, usp37, pms1, ska1, tipin, cenpn, RBBP8, ncapd2, swap70b, fsd1, gnl3, zw10, ercc6l, nuf2, aurkb, top2a

cell cycle	206 of 8294 genes, 2.5%	399 of 22409 genes, 1.8%	4.28E-06	0.00%	0	mastl, prc1b, ndc80, cep72, cdc26, mad1l1, rps15a, pafah1b1b, fam83d, ercc4, lin54, ccne2, cdkn1ba, snap25a, katna1, hm:zeh0351, nabp1a, cdkn1bb, wee1, CCNA2, maea, mcmbp, rbb4, smarcb1a, ccdc124, zgc:86764, ska3, plk4, snx18a, TXNL4B, lin37, trip13, sept8a, nusap1, erh, chek1, prkci, cep63, tbl3, esco2, chfr, sesn2, gadd45g, ccne1, ube2i2, anapc10, gadd45ab, rpl23a, ttk, sept5a, tdrd9, usp22, smc3, foxm1, cdc45, fbxo5, mre11a, insm1a, fam58a, CDC37, cenph, rb1, mcm5, ints3, seh1l, prkcda, ppp2r4, gadd45ba, gadd45gip1, ect2, arl8ba, rcc2, mnd1, usp37, tp53bp2, rbl1, antxr2a, haus5, ccnd2a, sept5b, cenpn, rpl36a, swap70b, chaf1b, wnt11, cdc27, zw10, rpl36, nuf2, zgc:63587, rhoab, bap1, top2a, plk1, ccnb2, fbxl15, rnf2, gnl2, prc1a, cdc73, snx33, rps29, cdca8, tpx2, smc2, rps8a, actr8, cdc40, zwilch, rpl7, ANAPC4, pafah1b1a, cdc42l, bora, ncaph, hells, tp73, rad21a, cdc25, cks2, ccnf, nek8, ncapd3, ccnd1, stil, SETDB2, rps7, aspm, bre, ssrp1a, src, kif23, dctn2, cdc42, chaf1a, mybl2, sass6, klhl21, zgc:56231, si:dkey-283b15.3, cdc5l, tfdp1a, cdk5rap3, dtl, ncapg, smarcal1, vangl2, atoh7, ccnk, ticrr, cdc16, tex15, usp33, cdkn1a, mad2l1, fam175a, CDKN1C, haus3, gadd45bb, sesn1, wee2, spast, KIF20A, top2b, haus6, atm, mcts1, setd8a, bop1, pes, pold1, mdm2, txnl4a, dscc1, haus1, spc24, pelo, cdc14b, h2afx, ube2i, cdk1, mns1, bccip, tceb2, ccnb1, HAUS4, ska1, pms1, tipin, rpl13, rpl35, kdm8, cdc20, ccnb3, rps18, tmpoa, tp53, RBBP8, vcp, rad1, ncapd2, fsd1, gnl3, ercc6l, cks1b, aurkb
DNA replication	61 of 8294 genes, 0.7%	94 of 22409 genes, 0.4%	0.00011	0.00%	0	ticrr, pold3, gins2, tert, RFC4, ssbp1, mcm7, cdc45, rrm1, dna2, pinx1, mcm10, top2b, orc3, mcm5, fen1, ORC4, GINS4, RRM2, pold1, INO80E, rpa1, dscc1, rfc2, prim1, pola2, rmi1, lrwd1, pole2, orc5, helb, mcmbp, rfc3, rbb4, TK1, rbb4l, gins1, LIG3, MCM3, mcm4, pole, pcna, tipin, zgc:110727, pola1, chaf1a, igfbp2b, pold2, lonp1, gmnn, WRNIP1, esco2, dtl, orc1, mcm2, mcm6, top1l, polb, wrn, orc6, top2a
rRNA metabolic process	36 of 8294 genes, 0.4%	48 of 22409 genes, 0.2%	0.00028	0.00%	0	dkc1, exosc6, HEATR1, c1d, nop10, pes, bop1, rps8a, tsr2, rpsa, dcaf13, wdr36, wdr55, nol11, krr1, rrnad1, wdr12, gar1, utp15, ybey, nhp2, fbl, nop2, RPS6, mphosph10, rps7, WBP11, rps21, rnasen, dimt1l, tbl3, ftsj, rps24, bms1l, rrp36, rnaset2
organelle fission	81 of 8294 genes, 1.0%	137 of 22409 genes, 0.6%	0.00034	0.00%	0	mastl, ndc80, march5l, gnl2, snx33, cdca8, cdc26, tpx2, mad1l1, pafah1b1b, smc2, fam83d, actr8, ercc4, zwilch, pafah1b1a, ANAPC4, ncaph, bora, hells, katna1, wee1, rad21a, mcmbp, nek8, ccnf, ncapd3, ska3, snx18a, TXNL4B, trip13, SETDB2, nusap1, apg3l, dctn2, PEX11G, cep63, sass6, klhl21, ncapg, chfr, ube2i2, anapc10, ttk, tdrd9, tex15, smc3, fbxo5, mad2l1, MTFP1, mre11a, wee2, top2b, haus6, seh1l, setd8a, apoa2, txnl4a, dscc1, spc24, arl8ba, h2afx, rcc2, ube2i, mnd1, mns1, usp37, pms1, ska1, tipin, cenpn, RBBP8, ncapd2, swap70b, fsd1, gnl3, zw10, ercc6l, nuf2, aurkb, top2a
rRNA processing	34 of 8294 genes, 0.4%	46 of 22409 genes, 0.2%	0.00117	0.00%	0	dkc1, exosc6, HEATR1, c1d, nop10, pes, bop1, rps8a, tsr2, rpsa, dcaf13, wdr36, wdr55, nol11, krr1, rrnad1, wdr12, gar1, utp15, ybey, nhp2, fbl, nop2, RPS6, mphosph10, rps7, WBP11, rps21, dimt1l, tbl3, ftsj, rps24, bms1l, rrp36
cellular response to DNA damage stimulus	114 of 8294 genes, 1.4%	213 of 22409 genes, 1.0%	0.00188	0.00%	0	tdp2b, mastl, rad51, phlda3, dna2, inip, fanci, actr5, parpbp, rad18, mcl1b, smc2, actr8, rad52, gtf2h2, stra13, INO80E, ercc4, ruvbl1, xrcc1, sfr1, dclre1b, nthl1, bcl2l10, rad51ap1, prp19, neil3, BCL2, ercc5, ube2t, tp73, nsmce4a, brca2, nabp1a, mgmt, smarcb1a, scrt2, LIG3, prmt6, ccnd1, xrcc5, trip13, pmaip1, chek1, bre, chchd6a, cry4, chaf1a, ube2nb, gtf2h1, cep63, chd1l, apex1, dtl, pif1, gtf2h4, polb, PIDD, ruvbl2, RNF8, mcl1a, zgc:112496, xrcc4, ube2na, smc1al, ticrr, tex15, smc4, zgc:66475, fam175a, mre11a, zgc:110224, baxa, dmap1, fen1, ints3, gtf2h3, atm, pold1, POLH, tdp1, ercc3, setx, h2afx, smc5, rad23b, baxb, TDG, bccip, smyd2a, KIF22, crip1, jmy, pcna, pms1, tipin, rad50, bcl2l1, pola1, tp53, RBBP8, ogg1, vcp, rad1, nudt1, aptx, ccdc94, poli, mutyh, plrg1, wrn, rad23aa, pms2, mus81

ribonucleo-protein complex subunit [illegible]	33 of 8294 genes, 0.4%	45 of 22409 genes, 0.2%	0.00237	0.00%	0	ticrr, eif3i, eif3jb, icln, dicer1, eif3c, eif3d, eif3eb, prpf8, rpsa, EIF3F, eif6, eif3ha, eif3m, eif3g, eif3s6ip, shq1, snrpc, eif3ba, prmt7, sart3, eif3ea, patl1, coil, EIF3K, dctn2, pwp2h, eif3s10, tarbp2, eif3ja, nip7, prpf31, RPS17
DNA metabolic process	181 of 8294 genes, 2.2%	367 of 22409 genes, 1.6%	0.0025	0.00%	0	zgc:110216, tdp2b, zgc:194285, RFC4, ssbp1, mcm7, rrm1, dna2, inip, fanci, zgc:77816, rad18, stra13, ercc4, rpa1, sfr1, dclre1b, neil3, ercc5, nsmce4a, brca2, nabp1a, dnmt4, mgmt, mcmbp, rbb4, smarcb1a, rbb4l, pole, xrcc5, trip13, zgc:110727, ppfibp2b, cenpp, ube2nb, gtf2h1, gmnn, apex1, esco2, pif1, polb, dnmt3b, h1fx, xrcc4, smc1al, gins2, tdrd9, tert, terfa, dnase2, cdc45, zgc:66475, pinx1, mre11a, zgc:110224, mcm5, fen1, ints3, ORC4, setb, tdp1, rfc2, prim1, xrcc3, pole2, smc5, helb, TDG, mnd1, TK1, MCM3, rad50, gng5, pola1, phc2b, nudt1, h2afy, aptx, poli, mutyh, top1l, dnase1l3, rad23aa, hist2h2l, top2a, dnmt1, rad51, actr5, mcm10, TOP1MT, DDX11, parpbp, GINS4, RRM2, smc2, actr8, gtf2h2, rad52, INO80E, top3a, ruvbl1, h1f0, xrcc1, ncaph, nthl1, rmi1, lrwd1, rad51ap1, ube2t, orc5, histh1l, ncapd3, gins1, LIG3, nap1l4a, prmt6, bre, cry4, rad51d, chaf1a, chd1l, igfbp2b, WRNIP1, dtl, ncapg, orc1, smarcal1, gtf2h4, mcm2, mcm6, ruvbl2, RNF8, orc6, zgc:112496, ticrr, pold3, tex15, smc4, cebpb, fam175a, top2b, dmap1, orc3, gtf2h3, atm, pold1, hp1bp3, POLH, ercc3, dscc1, setx, pola2, zgc:171759, H2AFY2, h2afx, rad23b, bccip, rfc3, mcm4, KIF22, ncaph2, jmy, pcna, pms1, tipin, prmt7, dffb, ogg1, pold2, pot1, RBBP8, vcp, rad1, lonp1, ncapd2, nap1l1, obfc1, seta, wrn, mus81, pms2
response to stress	299 of 8294 genes, 3.6%	651 of 22409 genes, 2.9%	0.0043	0.00%	0	brd4, wdr45l, mastl, phlda3, dna2, rac2, fanci, cxcl12a, adra2a, msxb, tnfa, smarca4, dclre1b, bcl2l10, neil3, gpx7, ercc5, mgmt, ppiab, oxsr1a, ifnphi1, ube2nb, plcg1, apex1, pgk1, pdgfab, hamp2, TRAP1, gadd45ab, smc1al, lgals2a, dnaja2l, zgc:66475, defbl1, dusp22b, junbb, aldh1a2, si:dkey-260c8.4, tgif1, ints3, fen1, tdp1, pak2a, gadd45ba, igfbp1a, vdac3, sod1, smc5, ascl1a, smyd2a, gclc, TMEM173, skiv2l2, rad50, anxa6, pola1, WIPI1, cxcr4b, bambia, MAP3K1, ldha, rad51, pdgfaa, nfe2l2a, actr5, si:dkey-269d20.3, parpbp, il1b, actr8, rad52, gtf2h2, hsp70.3, xrcc1, igf2b, nthl1, si:dkey-163m14.2, rad51ap1, ube2t, tp73, sly1, scrt2, bre, apg3l, chchd6a, HSPB11, cry4, chd1l, fzd2, sgk1, cd40, pak4, tlr5b, pycard, ube2na, NOD1, lyn, tex15, sox11b, stat3, fam175a, traf3, il22, atm, POLH, acvr1b, ercc3, dixdc1a, cirbp, baxb, hspb1, saal1, bcl2l1, f3b, gpx4b, hspd1, tp53, prph, rad1, npc1, tlr21, naprt1, ccdc94, f3a, csf1ra, hsp90aa1.1, wrn, SCUBE3, pms2, tdp2b, ddit3, hsp70.1, hspe1, inip, tollip, ptgs2b, rad18, mcl1b, tlr3, ercc4, stra13, CD59, sfr1, sdcbp2, BCL2, prp19, hspb8, lipf, brca2, csrp1a, nsmce4a, nabp1a, si:dkey-81j8.6, park7, smarcb1a, mmp9, hsp90b1, saa, tnikb, f7i, xrcc5, trip13, chek1, apln, cep63, gtf2h1, atp6v1aa, pif1, gpx4a, thbs4b, acvrl1, dnaja2, gadd45g, polb, mpx, PIDD, adra2db, gpx3, mmp14a, mcl1a, xrcc4, ttk, pak1, foxm1, insm1a, lepb, mre11a, zgc:110224, myd88, nod2, six3b, atf6, hspb9, neurog1, si:ch211-253b1.4, cat, hspa9, TDG, dvl2, CCS, erbb2, crip1, hbaa1, dcbld2, il17a/f3, cart4, strada, tnc, tnfaip8l2a, nudt1, aptx, mutyh, plrg1, poli, alas2, rad23aa, anxa1a, fgf20a, hspa8, dpysl2b, mb, il4r, ncf1, aida, smc2, smo, INO80E, duox, ruvbl1, vdac2, ctsba, itgb3b, nadl1.1, TNFRSF1A, hbegfa, LIG3, ccnd1, bbc3, prmt6, GPX8, f2r, pmaip1, hspb7, chaf1a, mapk8ip3, dtl, loxl2b, gtf2h4, axin1, RNF8, rsad2, ruvbl2, zgc:112496, ticrr, tfpia, taok3a, cct4, mavs, cnp, smc4, wnt2ba, gadd45bb, baxa, alkbh5, lta4h, dmap1, sdc4, hspa4b, gtf2h3, fam53b, hsp90ab1, pold1, apaf1, setd6, hspa4a, setx, gap43, pak2b, h2afx, dkk1a, rad23b, bccip, wdr45, KIF22, notch2, jmy, pcna, pms1, tipin, hmgb1a, ryr1b, cdh2, ahsa1l, ogg1, RBBP8, vcp, lonp1, nupr1, hspa5, mus81

cellular component organization or biogenesis	603 of 8294 genes, 7.3%	1400 of 22409 genes, 6.2%	0.00267	0.00%	0	tmem231, brd4, alcamb, dna2, smarca5, ndc80, fzd6, slc35b2, asna1, cnn3b, mad1l1, etf1, gtpbp4, tubb4b, znf703, dclre1b, nrp1a, nol11, si:ch211-114n24.6, wee1, epb41b, cnn2, wisp3, ska3, snx18a, appb, TXNL4B, crb2b, whsc1, mdn1, stmn2a, ube2nb, prkci, hmp19, kctd9, spna2, grna, chfr, tnnt2c, carm1, cyfip2, MTFP1, fbxo5, eif3jb, ptk2.2, eif3c, rb1, seh1l, tnfaip1, stmn2b, parp3, si:ch73-334d15.4, ect2, rpsa, tagln2, pmm2, MTRF1, TMEM17, rcc2, psmg1, mnd1, smyd2a, fbl, elp3, rpl7a, WBP11, nlgn3b, haus5, anxa6, ctnnb1, phc2b, swap70b, jagn1a, rab13, her4.2, kctd3, bap1, fmn1, kctd15a, smn1, tuba2, hira, rnf2, march5l, gnl2, ezh2, hdac8, hdac1, nup107, ANAPC4, actr2b, bora, rilpl1, hells, lrrc4bb, eif3ha, dtnbp1a, rrs1, utp15, ncapd3, nap1l4a, eif5a2, stil, SETDB2, patl1, apg3l, coil, chd1l, klhl21, ncapg, kirrelb, smarcal1, cap1, kctd5, eny2, b9d1, agrn, prnprs3, mad2l1, cdc42se1, fam175a, icln, wee2, spast, HEATR1, itgb4, arpc1b, apoa2, ehmt2, dixdc1a, dscc1, slc39a6, mns1, krr1, eif3g, nhp2, ska1, kdm8, plxnb1a, foxd3, tp53, nhp2l1b, isl2a, gnl3, bms1l, hsp90aa1.1, foxd1, P4HTM, map7d1b, eif3i, cenpi, prc1b, kat2a, igfbp5b, ehmt1a, msrb2, gnl3l, wipf1, timm10, ercc4, eif3eb, bfsp2, notch1b, capza1b, dyx1c1, rbb4, smarcb1a, arpc5la, rsl24d1, rbb4l, daam1b, ift46, vegfaa, xrcc5, tbl3, bmi1b, fzd8a, slc33a1, esco2, cfl1l, srp72, dsc2l, tert, arpc5a, adcy8, psmd11a, orai1b, her4.4, cbfb, flncb, setb, alcama, nrp2b, sec13, aebp2, sprn2, ist1, shq1, gar1, erbb2, nop2, poc1b, cenpn, tnc, TEKT4, pfn1, h2afy, tubg1, tuba8l3, cmlc1, tarbp2, eif3ja, nuf2, srgap2a, map7d2a, RPS17, ctgfa, dnmt1, zgc:65894, ptprfb, fbln1, dpysl2b, p2rx4a, slc3a2a, igfbp7, gpc4, snx33, gli2a, map1aa, srp19, smc2, h1f0, map7d2b, tsr2, mib, tube1, dzip1, EIF3F, cnn3a, rad21a, shroom4, wdr12, SSR3, si:ch73-362m14.4, foxc1b, rps7, foxg1b, pwp2h, dctn2, notch3, chaf1a, tpm4a, igfbp2b, sass6, loxl2b, tyrp1b, axin1, ruvbl2, RNF8, taf1b, cnp, aldoaa, haus3, zgc:109889, SHROOM3, top2b, dicer1, ush1c, robo1, haus6, tuba8l, setd8a, bop1, gna13a, dld, H2AFY2, haus1, spc24, ube2i, wdr45, rab5c, dnajc2, nrp2a, tipin, psmg2, prmt7, kdm1a, asf1bb, coro7, rps18, cdh2, rtf1, RBBP8, lonp1, fzd8b, nap1l1, obfc1, eed, ercc6l, seta, aurkb, wdr45l, mastl, slc3a2b, kctd6a, LAMA1, nrp1b, p2rx3a, cxcl12a, cep72, igfbp3, cdc26, hat1, cav1, fam83d, buc, sdad1, ykt6, wdr55, katna1, zgc:112335, acvr1l, mcmbp, si:ch73-199e17.1, arpc1a, efnb2a, nusap1, tln1, olfm1a, cenpp, zgc:162967, noc4l, sec24d, ube2i2, h1fx, kctd12.2, nip7, ESM1, smc1al, cetn4, dkc1, tdrd9, usp22, smc3, enpp2, nkx2.2a, cenph, vps11, atp6v1e1b, ppp2r4, cep57l1, MEAF6, pak2a, igfbp1a, zgc:158689, eif5a, eif6, nup93, strumpellin,
cellular response to stress	143 of 8294 genes, 1.7%	280 of 22409 genes, 1.2%	0.00297	0.00%	0	wdr45l, tdp2b, mastl, ddit3, phlda3, dna2, inip, fanci, rad18, mcl1b, stra13, ercc4, sfr1, dclre1b, bcl2l10, prp19, neil3, BCL2, ercc5, nsmce4a, csrp1a, brca2, nabp1a, mgmt, park7, smarcb1a, xrcc5, trip13, chek1, ube2nb, gtf2h1, cep63, apex1, pif1, polb, PIDD, mcl1a, xrcc4, smc1al, foxm1, zgc:66475, mre11a, zgc:110224, dusp22b, fen1, ints3, atf6, tdp1, neurog1, sod1, smc5, cat, TDG, smyd2a, dvl2, CCS, crip1, rad50, pola1, cart4, tnc, WIPI1, nudt1, aptx, poli, plrg1, mutyh, rad23aa, dpysl2b, rad51, actr5, parpbp, aida, actr8, smc2, gtf2h2, rad52, smo, INO80E, ruvbl1, xrcc1, nthl1, rad51ap1, ube2t, tp73, nadl1.1, scrt2, LIG3, ccnd1, bbc3, prmt6, pmaip1, bre, apg3l, chchd6a, cry4, chaf1a, mapk8ip3, chd1l, dtl, gtf2h4, axin1, RNF8, ruvbl2, zgc:112496, ube2na, ticrr, cnp, tex15, smc4, fam175a, baxa, dmap1, gtf2h3, atm, pold1, POLH, ercc3, dixdc1a, setx, h2afx, rad23b, baxb, bccip, wdr45, KIF22, jmy, pcna, pms1, tipin, bcl2l1, ryr1b, ogg1, RBBP8, tp53, vcp, rad1, lonp1, nupr1, ccdc94, wrn, mus81, pms2
formation of translation preinitiation complex	16 of 8294 genes, 0.2%	17 of 22409 genes, 0.1%	0.00414	0.00%	0	ticrr, eif3i, eif3s6ip, eif3g, eif3jb, eif3ba, eif3c, eif3ea, EIF3K, eif3d, eif3eb, eif3s10, eif3ja, EIF3F, eif3ha, eif3m
