# Supplementary material for: Vascular Endothelial Growth Factor A and Leptin Expression Associated with Ectopic Proliferation and Retinal Dysplasia in Zebrafish Optic Pathway Tumors
Source: Zebrafish. 2017 Aug 1;14(4):343–56. doi: 10.1089/zeb.2016.1366 (PMC5549800; doi:10.1089/zeb.2016.1366)
Supplement: Supplemental data [file Supp_Data.zip › Supp_Table5.pdf]

Supplementary Table S5. Zebrafish *Tg(flk1:RFP)is18/+* Dysplastic retina and retinal Tumor 3 fold DGE and human homologs

Human Ensembl Gene ID	Gene name	Zebrafish Ensembl Gene ID	Wildtype to Dysplastic retina log(2) fold change	Wildtype to Dysplastic retina *p*value	Wildtype to Tumor log(2) fold change	Wildtype to Tumor *p*value
ENSG00000160202	cryaa	ENSDARG00000053502	12.4019	0.0000	6.7682	0.0000
ENSG00000174697	lepb	ENSDARG00000045548	8.9940	0.0000	8.6567	0.0000
ENSG00000130300	si:dkey-208k22.3	ENSDARG00000076972	8.3259	0.0000	8.3949	0.0000
ENSG00000163273	zgc:195023	ENSDARG00000052948	7.8887	0.0000	11.9936	0.0000
ENSG00000178750	STX19	ENSDARG00000074029	6.9896	0.0000	6.8455	0.0000
ENSG00000165175	mid1ip1a	ENSDARG00000041051	6.8869	0.0000	5.9857	0.0000
ENSG00000127318	il22	ENSDARG00000045673	6.7609	0.0000	2.0000	0.0000
ENSG00000174175	sele	ENSDARG00000009123	5.8754	0.0000	6.0354	0.0000
ENSG00000109846	cryaba	ENSDARG00000042621	5.8207	0.0000	-0.9386	0.0895
ENSG00000152049	kcne4	ENSDARG00000068257	5.7411	0.0000	2.5236	0.0000
ENSG00000181649	phlda2	ENSDARG00000042874	5.3306	0.0000	5.7335	0.0000
ENSG00000167996	BX511021.2	ENSDARG00000073936	5.3272	0.0000	5.2387	0.0000
ENSG00000137965	CABZ01073795.1	ENSDARG00000010729	5.3054	0.0000	8.4244	0.0000
ENSG00000148926	ADM	ENSDARG00000069027	5.1820	0.0000	4.4333	0.0000
ENSG00000172216	cebpb	ENSDARG00000042725	5.1508	0.0000	4.8147	0.0000
ENSG00000108821	col1a1a	ENSDARG00000012405	5.1359	0.0000	6.4665	0.0000
ENSG00000165449	slc16a9a	ENSDARG00000013926	5.0570	0.0000	4.3601	0.0000
ENSG00000005882	pdk2b	ENSDARG00000059054	5.0317	0.0000	2.1480	0.0000
ENSG00000244752	crybb2	ENSDARG00000053512	5.0210	0.0000	5.7764	0.0000
ENSG00000182718	anxa2a	ENSDARG00000003216	5.0120	0.0000	7.8593	0.0000
ENSG00000112936	C7	ENSDARG00000057121	4.8495	0.0000	4.6019	0.0000
ENSG00000128165	adm2a	ENSDARG00000045708	4.7704	0.0000	3.6818	0.0000
ENSG00000187608	isg15	ENSDARG00000086374	4.6274	0.0000	8.6987	0.0000
ENSG00000113070	hbegfa	ENSDARG00000075121	4.6085	0.0000	5.6076	0.0000
ENSG00000135046	anxa1a	ENSDARG00000026726	4.5161	0.0000	5.5992	0.0000
ENSG00000129521	egln3	ENSDARG00000032553	4.4835	0.0000	4.5047	0.0000
ENSG00000131781	si:dkey-239i20.4	ENSDARG00000043514	4.4801	0.0000	3.6387	0.0000
ENSG00000173372	c1qa	ENSDARG00000044613	4.4352	0.0000	5.3692	0.0000
ENSG00000148450	msrb2	ENSDARG00000018459	4.4295	0.0000	2.4121	0.0000
ENSG00000198125	mb	ENSDARG00000031952	4.4214	0.0000	3.6951	0.0000
ENSG00000113594	lifrb	ENSDARG00000039863	4.3834	0.0000	5.3923	0.0000
ENSG00000116711	pla2g4ab	ENSDARG00000017141	4.3681	0.0000	3.0834	0.0000
ENSG00000105643	arrdc2	ENSDARG00000020761	4.3473	0.0000	3.1688	0.0000
ENSG00000137745	mmp13a	ENSDARG00000012395	4.3189	0.0000	7.0323	0.0000
ENSG00000162139	neu3.5	ENSDARG00000096368	4.2574	0.0000	3.5078	0.0000
ENSG00000039537	c6	ENSDARG00000057113	4.2555	0.0000	4.4368	0.0000
ENSG00000159189	c1qc	ENSDARG00000095627	4.2464	0.0000	5.3597	0.0000
ENSG00000130201	exoc3l2a	ENSDARG00000008414	4.1674	0.0000	4.5850	0.0000
ENSG00000197766	cfd	ENSDARG00000039579	4.1290	0.0000	3.6561	0.0000
ENSG00000100450	si:dkey-78l4.6	ENSDARG00000071389	4.0821	0.0000	-0.6077	0.1690
ENSG00000011465	dcn	ENSDARG00000012066	4.0721	0.0000	5.6845	0.0000
ENSG00000240764	pcdhgc5	ENSDARG00000077389	4.0414	0.0000	-0.0942	0.5860
ENSG00000173369	c1qb	ENSDARG00000044612	4.0026	0.0000	4.8269	0.0000
ENSG00000181885	cldn7a	ENSDARG00000036376	3.9765	0.0000	3.8156	0.0000
ENSG00000079150	fkbp7	ENSDARG00000010962	3.9658	0.0000	4.2046	0.0000
ENSG00000180537	CU019646.2	ENSDARG00000091234	3.9533	0.0000	5.7533	0.0000
ENSG00000186352	ankrd37	ENSDARG00000056376	3.9507	0.0000	1.7647	0.0000
ENSG00000073756	ptgs2b	ENSDARG00000010276	3.8935	0.0000	1.8398	0.0000
ENSG00000137699	ftr59	ENSDARG00000095864	3.8861	0.0000	3.7162	0.0000
ENSG00000115641	fhl2a	ENSDARG00000042018	3.8767	0.0000	0.6966	0.0505
ENSG00000165471	hbl2	ENSDARG00000094681	3.8002	0.0000	2.0247	0.0000
ENSG00000250264	abcb3l1	ENSDARG00000036787	3.7949	0.0000	5.3512	0.0000

ENSG00000145826	LECT2	ENSDARG00000090889	3.7680	0.0000	4.7212	0.0000
ENSG00000180815	map3k15	ENSDARG00000013310	3.7492	0.0000	3.2020	0.0000
ENSG00000130529	trpm4a	ENSDARG00000059993	3.7304	0.0000	2.6000	0.0000
ENSG00000221869	cebpd	ENSDARG00000087303	3.6709	0.0000	0.8432	0.0000
ENSG00000111911	si:dkey-25e12.3	ENSDARG00000055365	3.6674	0.0000	6.0521	0.0000
ENSG00000184557	socs3a	ENSDARG00000025428	3.6389	0.0000	3.4391	0.0000
ENSG00000213085	cfap45	ENSDARG00000068103	3.6110	0.0000	3.5118	0.0000
ENSG00000204010	ifit14	ENSDARG00000071012	3.5546	0.0000	3.9542	0.0000
ENSG00000111057	krt18	ENSDARG00000018404	3.5540	0.0000	3.5100	0.0000
ENSG00000136997	mych	ENSDARG00000077473	3.5523	0.0000	4.9267	0.0000
ENSG00000189143	zgc:112437	ENSDARG00000009215	3.5502	0.0000	4.7308	0.0000
ENSG00000049249	tnfrsf9a	ENSDARG00000060498	3.5481	0.0000	3.1198	0.0000
ENSG00000204010	ifit16	ENSDARG00000056976	3.5380	0.0000	6.8353	0.0000
ENSG00000116176	si:dkey-16l2.17	ENSDARG00000027196	3.4819	0.0000	3.7119	0.0000
ENSG00000118523	ctgfa	ENSDARG00000042934	3.4805	0.0000	1.0314	0.0000
ENSG00000112715	vegfab	ENSDARG00000034700	3.4474	0.0000	3.0845	0.0000
ENSG00000164111	anxa5b	ENSDARG00000016470	3.4236	0.0000	5.0701	0.0000
ENSG00000059804	slc2a3b	ENSDARG00000037861	3.4198	0.0000	3.4646	0.0000
ENSG00000130203	apoeb	ENSDARG00000040295	3.4167	0.0000	3.7814	0.0000
ENSG00000119632	si:dkey-188i13.7	ENSDARG00000078389	3.3794	0.0000	6.8408	0.0000
ENSG00000179820	MYADM	ENSDARG00000086815	3.3716	0.0000	4.7629	0.0000
ENSG00000123496	il13ra2	ENSDARG00000039436	3.3699	0.0000	-0.2671	0.3347
ENSG00000113739	stc2a	ENSDARG00000056680	3.3528	0.0000	2.8751	0.0000
ENSG00000130598	tnni2b.1	ENSDARG00000035958	3.3505	0.0000	3.3785	0.0000
ENSG00000116774	olfml3b	ENSDARG00000062171	3.3494	0.0000	2.6758	0.0000
ENSG00000158104	hpdb	ENSDARG00000044935	3.3350	0.0000	1.1255	0.0410
ENSG00000164692	col1a2	ENSDARG00000020007	3.3310	0.0000	4.1966	0.0000
ENSG00000155962	clic2	ENSDARG00000010625	3.3160	0.0000	3.7335	0.0000
ENSG00000108821	col1a1b	ENSDARG00000035809	3.3133	0.0000	4.1007	0.0000
ENSG00000100906	nfkbiaa	ENSDARG00000005481	3.3047	0.0000	4.1761	0.0000
ENSG00000019549	snai2	ENSDARG00000040046	3.2993	0.0000	1.4443	0.0000
ENSG00000185338	socs1a	ENSDARG00000038095	3.2978	0.0000	1.6573	0.0000
ENSG00000149591	tagln2	ENSDARG00000033466	3.2930	0.0000	3.9008	0.0000
ENSG00000166959	ms4a17a.1	ENSDARG00000043798	3.2840	0.0000	3.4100	0.0000
ENSG00000204264	psmb8a	ENSDARG00000001303	3.2719	0.0000	5.0573	0.0000
ENSG00000165949	zgc:152791	ENSDARG00000039243	3.2718	0.0000	5.9722	0.0000
ENSG00000100292	hmox1a	ENSDARG00000027529	3.2643	0.0000	5.1414	0.0000
ENSG00000122884	p4ha1b	ENSDARG00000071082	3.2599	0.0000	2.6960	0.0000
ENSG00000115902	slc1a4	ENSDARG00000000551	3.2519	0.0000	2.8152	0.0000
ENSG00000106608	BX005336.3	ENSDARG00000078731	3.2327	0.0000	5.8003	0.0000
ENSG00000242366	ugt1ab	ENSDARG00000006220	3.2194	0.0000	2.3388	0.0000
ENSG00000139549	dhh	ENSDARG00000037062	3.1758	0.0000	1.7208	0.0000
ENSG00000177469	ptrfb	ENSDARG00000059362	3.1697	0.0000	2.0290	0.0000
ENSG00000106785	si:dkey-286j15.3	ENSDARG00000071036	3.1595	0.0000	3.5654	0.0000
ENSG00000265107	cx41.8	ENSDARG00000069450	3.1588	0.0000	2.5514	0.0000
ENSG00000173585	ccr9a	ENSDARG00000055186	3.1402	0.0000	3.2752	0.0000
ENSG00000196154	icn	ENSDARG00000009978	3.1379	0.0000	3.6133	0.0000
ENSG00000148926	adma	ENSDARG00000015263	3.1363	0.0000	3.8236	0.0000
ENSG00000092010	psme1	ENSDARG00000002165	3.1310	0.0000	4.6646	0.0000
ENSG00000121797	CCRL2	ENSDARG00000086314	3.1293	0.0000	2.7334	0.0000
ENSG00000132688	nes	ENSDARG00000088805	3.1282	0.0000	7.3408	0.0000
ENSG00000128591	flnca	ENSDARG00000018566	3.1212	0.0000	5.6387	0.0000
ENSG00000215018	col28a1	ENSDARG00000077084	3.1124	0.0000	1.8817	0.0000
ENSG00000087074	ppp1r15a	ENSDARG00000069135	3.1083	0.0000	2.6420	0.0000
ENSG00000119147	C9H2orf40	ENSDARG00000056087	3.1078	0.0000	2.6930	0.0000
ENSG00000196954	caspb	ENSDARG00000052039	3.1015	0.0000	4.3226	0.0000
ENSG00000019582	cd74a	ENSDARG00000009087	3.1003	0.0000	3.9126	0.0000
ENSG00000167670	chaf1a	ENSDARG00000062152	3.0974	0.0000	3.5652	0.0000

ENSG00000169403	ptafr	ENSDARG00000042370	3.0952	0.0000	3.6801	0.0000
ENSG00000083444	plod1a	ENSDARG00000059746	3.0898	0.0000	3.2168	0.0000
ENSG00000181104	f2r	ENSDARG00000060012	3.0784	0.0000	4.3687	0.0000
ENSG00000124449	irgf1	ENSDARG00000070774	3.0780	0.0000	4.6245	0.0000
ENSG00000054598	foxc1b	ENSDARG00000055398	3.0652	0.0000	3.4988	0.0000
ENSG00000152583	sparcl1	ENSDARG00000074989	3.0482	0.0000	0.0000	1.0000
ENSG00000178445	GLDC	ENSDARG00000035120	3.0442	0.0000	3.3023	0.0000
ENSG00000185215	tnfaip2b	ENSDARG00000058160	3.0402	0.0000	5.7932	0.0000
ENSG00000096696	dspa	ENSDARG00000022309	3.0376	0.0000	2.7491	0.0000
ENSG00000185674	lygl1	ENSDARG00000056874	3.0375	0.0000	4.8448	0.0000
ENSG00000166710	b2m	ENSDARG00000053136	3.0274	0.0000	4.6180	0.0000
ENSG00000121361	kcnj8	ENSDARG00000045589	3.0247	0.0000	1.9036	0.0000
ENSG00000144847	si:ch211-24o10.6	ENSDARG00000088251	3.0205	0.0000	6.3269	0.0000
ENSG00000175567	ucp2	ENSDARG00000043154	3.0179	0.0000	3.9286	0.0000
ENSG00000124216	snai1a	ENSDARG00000056995	3.0168	0.0000	2.8455	0.0000
ENSG00000162618	adgrl4	ENSDARG00000013653	3.0075	0.0000	2.4937	0.0000
ENSG00000179403	vwa1	ENSDARG00000075468	3.0041	0.0000	3.6878	0.0000
ENSG00000175592	fosl1a	ENSDARG00000015355	3.0000	0.0000	3.8686	0.0000
ENSG00000024422	ehd2b	ENSDARG00000040362	2.9938	0.0000	2.3896	0.0000
ENSG00000180772	agtr2	ENSDARG00000035552	2.9853	0.0000	1.5979	0.0000
ENSG00000211445	gpx3	ENSDARG00000043342	2.9793	0.0000	3.2439	0.0000
ENSG00000129048	ackr4a	ENSDARG00000078729	2.9696	0.0000	3.1155	0.0000
ENSG00000157227	mmp14a	ENSDARG00000002235	2.9641	0.0000	3.6758	0.0000
ENSG00000158615	ppp1r15b	ENSDARG00000068128	2.9641	0.0000	3.3456	0.0000
ENSG00000092531	snap23.2	ENSDARG00000055252	2.9608	0.0000	3.6189	0.0000
ENSG00000188910	cx35.4	ENSDARG00000042866	2.9500	0.0000	2.6386	0.0000
ENSG00000162772	atf3	ENSDARG00000007823	2.9462	0.0000	5.9796	0.0000
ENSG00000081138	CDH7	ENSDARG00000017649	2.9458	0.0000	1.5824	0.0000
ENSG00000131480	aoc2	ENSDARG00000014646	2.9440	0.0000	2.2111	0.0000
ENSG00000159176	csrp1a	ENSDARG00000006603	2.9296	0.0000	3.7232	0.0000
ENSG00000154096	thy1	ENSDARG00000035018	2.9132	0.0000	6.5027	0.0000
ENSG00000180340	fzd2	ENSDARG00000054438	2.9127	0.0000	3.4963	0.0000
ENSG00000223953	c1qtnf5	ENSDARG00000056134	2.9069	0.0000	3.4919	0.0000
ENSG00000277893	srd5a2a	ENSDARG00000043587	2.8998	0.0000	2.3906	0.0000
ENSG00000106785	zgc:171679	ENSDARG00000071024	2.8917	0.0000	1.4253	0.0000
ENSG00000155254	marveld1	ENSDARG00000011724	2.8904	0.0000	2.8224	0.0000
ENSG00000243056	eif4ebp3l	ENSDARG00000041607	2.8830	0.0000	1.5061	0.0000
ENSG00000112118	mcm3	ENSDARG00000024204	2.8786	0.0000	3.7433	0.0000
ENSG00000255587	rab44	ENSDARG00000028389	2.8601	0.0000	2.7244	0.0000
ENSG00000019169	marco	ENSDARG00000059294	2.8600	0.0000	5.2089	0.0000
ENSG00000196811	chrng	ENSDARG00000086647	2.8554	0.0000	2.4594	0.0000
ENSG00000177363	si:ch1073-303k11.2	ENSDARG00000088247	2.8497	0.0000	2.5781	0.0000
ENSG00000187498	col4a1	ENSDARG00000055009	2.8471	0.0000	2.9163	0.0000
ENSG00000158315	rhbdl2	ENSDARG00000069833	2.8458	0.0000	2.5454	0.0000
ENSG00000186529	cyp4f3	ENSDARG00000053530	2.8456	0.0000	2.4616	0.0000
ENSG00000078401	edn1	ENSDARG00000036912	2.8413	0.0000	-0.2313	0.4841
ENSG00000180549	fut7	ENSDARG00000044775	2.8289	0.0000	3.4935	0.0000
ENSG00000189233	NUGGC	ENSDARG00000091951	2.8289	0.0000	2.1444	0.0000
ENSG00000127074	rgs13	ENSDARG00000017653	2.8213	0.0000	3.2988	0.0000
ENSG00000216490	ifi30	ENSDARG00000056378	2.8209	0.0000	4.3884	0.0000
ENSG00000158428	catip	ENSDARG00000063009	2.8205	0.0000	2.9373	0.0000
ENSG00000121966	cxcr4b	ENSDARG00000041959	2.8196	0.0000	3.1612	0.0000
ENSG00000133392	myh11a	ENSDARG00000009782	2.8194	0.0000	2.0211	0.0000
ENSG00000151651	adam8a	ENSDARG00000001452	2.8017	0.0000	4.8890	0.0000
ENSG00000167703	slc43a2b	ENSDARG00000061120	2.7987	0.0000	0.5799	0.0000
ENSG00000163131	ctssb.1	ENSDARG00000074656	2.7867	0.0000	3.8048	0.0000
ENSG00000138119	MYOF	ENSDARG00000017128	2.7809	0.0000	2.2075	0.0000
ENSG00000174600	cmklr1	ENSDARG00000090890	2.7733	0.0000	3.2164	0.0000

ENSG00000164932	cthrc1a	ENSDARG00000087198	2.7620	0.0000	1.6323	0.0000
ENSG00000106333	pcolcea	ENSDARG00000056913	2.7595	0.0000	1.5268	0.0000
ENSG00000157601	mxe	ENSDARG00000014427	2.7590	0.0000	6.7300	0.0000
ENSG00000111057	zgc:77517	ENSDARG00000028618	2.7419	0.0000	4.2904	0.0000
ENSG00000124491	f13a1b	ENSDARG00000036893	2.7345	0.0000	2.6001	0.0000
ENSG00000135919	serpine2	ENSDARG00000029353	2.7293	0.0000	4.2261	0.0000
ENSG00000128567	podxl	ENSDARG00000031228	2.7286	0.0000	1.8027	0.0000
ENSG00000170421	krt4	ENSDARG00000017624	2.7116	0.0000	1.9518	0.0000
ENSG00000133574	CR933791.1	ENSDARG00000052876	2.7105	0.0000	2.6491	0.0000
ENSG00000099860	gadd45ba	ENSDARG00000027744	2.6942	0.0000	1.4280	0.0000
ENSG00000204103	mafbb	ENSDARG00000070542	2.6904	0.0000	3.3750	0.0000
ENSG00000132530	XAF1	ENSDARG00000068939	2.6898	0.0000	4.9994	0.0000
ENSG00000168884	tnip2	ENSDARG00000074501	2.6889	0.0000	3.8320	0.0000
ENSG00000168329	BX537113.1	ENSDARG00000079829	2.6888	0.0000	2.9865	0.0000
ENSG00000197879	MYO1C	ENSDARG00000020924	2.6884	0.0000	3.7809	0.0000
ENSG00000168209	ddit4	ENSDARG00000037618	2.6810	0.0000	2.6344	0.0000
ENSG00000188313	si:ch211-71m22.1	ENSDARG00000058638	2.6804	0.0000	3.2576	0.0000
ENSG00000244165	p2ry11	ENSDARG00000014929	2.6801	0.0000	3.2410	0.0000
ENSG00000125931	cited1	ENSDARG00000077769	2.6741	0.0000	2.5850	0.0000
ENSG00000179761	pipox	ENSDARG00000055591	2.6689	0.0000	3.7928	0.0000
ENSG00000125810	CD93	ENSDARG00000074434	2.6670	0.0000	2.1510	0.0000
ENSG00000010278	cd9	ENSDARG00000044990	2.6640	0.0000	5.1154	0.0000
ENSG00000100097	lgals2a	ENSDARG00000054942	2.6616	0.0000	5.2243	0.0000
ENSG00000022267	fhl1b	ENSDARG00000056653	2.6503	0.0000	5.1942	0.0000
ENSG00000198075	sult1st6	ENSDARG00000006811	2.6357	0.0000	0.5424	0.0004
ENSG00000181195	penkb	ENSDARG00000036045	2.6258	0.0000	4.6389	0.0000
ENSG00000036473	otc	ENSDARG00000062147	2.6199	0.0000	-0.3964	0.0011
ENSG00000143387	ctsk	ENSDARG00000040251	2.6104	0.0000	3.9660	0.0000
ENSG00000141526	slc16a3	ENSDARG00000045051	2.6019	0.0000	1.4103	0.0000
ENSG00000112212	tspo	ENSDARG00000026655	2.5908	0.0000	2.2217	0.0000
ENSG00000162881	hcar1-4	ENSDARG00000087084	2.5902	0.0000	2.9326	0.0000
ENSG00000164323	cfap97	ENSDARG00000056381	2.5863	0.0000	0.7394	0.0000
ENSG00000166961	ms4a17a.3	ENSDARG00000091970	2.5850	0.0000	2.9608	0.0000
ENSG00000196154	icn2	ENSDARG00000055514	2.5850	0.0000	2.6501	0.0000
ENSG00000100453	si:dkey-21e2.15	ENSDARG00000092788	2.5850	0.0000	2.0952	0.0000
ENSG00000139567	acvrl1	ENSDARG00000018179	2.5750	0.0000	2.0021	0.0000
ENSG00000102096	pim2	ENSDARG00000059001	2.5654	0.0000	2.6053	0.0000
ENSG00000117450	prdx1	ENSDARG00000058734	2.5606	0.0000	3.2474	0.0000
ENSG00000099875	mknk2b	ENSDARG00000015164	2.5559	0.0000	2.0214	0.0000
ENSG00000135502	slc26a10	ENSDARG00000078800	2.5555	0.0000	0.3132	0.0714
ENSG00000240065	psmb9a	ENSDARG00000000656	2.5546	0.0000	4.4948	0.0000
ENSG00000105355	si:rp71-61h23.4	ENSDARG00000013711	2.5523	0.0000	2.4881	0.0000
ENSG00000130429	arpc1b	ENSDARG00000027063	2.5503	0.0000	3.5038	0.0000
ENSG00000076706	mcamb	ENSDARG00000005368	2.5428	0.0000	2.7532	0.0000
ENSG00000168395	ing5b	ENSDARG00000068175	2.5346	0.0000	4.0546	0.0000
ENSG00000133466	c1qtnf6a	ENSDARG00000055175	2.5236	0.0000	3.2730	0.0000
ENSG00000102879	coro1a	ENSDARG00000054610	2.5208	0.0000	3.3669	0.0000
ENSG00000122877	egr2a	ENSDARG00000044098	2.5176	0.0000	0.8365	0.0000
ENSG00000232629	mhc2dab	ENSDARG00000079105	2.5140	0.0000	3.0025	0.0000
ENSG00000138061	cyp1b1	ENSDARG00000068934	2.5128	0.0000	1.6840	0.0000
ENSG00000100368	csf2rb	ENSDARG00000076586	2.5034	0.0000	3.3478	0.0000
ENSG00000107796	acta2	ENSDARG00000045180	2.5025	0.0000	1.5305	0.0000
ENSG00000117228	gbp2	ENSDARG00000038669	2.4986	0.0000	4.6046	0.0000
ENSG00000152253	spc25	ENSDARG00000078433	2.4919	0.0000	4.4363	0.0000
ENSG00000120279	myct1a	ENSDARG00000056125	2.4854	0.0000	2.4594	0.0000
ENSG00000160326	slc2a6	ENSDARG00000058731	2.4792	0.0000	4.3021	0.0000
ENSG00000151835	sacs	ENSDARG00000075668	2.4785	0.0000	6.4217	0.0000
ENSG00000136108	CKAP2	ENSDARG00000086112	2.4769	0.0000	4.8823	0.0000

ENSG00000164430	mb21d1	ENSDARG00000021572	2.4695	0.0000	3.4894	0.0000
ENSG00000131477	ramp2	ENSDARG00000037895	2.4694	0.0000	2.0137	0.0000
ENSG00000166959	ms4a17a.6	ENSDARG00000007018	2.4692	0.0000	3.4278	0.0000
ENSG00000166959	ms4a17a.2	ENSDARG00000093546	2.4493	0.0000	2.2768	0.0000
ENSG00000109787	klf3	ENSDARG00000015495	2.4431	0.0000	1.7190	0.0000
ENSG00000178226	si:dkeyp-93a5.2	ENSDARG00000052064	2.4393	0.0000	2.1769	0.0000
ENSG00000126456	irf3	ENSDARG00000076251	2.4360	0.0000	2.9540	0.0000
ENSG00000184574	lpar5a	ENSDARG00000010384	2.4338	0.0000	2.4721	0.0000
ENSG00000164093	pitx2	ENSDARG00000036194	2.4303	0.0000	2.5969	0.0000
ENSG00000142173	col6a2	ENSDARG00000061436	2.4279	0.0000	4.0423	0.0000
ENSG00000104938	si:ch73-111e15.1	ENSDARG00000008182	2.4263	0.0000	3.0150	0.0000
ENSG00000173193	PARP14	ENSDARG00000040445	2.4215	0.0000	4.1649	0.0000
ENSG00000110651	cd81b	ENSDARG00000022437	2.4211	0.0000	3.2389	0.0000
ENSG00000100599	rin3	ENSDARG00000077618	2.4150	0.0000	2.5814	0.0000
ENSG00000184117	nipsnap1	ENSDARG00000005320	2.4060	0.0000	1.5361	0.0000
ENSG00000039537	c6	ENSDARG00000093052	2.4050	0.0000	2.3112	0.0000
ENSG00000203883	sox18	ENSDARG00000058598	2.4043	0.0000	2.1128	0.0000
ENSG00000184897	h1fx	ENSDARG00000054058	2.4042	0.0000	1.5160	0.0000
ENSG00000265972	txnipa	ENSDARG00000036107	2.4032	0.0000	3.1837	0.0000
ENSG00000188886	npsn	ENSDARG00000010423	2.3923	0.0000	1.5423	0.0000
ENSG00000143028	sypl2b	ENSDARG00000000690	2.3874	0.0000	3.1454	0.0000
ENSG00000257341	crip1	ENSDARG00000053858	2.3870	0.0000	2.6636	0.0000
ENSG00000108771	dhx58	ENSDARG00000089463	2.3833	0.0000	5.4644	0.0000
ENSG00000121634	cx50.5	ENSDARG00000069451	2.3785	0.0000	0.4854	0.3123
ENSG00000132879	si:dkey-147f3.4	ENSDARG00000092077	2.3764	0.0000	1.4493	0.0000
ENSG00000166959	ms4a17a.10	ENSDARG00000095695	2.3756	0.0000	2.9097	0.0000
ENSG00000115263	gcgb	ENSDARG00000040907	2.3539	0.0000	2.1008	0.0000
ENSG00000214102	wee2	ENSDARG00000012718	2.3472	0.0000	3.7134	0.0000
ENSG00000130222	gadd45ga	ENSDARG00000019417	2.3414	0.0000	0.6813	0.0000
ENSG00000134057	ccnb1	ENSDARG00000051923	2.3395	0.0000	4.8951	0.0000
ENSG00000119125	gda	ENSDARG00000002986	2.3314	0.0000	3.4443	0.0000
ENSG00000139626	itgb7	ENSDARG00000044318	2.3277	0.0000	4.2362	0.0000
ENSG00000107562	cxcl12a	ENSDARG00000037116	2.3257	0.0000	3.6816	0.0000
ENSG00000145365	TIFA	ENSDARG00000091111	2.3225	0.0000	2.8242	0.0000
ENSG00000103241	foxf1	ENSDARG00000015399	2.3219	0.0000	2.0589	0.0000
ENSG00000178726	THBD	ENSDARG00000092470	2.3219	0.0000	1.0240	0.0000
ENSG00000128340	rac2	ENSDARG00000038010	2.3184	0.0000	3.1600	0.0000
ENSG00000156885	cox6a2	ENSDARG00000054588	2.3108	0.0000	3.0940	0.0000
ENSG00000204983	try	ENSDARG00000042993	2.3074	0.0000	3.9212	0.0000
ENSG00000137699	ftr73	ENSDARG00000095298	2.3049	0.0000	3.8462	0.0000
ENSG00000176890	tyms	ENSDARG00000042894	2.3032	0.0000	4.6794	0.0000
ENSG00000039987	best2	ENSDARG00000079163	2.2953	0.0000	1.2429	0.0000
ENSG00000077152	ube2t	ENSDARG00000063285	2.2942	0.0000	4.2352	0.0000
ENSG00000106211	hspb1	ENSDARG00000041065	2.2866	0.0000	2.9629	0.0000
ENSG00000163131	ctssb.2	ENSDARG00000013771	2.2859	0.0000	3.4928	0.0000
ENSG00000162654	gbp1	ENSDARG00000038668	2.2811	0.0000	3.9831	0.0000
ENSG00000142405	si:ch211-66k16.28	ENSDARG00000088423	2.2801	0.0000	3.2801	0.0000
ENSG00000260314	mrc1b	ENSDARG00000038822	2.2773	0.0000	2.5652	0.0000
ENSG00000144476	ACKR3	ENSDARG00000062478	2.2768	0.0000	0.8231	0.1325
ENSG00000245848	cebpa	ENSDARG00000036074	2.2747	0.0000	3.2479	0.0000
ENSG00000088882	cpxm1a	ENSDARG00000073716	2.2746	0.0000	2.3428	0.0000
ENSG00000135604	stx11a	ENSDARG00000044039	2.2735	0.0000	3.5144	0.0000
ENSG00000242173	arhgdig	ENSDARG00000004034	2.2707	0.0000	3.6546	0.0000
ENSG00000152779	slc16a12a	ENSDARG00000017773	2.2701	0.0000	2.7891	0.0000
ENSG00000026103	fas	ENSDARG00000043586	2.2563	0.0000	3.7268	0.0000
ENSG00000155761	SPAG17	ENSDARG00000058280	2.2538	0.0000	2.1575	0.0000
ENSG00000233493	tmem238	ENSDARG00000093684	2.2533	0.0000	0.7126	0.0000
ENSG00000175946	klhl38b	ENSDARG00000040278	2.2519	0.0000	-1.7885	0.0001

ENSG00000108679	lgals3bpb	ENSDARG00000040528	2.2431	0.0000	4.1269	0.0000
ENSG00000170421	krt8	ENSDARG00000058358	2.2379	0.0000	2.5967	0.0000
ENSG00000123610	tnfaip6	ENSDARG00000093440	2.2376	0.0000	2.7331	0.0000
ENSG00000170312	cdk1	ENSDARG00000087554	2.2368	0.0000	4.8699	0.0000
ENSG00000076003	mcm6	ENSDARG00000057683	2.2350	0.0000	4.3311	0.0000
ENSG00000129151	bbox1	ENSDARG00000036135	2.2309	0.0000	0.5912	0.0000
ENSG00000125534	ppdpfb	ENSDARG00000031317	2.2260	0.0000	2.7427	0.0000
ENSG00000161800	racgap1	ENSDARG00000015460	2.2254	0.0000	4.8329	0.0000
ENSG00000135404	cd63	ENSDARG00000025147	2.2200	0.0000	3.5577	0.0000
ENSG00000137752	zgc:171731	ENSDARG00000014657	2.2192	0.0000	3.7177	0.0000
ENSG00000126759	CFP	ENSDARG00000078114	2.2190	0.0000	2.2897	0.0000
ENSG00000115414	fn1a	ENSDARG00000019815	2.2163	0.0000	4.9644	0.0000
ENSG00000103313	BX957278.1	ENSDARG00000010940	2.2161	0.0000	2.7484	0.0000
ENSG00000139352	ascl1a	ENSDARG00000038386	2.2147	0.0000	3.7843	0.0000
ENSG00000012779	alox5b.2	ENSDARG00000043089	2.2130	0.0000	6.3923	0.0000
ENSG00000100351	grap2a	ENSDARG00000006468	2.2088	0.0000	2.3454	0.0000
ENSG00000179044	exoc3l1	ENSDARG00000051899	2.2060	0.0000	2.2964	0.0000
ENSG00000100122	crybb1	ENSDARG00000068507	2.2051	0.0000	-0.1699	0.8642
ENSG00000156374	pcgf6	ENSDARG00000069681	2.2041	0.0000	1.3106	0.0000
ENSG00000187554	tlr5a	ENSDARG00000044415	2.1990	0.0000	2.4277	0.0000
ENSG00000013810	tacc3	ENSDARG00000005454	2.1950	0.0000	4.5881	0.0000
ENSG00000197635	fap	ENSDARG00000078468	2.1935	0.0000	-0.0753	0.3532
ENSG00000170298	zgc:92326	ENSDARG00000041060	2.1926	0.0000	3.7876	0.0000
ENSG00000111199	trpv4	ENSDARG00000061941	2.1917	0.0000	1.8082	0.0000
ENSG00000135205	ccdc146	ENSDARG00000004794	2.1903	0.0000	1.0875	0.0000
ENSG00000157227	mmp14b	ENSDARG00000008388	2.1865	0.0000	5.0877	0.0000
ENSG00000103966	ehd4	ENSDARG00000054302	2.1827	0.0000	2.7599	0.0000
ENSG00000129048	ackr4b	ENSDARG00000040133	2.1810	0.0000	1.9473	0.0000
ENSG00000174791	rin1a	ENSDARG00000079095	2.1791	0.0000	3.2695	0.0000
ENSG00000179348	gata2a	ENSDARG00000059327	2.1736	0.0000	1.7336	0.0000
ENSG00000156804	fbxo32	ENSDARG00000040277	2.1690	0.0000	0.8324	0.0000
ENSG00000140968	irf8	ENSDARG00000056407	2.1642	0.0000	3.1987	0.0000
ENSG00000167080	b4galnt2.1	ENSDARG00000094579	2.1615	0.0000	1.2439	0.0000
ENSG00000110047	ehd1b	ENSDARG00000014793	2.1592	0.0000	2.5416	0.0000
ENSG00000182566	CLEC17A	ENSDARG00000059049	2.1569	0.0000	3.2582	0.0000
ENSG00000124491	f13a1a.1	ENSDARG00000045453	2.1506	0.0000	1.1057	0.0000
ENSG00000128918	aldh1a2	ENSDARG00000053493	2.1487	0.0000	0.4347	0.0000
ENSG00000137672	trpc6a	ENSDARG00000056625	2.1484	0.0000	0.8288	0.0000
ENSG00000169508	gpr183a	ENSDARG00000010317	2.1444	0.0000	2.8984	0.0000
ENSG00000148834	gsto2	ENSDARG00000033285	2.1416	0.0000	2.5736	0.0000
ENSG00000158517	ncf1	ENSDARG00000033735	2.1415	0.0000	3.6480	0.0000
ENSG00000186918	znf395a	ENSDARG00000060113	2.1406	0.0000	2.6730	0.0000
ENSG00000176928	gcnt4a	ENSDARG00000035198	2.1405	0.0000	1.1699	0.0000
ENSG00000152778	ifit12	ENSDARG00000090977	2.1389	0.0000	4.7611	0.0000
ENSG00000148344	ptges	ENSDARG00000020136	2.1387	0.0000	0.4865	0.0000
ENSG00000136383	alpk3a	ENSDARG00000078989	2.1375	0.0000	1.4396	0.0000
ENSG00000120054	cpn1	ENSDARG00000005185	2.1369	0.0000	2.3856	0.0000
ENSG00000064666	cnn2	ENSDARG00000035858	2.1344	0.0000	2.0005	0.0000
ENSG00000162909	capn2b	ENSDARG00000055592	2.1340	0.0000	2.5465	0.0000
ENSG00000132958	tpte	ENSDARG00000056985	2.1296	0.0000	2.7980	0.0000
ENSG00000138772	anxa3b	ENSDARG00000044254	2.1291	0.0000	3.3197	0.0000
ENSG00000119632	zgc:123068	ENSDARG00000017489	2.1265	0.0000	5.1912	0.0000
ENSG00000131747	top2a	ENSDARG00000024488	2.1256	0.0000	4.8641	0.0000
ENSG00000185156	mfsd6l	ENSDARG00000014761	2.1234	0.0000	3.4310	0.0000
ENSG00000104228	trim35-10	ENSDARG00000033071	2.1234	0.0000	2.5325	0.0000
ENSG00000013364	mvp	ENSDARG00000021242	2.1220	0.0000	3.8263	0.0000
ENSG00000015592	stmn4l	ENSDARG00000043932	2.1216	0.0000	3.9647	0.0000
ENSG00000068079	ifi35	ENSDARG00000075643	2.1214	0.0000	4.0984	0.0000

ENSG00000143119	CD53	ENSDARG00000068233	2.1186	0.0000	3.2687	0.0000
ENSG00000116663	si:dkey-147f3.4	ENSDARG00000071029	2.1183	0.0000	1.9677	0.0000
ENSG00000177374	hic1	ENSDARG00000055493	2.1155	0.0000	2.1440	0.0000
ENSG00000116774	olfml3a	ENSDARG00000061852	2.1155	0.0000	1.7680	0.0000
ENSG00000213934	zgc:92880	ENSDARG00000069734	2.1093	0.0000	2.6545	0.0000
ENSG00000140297	gcnt3	ENSDARG00000060471	2.1069	0.0000	2.9720	0.0000
ENSG00000167460	tpm4a	ENSDARG00000023963	2.0966	0.0000	1.9959	0.0000
ENSG00000158092	nck1a	ENSDARG00000074262	2.0935	0.0000	2.4577	0.0000
ENSG00000108255	cryba1a	ENSDARG00000041141	2.0883	0.0000	-1.1699	0.0000
ENSG00000166292	TMEM100	ENSDARG00000087687	2.0875	0.0000	1.8437	0.0000
ENSG00000103313	btr09	ENSDARG00000054805	2.0834	0.0000	4.8171	0.0000
ENSG00000105499	PLA2G4C	ENSDARG00000033355	2.0816	0.0000	5.7283	0.0000
ENSG00000157601	mxa	ENSDARG00000021688	2.0780	0.0000	5.1714	0.0000
ENSG00000107485	gata3	ENSDARG00000016526	2.0764	0.0000	1.6663	0.0000
ENSG00000172724	ccl19b	ENSDARG00000039351	2.0753	0.0000	0.8365	0.1081
ENSG00000167772	angptl4	ENSDARG00000035859	2.0742	0.0000	1.4832	0.0000
ENSG00000073150	panx2	ENSDARG00000063019	2.0739	0.0000	1.7131	0.0000
ENSG00000215018	COL28A1	ENSDARG00000087087	2.0704	0.0000	2.4005	0.0000
ENSG00000184557	socs3b	ENSDARG00000026611	2.0683	0.0000	3.0915	0.0000
ENSG00000120889	tnfrsfa	ENSDARG00000004451	2.0646	0.0000	2.3826	0.0000
ENSG00000119509	invs	ENSDARG00000002213	2.0634	0.0000	2.4637	0.0000
ENSG00000117394	slc2a1b	ENSDARG00000007412	2.0602	0.0000	1.0085	0.0000
ENSG00000135926	tmbim1	ENSDARG00000058323	2.0599	0.0000	3.5768	0.0000
ENSG00000214367	haus3	ENSDARG00000061385	2.0566	0.0000	4.2913	0.0000
ENSG00000064989	calcrlb	ENSDARG00000011571	2.0561	0.0000	2.0834	0.0000
ENSG00000034510	tmsb	ENSDARG00000054911	2.0546	0.0000	6.0044	0.0000
ENSG00000179144	si:dkeyp-52c3.2	ENSDARG00000070516	2.0531	0.0000	1.9635	0.0000
ENSG00000152518	zfp36l2	ENSDARG00000021806	2.0485	0.0000	1.0076	0.0000
ENSG00000168394	tap1	ENSDARG00000079766	2.0480	0.0000	2.6121	0.0000
ENSG00000082074	FYB	ENSDARG00000044694	2.0475	0.0000	3.1759	0.0000
ENSG00000150455	tirap	ENSDARG00000074371	2.0444	0.0000	1.8074	0.0000
ENSG00000171401	krt94	ENSDARG00000044975	2.0424	0.0000	0.9445	0.0000
ENSG00000130307	ushbp1	ENSDARG00000089071	2.0413	0.0000	0.4594	0.0000
ENSG00000144959	nceh1a	ENSDARG00000020427	2.0408	0.0000	0.3547	0.1745
ENSG00000111450	stx2a	ENSDARG00000004618	2.0406	0.0000	3.1362	0.0000
ENSG00000163430	fstl1a	ENSDARG00000015559	2.0371	0.0000	2.7325	0.0000
ENSG00000117525	f3b	ENSDARG00000005673	2.0344	0.0000	1.0698	0.0000
ENSG00000088325	tpx2	ENSDARG00000078654	2.0301	0.0000	4.8117	0.0000
ENSG00000100297	mcm5	ENSDARG00000019507	2.0295	0.0000	3.8297	0.0000
ENSG00000169291	she	ENSDARG00000087956	2.0293	0.0000	2.7683	0.0000
ENSG00000077009	nmrk2	ENSDARG00000067848	2.0275	0.0000	4.6789	0.0000
ENSG00000101265	rassf2b	ENSDARG00000044044	2.0263	0.0000	1.8194	0.0000
ENSG00000110811	p3h3	ENSDARG00000057879	2.0256	0.0000	1.6172	0.0000
ENSG00000135940	cox5b2	ENSDARG00000068738	2.0230	0.0000	3.4809	0.0000
ENSG00000139192	tapbpl	ENSDARG00000058351	2.0227	0.0000	3.2122	0.0000
ENSG00000103490	pycard	ENSDARG00000040076	2.0211	0.0000	3.2072	0.0000
ENSG00000131781	fmo5	ENSDARG00000016357	2.0197	0.0000	1.9834	0.0000
ENSG00000129226	CD68	ENSDARG00000055504	2.0185	0.0000	4.4895	0.0000
ENSG00000168528	serinc2	ENSDARG00000056532	2.0181	0.0000	2.8280	0.0000
ENSG00000163499	cryba2b	ENSDARG00000041925	2.0128	0.0000	1.9475	0.0000
ENSG00000170915	paqr8	ENSDARG00000088828	2.0128	0.0000	0.6411	0.0016
ENSG00000183160	tmem119b	ENSDARG00000068036	2.0114	0.0000	2.1371	0.0000
ENSG00000135625	EGR4	ENSDARG00000077799	2.0081	0.0000	-0.1532	0.2491
ENSG00000101335	myl9a	ENSDARG00000038123	2.0047	0.0000	2.5693	0.0000
ENSG00000116990	mycla	ENSDARG00000006003	2.0000	0.0000	2.4475	0.0000
ENSG00000183044	abat	ENSDARG00000006031	1.9986	0.0000	1.5979	0.0000
ENSG00000091136	lamb1b	ENSDARG00000045524	1.9944	0.0000	1.5781	0.0000
ENSG00000249751	ecscr	ENSDARG00000087457	1.9901	0.0000	1.3376	0.0000

ENSG00000150637	cd226	ENSDARG00000045391	1.9886	0.0000	0.7313	0.0000
ENSG00000160345	C8H9orf116	ENSDARG00000058940	1.9883	0.0000	-0.0962	0.8974
ENSG00000178878	APOLD1	ENSDARG00000068030	1.9827	0.0000	2.6466	0.0000
ENSG00000137440	fgfbp1b	ENSDARG00000039963	1.9826	0.0000	2.5120	0.0000
ENSG00000105122	rasal3	ENSDARG00000089933	1.9809	0.0000	2.3930	0.0000
ENSG00000164850	gper1	ENSDARG00000074661	1.9807	0.0000	0.7988	0.0000
ENSG00000162909	capn2a	ENSDARG00000091699	1.9792	0.0000	2.8010	0.0000
ENSG00000156150	ALX3	ENSDARG00000091086	1.9786	0.0000	1.5564	0.0001
ENSG00000064932	SBNO2	ENSDARG00000016188	1.9774	0.0000	3.1966	0.0000
ENSG00000120279	myct1b	ENSDARG00000088546	1.9773	0.0000	1.4037	0.0000
ENSG00000169313	p2ry12	ENSDARG00000069945	1.9756	0.0000	2.9890	0.0000
ENSG00000106608	BX569789.2	ENSDARG00000087575	1.9746	0.0000	6.1648	0.0000
ENSG00000115415	stat1b	ENSDARG00000076182	1.9714	0.0000	4.9135	0.0000
ENSG00000165480	ska3	ENSDARG00000069917	1.9712	0.0000	4.5203	0.0000
ENSG00000172061	lrrc15	ENSDARG00000070792	1.9687	0.0000	3.3586	0.0000
ENSG00000168685	il7r	ENSDARG00000078970	1.9668	0.0000	2.9069	0.0000
ENSG00000168298	si:ch211-103n10.5	ENSDARG00000077504	1.9650	0.0000	1.8759	0.0000
ENSG00000143384	mcl1b	ENSDARG00000008363	1.9611	0.0000	0.4240	0.0000
ENSG00000174576	npas4a	ENSDARG00000055752	1.9588	0.0000	-1.6345	0.0000
ENSG00000151617	ednraa	ENSDARG00000011876	1.9585	0.0000	1.6344	0.0000
ENSG00000254087	lyn	ENSDARG00000031715	1.9579	0.0000	2.7485	0.0000
ENSG00000177105	rhogb	ENSDARG00000004301	1.9522	0.0000	2.7259	0.0000
ENSG00000166523	si:ch211-154o6.6	ENSDARG00000056379	1.9504	0.0000	2.1999	0.0000
ENSG00000156675	rab11fip1a	ENSDARG00000046124	1.9475	0.0000	0.8931	0.0807
ENSG00000136156	itm2bb	ENSDARG00000041505	1.9472	0.0000	2.5739	0.0000
ENSG00000006016	crlf1a	ENSDARG00000024365	1.9456	0.0000	3.3426	0.0000
ENSG00000137507	lrrc32	ENSDARG00000040621	1.9436	0.0000	-0.4008	0.0000
ENSG00000150681	rgs18	ENSDARG00000036106	1.9434	0.0000	2.8759	0.0000
ENSG00000125398	sox8b	ENSDARG00000037782	1.9434	0.0000	-1.1979	0.0046
ENSG00000179388	egr3	ENSDARG00000089156	1.9424	0.0000	1.1528	0.0000
ENSG00000164306	primpol	ENSDARG00000033273	1.9419	0.0000	3.7521	0.0000
ENSG00000100448	si:dkey-21e2.8	ENSDARG00000094662	1.9386	0.0000	2.5443	0.0000
ENSG00000123560	plp1b	ENSDARG00000011929	1.9369	0.0000	1.7906	0.0000
ENSG00000015285	wasb	ENSDARG00000026350	1.9368	0.0000	2.5607	0.0000
ENSG00000112499	slc22a2	ENSDARG00000030530	1.9354	0.0000	-0.0375	0.8161
ENSG00000179914	zgc:153219	ENSDARG00000054054	1.9329	0.0000	3.1699	0.0000
ENSG00000127954	steap4	ENSDARG00000055901	1.9288	0.0000	1.0728	0.0000
ENSG00000104903	tal1	ENSDARG00000019930	1.9228	0.0000	0.9386	0.0115
ENSG00000136824	smc2	ENSDARG00000017744	1.9225	0.0000	4.4335	0.0000
ENSG00000099337	kcnk6	ENSDARG00000007383	1.9216	0.0000	2.1069	0.0000
ENSG00000173221	glrx	ENSDARG00000028106	1.9190	0.0000	3.5211	0.0000
ENSG00000198734	f5	ENSDARG00000055705	1.9189	0.0000	-1.7821	0.0000
ENSG00000118515	sgk1	ENSDARG00000025522	1.9157	0.0000	0.2847	0.0000
ENSG00000116962	nid1a	ENSDARG00000068710	1.9156	0.0000	-0.2511	0.0005
ENSG00000172426	rsph9	ENSDARG00000017355	1.9156	0.0000	2.0797	0.0000
ENSG00000109099	pmp22b	ENSDARG00000060457	1.9153	0.0000	1.7217	0.0000
ENSG00000067182	tnfrsf1a	ENSDARG00000018569	1.9145	0.0000	2.3172	0.0000
ENSG00000124449	irge4	ENSDARG00000070317	1.9143	0.0000	4.5988	0.0000
ENSG00000106541	agr2	ENSDARG00000070480	1.9143	0.0000	2.5663	0.0000
ENSG00000105538	rasip1	ENSDARG00000074829	1.9104	0.0000	1.6491	0.0000
ENSG00000198959	tgm2b	ENSDARG00000074094	1.9054	0.0000	1.5995	0.0000
ENSG00000164109	mad2l1	ENSDARG00000004713	1.9054	0.0000	4.2076	0.0000
ENSG00000137699	ftr14	ENSDARG00000053293	1.9044	0.0000	3.4116	0.0000
ENSG00000015285	wasa	ENSDARG00000015149	1.9041	0.0000	2.3626	0.0000
ENSG00000105879	zgc:101783	ENSDARG00000045824	1.9027	0.0000	2.6859	0.0000
ENSG00000137752	caspa	ENSDARG00000008165	1.9018	0.0000	3.2912	0.0000
ENSG00000100605	itpk1a	ENSDARG00000013056	1.9011	0.0000	0.9196	0.0000
ENSG00000162139	neu3.4	ENSDARG00000096381	1.8981	0.0000	2.2410	0.0000

ENSG00000119138	klf9	ENSDARG00000068194	1.8970	0.0000	-1.3760	0.0000
ENSG00000171428	si:dkey-78a14.5	ENSDARG00000036067	1.8958	0.0000	1.2392	0.0000
ENSG00000135378	prrg4	ENSDARG00000030753	1.8948	0.0000	3.2143	0.0000
ENSG00000187554	tlr5b	ENSDARG00000052322	1.8935	0.0000	1.5528	0.0000
ENSG00000169684	chrna5	ENSDARG00000003420	1.8924	0.0000	2.6467	0.0000
ENSG00000122642	fkbp9	ENSDARG00000005023	1.8911	0.0000	2.7279	0.0000
ENSG00000159259	chaf1b	ENSDARG00000056473	1.8866	0.0000	4.3436	0.0000
ENSG00000205730	ITPRIPL2	ENSDARG00000054688	1.8851	0.0000	2.7733	0.0000
ENSG00000163513	TGFBR2	ENSDARG00000059363	1.8839	0.0000	2.7336	0.0000
ENSG00000142627	epha2a	ENSDARG00000017354	1.8834	0.0000	3.0704	0.0000
ENSG00000065534	mylka	ENSDARG00000034801	1.8824	0.0000	0.1908	0.0199
ENSG00000185361	tnfaip8l1	ENSDARG00000086457	1.8809	0.0000	2.3704	0.0000
ENSG00000174697	lepa	ENSDARG00000091085	1.8800	0.0000	4.9453	0.0000
ENSG00000130589	HELZ2	ENSDARG00000012600	1.8790	0.0000	4.3974	0.0000
ENSG00000137699	ftr97	ENSDARG00000055436	1.8780	0.0000	2.2663	0.0000
ENSG00000121104	fam117ab	ENSDARG00000079252	1.8697	0.0000	2.2465	0.0000
ENSG00000117791	marc2	ENSDARG00000070487	1.8689	0.0000	1.7326	0.0000
ENSG00000136982	dscc1	ENSDARG00000019907	1.8674	0.0000	3.3258	0.0000
ENSG00000164466	sfxn1	ENSDARG00000019963	1.8643	0.0000	1.6984	0.0000
ENSG00000148672	glud1a	ENSDARG00000008816	1.8626	0.0000	2.6083	0.0000
ENSG00000166148	avpr1ab	ENSDARG00000045788	1.8625	0.0000	0.9668	0.0125
ENSG00000080546	sesn1	ENSDARG00000020693	1.8624	0.0000	0.9805	0.0000
ENSG00000146918	ncapg2	ENSDARG00000060023	1.8616	0.0000	2.6669	0.0000
ENSG00000090376	irak3	ENSDARG00000053131	1.8598	0.0000	2.3770	0.0000
ENSG00000172638	efemp2a	ENSDARG00000094324	1.8594	0.0000	3.0630	0.0000
ENSG00000128052	kdr	ENSDARG00000017321	1.8540	0.0000	1.1494	0.0000
ENSG00000138160	kif11	ENSDARG00000010948	1.8509	0.0000	4.5380	0.0000
ENSG00000092068	SLC7A8	ENSDARG00000054343	1.8508	0.0000	0.2528	0.0001
ENSG00000107317	PTGDS	ENSDARG00000045979	1.8461	0.0000	0.5068	0.0000
ENSG00000105928	dfna5a	ENSDARG00000086762	1.8413	0.0000	2.2563	0.0000
ENSG00000105146	aurkb	ENSDARG00000037640	1.8406	0.0000	4.3153	0.0000
ENSG00000077420	apbb1ip	ENSDARG00000016505	1.8385	0.0000	2.4639	0.0000
ENSG00000244731	c4	ENSDARG00000015065	1.8365	0.0000	5.1497	0.0000
ENSG00000069399	bcl3	ENSDARG00000087832	1.8337	0.0000	3.0000	0.0000
ENSG00000179913	b3gnt3	ENSDARG00000058100	1.8281	0.0000	1.8281	0.0000
ENSG00000197629	mpeg1.1	ENSDARG00000055290	1.8278	0.0000	3.3267	0.0000
ENSG00000188833	entpd8	ENSDARG00000005565	1.8273	0.0000	1.3149	0.0000
ENSG00000277737	aqp7	ENSDARG00000026787	1.8260	0.0001	2.8981	0.0000
ENSG00000206384	col6a6	ENSDARG00000079752	1.8255	0.0000	0.7417	0.0000
ENSG00000027869	SH2D2A	ENSDARG00000089983	1.8244	0.0000	1.4485	0.0000
ENSG00000103313	si:ch211-247l8.10	ENSDARG00000058329	1.8231	0.0000	1.5475	0.0006
ENSG00000161940	BCL6B	ENSDARG00000069335	1.8226	0.0000	0.8273	0.0000
ENSG00000105329	tgfb1b	ENSDARG00000034895	1.8223	0.0000	1.4315	0.0000
ENSG00000164691	tagapa	ENSDARG00000002353	1.8217	0.0000	3.2672	0.0000
ENSG00000166592	rrad	ENSDARG00000052011	1.8201	0.0000	1.9403	0.0000
ENSG00000197930	ero1a	ENSDARG00000015228	1.8149	0.0000	2.6219	0.0000
ENSG00000175832	etv4	ENSDARG00000018303	1.8117	0.0000	3.0549	0.0000
ENSG00000164815	orc5	ENSDARG00000021579	1.8099	0.0000	3.2611	0.0000
ENSG00000104808	dhdhl	ENSDARG00000028336	1.8074	0.0000	2.2849	0.0000
ENSG00000158955	wnt9b	ENSDARG00000037889	1.8074	0.0000	1.8759	0.0000
ENSG00000141526	SLC16A3	ENSDARG00000028583	1.8069	0.0000	1.3254	0.0000
ENSG00000163083	inhbb	ENSDARG00000040777	1.8056	0.0000	2.7932	0.0000
ENSG00000237452	BHMG1	ENSDARG00000094059	1.8043	0.0000	1.3653	0.0000
ENSG00000137699	ftr56	ENSDARG00000030954	1.8021	0.0000	2.8439	0.0000
ENSG00000122145	tbx22	ENSDARG00000091748	1.8011	0.0000	1.3819	0.0000
ENSG00000125384	ptger2b	ENSDARG00000037033	1.8009	0.0000	0.8329	0.0002
ENSG00000187824	tmem220	ENSDARG00000088981	1.7996	0.0000	1.7029	0.0000
ENSG00000164125	fam198b	ENSDARG00000077054	1.7985	0.0000	1.6765	0.0000

ENSG00000137699	ftr57	ENSDARG00000074360	1.7975	0.0000	2.9652	0.0000
ENSG00000137825	itpka	ENSDARG00000042856	1.7969	0.0000	1.6906	0.0000
ENSG00000180921	fam83ha	ENSDARG00000068849	1.7942	0.0000	2.2675	0.0000
ENSG00000213853	emp2	ENSDARG00000044588	1.7940	0.0000	1.6897	0.0000
ENSG00000112769	lama4	ENSDARG00000020785	1.7931	0.0000	1.9766	0.0000
ENSG00000165168	cybb	ENSDARG00000056615	1.7921	0.0000	2.5520	0.0000
ENSG00000183688	fam101b	ENSDARG00000093931	1.7906	0.0000	0.9100	0.0000
ENSG00000155629	pik3ap1	ENSDARG00000078285	1.7900	0.0000	1.7054	0.0000
ENSG00000088002	sult2st3	ENSDARG00000028367	1.7890	0.0000	1.2474	0.0000
ENSG00000135517	mipb	ENSDARG00000013963	1.7866	0.0000	-0.4150	0.4996
ENSG00000187513	cx39.4	ENSDARG00000070357	1.7856	0.0000	0.9035	0.0000
ENSG00000140749	IGSF6	ENSDARG00000093074	1.7855	0.0000	2.8448	0.0000
ENSG00000163273	nppc	ENSDARG00000068126	1.7826	0.0000	1.4364	0.0000
ENSG00000167513	cdt1	ENSDARG00000051854	1.7816	0.0000	2.5629	0.0000
ENSG00000174004	nrros	ENSDARG00000071491	1.7802	0.0000	2.3591	0.0000
ENSG00000142961	mob3c	ENSDARG00000043705	1.7790	0.0000	2.5629	0.0000
ENSG00000138135	CH25H	ENSDARG00000093818	1.7790	0.0000	0.8260	0.0027
ENSG00000129173	e2f8	ENSDARG00000057323	1.7726	0.0001	4.0150	0.0000
ENSG00000110079	ms4a17a.9	ENSDARG00000043802	1.7700	0.0000	2.0647	0.0000
ENSG00000158578	alas2	ENSDARG00000038643	1.7690	0.0000	1.9010	0.0000
ENSG00000105711	SCN1B	ENSDARG00000070170	1.7655	0.0004	3.0000	0.0000
ENSG00000137965	FO704622.1	ENSDARG00000089661	1.7625	0.0000	3.5010	0.0000
ENSG00000162444	rbp7a	ENSDARG00000091906	1.7590	0.0000	2.4081	0.0000
ENSG00000166959	ms4a17a.5	ENSDARG00000092204	1.7590	0.0000	1.7914	0.0000
ENSG00000137699	ftr19	ENSDARG00000052971	1.7521	0.0000	3.6347	0.0000
ENSG00000170075	gpr37l1a	ENSDARG00000006079	1.7521	0.0000	3.4316	0.0000
ENSG00000160868	cyp3c1	ENSDARG00000015575	1.7506	0.0000	1.4082	0.0000
ENSG00000163762	tm4sf18	ENSDARG00000060668	1.7504	0.0000	-0.3716	0.0000
ENSG00000186871	ercc6l	ENSDARG00000002479	1.7500	0.0000	4.0499	0.0000
ENSG00000183486	si:dkeyp-110c12.3	ENSDARG00000051795	1.7500	0.0002	2.1255	0.0000
ENSG00000089685	birc5a	ENSDARG00000075621	1.7476	0.0000	4.0631	0.0000
ENSG00000116285	ERRFI1	ENSDARG00000086098	1.7464	0.0000	0.6210	0.0000
ENSG00000189233	NUGGC	ENSDARG00000071001	1.7454	0.0000	1.7580	0.0000
ENSG00000165029	abca1a	ENSDARG00000074635	1.7446	0.0000	2.1233	0.0000
ENSG00000130656	HBZ	ENSDARG00000045144	1.7442	0.0000	1.3785	0.0002
ENSG00000135048	tmem2	ENSDARG00000061600	1.7419	0.0000	2.4264	0.0000
ENSG00000013561	RNF14	ENSDARG00000078683	1.7414	0.0000	3.8315	0.0000
ENSG00000144354	cdca7a	ENSDARG00000077620	1.7412	0.0000	4.0228	0.0000
ENSG00000173786	cnp	ENSDARG00000070822	1.7411	0.0000	4.1391	0.0000
ENSG00000155926	sla1	ENSDARG00000054340	1.7383	0.0000	2.6615	0.0000
ENSG00000182481	kpna2	ENSDARG00000038066	1.7378	0.0000	4.4411	0.0000
ENSG00000096060	fkbp5	ENSDARG00000028396	1.7327	0.0000	-0.5672	0.0000
ENSG00000185475	tmem179b	ENSDARG00000069495	1.7321	0.0000	0.5768	0.0206
ENSG00000116745	rpe65b	ENSDARG00000094752	1.7312	0.0000	2.3045	0.0000
ENSG00000105971	cav2	ENSDARG00000052000	1.7304	0.0000	-1.3833	0.0000
ENSG00000083457	itgae.2	ENSDARG00000057787	1.7298	0.0000	2.6232	0.0000
ENSG00000187808	sowahd	ENSDARG00000087152	1.7288	0.0000	2.7239	0.0000
ENSG00000158764	itln1	ENSDARG00000007534	1.7258	0.0001	2.1069	0.0000
ENSG00000163428	lrrc58a	ENSDARG00000076773	1.7246	0.0000	-0.5659	0.0000
ENSG00000142347	myo1f	ENSDARG00000078734	1.7237	0.0000	2.5107	0.0000
ENSG00000160255	itgb2	ENSDARG00000016939	1.7231	0.0000	2.8258	0.0000
ENSG00000137699	ftr35	ENSDARG00000075355	1.7225	0.0006	4.1293	0.0000
ENSG00000137699	TRIM47	ENSDARG00000055476	1.7225	0.0000	1.3674	0.0000
ENSG00000166741	NNMT	ENSDARG00000086998	1.7208	0.0000	4.1000	0.0000
ENSG00000011478	qpctlb	ENSDARG00000062293	1.7204	0.0000	1.5257	0.0000
ENSG00000130766	sesn2	ENSDARG00000070012	1.7174	0.0000	1.3756	0.0000
ENSG00000075420	fndc3bb	ENSDARG00000062023	1.7170	0.0000	2.4424	0.0000
ENSG00000050555	lamc3	ENSDARG00000093572	1.7165	0.0000	0.5378	0.0000

ENSG00000125245	gpr18	ENSDARG00000062009	1.7146	0.0000	3.6155	0.0000
ENSG00000131969	ABHD12B	ENSDARG00000092071	1.7127	0.0000	1.0780	0.0105
ENSG00000196189	sema4aa	ENSDARG00000077103	1.7117	0.0000	2.4190	0.0000
ENSG00000162998	frzb	ENSDARG00000018383	1.7115	0.0000	2.4919	0.0000
ENSG00000180336	C3H17orf104	ENSDARG00000090664	1.7105	0.0003	2.7694	0.0000
ENSG00000092054	vmhcl	ENSDARG00000079782	1.7105	0.0000	1.7885	0.0000
ENSG00000134013	loxl2a	ENSDARG00000044010	1.7105	0.0000	0.3886	0.0382
ENSG00000064012	casp8	ENSDARG00000058325	1.7099	0.0000	3.4320	0.0000
ENSG00000177984	lcn15	ENSDARG00000067851	1.7081	0.0000	1.8809	0.0000
ENSG00000054598	foxc1a	ENSDARG00000091481	1.7066	0.0000	1.9629	0.0000
ENSG00000102034	ef1	ENSDARG00000020759	1.7063	0.0000	2.6685	0.0000
ENSG00000139132	FGD4	ENSDARG00000060248	1.7053	0.0000	2.1366	0.0000
ENSG00000010282	hhatla	ENSDARG00000039051	1.7048	0.0000	0.3135	0.0001
ENSG00000131969	ABHD12B	ENSDARG00000069828	1.7004	0.0000	1.1375	0.0037
ENSG00000101017	cd40	ENSDARG00000054968	1.6994	0.0000	3.3867	0.0000
ENSG00000154025	SLC5A10	ENSDARG00000074212	1.6959	0.0000	2.4659	0.0000
ENSG00000124104	snx21	ENSDARG00000062770	1.6956	0.0000	0.4190	0.0405
ENSG00000172059	klf11b	ENSDARG00000013794	1.6939	0.0000	1.0245	0.0000
ENSG00000109321	AREG	ENSDARG00000076853	1.6939	0.0000	-0.0875	1.0000
ENSG00000124942	ahnak	ENSDARG00000061764	1.6937	0.0000	1.6971	0.0000
ENSG00000131153	gins2	ENSDARG00000002304	1.6933	0.0000	3.0341	0.0000
ENSG00000132259	cnga4	ENSDARG00000069383	1.6930	0.0000	1.5850	0.0000
ENSG00000130726	trim33l	ENSDARG00000086126	1.6923	0.0000	2.2353	0.0000
ENSG00000130561	sagb	ENSDARG00000038378	1.6886	0.0000	-0.9912	0.0000
ENSG00000185480	parpbp	ENSDARG00000029944	1.6865	0.0000	3.1189	0.0000
ENSG00000168497	sdprb	ENSDARG00000071196	1.6856	0.0000	0.4561	0.0000
ENSG00000099875	mknk2a	ENSDARG00000011373	1.6850	0.0000	-2.0152	0.0000
ENSG00000182187	crygm1	ENSDARG00000016421	1.6845	0.0001	3.1699	0.0000
ENSG00000232258	TMEM114	ENSDARG00000038089	1.6835	0.0000	1.1520	0.0000
ENSG00000127863	tnfrsf19	ENSDARG00000041869	1.6835	0.0000	0.4634	0.0551
ENSG00000164530	PI16	ENSDARG00000046021	1.6818	0.0000	0.8745	0.0008
ENSG00000176971	fibinb	ENSDARG00000087196	1.6813	0.0000	1.9665	0.0000
ENSG00000137699	ftr04	ENSDARG00000093932	1.6781	0.0009	3.4854	0.0000
ENSG00000114854	tnnc1b	ENSDARG00000037539	1.6781	0.0009	1.3219	0.0167
ENSG00000134013	loxl2b	ENSDARG00000044074	1.6708	0.0000	1.8809	0.0000
ENSG00000155465	slc7a7	ENSDARG00000055226	1.6701	0.0000	2.7301	0.0000
ENSG00000140030	gpr65	ENSDARG00000045957	1.6688	0.0000	1.8643	0.0000
ENSG00000009790	TRAF3IP3	ENSDARG00000086708	1.6674	0.0000	1.7070	0.0000
ENSG00000204389	hsp70.3	ENSDARG00000021924	1.6660	0.0000	3.7609	0.0000
ENSG00000124713	gnmt	ENSDARG00000006840	1.6646	0.0000	1.7215	0.0000
ENSG00000109113	rab34a	ENSDARG00000045628	1.6641	0.0000	2.9557	0.0000
ENSG00000176928	GCNT4	ENSDARG00000044755	1.6630	0.0000	3.0171	0.0000
ENSG00000156398	sfxn2	ENSDARG00000017960	1.6627	0.0000	2.0698	0.0000
ENSG00000123977	daw1	ENSDARG00000021462	1.6624	0.0000	2.0635	0.0000
ENSG00000087303	nid2a	ENSDARG00000075707	1.6559	0.0000	1.3407	0.0000
ENSG00000158716	dusp23a	ENSDARG00000009844	1.6545	0.0000	2.2785	0.0000
ENSG00000197629	mpeg1.2	ENSDARG00000043093	1.6540	0.0000	3.4862	0.0000
ENSG00000126458	rras	ENSDARG00000006553	1.6537	0.0000	2.3364	0.0000
ENSG00000134690	cdca8	ENSDARG00000043137	1.6521	0.0000	4.4555	0.0000
ENSG00000160838	LRRC71	ENSDARG00000036412	1.6491	0.0000	2.6067	0.0000
ENSG00000132205	emilin2a	ENSDARG00000061196	1.6490	0.0000	1.7206	0.0000
ENSG00000160789	lmna	ENSDARG00000013415	1.6484	0.0000	1.9433	0.0000
ENSG00000135269	tes	ENSDARG00000051857	1.6477	0.0000	3.8603	0.0000
ENSG00000131233	cx55.5	ENSDARG00000069830	1.6450	0.0000	-0.4145	0.0000
ENSG00000071282	lmcd1	ENSDARG00000002002	1.6444	0.0000	3.8580	0.0000
ENSG00000272573	mustn1a	ENSDARG00000054026	1.6429	0.0000	2.2667	0.0000
ENSG00000104738	mcm4	ENSDARG00000040041	1.6425	0.0000	3.8572	0.0000
ENSG00000137699	ftr98	ENSDARG00000052332	1.6391	0.0000	1.3454	0.0000

ENSG00000144891	agtr1b	ENSDARG00000045443	1.6356	0.0000	1.8509	0.0000
ENSG00000153048	carhsp1	ENSDARG00000053129	1.6326	0.0000	1.2293	0.0000
ENSG00000144655	csrnp1a	ENSDARG00000031426	1.6324	0.0000	2.7522	0.0000
ENSG00000189060	h1f0	ENSDARG00000038559	1.6324	0.0000	2.3574	0.0000
ENSG00000142227	EMP3	ENSDARG00000036428	1.6323	0.0000	0.8074	0.0581
ENSG00000116701	ncf2	ENSDARG00000005821	1.6299	0.0000	2.5792	0.0000
ENSG00000172020	gap43	ENSDARG00000015775	1.6294	0.0000	5.7213	0.0000
ENSG00000158764	BX571811.1	ENSDARG00000095082	1.6288	0.0000	2.7529	0.0000
ENSG00000119632	si:dkey-188i13.9	ENSDARG00000074754	1.6280	0.0000	4.3735	0.0000
ENSG00000178075	GRAMD1C	ENSDARG00000076238	1.6280	0.0008	3.8074	0.0000
ENSG00000164039	bdh2	ENSDARG00000052696	1.6250	0.0000	3.0401	0.0000
ENSG00000087245	mmp2	ENSDARG00000017676	1.6242	0.0000	3.4773	0.0000
ENSG00000151702	fli1a	ENSDARG00000054632	1.6198	0.0000	1.0848	0.0000
ENSG00000052850	alx4a	ENSDARG00000088332	1.6189	0.0000	1.4657	0.0000
ENSG00000188820	fam26f	ENSDARG00000041980	1.6181	0.0000	1.6718	0.0000
ENSG00000112695	COX7A2	ENSDARG00000086341	1.6156	0.0000	1.8670	0.0000
ENSG00000176907	C5H8orf4	ENSDARG00000079497	1.6155	0.0000	1.6635	0.0000
ENSG00000184489	PTP4A3	ENSDARG00000054814	1.6132	0.0000	-0.5599	0.0000
ENSG00000149633	quo	ENSDARG00000073684	1.6118	0.0000	1.7355	0.0000
ENSG00000051523	cyba	ENSDARG00000018283	1.6096	0.0000	2.5587	0.0000
ENSG00000168056	ltbp3	ENSDARG00000035682	1.6094	0.0000	2.0743	0.0000
ENSG00000137699	ftr02	ENSDARG00000079412	1.6088	0.0000	2.9635	0.0000
ENSG00000121053	mpx	ENSDARG00000019521	1.6088	0.0000	1.4647	0.0000
ENSG00000140853	NLRC5	ENSDARG00000024631	1.6084	0.0000	2.9680	0.0000
ENSG00000157601	mxd	ENSDARG00000023369	1.6067	0.0000	1.5178	0.0000
ENSG00000182645	ccdc172	ENSDARG00000039937	1.6067	0.0000	0.6699	0.1112
ENSG00000101945	suv39h1b	ENSDARG00000055753	1.6057	0.0000	3.1239	0.0000
ENSG00000163808	kif15	ENSDARG00000012073	1.6043	0.0000	3.8504	0.0000
ENSG00000123374	cdk2	ENSDARG00000026577	1.6041	0.0000	3.5373	0.0000
ENSG00000232810	tnfb	ENSDARG00000013598	1.6027	0.0000	3.2338	0.0000
ENSG00000157554	erg	ENSDARG00000077304	1.6012	0.0000	1.2923	0.0000
ENSG00000261371	pecam1	ENSDARG00000060263	1.6001	0.0000	1.7975	0.0000
ENSG00000116678	lepr	ENSDARG00000070961	1.5999	0.0000	3.5045	0.0000
ENSG00000103522	il21r.1	ENSDARG00000069961	1.5977	0.0000	2.6515	0.0000
ENSG00000154016	grapa	ENSDARG00000005414	1.5965	0.0000	3.2658	0.0000
ENSG00000137959	si:dkeyp-9d4.2	ENSDARG00000029720	1.5954	0.0000	3.4988	0.0000
ENSG00000021355	serpinb1	ENSDARG00000055416	1.5938	0.0000	3.1761	0.0000
ENSG00000137804	nusap1	ENSDARG00000002403	1.5921	0.0000	4.6714	0.0000
ENSG00000137491	slco2b1	ENSDARG00000054609	1.5917	0.0000	2.6414	0.0000
ENSG00000100060	mfng	ENSDARG00000042925	1.5917	0.0000	2.1341	0.0000
ENSG00000197747	s100a10b	ENSDARG00000025254	1.5903	0.0000	1.8783	0.0000
ENSG00000169499	PLEKHA2	ENSDARG00000078221	1.5902	0.0000	2.4435	0.0000
ENSG00000171433	glod5	ENSDARG00000071871	1.5900	0.0000	3.4854	0.0000
ENSG00000101335	myl9b	ENSDARG00000008030	1.5894	0.0000	0.6671	0.0000
ENSG00000080819	cpox	ENSDARG00000062025	1.5894	0.0000	2.1858	0.0000
ENSG00000116016	epas1a	ENSDARG00000008697	1.5855	0.0000	-1.1783	0.0000
ENSG00000011426	anln	ENSDARG00000060917	1.5850	0.0000	3.8874	0.0000
ENSG00000101057	mybl2b	ENSDARG00000032264	1.5850	0.0000	3.5531	0.0000
ENSG00000082175	pgr	ENSDARG00000035966	1.5850	0.0000	2.2076	0.0000
ENSG00000087266	sh3bp2	ENSDARG00000021982	1.5850	0.0000	2.1988	0.0000
ENSG00000134996	ostf1	ENSDARG00000091555	1.5850	0.0000	2.1748	0.0000
ENSG00000153071	DAB2	ENSDARG00000053091	1.5850	0.0013	1.1844	0.0288
ENSG00000107447	dntt	ENSDARG00000038540	1.5850	0.0000	0.7370	0.0009
ENSG00000188716	DUPD1	ENSDARG00000039682	1.5850	0.0022	0.5850	0.4244
ENSG00000134716	cyp2ad2	ENSDARG00000021172	1.5850	0.0000	-0.0875	0.9022
ENSG00000185960	shox	ENSDARG00000025891	1.5850	0.0004	-0.2410	0.8388
ENSG00000127329	ptprb	ENSDARG00000076624	1.5810	0.0000	0.4390	0.0000
ENSG00000050165	dkk3b	ENSDARG00000070683	1.5806	0.0000	3.1612	0.0000

ENSG00000158887	mpz	ENSDARG00000038609	1.5787	0.0000	4.5375	0.0000
ENSG00000115594	CABZ01054965.1	ENSDARG00000090844	1.5782	0.0000	2.3620	0.0000
ENSG00000159251	acta1b	ENSDARG00000055618	1.5688	0.0000	2.8077	0.0000
ENSG00000169129	afap1l2	ENSDARG00000074806	1.5676	0.0000	2.2154	0.0000
ENSG00000167617	cdc42ep5	ENSDARG00000062390	1.5674	0.0000	1.9511	0.0000
ENSG00000182885	ADGRG3	ENSDARG00000074782	1.5663	0.0000	2.9383	0.0000
ENSG00000158352	shroom4	ENSDARG00000079900	1.5656	0.0000	2.1640	0.0000
ENSG00000160200	cbsa	ENSDARG00000053500	1.5648	0.0000	3.0732	0.0000
ENSG00000108556	chrne	ENSDARG00000034307	1.5648	0.0000	2.4547	0.0000
ENSG00000117724	cenpf	ENSDARG00000055133	1.5639	0.0000	4.1858	0.0000
ENSG00000128815	wdfy4	ENSDARG00000077009	1.5630	0.0000	2.3279	0.0000
ENSG00000025770	ncaph2	ENSDARG00000033757	1.5618	0.0000	3.9526	0.0000
ENSG00000138670	rasgef1bb	ENSDARG00000044251	1.5587	0.0000	1.7372	0.0000
ENSG00000121152	ncaph	ENSDARG00000061468	1.5481	0.0000	3.9794	0.0000
ENSG00000137699	ftr95	ENSDARG00000073735	1.5475	0.0000	1.9143	0.0000
ENSG00000185338	socs1b	ENSDARG00000089873	1.5425	0.0000	3.4764	0.0000
ENSG00000113263	itk	ENSDARG00000017565	1.5425	0.0000	1.6057	0.0000
ENSG00000066336	spi1a	ENSDARG00000067797	1.5412	0.0000	1.9259	0.0000
ENSG00000021355	serpinb1l2	ENSDARG00000070396	1.5384	0.0000	2.9784	0.0000
ENSG00000156970	bub1bb	ENSDARG00000074927	1.5361	0.0000	3.8235	0.0000
ENSG00000074935	tube1	ENSDARG00000043982	1.5361	0.0000	2.8227	0.0000
ENSG00000165272	aqp3b	ENSDARG00000069518	1.5343	0.0000	2.6966	0.0000
ENSG00000112667	dnph1	ENSDARG00000075730	1.5325	0.0000	3.0568	0.0000
ENSG00000165895	arhgap42a	ENSDARG00000056619	1.5299	0.0000	2.3618	0.0000
ENSG00000168802	chtf8	ENSDARG00000069045	1.5293	0.0000	2.7790	0.0000
ENSG00000167524	sgk494a	ENSDARG00000087176	1.5285	0.0000	1.6174	0.0000
ENSG00000177119	ano6	ENSDARG00000061544	1.5258	0.0000	1.8610	0.0000
ENSG00000187796	card9	ENSDARG00000067672	1.5251	0.0000	2.7527	0.0000
ENSG00000166888	stat6	ENSDARG00000015902	1.5236	0.0002	2.7142	0.0000
ENSG00000100092	sh3bp1	ENSDARG00000078119	1.5236	0.0000	2.4854	0.0000
ENSG00000113810	smc4	ENSDARG00000038882	1.5188	0.0000	3.8527	0.0000
ENSG00000126787	dlgap5	ENSDARG00000045167	1.5158	0.0000	4.0439	0.0000
ENSG00000182827	ACBD3	ENSDARG00000024602	1.5146	0.0000	2.7057	0.0000
ENSG00000060491	ogfrl2	ENSDARG00000024815	1.5135	0.0000	3.3753	0.0000
ENSG00000168918	inpp5d	ENSDARG00000074283	1.5090	0.0000	2.3138	0.0000
ENSG00000196408	noxo1b	ENSDARG00000056374	1.5071	0.0000	2.0238	0.0000
ENSG00000112378	perp	ENSDARG00000063572	1.5065	0.0000	1.6111	0.0000
ENSG00000162704	arpc5b	ENSDARG00000019062	1.5060	0.0000	1.9759	0.0000
ENSG00000122861	plaua	ENSDARG00000075265	1.5055	0.0000	3.4070	0.0000
ENSG00000137812	casc5	ENSDARG00000070239	1.5025	0.0000	3.9702	0.0000
ENSG00000105612	dnase2	ENSDARG00000073893	1.5000	0.0000	2.1755	0.0000
ENSG00000136436	calcoco2	ENSDARG00000052515	1.4957	0.0000	2.4211	0.0000
ENSG00000166851	plk1	ENSDARG00000058471	1.4956	0.0000	3.8035	0.0000
ENSG00000170340	b3gnt2a	ENSDARG00000052376	1.4935	0.0000	1.6946	0.0000
ENSG00000010278	cd9b	ENSDARG00000016691	1.4926	0.0000	1.7078	0.0000
ENSG00000102996	mmp15a	ENSDARG00000051962	1.4912	0.0000	2.7607	0.0000
ENSG00000137807	kif23	ENSDARG00000014943	1.4906	0.0000	3.7804	0.0000
ENSG00000137699	ftr23	ENSDARG00000067985	1.4906	0.0000	2.7890	0.0000
ENSG00000138182	kif20ba	ENSDARG00000071009	1.4906	0.0000	3.9458	0.0000
ENSG00000123146	adgre5b.3	ENSDARG00000089904	1.4900	0.0000	2.6554	0.0000
ENSG00000196975	anxa4	ENSDARG00000036456	1.4832	0.0000	2.6878	0.0000
ENSG00000145685	lhfpl2a	ENSDARG00000009653	1.4824	0.0000	3.7643	0.0000
ENSG00000132879	zgc:114081	ENSDARG00000057899	1.4824	0.0000	2.7517	0.0000
ENSG00000151503	ncapd3	ENSDARG00000034773	1.4820	0.0000	3.6455	0.0000
ENSG00000026025	vim	ENSDARG00000010008	1.4763	0.0000	3.2686	0.0000
ENSG00000134901	kdelc1	ENSDARG00000031971	1.4732	0.0000	1.9888	0.0000
ENSG00000145384	fabp2	ENSDARG00000006427	1.4710	0.0000	1.8543	0.0000
ENSG00000163359	col6a3	ENSDARG00000077139	1.4668	0.0000	2.2222	0.0000

ENSG00000075702	wdr62	ENSDARG00000060312	1.4598	0.0000	2.3681	0.0000
ENSG00000012779	alox5b.1	ENSDARG00000043085	1.4594	0.0004	5.0444	0.0000
ENSG00000104228	trim35-1	ENSDARG00000052037	1.4594	0.0000	3.2533	0.0000
ENSG00000128604	irf5	ENSDARG00000045681	1.4594	0.0000	2.7074	0.0000
ENSG00000186847	krt15	ENSDARG00000036840	1.4576	0.0000	2.2554	0.0000
ENSG00000080986	ndc80	ENSDARG00000071694	1.4572	0.0000	3.4880	0.0000
ENSG00000177606	jun	ENSDARG00000043531	1.4558	0.0000	3.5445	0.0000
ENSG00000131781	si:dkey-239i20.2	ENSDARG00000043518	1.4475	0.0000	1.7800	0.0000
ENSG00000138400	mdh1b	ENSDARG00000018008	1.4458	0.0000	3.0845	0.0000
ENSG00000188505	nccrp1	ENSDARG00000035326	1.4435	0.0000	1.9327	0.0000
ENSG00000155760	fzd7a	ENSDARG00000060004	1.4432	0.0000	1.7109	0.0000
ENSG00000004399	PLXND1	ENSDARG00000086057	1.4421	0.0000	1.9913	0.0000
ENSG00000004939	slc4a1a	ENSDARG00000012881	1.4421	0.0000	1.8177	0.0000
ENSG00000160783	PMF1	ENSDARG00000077822	1.4420	0.0000	3.4644	0.0000
ENSG00000161847	raver1	ENSDARG00000052617	1.4406	0.0001	2.9914	0.0000
ENSG00000177191	b3gnt2l	ENSDARG00000068219	1.4406	0.0000	2.3626	0.0000
ENSG00000184232	oafa	ENSDARG00000075851	1.4380	0.0000	1.6139	0.0000
ENSG00000137310	tcf19l	ENSDARG00000040036	1.4374	0.0000	2.4263	0.0000
ENSG00000152952	plod2	ENSDARG00000011821	1.4367	0.0000	2.3166	0.0000
ENSG00000116745	rpe65c	ENSDARG00000054420	1.4361	0.0003	2.0623	0.0000
ENSG00000107957	sh3pxd2ab	ENSDARG00000061758	1.4330	0.0000	3.0356	0.0000
ENSG00000178623	GPR35	ENSDARG00000075877	1.4330	0.0076	2.0356	0.0000
ENSG00000143228	nuf2	ENSDARG00000034624	1.4288	0.0000	3.9919	0.0000
ENSG00000165591	faah2b	ENSDARG00000054786	1.4288	0.0021	1.7914	0.0000
ENSG00000049768	foxp3a	ENSDARG00000055750	1.4286	0.0000	-2.0728	0.0000
ENSG00000167123	cercam	ENSDARG00000079049	1.4241	0.0000	2.0929	0.0000
ENSG00000089820	arhgap4a	ENSDARG00000039265	1.4239	0.0000	2.1366	0.0000
ENSG00000129534	mis18bp1	ENSDARG00000042563	1.4238	0.0000	3.4498	0.0000
ENSG00000142867	bcl10	ENSDARG00000077130	1.4215	0.0000	2.7162	0.0000
ENSG00000183763	traip	ENSDARG00000011262	1.4194	0.0000	2.7696	0.0000
ENSG00000127415	idua	ENSDARG00000062904	1.4167	0.0000	1.7905	0.0000
ENSG00000117632	stmn1a	ENSDARG00000004169	1.4150	0.0000	3.7296	0.0000
ENSG00000010319	sema3bl	ENSDARG00000007560	1.4150	0.0000	1.8875	0.0000
ENSG00000134762	dsc2l	ENSDARG00000039677	1.4126	0.0000	2.1102	0.0000
ENSG00000152137	hspb8	ENSDARG00000058365	1.4120	0.0000	3.4793	0.0000
ENSG00000138185	entpd1	ENSDARG00000045066	1.4118	0.0000	1.8906	0.0000
ENSG00000126267	cox6b1	ENSDARG00000045230	1.4099	0.0000	2.9897	0.0000
ENSG00000158270	colec12	ENSDARG00000061140	1.4097	0.0000	1.8750	0.0000
ENSG00000135862	lamc1	ENSDARG00000036279	1.4085	0.0000	1.8100	0.0000
ENSG00000237649	kifc1	ENSDARG00000001558	1.4047	0.0000	3.9590	0.0000
ENSG00000164049	FBXW12	ENSDARG00000058871	1.4040	0.0000	2.5743	0.0000
ENSG00000164307	erap1b	ENSDARG00000021859	1.3996	0.0000	2.9748	0.0000
ENSG00000137699	ftr93	ENSDARG00000039852	1.3959	0.0000	2.9094	0.0000
ENSG00000102901	CENPT	ENSDARG00000091271	1.3953	0.0000	3.2021	0.0000
ENSG00000139445	foxn4	ENSDARG00000010591	1.3893	0.0000	4.2886	0.0000
ENSG00000140368	pstpip1b	ENSDARG00000053568	1.3890	0.0001	1.9115	0.0000
ENSG00000140988	rps2	ENSDARG00000077291	1.3859	0.0000	2.8131	0.0000
ENSG00000092140	g2e3	ENSDARG00000001313	1.3847	0.0000	3.8016	0.0000
ENSG00000123219	cenpk	ENSDARG00000039616	1.3814	0.0000	3.0513	0.0000
ENSG00000143344	rgl1	ENSDARG00000005989	1.3810	0.0000	2.6121	0.0000
ENSG00000023839	abcc2	ENSDARG00000014031	1.3809	0.0000	1.5954	0.0000
ENSG00000142945	kif2c	ENSDARG00000076228	1.3796	0.0000	3.8651	0.0000
ENSG00000163430	fstl1b	ENSDARG00000039576	1.3795	0.0000	1.8869	0.0000
ENSG00000148584	a1cf	ENSDARG00000002968	1.3785	0.0113	2.1699	0.0000
ENSG00000184113	cldn5a	ENSDARG00000043716	1.3781	0.0000	2.0382	0.0000
ENSG00000197943	plcg2	ENSDARG00000068763	1.3773	0.0000	2.0265	0.0000
ENSG00000170298	lgals9l1	ENSDARG00000025903	1.3772	0.0000	2.9824	0.0000
ENSG00000109991	p2rx3a	ENSDARG00000010477	1.3750	0.0000	2.5774	0.0000

ENSG00000213928	irf9	ENSDARG00000016457	1.3741	0.0000	2.3571	0.0000
ENSG00000147168	il2rga	ENSDARG00000068858	1.3733	0.0000	1.7916	0.0000
ENSG00000162777	dennd2da	ENSDARG00000058666	1.3724	0.0000	3.6224	0.0000
ENSG00000111679	ptpn6	ENSDARG00000089043	1.3706	0.0000	2.3067	0.0000
ENSG00000171223	junba	ENSDARG00000074378	1.3672	0.0000	1.9768	0.0000
ENSG00000135355	cx52.6	ENSDARG00000034930	1.3642	0.0000	-2.8081	0.0000
ENSG00000118194	tnnt2c	ENSDARG00000032242	1.3626	0.0000	3.0875	0.0000
ENSG00000148677	ankrd1a	ENSDARG00000075263	1.3626	0.0026	1.8651	0.0000
ENSG00000136448	nmt1b	ENSDARG00000057206	1.3626	0.0000	1.7318	0.0000
ENSG00000005022	si:dkey-251i10.1	ENSDARG00000042521	1.3612	0.0000	2.3563	0.0000
ENSG00000152229	pstpip2	ENSDARG00000089569	1.3604	0.0000	1.9403	0.0000
ENSG00000122585	npy	ENSDARG00000036222	1.3564	0.0000	2.3390	0.0000
ENSG00000007314	scn4ab	ENSDARG00000034588	1.3550	0.0000	5.0071	0.0000
ENSG00000163694	rbm47	ENSDARG00000061985	1.3548	0.0000	2.5896	0.0000
ENSG00000166153	DEPDC4	ENSDARG00000089908	1.3536	0.0000	3.9975	0.0000
ENSG00000175279	apitd1	ENSDARG00000070485	1.3505	0.0000	2.5484	0.0000
ENSG00000142273	cblc	ENSDARG00000078217	1.3447	0.0000	1.7190	0.0000
ENSG00000134817	aplnrb	ENSDARG00000036670	1.3440	0.0000	2.7059	0.0000
ENSG00000189433	cx34.4	ENSDARG00000075854	1.3440	0.0000	1.9578	0.0000
ENSG00000065833	me1	ENSDARG00000053215	1.3425	0.0000	1.7613	0.0000
ENSG00000035862	timp2a	ENSDARG00000061226	1.3399	0.0000	2.0604	0.0000
ENSG00000102384	cenpi	ENSDARG00000052565	1.3366	0.0000	2.8089	0.0000
ENSG00000115935	wipf1b	ENSDARG00000071084	1.3351	0.0000	2.1356	0.0000
ENSG00000172578	klhl6	ENSDARG00000026990	1.3344	0.0000	2.3188	0.0000
ENSG00000170684	znf296	ENSDARG00000077897	1.3318	0.0000	2.1476	0.0000
ENSG00000167014	C7H15orf43	ENSDARG00000039477	1.3306	0.0000	2.2130	0.0000
ENSG00000166483	wee1	ENSDARG00000093864	1.3266	0.0000	2.0013	0.0000
ENSG00000183979	npb	ENSDARG00000078828	1.3242	0.0000	2.6672	0.0000
ENSG00000181333	hephl1a	ENSDARG00000059231	1.3237	0.0000	3.1780	0.0000
ENSG00000064012	casp8l1	ENSDARG00000058341	1.3219	0.0079	2.7370	0.0000
ENSG00000135406	prph	ENSDARG00000028306	1.3197	0.0000	4.5920	0.0000
ENSG00000066336	spi1b	ENSDARG00000000767	1.3193	0.0000	2.8176	0.0000
ENSG00000185432	si:ch211-173n18.4	ENSDARG00000060889	1.3184	0.0000	1.7582	0.0000
ENSG00000151247	eif4eb	ENSDARG00000013274	1.3170	0.0000	1.8953	0.0000
ENSG00000136810	txn	ENSDARG00000044125	1.3142	0.0000	4.6140	0.0000
ENSG00000043462	lcp2a	ENSDARG00000055955	1.3132	0.0000	2.5021	0.0000
ENSG00000133789	swap70b	ENSDARG00000057286	1.3119	0.0000	2.1967	0.0000
ENSG00000024526	depdc1a	ENSDARG00000018165	1.3115	0.0000	2.9336	0.0000
ENSG00000132669	rin2	ENSDARG00000074589	1.3106	0.0000	1.8188	0.0000
ENSG00000171848	rrm2	ENSDARG00000020711	1.3105	0.0000	2.8276	0.0000
ENSG00000163491	nek10	ENSDARG00000025335	1.3093	0.0000	1.9961	0.0000
ENSG00000133433	gstt1a	ENSDARG00000042428	1.3084	0.0000	3.4348	0.0000
ENSG00000182010	rtkn2a	ENSDARG00000053558	1.3081	0.0000	2.8389	0.0000
ENSG00000158792	spata2l	ENSDARG00000068814	1.3065	0.0000	1.7427	0.0000
ENSG00000177464	gpr4	ENSDARG00000079479	1.3049	0.0015	1.7705	0.0000
ENSG00000138944	si:dkey-14k9.2	ENSDARG00000045594	1.3043	0.0000	1.7306	0.0000
ENSG00000166920	C18H15orf48	ENSDARG00000092903	1.3009	0.0000	5.2197	0.0000
ENSG00000196924	flna	ENSDARG00000074201	1.3005	0.0000	2.2972	0.0000
ENSG00000134216	chia.3	ENSDARG00000009612	1.2996	0.0001	1.8995	0.0000
ENSG00000168267	ptf1a	ENSDARG00000014479	1.2983	0.0000	3.5320	0.0000
ENSG00000155366	rhoad	ENSDARG00000018328	1.2965	0.0000	2.2314	0.0000
ENSG00000135069	psat1	ENSDARG00000016733	1.2964	0.0000	2.0845	0.0000
ENSG00000151135	tmem263	ENSDARG00000030465	1.2955	0.0000	1.9443	0.0000
ENSG00000147065	msna	ENSDARG00000058128	1.2947	0.0000	2.0844	0.0000
ENSG00000031081	arhgap31	ENSDARG00000059472	1.2943	0.0000	1.9773	0.0000
ENSG00000003137	cyp26b1	ENSDARG00000077121	1.2928	0.0000	2.5454	0.0000
ENSG00000114544	SLC41A3	ENSDARG00000061272	1.2873	0.0000	1.7483	0.0000
ENSG00000115598	CABZ01054962.1	ENSDARG00000088672	1.2787	0.0000	1.7035	0.0000

ENSG00000177697	cd151l	ENSDARG00000068629	1.2773	0.0000	3.8996	0.0000
ENSG00000138778	cenpe	ENSDARG00000063385	1.2768	0.0000	3.2911	0.0000
ENSG00000113070	hbegfb	ENSDARG00000031246	1.2752	0.0000	3.2299	0.0000
ENSG00000174442	zwilch	ENSDARG00000057100	1.2744	0.0000	2.4594	0.0000
ENSG00000110169	hpx	ENSDARG00000012609	1.2713	0.0000	2.3804	0.0000
ENSG00000168899	vamp5	ENSDARG00000068262	1.2692	0.0000	2.0914	0.0000
ENSG00000136830	fam129bb	ENSDARG00000008026	1.2691	0.0000	2.3094	0.0000
ENSG00000173207	cks1b	ENSDARG00000007971	1.2645	0.0000	3.3166	0.0000
ENSG00000186994	kank3	ENSDARG00000076566	1.2630	0.0243	1.8074	0.0002
ENSG00000057294	pkp2	ENSDARG00000023026	1.2604	0.0000	1.9457	0.0000
ENSG00000184113	cldn5b	ENSDARG00000014059	1.2598	0.0000	2.7233	0.0000
ENSG00000085840	orc1	ENSDARG00000039217	1.2594	0.0000	2.2639	0.0000
ENSG00000219200	rnaseka	ENSDARG00000069461	1.2589	0.0000	2.7146	0.0000
ENSG00000111144	lta4h	ENSDARG00000006029	1.2589	0.0000	2.4580	0.0000
ENSG00000197971	mbpb	ENSDARG00000089413	1.2584	0.0000	2.4005	0.0000
ENSG00000166710	b2ml	ENSDARG00000015887	1.2582	0.0000	1.8437	0.0000
ENSG00000075624	actb1	ENSDARG00000037746	1.2582	0.0000	2.3882	0.0000
ENSG00000140750	arhgap17b	ENSDARG00000062263	1.2578	0.0004	1.9842	0.0000
ENSG00000143891	galm	ENSDARG00000057630	1.2563	0.0000	1.6825	0.0000
ENSG00000091128	lamb4	ENSDARG00000039133	1.2510	0.0000	3.3472	0.0000
ENSG00000121104	fam117aa	ENSDARG00000078640	1.2510	0.0000	1.7991	0.0000
ENSG00000113790	ehhadh	ENSDARG00000070029	1.2505	0.0000	2.1532	0.0000
ENSG00000133115	stoml3b	ENSDARG00000057035	1.2497	0.0000	3.4004	0.0000
ENSG00000184937	wt1b	ENSDARG00000007990	1.2444	0.0000	3.0992	0.0000
ENSG00000165029	abca1b	ENSDARG00000079009	1.2421	0.0000	2.9853	0.0000
ENSG00000178078	stap2a	ENSDARG00000092810	1.2413	0.0000	1.6663	0.0000
ENSG00000124523	sirt5	ENSDARG00000039684	1.2403	0.0000	-3.3692	0.0000
ENSG00000185272	rbm11	ENSDARG00000090202	1.2398	0.0000	1.7786	0.0000
ENSG00000150093	itgb1a	ENSDARG00000071863	1.2396	0.0000	1.7288	0.0000
ENSG00000168310	irf2	ENSDARG00000040465	1.2395	0.0000	1.9490	0.0000
ENSG00000119630	pgfb	ENSDARG00000076767	1.2395	0.0000	1.8524	0.0000
ENSG00000065675	prkcq	ENSDARG00000034173	1.2379	0.0000	2.4470	0.0000
ENSG00000181938	gins3	ENSDARG00000038847	1.2352	0.0000	2.7097	0.0000
ENSG00000180448	hmha1b	ENSDARG00000062049	1.2323	0.0000	1.9642	0.0000
ENSG00000181195	penka	ENSDARG00000004869	1.2322	0.0000	2.3501	0.0000
ENSG00000108479	galk1	ENSDARG00000028088	1.2321	0.0000	2.4135	0.0000
ENSG00000177084	pole	ENSDARG00000058533	1.2317	0.0000	3.3017	0.0000
ENSG00000122121	xpnpep2	ENSDARG00000026017	1.2316	0.0000	1.8950	0.0000
ENSG00000231925	TAPBP	ENSDARG00000045011	1.2295	0.0000	2.2653	0.0000
ENSG00000197355	uap1l1	ENSDARG00000013082	1.2273	0.0000	1.7726	0.0000
ENSG00000132541	hrsp12	ENSDARG00000035882	1.2264	0.0000	1.8827	0.0000
ENSG00000034533	aste1	ENSDARG00000070132	1.2253	0.0000	4.2078	0.0000
ENSG00000174238	pitpnaa	ENSDARG00000039490	1.2243	0.0000	2.7959	0.0000
ENSG00000090932	dlc	ENSDARG00000002336	1.2231	0.0000	2.6307	0.0000
ENSG00000008382	mpnd	ENSDARG00000061989	1.2210	0.0000	2.1248	0.0000
ENSG00000204267	si:dkey-57h18.2	ENSDARG00000033446	1.2205	0.0000	2.0815	0.0000
ENSG00000182791	ccdc87	ENSDARG00000045683	1.2168	0.0000	3.5914	0.0000
ENSG00000123338	NCKAP1L	ENSDARG00000075748	1.2154	0.0000	2.2621	0.0000
ENSG00000177600	rplp2l	ENSDARG00000011201	1.2154	0.0000	3.1836	0.0000
ENSG00000136560	tank	ENSDARG00000067723	1.2130	0.0000	1.7500	0.0000
ENSG00000179636	tppp2	ENSDARG00000035338	1.2119	0.0000	2.3982	0.0000
ENSG00000134250	notch2	ENSDARG00000043130	1.2113	0.0000	1.6304	0.0000
ENSG00000243480	zgc:92137	ENSDARG00000009443	1.2081	0.0001	2.5850	0.0000
ENSG00000169504	clic4	ENSDARG00000022995	1.2080	0.0000	1.6344	0.0000
ENSG00000174307	phlda3	ENSDARG00000037804	1.2074	0.0000	2.1748	0.0000
ENSG00000176887	sox11b	ENSDARG00000095743	1.2068	0.0000	4.2126	0.0000
ENSG00000055332	pkz	ENSDARG00000052396	1.2007	0.0000	2.9535	0.0000
ENSG00000163531	NFASC	ENSDARG00000056910	1.2005	0.0000	2.4857	0.0000

ENSG00000179163	fuca1.2	ENSDARG00000035879	1.1996	0.0000	2.4561	0.0000
ENSG00000196932	tmem26a	ENSDARG00000077979	1.1993	0.0003	2.1993	0.0000
ENSG00000171848	rrm2	ENSDARG00000078069	1.1986	0.0000	3.4692	0.0000
ENSG00000168348	INSM2	ENSDARG00000020655	1.1979	0.0000	3.1438	0.0000
ENSG00000173404	insm1a	ENSDARG00000091756	1.1974	0.0000	3.6614	0.0000
ENSG00000132669	RIN2	ENSDARG00000077040	1.1971	0.0000	1.5850	0.0000
ENSG00000119888	epcam	ENSDARG00000040534	1.1926	0.0016	3.0000	0.0000
ENSG00000110057	unc93b1	ENSDARG00000069114	1.1926	0.0000	2.2654	0.0000
ENSG00000145861	c1qtnf2	ENSDARG00000059944	1.1926	0.0016	1.7162	0.0000
ENSG00000139278	glipr1b	ENSDARG00000030078	1.1923	0.0000	2.3021	0.0000
ENSG00000162694	extl2	ENSDARG00000063191	1.1909	0.0000	2.6075	0.0000
ENSG00000107798	lipf	ENSDARG00000018529	1.1850	0.0000	2.1236	0.0000
ENSG00000112208	bag2	ENSDARG00000035005	1.1844	0.0001	1.6699	0.0000
ENSG00000007944	mylipb	ENSDARG00000055118	1.1811	0.0000	-2.7221	0.0000
ENSG00000198719	dld	ENSDARG00000020219	1.1802	0.0000	2.6304	0.0000
ENSG00000100911	psme2	ENSDARG00000033144	1.1759	0.0000	2.5197	0.0000
ENSG00000006062	MAP3K14	ENSDARG00000074060	1.1737	0.0000	1.5952	0.0000
ENSG00000184060	adap2	ENSDARG00000070565	1.1722	0.0000	2.6398	0.0000
ENSG00000102181	cd99	ENSDARG00000051975	1.1714	0.0000	2.1013	0.0000
ENSG00000108639	syngr2a	ENSDARG00000052429	1.1699	0.0000	2.3219	0.0000
ENSG00000101825	mxra5a	ENSDARG00000074815	1.1688	0.0000	1.6566	0.0000
ENSG00000125775	sdcbp2	ENSDARG00000012513	1.1683	0.0000	2.2570	0.0000
ENSG00000059378	parp12a	ENSDARG00000042496	1.1667	0.0000	3.3551	0.0000
ENSG00000197496	slc2a10	ENSDARG00000090820	1.1626	0.0000	2.5470	0.0000
ENSG00000100219	xbp1	ENSDARG00000035622	1.1621	0.0000	1.9857	0.0000
ENSG00000109805	ncapg	ENSDARG00000070109	1.1568	0.0000	3.6927	0.0000
ENSG00000169136	atf5b	ENSDARG00000077785	1.1566	0.0000	2.8972	0.0000
ENSG00000164434	fabp7a	ENSDARG00000007697	1.1489	0.0000	4.1453	0.0000
ENSG00000185507	irf7	ENSDARG00000045661	1.1489	0.0000	2.7933	0.0000
ENSG00000157510	afap1l1b	ENSDARG00000056453	1.1477	0.0000	2.4818	0.0000
ENSG00000155363	mov10b.1	ENSDARG00000061177	1.1472	0.0000	3.1225	0.0000
ENSG00000162881	hcar1-3	ENSDARG00000062874	1.1468	0.0004	1.6020	0.0000
ENSG00000094804	cdc6	ENSDARG00000009942	1.1465	0.0000	2.9698	0.0000
ENSG00000138119	myof	ENSDARG00000006112	1.1461	0.0000	3.0777	0.0000
ENSG00000028137	tnfrsf1b	ENSDARG00000070165	1.1444	0.0000	2.4386	0.0000
ENSG00000176381	prr18	ENSDARG00000093768	1.1376	0.0000	2.5587	0.0000
ENSG00000100985	mmp9	ENSDARG00000042816	1.1364	0.0000	2.8863	0.0000
ENSG00000112936	c7a	ENSDARG00000042172	1.1362	0.0000	1.9799	0.0000
ENSG00000146374	rspo3	ENSDARG00000070081	1.1360	0.0000	2.6718	0.0000
ENSG00000057657	prdm1a	ENSDARG00000002445	1.1292	0.0000	3.8127	0.0000
ENSG00000137462	tlr2	ENSDARG00000037758	1.1268	0.0000	1.9399	0.0000
ENSG00000174021	gng5	ENSDARG00000039830	1.1260	0.0000	2.4603	0.0000
ENSG00000100139	micall1b	ENSDARG00000040502	1.1255	0.0000	1.7388	0.0000
ENSG00000184661	CDCA2	ENSDARG00000068759	1.1206	0.0000	2.9201	0.0000
ENSG00000140931	cmtm3	ENSDARG00000008811	1.1203	0.0000	1.8103	0.0000
ENSG00000134817	aplnra	ENSDARG00000002172	1.1198	0.0000	2.6208	0.0000
ENSG00000139631	csad	ENSDARG00000026348	1.1169	0.0000	2.7993	0.0000
ENSG00000006611	ush1c	ENSDARG00000051876	1.1148	0.0000	1.9922	0.0000
ENSG00000158417	eif5b	ENSDARG00000016548	1.1127	0.0000	1.7807	0.0000
ENSG00000169679	bub1	ENSDARG00000077029	1.1110	0.0000	3.7698	0.0000
ENSG00000125398	sox9b	ENSDARG00000043923	1.1098	0.0000	1.7247	0.0000
ENSG00000101160	ctsz	ENSDARG00000043081	1.1086	0.0000	2.9350	0.0000
ENSG00000089157	rplp0	ENSDARG00000051783	1.1063	0.0000	2.7491	0.0000
ENSG00000156802	atad2	ENSDARG00000076590	1.1061	0.0000	2.5891	0.0000
ENSG00000243910	tuba1b	ENSDARG00000045367	1.0998	0.0000	3.2443	0.0000
ENSG00000172732	mus81	ENSDARG00000069326	1.0995	0.0043	3.3890	0.0000
ENSG00000162704	arpc5a	ENSDARG00000039142	1.0978	0.0000	2.4246	0.0000
ENSG00000167771	rcor2	ENSDARG00000008278	1.0975	0.0000	3.1552	0.0000

ENSG00000060558	gna15.2	ENSDARG00000056654	1.0952	0.0000	4.2047	0.0000
ENSG00000159251	acta1a	ENSDARG00000036371	1.0941	0.0000	1.7074	0.0000
ENSG00000130222	gadd45gb.1	ENSDARG00000016725	1.0938	0.0000	2.8150	0.0000
ENSG00000161888	spc24	ENSDARG00000093622	1.0922	0.0000	2.8497	0.0000
ENSG00000143627	PKLR	ENSDARG00000042010	1.0913	0.0000	1.8365	0.0000
ENSG00000017483	slc38a5b	ENSDARG00000014587	1.0885	0.0000	1.7018	0.0000
ENSG00000177105	rhoga	ENSDARG00000068653	1.0882	0.0000	2.2418	0.0000
ENSG00000049245	vamp3	ENSDARG00000070161	1.0878	0.0000	1.7238	0.0000
ENSG00000170017	alcama	ENSDARG00000026531	1.0871	0.0000	1.7911	0.0000
ENSG00000138964	parvg	ENSDARG00000067711	1.0865	0.0000	1.8230	0.0000
ENSG00000171522	ptger4b	ENSDARG00000035415	1.0839	0.0000	1.6442	0.0000
ENSG00000103855	cd276	ENSDARG00000003061	1.0837	0.0000	2.5705	0.0000
ENSG00000168028	rpsa	ENSDARG00000019181	1.0832	0.0000	2.1720	0.0000
ENSG00000087076	hsd17b14	ENSDARG00000054842	1.0813	0.0000	2.2134	0.0000
ENSG00000168078	pbk	ENSDARG00000007221	1.0794	0.0000	3.9249	0.0000
ENSG00000188312	cenpp	ENSDARG00000044135	1.0789	0.0000	1.7803	0.0000
ENSG00000197594	enpp1	ENSDARG00000005789	1.0783	0.0000	1.6577	0.0000
ENSG00000152527	plekhh2	ENSDARG00000040851	1.0768	0.0000	1.9868	0.0000
ENSG00000117691	nenf	ENSDARG00000040192	1.0709	0.0000	2.4490	0.0000
ENSG00000137699	ftr22	ENSDARG00000076969	1.0704	0.0708	3.7549	0.0000
ENSG00000075429	cacng5b	ENSDARG00000039240	1.0695	0.0000	1.9018	0.0000
ENSG00000100228	rab36	ENSDARG00000014058	1.0686	0.0000	2.6144	0.0000
ENSG00000043355	zic2b	ENSDARG00000037178	1.0622	0.0000	4.2031	0.0000
ENSG00000067066	si:dkey-68o6.4	ENSDARG00000090449	1.0589	0.0470	2.5236	0.0000
ENSG00000152240	haus1	ENSDARG00000058968	1.0589	0.0000	2.4715	0.0000
ENSG00000263528	ikbke	ENSDARG00000070606	1.0566	0.0000	2.0731	0.0000
ENSG00000241343	rpl36a	ENSDARG00000058105	1.0531	0.0000	2.3942	0.0000
ENSG00000172380	gng12a	ENSDARG00000003375	1.0531	0.0001	2.2095	0.0000
ENSG00000147316	mcph1	ENSDARG00000058372	1.0480	0.0000	2.7079	0.0000
ENSG00000013297	cldn11b	ENSDARG00000030723	1.0473	0.0000	2.9466	0.0000
ENSG00000198901	prc1b	ENSDARG00000005993	1.0435	0.0000	2.7733	0.0000
ENSG00000116157	gpx7	ENSDARG00000091511	1.0424	0.0000	2.2147	0.0000
ENSG00000219626	fam228a	ENSDARG00000042936	1.0418	0.0175	2.4203	0.0000
ENSG00000174748	rpl15	ENSDARG00000009285	1.0410	0.0000	2.5517	0.0000
ENSG00000165806	casp7	ENSDARG00000091836	1.0408	0.0000	2.2714	0.0000
ENSG00000188263	il17rel	ENSDARG00000089131	1.0403	0.0000	4.3355	0.0000
ENSG00000129474	ajuba	ENSDARG00000077842	1.0397	0.0000	2.3073	0.0000
ENSG00000103811	ctsh	ENSDARG00000041108	1.0379	0.0000	2.2950	0.0000
ENSG00000005020	skap2	ENSDARG00000000906	1.0377	0.0000	2.0743	0.0000
ENSG00000133321	zgc:154040	ENSDARG00000093006	1.0375	0.0119	1.8814	0.0000
ENSG00000143418	cers2b	ENSDARG00000058992	1.0344	0.0000	1.7754	0.0000
ENSG00000130595	tnnt3b	ENSDARG00000068457	1.0339	0.0081	3.9827	0.0000
ENSG00000214711	zgc:162184	ENSDARG00000077217	1.0334	0.0000	1.7347	0.0000
ENSG00000163507	zgc:174160	ENSDARG00000058260	1.0323	0.0000	2.1889	0.0000
ENSG00000100162	pane1	ENSDARG00000024681	1.0316	0.0000	2.1679	0.0000
ENSG00000176907	C10H8orf4	ENSDARG00000076858	1.0311	0.0000	1.7847	0.0000
ENSG00000134283	PPHLN1	ENSDARG00000088062	1.0310	0.0000	2.2998	0.0000
ENSG00000148120	C8H9orf3	ENSDARG00000068461	1.0310	0.0056	2.0614	0.0000
ENSG00000108604	smarcd2	ENSDARG00000088877	1.0296	0.0000	2.3467	0.0000
ENSG00000142552	rcn3	ENSDARG00000037961	1.0290	0.0000	2.6075	0.0000
ENSG00000137818	rplp1	ENSDARG00000021864	1.0234	0.0000	2.6930	0.0000
ENSG00000136783	nipsnap3a	ENSDARG00000020387	1.0227	0.0000	1.8649	0.0000
ENSG00000147246	htr2cl2	ENSDARG00000013210	1.0213	0.0000	1.9528	0.0000
ENSG00000146535	gna12a	ENSDARG00000025826	1.0183	0.0000	1.7952	0.0000
ENSG00000165487	micu2	ENSDARG00000009939	1.0179	0.0000	1.6127	0.0000
ENSG00000177602	GSG2	ENSDARG00000092290	1.0155	0.0000	3.3021	0.0000
ENSG00000136040	plxnc1	ENSDARG00000015278	1.0151	0.0000	1.6406	0.0000
ENSG00000111206	foxm1	ENSDARG00000003200	1.0141	0.0000	3.0487	0.0000

ENSG00000137699	TRIM47	ENSDARG00000037190	1.0120	0.0000	2.6088	0.0000
ENSG00000180921	fam83hb	ENSDARG00000060830	1.0102	0.0000	1.9586	0.0000
ENSG00000162144	cyb561a3a	ENSDARG00000056281	1.0093	0.0000	1.6855	0.0000
ENSG00000090932	dlb	ENSDARG00000004232	1.0088	0.0000	3.3051	0.0000
ENSG00000010292	ncapd2	ENSDARG00000005058	1.0077	0.0000	3.0924	0.0000
ENSG00000121310	echdc2	ENSDARG00000016607	1.0076	0.0000	2.6038	0.0000
ENSG00000075624	actb2	ENSDARG00000037870	1.0073	0.0000	1.7535	0.0000
ENSG00000112394	slc16a10	ENSDARG00000020984	1.0038	0.0000	1.7515	0.0000
ENSG00000189143	cldna	ENSDARG00000069888	1.0000	0.0111	2.3356	0.0000
ENSG00000125691	rpl23	ENSDARG00000053457	0.9980	0.0000	2.5369	0.0000
ENSG00000144868	tmem108	ENSDARG00000090230	0.9974	0.0000	2.2096	0.0000
ENSG00000180370	pak2a	ENSDARG00000068177	0.9927	0.0000	1.6056	0.0000
ENSG00000151690	mfsd6a	ENSDARG00000032318	0.9902	0.0000	1.8295	0.0000
ENSG00000122406	rpl5b	ENSDARG00000015862	0.9897	0.0000	2.3519	0.0000
ENSG00000124766	sox4a	ENSDARG00000004588	0.9878	0.0000	2.4369	0.0000
ENSG00000182899	rpl35a	ENSDARG00000088030	0.9862	0.0000	2.2174	0.0000
ENSG00000110031	LPXN	ENSDARG00000079315	0.9852	0.0001	1.9852	0.0000
ENSG00000105639	jak3	ENSDARG00000010252	0.9826	0.0000	1.7168	0.0000
ENSG00000125384	ptger2a	ENSDARG00000011434	0.9819	0.0004	2.1859	0.0000
ENSG00000116132	prrx1a	ENSDARG00000033971	0.9765	0.0023	1.9647	0.0000
ENSG00000148180	gsna	ENSDARG00000011459	0.9718	0.0000	2.2821	0.0000
ENSG00000267534	s1pr2	ENSDARG00000036548	0.9698	0.0000	2.2627	0.0000
ENSG00000148053	ntrk2a	ENSDARG00000059897	0.9641	0.0000	1.9637	0.0000
ENSG00000168071	ccdc88b	ENSDARG00000076189	0.9638	0.0000	1.9964	0.0000
ENSG00000167842	mis12	ENSDARG00000026454	0.9635	0.0183	3.2479	0.0000
ENSG00000042980	adam28	ENSDARG00000035514	0.9615	0.0222	2.4103	0.0000
ENSG00000004975	dvl2	ENSDARG00000056184	0.9612	0.0000	2.2658	0.0000
ENSG00000108828	vat1	ENSDARG00000056481	0.9611	0.0000	1.8175	0.0000
ENSG00000275183	leng9	ENSDARG00000062374	0.9611	0.0000	2.4706	0.0000
ENSG00000164342	tlr3	ENSDARG00000016065	0.9601	0.0000	3.3232	0.0000
ENSG00000136942	rpl35	ENSDARG00000018334	0.9599	0.0000	2.3909	0.0000
ENSG00000137154	rps6	ENSDARG00000019778	0.9597	0.0000	2.5930	0.0000
ENSG00000161202	dvl3a	ENSDARG00000015707	0.9595	0.0000	1.8172	0.0000
ENSG00000170017	alcamb	ENSDARG00000058538	0.9592	0.0000	3.9561	0.0000
ENSG00000152939	marveld2a	ENSDARG00000025076	0.9578	0.0001	1.8153	0.0000
ENSG00000249115	haus5	ENSDARG00000019156	0.9564	0.0000	3.4811	0.0000
ENSG00000145592	rpl37	ENSDARG00000034291	0.9548	0.0000	2.3974	0.0000
ENSG00000124496	TRERF1	ENSDARG00000088631	0.9542	0.0000	1.6147	0.0000
ENSG00000160213	cst14b.1	ENSDARG00000045980	0.9520	0.0000	3.3055	0.0000
ENSG00000204389	si:ch211-199o1.2	ENSDARG00000056210	0.9515	0.0000	1.8226	0.0000
ENSG00000213977	tax1bp3	ENSDARG00000059177	0.9503	0.0000	2.2301	0.0000
ENSG00000168672	FAM84B	ENSDARG00000040280	0.9493	0.0000	1.7651	0.0000
ENSG00000240583	aqp1a.1	ENSDARG00000023713	0.9454	0.0000	-1.6935	0.0000
ENSG00000164932	cthrc1b	ENSDARG00000001971	0.9449	0.0000	1.9279	0.0000
ENSG00000122025	flt3	ENSDARG00000058503	0.9449	0.0000	1.8924	0.0000
ENSG00000148935	GAS2	ENSDARG00000001259	0.9447	0.0000	2.6188	0.0000
ENSG00000101224	cdc25b	ENSDARG00000010792	0.9403	0.0000	2.8185	0.0000
ENSG00000188783	prelp	ENSDARG00000070597	0.9386	0.0895	3.5850	0.0000
ENSG00000143971	etaa1	ENSDARG00000074915	0.9368	0.0000	3.0230	0.0000
ENSG00000197405	c5ar1	ENSDARG00000040319	0.9322	0.0000	2.3160	0.0000
ENSG00000090889	kif4	ENSDARG00000005462	0.9305	0.0000	3.1146	0.0000
ENSG00000198719	dla	ENSDARG00000010791	0.9292	0.0000	2.8463	0.0000
ENSG00000198892	SHISA4	ENSDARG00000013681	0.9263	0.0000	2.5716	0.0000
ENSG00000149328	si:dkey-224e22.2	ENSDARG00000034941	0.9244	0.0003	2.5197	0.0000
ENSG00000142156	col6a1	ENSDARG00000074908	0.9239	0.0000	2.1693	0.0000
ENSG00000196476	C11H20orf96	ENSDARG00000094037	0.9234	0.0060	1.6966	0.0000
ENSG00000122406	rpl5a	ENSDARG00000020197	0.9220	0.0000	2.3317	0.0000
ENSG00000089009	rpl6	ENSDARG00000058451	0.9202	0.0000	2.4038	0.0000

ENSG00000160818	gpatch4	ENSDARG00000091931	0.9170	0.0000	1.8484	0.0000
ENSG00000181163	npm1a	ENSDARG00000014329	0.9128	0.0000	2.1470	0.0000
ENSG00000161036	lrwd1	ENSDARG00000035147	0.9106	0.0000	2.0730	0.0000
ENSG00000128602	smo	ENSDARG00000002952	0.9074	0.0000	1.7106	0.0000
ENSG00000142676	rpl11	ENSDARG00000043509	0.9069	0.0000	2.5222	0.0000
ENSG00000119729	rhoq	ENSDARG00000025953	0.9069	0.0014	1.8074	0.0000
ENSG00000134548	spx	ENSDARG00000056859	0.9040	0.0000	2.2364	0.0000
ENSG00000167552	tuba1a	ENSDARG00000001889	0.9007	0.0000	3.2077	0.0000
ENSG00000129910	cdh15	ENSDARG00000068191	0.8999	0.0000	1.5957	0.0000
ENSG00000114942	eef1b2	ENSDARG00000044521	0.8979	0.0000	2.4396	0.0000
ENSG00000111799	col12a1a	ENSDARG00000078322	0.8971	0.0000	2.2241	0.0000
ENSG00000069122	BX545917.1	ENSDARG00000040194	0.8960	0.0000	2.0125	0.0000
ENSG00000159055	mis18a	ENSDARG00000035333	0.8955	0.0000	1.8829	0.0000
ENSG00000174669	slc29a2	ENSDARG00000001767	0.8938	0.0000	3.2195	0.0000
ENSG00000069812	hes2.1	ENSDARG00000074897	0.8931	0.0273	3.7370	0.0000
ENSG00000165633	vstm4a	ENSDARG00000010154	0.8931	0.0005	1.8282	0.0000
ENSG00000158321	auts2b	ENSDARG00000020946	0.8899	0.0000	-1.6078	0.0000
ENSG00000134440	nars	ENSDARG00000061100	0.8890	0.0000	1.9185	0.0000
ENSG00000163754	gyg1b	ENSDARG00000059391	0.8889	0.0000	-1.8717	0.0000
ENSG00000180448	HMHA1	ENSDARG00000052496	0.8885	0.0000	1.7004	0.0000
ENSG00000205129	C14H4orf47	ENSDARG00000037142	0.8875	0.0019	2.4725	0.0000
ENSG00000131096	pyyb	ENSDARG00000035832	0.8845	0.0989	4.5569	0.0000
ENSG00000154217	PITPNC1	ENSDARG00000022807	0.8845	0.0989	2.1069	0.0000
ENSG00000198755	rpl10a	ENSDARG00000042905	0.8827	0.0000	2.0259	0.0000
ENSG00000196843	arid5a	ENSDARG00000077120	0.8803	0.0000	1.6843	0.0000
ENSG00000169242	efna1b	ENSDARG00000018787	0.8800	0.0000	-2.2450	0.0000
ENSG00000077150	nfkb2	ENSDARG00000038687	0.8794	0.0000	1.8009	0.0000
ENSG00000196230	tubb5	ENSDARG00000037997	0.8745	0.0000	5.0648	0.0000
ENSG00000102024	pls3	ENSDARG00000037655	0.8724	0.0000	2.3413	0.0000
ENSG00000123473	stil	ENSDARG00000024904	0.8713	0.0000	2.6923	0.0000
ENSG00000112297	aim1a	ENSDARG00000031782	0.8712	0.0000	2.4232	0.0000
ENSG00000067191	cacnb1	ENSDARG00000002167	0.8703	0.0000	2.0000	0.0000
ENSG00000163827	LRRC2	ENSDARG00000087624	0.8702	0.0000	-1.8573	0.0000
ENSG00000277443	marcksa	ENSDARG00000004049	0.8699	0.0002	3.3392	0.0000
ENSG00000233927	rps28	ENSDARG00000035860	0.8697	0.0000	2.3448	0.0000
ENSG00000067167	tram1	ENSDARG00000019137	0.8689	0.0000	1.6270	0.0000
ENSG00000101670	lipg	ENSDARG00000031044	0.8674	0.0000	1.6461	0.0000
ENSG00000118193	kif14	ENSDARG00000062187	0.8643	0.0000	3.0018	0.0000
ENSG00000145425	rps3a	ENSDARG00000035692	0.8637	0.0000	2.2083	0.0000
ENSG00000134330	iah1	ENSDARG00000015765	0.8633	0.0000	2.1852	0.0000
ENSG00000163521	glb1l	ENSDARG00000029955	0.8629	0.0000	2.1845	0.0000
ENSG00000130956	HABP4	ENSDARG00000025174	0.8625	0.0300	2.6174	0.0000
ENSG00000161016	rpl8	ENSDARG00000014867	0.8619	0.0000	2.1914	0.0000
ENSG00000105640	rpl18a	ENSDARG00000025073	0.8614	0.0000	2.1660	0.0000
ENSG00000149781	FERMT3	ENSDARG00000079267	0.8614	0.0000	1.7103	0.0000
ENSG00000113389	npr3	ENSDARG00000035253	0.8601	0.0000	1.6270	0.0000
ENSG00000115268	rps15	ENSDARG00000070849	0.8564	0.0000	2.2304	0.0000
ENSG00000135842	fam129ab	ENSDARG00000023053	0.8560	0.0000	2.5135	0.0000
ENSG00000153044	cenph	ENSDARG00000094892	0.8556	0.0022	3.0794	0.0000
ENSG00000240303	acad11	ENSDARG00000045411	0.8548	0.0000	1.6742	0.0000
ENSG00000113296	thbs4b	ENSDARG00000020072	0.8538	0.0000	2.2084	0.0000
ENSG00000197728	rps26l	ENSDARG00000030408	0.8535	0.0000	2.1867	0.0000
ENSG00000111361	eif2b1	ENSDARG00000091402	0.8535	0.0000	1.5870	0.0000
ENSG00000188522	FAM83G	ENSDARG00000090340	0.8515	0.0014	1.6761	0.0000
ENSG00000102181	CD99L2	ENSDARG00000087508	0.8513	0.0000	2.7741	0.0000
ENSG00000176974	shmt1	ENSDARG00000052816	0.8508	0.0000	1.6031	0.0000
ENSG00000101916	tlr8b	ENSDARG00000073675	0.8480	0.1849	2.3785	0.0000
ENSG00000143947	rps27a	ENSDARG00000032725	0.8479	0.0000	2.0792	0.0000

ENSG00000169136	atf5a	ENSDARG00000068096	0.8473	0.0000	2.4575	0.0000
ENSG00000100138	snu13b	ENSDARG00000023299	0.8470	0.0000	2.0216	0.0000
ENSG00000197712	fam114a1	ENSDARG00000008287	0.8468	0.0000	1.8716	0.0000
ENSG00000170889	rps9	ENSDARG00000011405	0.8459	0.0000	2.1462	0.0000
ENSG00000132906	casp9	ENSDARG00000004325	0.8453	0.0000	1.7653	0.0000
ENSG00000164587	rps14	ENSDARG00000036629	0.8439	0.0000	2.1487	0.0000
ENSG00000101384	jag1a	ENSDARG00000030289	0.8433	0.0000	2.9182	0.0000
ENSG00000137699	TRIM47	ENSDARG00000067661	0.8425	0.0140	4.3515	0.0000
ENSG00000168878	sftpbb	ENSDARG00000067566	0.8419	0.0000	1.6862	0.0000
ENSG00000146232	nfkbie	ENSDARG00000068367	0.8411	0.0000	1.8488	0.0000
ENSG00000178695	kctd12.2	ENSDARG00000053542	0.8399	0.0000	2.2873	0.0000
ENSG00000147604	rpl7	ENSDARG00000007320	0.8397	0.0000	2.1439	0.0000
ENSG00000164111	anxa5a	ENSDARG00000026406	0.8395	0.0000	3.9257	0.0000
ENSG00000154920	eme1	ENSDARG00000076913	0.8395	0.0534	3.4386	0.0000
ENSG00000067066	si:dkey-68o6.2	ENSDARG00000055359	0.8389	0.0001	1.9949	0.0000
ENSG00000148303	rpl7a	ENSDARG00000019230	0.8379	0.0000	2.2971	0.0000
ENSG00000124614	rps10	ENSDARG00000034897	0.8330	0.0000	2.3565	0.0000
ENSG00000140451	pif1	ENSDARG00000020289	0.8329	0.0002	2.9858	0.0000
ENSG00000164291	arsk	ENSDARG00000059714	0.8327	0.0000	1.8303	0.0000
ENSG00000134419	rps15a	ENSDARG00000010160	0.8318	0.0000	2.1138	0.0000
ENSG00000163918	rfc4	ENSDARG00000042458	0.8282	0.0000	2.1975	0.0000
ENSG00000077943	itga8	ENSDARG00000078717	0.8274	0.0000	2.3747	0.0000
ENSG00000149636	DSN1	ENSDARG00000089922	0.8274	0.0000	2.2224	0.0000
ENSG00000134321	rsad2	ENSDARG00000004952	0.8274	0.0000	2.2255	0.0000
ENSG00000071082	rpl31	ENSDARG00000053365	0.8269	0.0000	2.3771	0.0000
ENSG00000185681	morn5	ENSDARG00000033610	0.8260	0.0396	1.9835	0.0000
ENSG00000198075	sult1st5	ENSDARG00000003475	0.8249	0.0000	2.7256	0.0000
ENSG00000117697	nsl1	ENSDARG00000024471	0.8231	0.0050	2.9044	0.0000
ENSG00000109736	mfsd10	ENSDARG00000086786	0.8206	0.0000	1.6174	0.0000
ENSG00000243480	zgc:66313	ENSDARG00000030357	0.8195	0.0000	1.7526	0.0000
ENSG00000254772	eef1g	ENSDARG00000056119	0.8174	0.0000	2.5181	0.0000
ENSG00000106100	nod1	ENSDARG00000036308	0.8168	0.0000	1.6376	0.0000
ENSG00000196305	iars	ENSDARG00000007955	0.8160	0.0000	1.5804	0.0000
ENSG00000123131	prdx4	ENSDARG00000069013	0.8139	0.0000	1.8303	0.0000
ENSG00000137275	ripk1l	ENSDARG00000006677	0.8137	0.0000	1.6376	0.0000
ENSG00000133106	epsti1	ENSDARG00000094578	0.8130	0.0000	2.2667	0.0000
ENSG00000134001	eif2s1b	ENSDARG00000052178	0.8123	0.0000	2.0100	0.0000
ENSG00000103335	piezo1	ENSDARG00000076870	0.8105	0.0000	1.8053	0.0000
ENSG00000132170	pparg	ENSDARG00000031848	0.8100	0.0000	1.6560	0.0000
ENSG00000213741	rps29	ENSDARG00000041232	0.8076	0.0000	2.0027	0.0000
ENSG00000180209	mylpfb	ENSDARG00000002589	0.8074	0.0961	2.6865	0.0000
ENSG00000129353	slc44a2	ENSDARG00000037059	0.8074	0.0000	2.5216	0.0000
ENSG00000137699	TRIM47	ENSDARG00000069514	0.8074	0.0035	2.3671	0.0000
ENSG00000119969	hells	ENSDARG00000057738	0.8030	0.0000	2.2364	0.0000
ENSG00000163682	rpl9	ENSDARG00000037350	0.8027	0.0000	2.0683	0.0000
ENSG00000133313	cndp2	ENSDARG00000003931	0.8025	0.0000	2.0984	0.0000
ENSG00000189127	ankrd34bb	ENSDARG00000061641	0.8017	0.0000	3.1581	0.0000
ENSG00000171863	rps7	ENSDARG00000042566	0.8011	0.0000	2.2303	0.0000
ENSG00000138385	ssb	ENSDARG00000029252	0.8010	0.0000	1.7652	0.0000
ENSG00000063177	rpl18	ENSDARG00000029533	0.7997	0.0000	2.2789	0.0000
ENSG00000108107	rpl28	ENSDARG00000005791	0.7953	0.0000	2.2543	0.0000
ENSG00000065308	tram2	ENSDARG00000030092	0.7931	0.0000	1.6076	0.0000
ENSG00000186468	rps23	ENSDARG00000021838	0.7918	0.0000	2.0500	0.0000
ENSG00000156482	rpl30	ENSDARG00000035871	0.7914	0.0000	1.8291	0.0000
ENSG00000145912	nhp2	ENSDARG00000069422	0.7899	0.0000	1.9614	0.0000
ENSG00000137699	ftr13	ENSDARG00000079537	0.7885	0.0519	2.3735	0.0000
ENSG00000151778	serp2	ENSDARG00000094003	0.7865	0.0000	1.5857	0.0000
ENSG00000164889	slc4a2a	ENSDARG00000028173	0.7859	0.0238	2.0000	0.0000

ENSG00000213512	gbp3	ENSDARG00000003244	0.7850	0.0000	1.9851	0.0000
ENSG00000198918	rpl39	ENSDARG00000036316	0.7845	0.0000	2.1369	0.0000
ENSG00000182774	RPS17	ENSDARG00000046157	0.7845	0.0000	2.3592	0.0000
ENSG00000145703	iqgap2	ENSDARG00000060010	0.7838	0.0000	2.0004	0.0000
ENSG00000155363	mov10b.2	ENSDARG00000056065	0.7832	0.0001	2.7561	0.0000
ENSG00000093009	cdc45	ENSDARG00000043720	0.7814	0.0000	2.1059	0.0000
ENSG00000197894	adh5	ENSDARG00000080010	0.7812	0.0000	2.2809	0.0000
ENSG00000151849	cenpj	ENSDARG00000018121	0.7795	0.0000	2.3798	0.0000
ENSG00000163482	stk36	ENSDARG00000061095	0.7790	0.0000	1.7690	0.0000
ENSG00000213920	MDP1	ENSDARG00000088040	0.7790	0.0000	2.4559	0.0000
ENSG00000134716	cyp2ae1	ENSDARG00000013524	0.7789	0.0000	1.8564	0.0000
ENSG00000125826	rbck1	ENSDARG00000031954	0.7776	0.0286	1.7318	0.0000
ENSG00000128606	lrrc17	ENSDARG00000037960	0.7771	0.0000	1.9104	0.0000
ENSG00000185475	TMEM179B	ENSDARG00000056233	0.7752	0.0000	2.4355	0.0000
ENSG00000105193	rps16	ENSDARG00000045487	0.7738	0.0000	2.2368	0.0000
ENSG00000159167	stc1	ENSDARG00000003303	0.7729	0.0000	3.3022	0.0000
ENSG00000188001	tprg1	ENSDARG00000056026	0.7726	0.0003	1.8301	0.0000
ENSG00000129484	parp2	ENSDARG00000079202	0.7726	0.0000	1.5817	0.0000
ENSG00000083845	rps5	ENSDARG00000043453	0.7706	0.0000	2.2146	0.0000
ENSG00000184640	sept9a	ENSDARG00000020235	0.7705	0.1038	2.4517	0.0000
ENSG00000103187	cotl1	ENSDARG00000026829	0.7703	0.0000	1.7582	0.0000
ENSG00000213889	ppm1na	ENSDARG00000010231	0.7695	0.0000	-4.0076	0.0000
ENSG00000163959	slc51a	ENSDARG00000045306	0.7675	0.0000	1.8089	0.0000
ENSG00000128989	arpp19b	ENSDARG00000039880	0.7673	0.0000	2.2756	0.0000
ENSG00000189058	apodb	ENSDARG00000057437	0.7673	0.0043	1.8548	0.0000
ENSG00000148773	mki67	ENSDARG00000091150	0.7658	0.0000	3.3968	0.0000
ENSG00000138092	cenpo	ENSDARG00000075619	0.7639	0.0025	2.0534	0.0000
ENSG00000176720	boka	ENSDARG00000052129	0.7638	0.0000	-1.9421	0.0000
ENSG00000162461	SLC25A34	ENSDARG00000087401	0.7627	0.0000	1.8107	0.0000
ENSG00000268104	slc6a14	ENSDARG00000029866	0.7590	0.1755	1.7914	0.0000
ENSG00000128590	dnajb9b	ENSDARG00000016886	0.7529	0.0027	2.1031	0.0000
ENSG00000110700	rps13	ENSDARG00000036298	0.7518	0.0000	2.1877	0.0000
ENSG00000198242	rpl23a	ENSDARG00000006316	0.7517	0.0000	2.1471	0.0000
ENSG00000113328	ccng1	ENSDARG00000076667	0.7495	0.0000	2.3359	0.0000
ENSG00000138363	atic	ENSDARG00000016706	0.7492	0.0000	1.9379	0.0000
ENSG00000144713	rpl32	ENSDARG00000054818	0.7490	0.0000	2.0884	0.0000
ENSG00000141522	arhgdia	ENSDARG00000043795	0.7463	0.0000	1.9324	0.0000
ENSG00000197958	rpl12	ENSDARG00000006691	0.7457	0.0000	2.3738	0.0000
ENSG00000173801	jupa	ENSDARG00000070787	0.7448	0.0000	2.3313	0.0000
ENSG00000196562	sulf2a	ENSDARG00000018423	0.7428	0.0050	3.4187	0.0000
ENSG00000162849	kif26bb	ENSDARG00000024575	0.7420	0.0000	2.4444	0.0000
ENSG00000142541	rpl13a	ENSDARG00000044093	0.7411	0.0000	1.8974	0.0000
ENSG00000107937	gtpbp4	ENSDARG00000018961	0.7402	0.0000	1.8074	0.0000
ENSG00000129951	lppr3a	ENSDARG00000010144	0.7396	0.0000	2.8669	0.0000
ENSG00000065978	ybx1	ENSDARG00000004757	0.7385	0.0000	1.9201	0.0000
ENSG00000166426	crabp1a	ENSDARG00000045926	0.7377	0.0000	1.6477	0.0000
ENSG00000112761	wisp3	ENSDARG00000071048	0.7370	0.0011	2.4313	0.0000
ENSG00000171490	rsl1d1	ENSDARG00000055868	0.7349	0.0000	1.6111	0.0000
ENSG00000184307	zdhhc23b	ENSDARG00000003899	0.7327	0.0010	1.6674	0.0000
ENSG00000106105	gars	ENSDARG00000059070	0.7320	0.0000	1.8056	0.0000
ENSG00000051180	rad51	ENSDARG00000041411	0.7313	0.0000	2.0124	0.0000
ENSG00000137996	rtca	ENSDARG00000002215	0.7310	0.0000	1.6898	0.0000
ENSG00000166228	pcbd1	ENSDARG00000031981	0.7288	0.0000	2.0888	0.0000
ENSG00000198034	rps4x	ENSDARG00000014690	0.7245	0.0000	2.2243	0.0000
ENSG00000105372	rps19	ENSDARG00000030602	0.7233	0.0000	2.1692	0.0000
ENSG00000101447	fam83d	ENSDARG00000077883	0.7225	0.0984	3.2016	0.0000
ENSG00000180616	SSTR2	ENSDARG00000069806	0.7208	0.0000	-3.0793	0.0000
ENSG00000142534	RPS11	ENSDARG00000093606	0.7192	0.0000	1.9887	0.0000

ENSG00000159640	ace	ENSDARG00000079166	0.7192	0.0000	2.0315	0.0000
ENSG00000227500	scamp4	ENSDARG00000010531	0.7159	0.0000	2.1631	0.0000
ENSG00000115457	igfbp2b	ENSDARG00000031422	0.7154	0.0000	2.3332	0.0000
ENSG00000130948	hsd17b3	ENSDARG00000023287	0.7141	0.0000	-1.8184	0.0000
ENSG00000150093	itgb1b.2	ENSDARG00000022689	0.7141	0.0002	1.7674	0.0000
ENSG00000112701	senp6b	ENSDARG00000079992	0.7134	0.0000	-1.7500	0.0000
ENSG00000147403	rpl10	ENSDARG00000025581	0.7125	0.0000	1.8885	0.0000
ENSG00000118960	hs1bp3	ENSDARG00000034222	0.7105	0.0000	2.0896	0.0000
ENSG00000122574	WIPF3	ENSDARG00000036245	0.7105	0.0314	1.8136	0.0000
ENSG00000166033	htra1a	ENSDARG00000032831	0.7095	0.0000	2.0521	0.0000
ENSG00000127586	chtf18	ENSDARG00000058480	0.7074	0.0006	2.5480	0.0000
ENSG00000088832	fkbp1ab	ENSDARG00000033567	0.7074	0.0000	2.0047	0.0000
ENSG00000136026	CKAP4	ENSDARG00000032405	0.7063	0.0008	1.6529	0.0000
ENSG00000137965	si:dkey-79f11.8	ENSDARG00000095673	0.7004	0.1641	3.4429	0.0000
ENSG00000127129	edn2	ENSDARG00000070578	0.7004	0.1641	2.4094	0.0000
ENSG00000091651	orc6	ENSDARG00000075682	0.6986	0.0000	2.1300	0.0000
ENSG00000145741	btf3	ENSDARG00000035400	0.6967	0.0000	1.6787	0.0000
ENSG00000118263	klf7b	ENSDARG00000043821	0.6966	0.0000	2.3100	0.0000
ENSG00000166451	cenpn	ENSDARG00000043640	0.6941	0.0000	2.1893	0.0000
ENSG00000136279	dbnla	ENSDARG00000008170	0.6919	0.0681	3.2826	0.0000
ENSG00000097046	cdc7	ENSDARG00000023584	0.6909	0.0018	2.5537	0.0000
ENSG00000183508	fam46c	ENSDARG00000010437	0.6866	0.0000	2.2456	0.0000
ENSG00000160307	s100b	ENSDARG00000057598	0.6843	0.0000	2.1009	0.0000
ENSG00000109475	rpl34	ENSDARG00000029500	0.6828	0.0000	2.0778	0.0000
ENSG00000165071	TMEM71	ENSDARG00000079784	0.6827	0.0000	1.7652	0.0000
ENSG00000151690	mfsd6b	ENSDARG00000054583	0.6812	0.0000	2.4426	0.0000
ENSG00000083093	palb2	ENSDARG00000076716	0.6812	0.0055	1.7859	0.0000
ENSG00000141433	adcyap1b	ENSDARG00000027740	0.6786	0.0000	2.7604	0.0000
ENSG00000187068	C9H3orf70	ENSDARG00000061664	0.6782	0.0000	-2.3138	0.0000
ENSG00000142534	rps11	ENSDARG00000053058	0.6781	0.0000	1.8346	0.0000
ENSG00000265681	C21H18orf32	ENSDARG00000057556	0.6755	0.0000	1.9907	0.0000
ENSG00000143320	crabp2a	ENSDARG00000073978	0.6746	0.0096	2.6746	0.0000
ENSG00000051341	polq	ENSDARG00000044622	0.6745	0.0000	1.9489	0.0000
ENSG00000092036	haus4	ENSDARG00000016594	0.6702	0.0001	2.3664	0.0000
ENSG00000108298	rpl19	ENSDARG00000013307	0.6701	0.0000	2.0145	0.0000
ENSG00000205220	PSMB10	ENSDARG00000043781	0.6683	0.0000	1.7024	0.0000
ENSG00000065328	mcm10	ENSDARG00000045815	0.6682	0.0022	2.7050	0.0000
ENSG00000198910	nadl1.1	ENSDARG00000015025	0.6680	0.0000	1.9026	0.0000
ENSG00000137699	ftr89	ENSDARG00000040536	0.6663	0.0168	1.9603	0.0000
ENSG00000096093	efhc1	ENSDARG00000009743	0.6656	0.0069	2.1699	0.0000
ENSG00000171858	rps21	ENSDARG00000025850	0.6656	0.0000	2.0067	0.0000
ENSG00000112742	ttk	ENSDARG00000041361	0.6630	0.0026	2.8216	0.0000
ENSG00000188229	tubb4b	ENSDARG00000002344	0.6628	0.0000	1.6860	0.0000
ENSG00000153551	cmtm7	ENSDARG00000010032	0.6625	0.0000	1.9151	0.0000
ENSG00000196689	trpv1	ENSDARG00000059883	0.6611	0.0000	2.3549	0.0000
ENSG00000198931	aprt	ENSDARG00000003519	0.6596	0.0000	1.6044	0.0000
ENSG00000177791	myoz1a	ENSDARG00000056209	0.6590	0.1524	1.7744	0.0000
ENSG00000156381	ankrd9	ENSDARG00000028804	0.6585	0.0000	-2.2789	0.0000
ENSG00000100316	rpl3	ENSDARG00000003599	0.6579	0.0000	1.8540	0.0000
ENSG00000137699	ftr50	ENSDARG00000054153	0.6565	0.0072	2.0774	0.0000
ENSG00000118181	rps25	ENSDARG00000041811	0.6556	0.0000	2.1033	0.0000
ENSG00000265203	irbp	ENSDARG00000059163	0.6543	0.0000	-3.2764	0.0000
ENSG00000163362	C11H1orf106	ENSDARG00000070571	0.6533	0.0003	2.0883	0.0000
ENSG00000101213	ptk6a	ENSDARG00000017947	0.6521	0.2430	2.4021	0.0000
ENSG00000214050	fbxo16	ENSDARG00000033949	0.6521	0.2430	1.5850	0.0002
ENSG00000132182	nup210	ENSDARG00000063333	0.6490	0.0000	1.8429	0.0000
ENSG00000197771	mcmbp	ENSDARG00000055314	0.6486	0.0000	1.8206	0.0000
ENSG00000178927	C12H17orf62	ENSDARG00000071414	0.6485	0.0002	2.0097	0.0000

ENSG00000134917	adamts8a	ENSDARG00000007709	0.6485	0.0396	1.6974	0.0000
ENSG00000104332	sfrp1a	ENSDARG00000035521	0.6480	0.0006	3.5650	0.0000
ENSG00000154277	uchl1	ENSDARG00000026871	0.6476	0.0000	3.3217	0.0000
ENSG00000087088	baxa	ENSDARG00000020623	0.6472	0.0000	1.8786	0.0000
ENSG00000134716	cyp2p6	ENSDARG00000042978	0.6471	0.0000	-2.0073	0.0000
ENSG00000197728	rps26	ENSDARG00000037071	0.6400	0.0000	1.6915	0.0000
ENSG00000147642	sybu	ENSDARG00000060112	0.6397	0.0000	-1.6905	0.0000
ENSG00000174444	rpl4	ENSDARG00000041182	0.6370	0.0000	1.8832	0.0000
ENSG00000118242	MREG	ENSDARG00000011076	0.6369	0.0000	-2.2980	0.0000
ENSG00000184144	cntn2	ENSDARG00000000472	0.6337	0.0000	3.3732	0.0000
ENSG00000166959	ms4a17a.11	ENSDARG00000053561	0.6331	0.0000	2.1322	0.0000
ENSG00000168003	SLC3A2	ENSDARG00000055791	0.6325	0.0000	-2.0480	0.0000
ENSG00000172680	mos	ENSDARG00000069744	0.6301	0.0329	2.5907	0.0000
ENSG00000125319	C3H17orf53	ENSDARG00000096229	0.6265	0.0125	1.7521	0.0000
ENSG00000170542	serpinb14	ENSDARG00000091801	0.6243	0.0000	2.0504	0.0000
ENSG00000164733	ctsba	ENSDARG00000055120	0.6231	0.0000	2.1994	0.0000
ENSG00000131470	psmc3ip	ENSDARG00000037892	0.6204	0.0071	2.2826	0.0000
ENSG00000162244	RPL29	ENSDARG00000077717	0.6160	0.0000	1.9486	0.0000
ENSG00000157456	ccnb2	ENSDARG00000036180	0.6138	0.0083	3.3350	0.0000
ENSG00000159337	pla2g4f.1	ENSDARG00000056535	0.6130	0.2221	1.6939	0.0000
ENSG00000163479	ssr2	ENSDARG00000005230	0.6114	0.0000	1.7323	0.0000
ENSG00000167536	dhrs13b	ENSDARG00000031814	0.6105	0.0000	1.6244	0.0000
ENSG00000000003	tspan7	ENSDARG00000015759	0.6099	0.0000	2.3343	0.0000
ENSG00000198765	sycp1	ENSDARG00000003904	0.6077	0.1690	1.6738	0.0000
ENSG00000164649	cdca7b	ENSDARG00000076659	0.6055	0.0000	1.8074	0.0000
ENSG00000138326	rps24	ENSDARG00000039347	0.6051	0.0000	1.8054	0.0000
ENSG00000146839	ZAN	ENSDARG00000056781	0.6037	0.0000	-2.5127	0.0000
ENSG00000092841	myl6	ENSDARG00000008494	0.5989	0.0000	2.2949	0.0000
ENSG00000101444	ahcy	ENSDARG00000005191	0.5980	0.0000	2.2716	0.0000
ENSG00000171320	esco2	ENSDARG00000014685	0.5967	0.0000	3.1986	0.0000
ENSG00000106305	aimp2	ENSDARG00000018903	0.5955	0.0000	1.7980	0.0000
ENSG00000164849	gpr146	ENSDARG00000059610	0.5953	0.0000	-1.8373	0.0000
ENSG00000167969	eci1	ENSDARG00000018002	0.5951	0.0000	1.7959	0.0000
ENSG00000104805	nucb1	ENSDARG00000054833	0.5941	0.0000	1.6723	0.0000
ENSG00000197614	mfap5	ENSDARG00000090560	0.5940	0.0000	-2.9044	0.0000
ENSG00000187037	GPR141	ENSDARG00000077026	0.5935	0.0008	-1.8074	0.0000
ENSG00000070756	pabpc1a	ENSDARG00000017219	0.5922	0.0000	1.5834	0.0000
ENSG00000156136	dck	ENSDARG00000044807	0.5886	0.0000	1.7408	0.0000
ENSG00000174607	ugt8	ENSDARG00000037455	0.5879	0.0001	2.0022	0.0000
ENSG00000142731	plk4	ENSDARG00000004576	0.5871	0.0000	2.4931	0.0000
ENSG00000121064	scpep1	ENSDARG00000040072	0.5870	0.0000	1.9118	0.0000
ENSG00000122026	rpl21	ENSDARG00000010516	0.5859	0.0000	1.7796	0.0000
ENSG00000132702	hapln2	ENSDARG00000003903	0.5850	0.0041	2.6796	0.0000
ENSG00000187098	mitfa	ENSDARG00000003732	0.5850	0.1053	1.9260	0.0000
ENSG00000231925	tapbp	ENSDARG00000079402	0.5842	0.0000	1.6405	0.0000
ENSG00000115718	proca	ENSDARG00000038258	0.5835	0.0000	-2.0044	0.0000
ENSG00000143761	arf1	ENSDARG00000009484	0.5816	0.0000	1.7906	0.0000
ENSG00000277443	marcksb	ENSDARG00000008803	0.5795	0.0000	3.3253	0.0000
ENSG00000241697	tmeff1b	ENSDARG00000056740	0.5732	0.0000	2.1557	0.0000
ENSG00000021488	slc7a9	ENSDARG00000005894	0.5674	0.0000	-1.7814	0.0000
ENSG00000131469	rpl27	ENSDARG00000015128	0.5669	0.0000	1.8300	0.0000
ENSG00000104763	asah1b	ENSDARG00000034817	0.5650	0.0000	2.0296	0.0000
ENSG00000134716	cyp2ad6	ENSDARG00000042956	0.5645	0.0000	-2.8733	0.0000
ENSG00000129757	cdkn1ca	ENSDARG00000010878	0.5639	0.0487	1.9522	0.0000
ENSG00000114346	ect2	ENSDARG00000007278	0.5630	0.0000	2.3710	0.0000
ENSG00000164082	GRM2	ENSDARG00000007195	0.5615	0.0000	-1.7041	0.0000
ENSG00000099992	tbc1d10ab	ENSDARG00000060096	0.5608	0.0000	-2.3309	0.0000
ENSG00000104490	ncalda	ENSDARG00000070688	0.5597	0.0001	1.5877	0.0000

ENSG00000179774	atoh7	ENSDARG00000069552	0.5589	0.0000	2.8366	0.0000
ENSG00000120500	arr3a	ENSDARG00000056511	0.5589	0.0000	-2.5729	0.0000
ENSG00000113396	slc27a6	ENSDARG00000046053	0.5585	0.0000	-2.2455	0.0000
ENSG00000022267	fhl1a	ENSDARG00000071498	0.5564	0.0400	2.9349	0.0000
ENSG00000134716	cyp2p7	ENSDARG00000042980	0.5512	0.0000	-1.9801	0.0000
ENSG00000188976	noc2l	ENSDARG00000001754	0.5498	0.0000	1.5931	0.0000
ENSG00000106266	snx8b	ENSDARG00000078057	0.5491	0.0000	-1.8422	0.0000
ENSG00000148602	lrit1a	ENSDARG00000019179	0.5488	0.0000	-2.9654	0.0000
ENSG00000185633	NDUFA4L2	ENSDARG00000087907	0.5479	0.0000	-1.7641	0.0000
ENSG00000057019	dcbld2	ENSDARG00000062177	0.5463	0.0000	1.8429	0.0000
ENSG00000068745	ip6k2a	ENSDARG00000008310	0.5456	0.0000	-1.9048	0.0000
ENSG00000180881	caps2	ENSDARG00000058486	0.5454	0.0142	2.0242	0.0000
ENSG00000179862	cited4a	ENSDARG00000035990	0.5434	0.0000	-1.8664	0.0000
ENSG00000102886	gdpd3a	ENSDARG00000074466	0.5431	0.0178	1.7534	0.0000
ENSG00000091986	ccdc80l1	ENSDARG00000030975	0.5417	0.0000	-2.2229	0.0000
ENSG00000138346	dna2	ENSDARG00000078759	0.5399	0.0007	2.2047	0.0000
ENSG00000225526	MKRN2OS	ENSDARG00000086655	0.5390	0.0000	2.1026	0.0000
ENSG00000204580	ddr1	ENSDARG00000078523	0.5314	0.0000	2.3343	0.0000
ENSG00000115461	igfbp5b	ENSDARG00000025348	0.5310	0.0000	1.9229	0.0000
ENSG00000181585	tmie	ENSDARG00000069423	0.5308	0.0000	2.0614	0.0000
ENSG00000109943	CRTAM	ENSDARG00000089327	0.5305	0.2912	2.6881	0.0000
ENSG00000278023	RDM1	ENSDARG00000038186	0.5305	0.1753	2.2563	0.0000
ENSG00000135083	ccnjl	ENSDARG00000077116	0.5305	0.0003	1.8914	0.0000
ENSG00000145386	ccna2	ENSDARG00000011094	0.5279	0.0016	3.1103	0.0000
ENSG00000180353	hcls1	ENSDARG00000012729	0.5265	0.0000	1.6219	0.0000
ENSG00000163584	rpl22l1	ENSDARG00000010244	0.5256	0.0000	1.8665	0.0000
ENSG00000139687	rb1	ENSDARG00000006782	0.5239	0.0000	1.6323	0.0000
ENSG00000137942	fnbp1l	ENSDARG00000020131	0.5235	0.0000	1.5938	0.0000
ENSG00000142494	SLC47A1	ENSDARG00000079340	0.5224	0.0000	-3.1260	0.0000
ENSG00000104147	oip5	ENSDARG00000093597	0.5220	0.1002	3.3108	0.0000
ENSG00000140519	rhcgl1	ENSDARG00000007080	0.5208	0.0721	2.0764	0.0000
ENSG00000130741	eif2s3	ENSDARG00000008292	0.5207	0.0000	1.6212	0.0000
ENSG00000179133	C2H10orf67	ENSDARG00000076896	0.5205	0.0009	-2.4795	0.0000
ENSG00000112312	gmnn	ENSDARG00000035957	0.5198	0.0136	1.6080	0.0000
ENSG00000067082	klf6a	ENSDARG00000029072	0.5190	0.0000	1.7754	0.0000
ENSG00000118513	myb	ENSDARG00000053666	0.5185	0.0000	2.8254	0.0000
ENSG00000135387	caprin1b	ENSDARG00000054272	0.5182	0.0000	1.9529	0.0000
ENSG00000244588	rad21l1	ENSDARG00000022493	0.5165	0.0000	-1.9429	0.0000
ENSG00000116251	rpl22	ENSDARG00000070437	0.5143	0.0000	1.6865	0.0000
ENSG00000278685	IQCA1L	ENSDARG00000069230	0.5136	0.0008	-2.0385	0.0000
ENSG00000165244	znf367	ENSDARG00000021086	0.5125	0.0290	2.2026	0.0000
ENSG00000154359	lonrf1l	ENSDARG00000078567	0.5093	0.0000	-1.7844	0.0000
ENSG00000146143	prim2	ENSDARG00000052721	0.5042	0.0092	2.2740	0.0000
ENSG00000160949	tonsl	ENSDARG00000071294	0.5020	0.0003	1.8173	0.0000
ENSG00000107562	cxcl12b	ENSDARG00000055100	0.5004	0.0000	-2.9527	0.0000
ENSG00000148288	gbgt1l4	ENSDARG00000068503	0.4975	0.3489	1.6674	0.0000
ENSG00000163630	synpr	ENSDARG00000044278	0.4948	0.2717	1.6067	0.0000
ENSG00000101197	birc7	ENSDARG00000058082	0.4887	0.0045	1.8402	0.0000
ENSG00000175197	ddit3	ENSDARG00000059836	0.4871	0.0000	2.1468	0.0000
ENSG00000178623	GPR35	ENSDARG00000086776	0.4854	0.5413	1.8074	0.0002
ENSG00000164236	ankrd33bb	ENSDARG00000015589	0.4852	0.0000	-3.0147	0.0000
ENSG00000120686	ufm1	ENSDARG00000043857	0.4846	0.0000	1.7121	0.0000
ENSG00000163577	eif5a2	ENSDARG00000056186	0.4838	0.0000	1.7655	0.0000
ENSG00000196091	mybpc1	ENSDARG00000045560	0.4829	0.0231	1.6694	0.0000
ENSG00000180758	gpr157	ENSDARG00000034975	0.4822	0.0504	2.1208	0.0000
ENSG00000096384	hsp90ab1	ENSDARG00000029150	0.4806	0.0000	1.9579	0.0000
ENSG00000133112	tpt1	ENSDARG00000092693	0.4800	0.0000	1.6072	0.0000
ENSG00000115484	cct4	ENSDARG00000013475	0.4800	0.0000	1.9355	0.0000

ENSG00000174721	FGFBP3	ENSDARG00000040162	0.4783	0.0026	4.1672	0.0000
ENSG00000173894	cbx2	ENSDARG00000044938	0.4764	0.0000	1.6207	0.0000
ENSG00000188747	noxa1	ENSDARG00000056047	0.4695	0.4731	2.2996	0.0000
ENSG00000189171	s100a11	ENSDARG00000093628	0.4678	0.1078	-1.6477	0.0001
ENSG00000151014	nocta	ENSDARG00000077726	0.4677	0.0000	-1.9474	0.0000
ENSG00000133019	fmn2a	ENSDARG00000023318	0.4667	0.0000	-2.3844	0.0000
ENSG00000125835	snrpb	ENSDARG00000011125	0.4665	0.0000	1.6682	0.0000
ENSG00000132718	syt11b	ENSDARG00000056105	0.4655	0.0000	2.8142	0.0000
ENSG00000154553	pdlim3b	ENSDARG00000014248	0.4639	0.0002	2.2121	0.0000
ENSG00000163060	tekt4	ENSDARG00000028899	0.4616	0.0673	1.7835	0.0000
ENSG00000165732	ddx21	ENSDARG00000063626	0.4604	0.0000	1.5966	0.0000
ENSG00000198554	wdhd1	ENSDARG00000015998	0.4594	0.2892	2.2224	0.0000
ENSG00000156052	gnaq	ENSDARG00000011487	0.4582	0.0142	1.9485	0.0000
ENSG00000197208	slc22a4	ENSDARG00000005335	0.4564	0.0071	-2.0112	0.0000
ENSG00000147082	ccnb3	ENSDARG00000034855	0.4561	0.0003	1.9578	0.0000
ENSG00000198844	ARHGEF15	ENSDARG00000061844	0.4547	0.0000	1.6402	0.0000
ENSG00000134571	mybpc3	ENSDARG00000011615	0.4546	0.0000	-1.6376	0.0000
ENSG00000106263	eif3ba	ENSDARG00000059654	0.4541	0.0000	1.8677	0.0000
ENSG00000088808	ppp1r13ba	ENSDARG00000004377	0.4524	0.0000	-1.8374	0.0000
ENSG00000165891	e2f7	ENSDARG00000008986	0.4498	0.1548	2.1970	0.0000
ENSG00000136122	bora	ENSDARG00000042688	0.4498	0.0098	2.0290	0.0000
ENSG00000103569	aqp9b	ENSDARG00000053480	0.4498	0.0000	-1.9575	0.0000
ENSG00000104408	eif3ea	ENSDARG00000090697	0.4493	0.0000	1.9425	0.0000
ENSG00000015592	stmn4	ENSDARG00000030106	0.4462	0.0000	3.4168	0.0000
ENSG00000137168	ppil1	ENSDARG00000015422	0.4415	0.0002	1.8877	0.0000
ENSG00000072422	RHOBTB1	ENSDARG00000079468	0.4401	0.0000	-3.4489	0.0000
ENSG00000164305	casp3a	ENSDARG00000017905	0.4392	0.0000	2.5199	0.0000
ENSG00000132872	syt4	ENSDARG00000036505	0.4391	0.0000	1.6637	0.0000
ENSG00000235156	tmem30c	ENSDARG00000009430	0.4361	0.4296	1.9349	0.0000
ENSG00000196954	caspbl	ENSDARG00000094433	0.4361	0.0687	1.8373	0.0000
ENSG00000106399	rpa3	ENSDARG00000002613	0.4353	0.0000	1.5955	0.0000
ENSG00000100823	apex1	ENSDARG00000045843	0.4350	0.0000	1.8688	0.0000
ENSG00000132780	nasp	ENSDARG00000039208	0.4332	0.0000	1.8699	0.0000
ENSG00000188290	her9	ENSDARG00000056438	0.4314	0.0030	3.6567	0.0000
ENSG00000164104	hmgb2b	ENSDARG00000053990	0.4308	0.0000	2.0500	0.0000
ENSG00000145692	bhmt	ENSDARG00000013430	0.4297	0.1354	1.6614	0.0000
ENSG00000150093	itgb1b.1	ENSDARG00000053232	0.4291	0.0000	1.6937	0.0000
ENSG00000179051	rcc2	ENSDARG00000011510	0.4291	0.0000	1.9432	0.0000
ENSG00000134326	cmpk2	ENSDARG00000031359	0.4265	0.0140	3.1928	0.0000
ENSG00000183571	pgpep1l	ENSDARG00000041092	0.4246	0.0026	-2.0875	0.0000
ENSG00000196526	afap1	ENSDARG00000055284	0.4236	0.0000	2.0530	0.0000
ENSG00000093072	cecr1b	ENSDARG00000015623	0.4233	0.0012	1.7423	0.0000
ENSG00000050748	mapk9	ENSDARG00000077364	0.4220	0.0000	2.1868	0.0000
ENSG00000171940	ZNF217	ENSDARG00000088123	0.4211	0.0000	1.5838	0.0000
ENSG00000135423	gls2b	ENSDARG00000002917	0.4211	0.1055	-1.9758	0.0000
ENSG00000131236	cap1	ENSDARG00000016350	0.4210	0.0034	1.7819	0.0000
ENSG00000156510	hkdc1	ENSDARG00000038703	0.4195	0.0001	-1.8141	0.0000
ENSG00000116748	ampd1	ENSDARG00000033832	0.4183	0.0012	1.8768	0.0000
ENSG00000065882	tbc1d1	ENSDARG00000062081	0.4176	0.0003	2.1637	0.0000
ENSG00000148840	pprc1	ENSDARG00000090337	0.4171	0.0000	1.8655	0.0000
ENSG00000149100	eif3m	ENSDARG00000013931	0.4170	0.0000	1.6892	0.0000
ENSG00000134160	trpm1a	ENSDARG00000011259	0.4166	0.0000	-1.8940	0.0000
ENSG00000175505	clcf1	ENSDARG00000076140	0.4150	0.1561	3.6842	0.0000
ENSG00000159251	si:ch73-187m15.4	ENSDARG00000076126	0.4150	0.3916	1.7574	0.0000
ENSG00000138172	calhm2	ENSDARG00000039482	0.4150	0.2543	1.5995	0.0000
ENSG00000111012	CYP27B1	ENSDARG00000045015	0.4150	0.0005	-1.9027	0.0000
ENSG00000007384	rhbdf1b	ENSDARG00000017367	0.4133	0.0000	-2.9289	0.0000
ENSG00000155636	rbm45	ENSDARG00000063731	0.4132	0.0000	-1.6848	0.0000

ENSG00000204262	COL5A2	ENSDARG00000031678	0.4119	0.0011	2.4923	0.0000
ENSG00000180879	ssr4	ENSDARG00000019444	0.4109	0.0000	1.6558	0.0000
ENSG00000130561	saga	ENSDARG00000012610	0.4091	0.0000	-2.5916	0.0000
ENSG00000115718	PROC	ENSDARG00000093079	0.4064	0.0008	-1.8898	0.0000
ENSG00000247596	twf2b	ENSDARG00000009727	0.4057	0.0035	2.8969	0.0000
ENSG00000166340	tpp1	ENSDARG00000042793	0.4039	0.0000	1.9081	0.0000
ENSG00000079215	slc1a3b	ENSDARG00000043148	0.4038	0.0083	5.4449	0.0000
ENSG00000100138	snu13a	ENSDARG00000069878	0.4034	0.1130	-1.6323	0.0000
ENSG00000170819	bfsp2	ENSDARG00000011998	0.4021	0.3211	2.1584	0.0000
ENSG00000056558	traf1	ENSDARG00000069482	0.3996	0.1797	1.6352	0.0000
ENSG00000136048	dram1	ENSDARG00000045561	0.3991	0.0010	2.0591	0.0000
ENSG00000198624	CCDC69	ENSDARG00000077166	0.3991	0.0000	-1.8962	0.0000
ENSG00000010626	lrrc23	ENSDARG00000006174	0.3959	0.4514	2.2278	0.0000
ENSG00000151651	adam8b	ENSDARG00000057644	0.3954	0.0000	2.3793	0.0000
ENSG00000106299	waslb	ENSDARG00000006283	0.3932	0.0000	-1.7363	0.0000
ENSG00000179163	fuca1.1	ENSDARG00000035890	0.3879	0.0000	2.0899	0.0000
ENSG00000237412	prss56	ENSDARG00000053158	0.3857	0.0000	-3.4085	0.0000
ENSG00000198056	prim1	ENSDARG00000040163	0.3840	0.0000	2.1873	0.0000
ENSG00000213626	lbh	ENSDARG00000087377	0.3831	0.0000	-1.9465	0.0000
ENSG00000069812	hes2.2	ENSDARG00000068168	0.3825	0.1587	2.4827	0.0000
ENSG00000117748	rpa2	ENSDARG00000037188	0.3801	0.0000	1.8065	0.0000
ENSG00000182405	PGBD4	ENSDARG00000079328	0.3720	0.0394	1.7272	0.0000
ENSG00000177685	cracr2b	ENSDARG00000053636	0.3720	0.0005	-2.3354	0.0000
ENSG00000188529	srsf10b	ENSDARG00000086411	0.3718	0.0000	1.6652	0.0000
ENSG00000128165	adm2b	ENSDARG00000078875	0.3704	0.2564	2.5732	0.0000
ENSG00000119185	itgb1bp1	ENSDARG00000007169	0.3700	0.0000	1.8567	0.0000
ENSG00000100097	lgals1l1	ENSDARG00000088711	0.3692	0.4188	2.1565	0.0000
ENSG00000141232	tob1a	ENSDARG00000032619	0.3692	0.0000	-3.0048	0.0000
ENSG00000178585	ctnnbip1	ENSDARG00000038954	0.3677	0.0000	2.3503	0.0000
ENSG00000071539	trip13	ENSDARG00000025043	0.3671	0.0551	1.6806	0.0000
ENSG00000166923	grem1a	ENSDARG00000090720	0.3665	0.0409	-2.0609	0.0000
ENSG00000176155	CCDC57	ENSDARG00000089863	0.3611	0.0571	2.0836	0.0000
ENSG00000054690	plekhh1	ENSDARG00000053835	0.3604	0.0000	1.6963	0.0000
ENSG00000124491	F13A1	ENSDARG00000094832	0.3536	0.2066	2.7489	0.0000
ENSG00000084628	nkain1	ENSDARG00000006859	0.3533	0.0000	2.2515	0.0000
ENSG00000106803	sec61b	ENSDARG00000076568	0.3517	0.0000	1.7201	0.0000
ENSG00000088727	KIF9	ENSDARG00000031693	0.3505	0.0526	2.6630	0.0000
ENSG00000163644	si:ch211-149b19.3	ENSDARG00000076011	0.3502	0.0000	-1.7542	0.0000
ENSG00000143147	GPR161	ENSDARG00000058903	0.3488	0.0076	2.3115	0.0000
ENSG00000111886	gabrr2b	ENSDARG00000014057	0.3479	0.0002	-2.2479	0.0000
ENSG00000143469	syt14a	ENSDARG00000010934	0.3463	0.0000	-2.7614	0.0000
ENSG00000140107	slc25a47a	ENSDARG00000059923	0.3462	0.0000	-1.8703	0.0000
ENSG00000139209	slc38a4	ENSDARG00000018149	0.3460	0.0000	-2.7137	0.0000
ENSG00000157404	kita	ENSDARG00000043317	0.3445	0.0000	-1.6756	0.0000
ENSG00000197057	DTHD1	ENSDARG00000086452	0.3444	0.0000	-2.2792	0.0000
ENSG00000159216	runx1	ENSDARG00000087646	0.3440	0.4350	1.7425	0.0000
ENSG00000115165	CYTIP	ENSDARG00000061717	0.3434	0.0000	-2.0509	0.0000
ENSG00000133110	postna	ENSDARG00000043806	0.3426	0.0149	1.5825	0.0000
ENSG00000138031	ADCY3	ENSDARG00000077145	0.3420	0.0000	-1.6848	0.0000
ENSG00000124159	matn4	ENSDARG00000015947	0.3396	0.0000	-3.1794	0.0000
ENSG00000116584	arhgef2	ENSDARG00000075819	0.3377	0.0000	1.7957	0.0000
ENSG00000150722	ppp1r1c	ENSDARG00000038941	0.3331	0.0000	2.0154	0.0000
ENSG00000112984	kif20a	ENSDARG00000044491	0.3324	0.0049	2.8126	0.0000
ENSG00000049246	per3	ENSDARG00000010519	0.3322	0.0000	-4.4594	0.0000
ENSG00000197177	adgra1a	ENSDARG00000054177	0.3292	0.0000	1.6675	0.0000
ENSG00000155368	dbi	ENSDARG00000026369	0.3238	0.0000	1.6654	0.0000
ENSG00000082126	mpp4a	ENSDARG00000058222	0.3229	0.0000	-2.7811	0.0000
ENSG00000100625	six4b	ENSDARG00000031983	0.3219	0.1815	3.0125	0.0000

ENSG00000165633	vstm4b	ENSDARG00000053186	0.3219	0.2467	2.0630	0.0000
ENSG00000058085	lamc2	ENSDARG00000068288	0.3219	0.3742	1.9069	0.0000
ENSG00000167588	gpd1b	ENSDARG00000043180	0.3217	0.0000	-2.9311	0.0000
ENSG00000171604	cxxc5a	ENSDARG00000078865	0.3203	0.0000	1.7054	0.0000
ENSG00000036672	usp2a	ENSDARG00000020107	0.3190	0.0000	-2.4727	0.0000
ENSG00000143653	SCCPDH	ENSDARG00000075053	0.3186	0.0335	-1.6881	0.0000
ENSG00000197381	adarb1a	ENSDARG00000041609	0.3089	0.0000	-1.8234	0.0000
ENSG00000170145	sik2b	ENSDARG00000062315	0.3083	0.0000	-2.7583	0.0000
ENSG00000118257	nrp2b	ENSDARG00000038446	0.3054	0.0000	2.0091	0.0000
ENSG00000175985	plekhd1	ENSDARG00000091349	0.3048	0.0000	-2.4730	0.0000
ENSG00000048028	usp28	ENSDARG00000008880	0.2983	0.2128	2.7447	0.0000
ENSG00000006534	aldh3b1	ENSDARG00000013839	0.2960	0.1423	1.9116	0.0000
ENSG00000126351	thrab	ENSDARG00000052654	0.2933	0.0000	-2.0533	0.0000
ENSG00000106078	cobl	ENSDARG00000091006	0.2920	0.0000	-1.6295	0.0000
ENSG00000104529	eef1db	ENSDARG00000030053	0.2916	0.0000	1.6063	0.0000
ENSG00000185046	anks1b	ENSDARG00000003512	0.2916	0.0000	-1.5846	0.0000
ENSG00000104537	anxa13l	ENSDARG00000013613	0.2911	0.0000	2.3886	0.0000
ENSG00000081985	il12rb2	ENSDARG00000052157	0.2895	0.6358	3.0969	0.0000
ENSG00000183196	CHST5	ENSDARG00000061357	0.2895	0.0225	-1.6394	0.0000
ENSG00000144852	nr1i2	ENSDARG00000029766	0.2867	0.0000	-1.8450	0.0000
ENSG00000170502	nudt9	ENSDARG00000087927	0.2843	0.0000	1.6185	0.0000
ENSG00000137699	ftr43	ENSDARG00000076106	0.2824	0.4397	2.0097	0.0000
ENSG00000170558	cdh2	ENSDARG00000018693	0.2808	0.0000	1.7586	0.0000
ENSG00000100767	paplnb	ENSDARG00000042186	0.2808	0.0001	-3.7004	0.0000
ENSG00000182177	asb18	ENSDARG00000012948	0.2799	0.0001	-2.3576	0.0000
ENSG00000108370	rgs9b	ENSDARG00000045156	0.2787	0.0000	-1.8519	0.0000
ENSG00000113889	kng1	ENSDARG00000022372	0.2787	0.3017	3.1752	0.0000
ENSG00000173662	tas1r1	ENSDARG00000056739	0.2764	0.0272	-3.1136	0.0000
ENSG00000186867	qrfpra	ENSDARG00000039349	0.2763	0.2018	-2.7914	0.0000
ENSG00000204815	ttc25	ENSDARG00000058140	0.2756	0.6440	2.1444	0.0000
ENSG00000105722	erfl3	ENSDARG00000062801	0.2715	0.0000	-2.3516	0.0000
ENSG00000162191	ubxn1	ENSDARG00000090309	0.2700	0.0026	1.6993	0.0000
ENSG00000272636	doc2b	ENSDARG00000088293	0.2697	0.0000	-1.6176	0.0000
ENSG00000141232	tob1b	ENSDARG00000021372	0.2690	0.0000	-3.3828	0.0000
ENSG00000143367	tuft1b	ENSDARG00000071010	0.2677	0.1842	2.3524	0.0000
ENSG00000154839	ska1	ENSDARG00000039354	0.2668	0.3122	2.4094	0.0000
ENSG00000092964	dpysl2b	ENSDARG00000032083	0.2649	0.0000	2.2184	0.0000
ENSG00000013523	angel1	ENSDARG00000077567	0.2646	0.0001	1.6539	0.0000
ENSG00000068078	fgfr3	ENSDARG00000004782	0.2642	0.0558	-2.2811	0.0000
ENSG00000188038	nrn1la	ENSDARG00000069368	0.2614	0.0011	-1.6265	0.0000
ENSG00000176887	sox11a	ENSDARG00000077811	0.2605	0.0000	2.1306	0.0000
ENSG00000132326	per2	ENSDARG00000034503	0.2565	0.0000	-2.5726	0.0000
ENSG00000138472	guca1c	ENSDARG00000030758	0.2506	0.0000	-7.2269	0.0000
ENSG00000119938	ppp1r3cb	ENSDARG00000014554	0.2504	0.0000	-1.8204	0.0000
ENSG00000162073	paqr4b	ENSDARG00000074160	0.2487	0.0000	-2.1614	0.0000
ENSG00000056972	traf3ip2b	ENSDARG00000089756	0.2479	0.1210	1.7115	0.0000
ENSG00000104722	nefma	ENSDARG00000021351	0.2468	0.0064	1.6682	0.0000
ENSG00000196581	ajap1	ENSDARG00000038655	0.2459	0.0000	-2.5731	0.0000
ENSG00000104419	ndrg1b	ENSDARG00000010420	0.2438	0.0000	-1.8717	0.0000
ENSG00000059915	PSD	ENSDARG00000078102	0.2418	0.0000	-1.6810	0.0000
ENSG00000079101	clul1	ENSDARG00000055595	0.2412	0.0000	-2.4221	0.0000
ENSG00000166598	hsp90b1	ENSDARG00000003570	0.2407	0.0000	1.8701	0.0000
ENSG00000084731	kif3cb	ENSDARG00000079500	0.2393	0.0002	2.5034	0.0000
ENSG00000124507	pacsin1b	ENSDARG00000042128	0.2382	0.0226	1.5810	0.0000
ENSG00000168393	dtymk	ENSDARG00000052103	0.2345	0.0966	1.8745	0.0000
ENSG00000130222	GADD45G	ENSDARG00000044676	0.2345	0.7428	1.8433	0.0000
ENSG00000205639	mfsd2b	ENSDARG00000030263	0.2273	0.0000	-2.4565	0.0000
ENSG00000138798	egf	ENSDARG00000052739	0.2262	0.0000	-2.1449	0.0000

ENSG00000197461	pdgfaa	ENSDARG00000055505	0.2252	0.0171	2.0385	0.0000
ENSG00000146386	abracl	ENSDARG00000042876	0.2248	0.0097	2.0335	0.0000
ENSG00000075618	fscn1a	ENSDARG00000059680	0.2230	0.0000	1.8213	0.0000
ENSG00000107317	ptgdsb	ENSDARG00000027088	0.2216	0.0000	-4.0435	0.0000
ENSG00000171119	nrtn	ENSDARG00000079849	0.2203	0.0147	-2.5999	0.0000
ENSG00000121380	BCL2L14	ENSDARG00000090401	0.2201	0.3185	1.8498	0.0000
ENSG00000134874	dzip1	ENSDARG00000000943	0.2188	0.0000	1.6257	0.0000
ENSG00000133083	dclk1a	ENSDARG00000018856	0.2163	0.0000	-2.0750	0.0000
ENSG00000115183	tanc1a	ENSDARG00000077073	0.2147	0.0000	-2.1542	0.0000
ENSG00000149084	hsd17b12a	ENSDARG00000015709	0.2139	0.0000	-2.6401	0.0000
ENSG00000149091	dgkzb	ENSDARG00000076025	0.2138	0.0000	-1.7931	0.0000
ENSG00000274588	si:dkey-172j4.3	ENSDARG00000074183	0.2126	0.0000	-1.8115	0.0000
ENSG00000111912	NCOA7	ENSDARG00000077559	0.2115	0.7552	2.9119	0.0000
ENSG00000065809	fam107b	ENSDARG00000026865	0.2061	0.0000	-2.1367	0.0000
ENSG00000134107	bhlhe40	ENSDARG00000004060	0.2058	0.0000	-3.4471	0.0000
ENSG00000184979	USP41	ENSDARG00000088078	0.2019	0.1287	1.6075	0.0000
ENSG00000154917	rab6bb	ENSDARG00000031343	0.1995	0.0000	2.0820	0.0000
ENSG00000203910	C6H1orf146	ENSDARG00000075814	0.1966	0.0000	-1.9611	0.0000
ENSG00000026652	agpat4	ENSDARG00000069044	0.1966	0.1077	2.2474	0.0000
ENSG00000167178	islr2	ENSDARG00000051875	0.1931	0.0000	1.8570	0.0000
ENSG00000171885	aqp4	ENSDARG00000010565	0.1916	0.0000	-1.8465	0.0000
ENSG00000214338	soga3a	ENSDARG00000063311	0.1876	0.0000	1.7226	0.0000
ENSG00000156876	sass6	ENSDARG00000058346	0.1860	0.3591	1.6412	0.0000
ENSG00000137699	ftr83	ENSDARG00000025403	0.1856	0.3786	-2.9720	0.0000
ENSG00000164675	iqub	ENSDARG00000061899	0.1852	0.4713	1.7231	0.0000
ENSG00000139793	mbnl2	ENSDARG00000018460	0.1837	0.0000	-1.6359	0.0000
ENSG00000120334	cenpl	ENSDARG00000038802	0.1814	0.4043	1.6289	0.0000
ENSG00000112041	tulp1b	ENSDARG00000078210	0.1800	0.0000	-1.6056	0.0000
ENSG00000253309	serpine3	ENSDARG00000057108	0.1795	0.0000	-2.4283	0.0000
ENSG00000120539	mastl	ENSDARG00000055566	0.1782	0.2050	1.9830	0.0000
ENSG00000134802	slc43a3b	ENSDARG00000057949	0.1758	0.1562	1.7013	0.0000
ENSG00000137699	ftr92	ENSDARG00000095825	0.1750	0.0787	-2.0106	0.0000
ENSG00000149428	hyou1	ENSDARG00000013670	0.1744	0.0000	1.6909	0.0000
ENSG00000143870	pdia6	ENSDARG00000009001	0.1729	0.0000	1.6013	0.0000
ENSG00000180304	oaz2a	ENSDARG00000045929	0.1718	0.0000	-2.7444	0.0000
ENSG00000114349	gnat1	ENSDARG00000044199	0.1668	0.0000	-1.7276	0.0000
ENSG00000148604	rgra	ENSDARG00000054890	0.1646	0.0000	-3.0961	0.0000
ENSG00000205279	CTXN3	ENSDARG00000033508	0.1605	0.7239	2.5564	0.0000
ENSG00000137699	TRIM47	ENSDARG00000093263	0.1575	0.7877	1.9861	0.0000
ENSG00000121274	PAPD5	ENSDARG00000060071	0.1571	0.0000	-2.3668	0.0000
ENSG00000148798	inab	ENSDARG00000053248	0.1495	0.0000	2.2008	0.0000
ENSG00000160957	RECQL4	ENSDARG00000077516	0.1474	0.2583	1.6615	0.0000
ENSG00000184716	SERINC4	ENSDARG00000044815	0.1458	0.0000	-1.6041	0.0000
ENSG00000204928	GRXCR2	ENSDARG00000053692	0.1449	0.4182	-1.9798	0.0000
ENSG00000134339	saa	ENSDARG00000045999	0.1436	0.5522	2.9466	0.0000
ENSG00000116981	nt5c1aa	ENSDARG00000035883	0.1432	0.0000	-2.7086	0.0000
ENSG00000112029	fbxo5	ENSDARG00000039020	0.1430	0.6908	2.4703	0.0000
ENSG00000167780	soat2	ENSDARG00000059824	0.1423	0.1453	-1.9414	0.0000
ENSG00000123612	ACVR1C	ENSDARG00000086172	0.1415	0.0000	-3.1831	0.0000
ENSG00000116337	ampd2b	ENSDARG00000029952	0.1394	0.0000	-2.2404	0.0000
ENSG00000138430	ola1	ENSDARG00000044565	0.1385	0.0000	1.6793	0.0000
ENSG00000116525	TRIM62	ENSDARG00000095536	0.1362	0.5366	-1.7040	0.0000
ENSG00000184702	sept5a	ENSDARG00000013843	0.1352	0.2695	1.6445	0.0000
ENSG00000179580	rnf151	ENSDARG00000075046	0.1342	0.1479	-2.1986	0.0000
ENSG00000087586	aurka	ENSDARG00000012485	0.1333	0.2824	1.9352	0.0000
ENSG00000135540	nhsl1a	ENSDARG00000054537	0.1325	0.0000	-1.9886	0.0000
ENSG00000115221	itgb6	ENSDARG00000002494	0.1315	0.3057	1.5886	0.0000
ENSG00000099998	GGT5	ENSDARG00000078258	0.1309	0.0133	-2.4549	0.0000

ENSG00000166949	smad3a	ENSDARG00000036096	0.1302	0.0002	-1.9584	0.0000
ENSG00000101331	ccm2l	ENSDARG00000063089	0.1233	0.0015	-2.7305	0.0000
ENSG00000091409	itga6a	ENSDARG00000042282	0.1220	0.0831	2.1565	0.0000
ENSG00000082126	MPP4	ENSDARG00000009386	0.1217	0.3243	-2.0623	0.0000
ENSG00000165322	arhgap12b	ENSDARG00000026482	0.1203	0.0000	-1.5946	0.0000
ENSG00000069424	kcnab2b	ENSDARG00000062134	0.1199	0.0000	-1.7131	0.0000
ENSG00000214711	si:ch211-202f3.4	ENSDARG00000091723	0.1169	0.0774	-1.6083	0.0000
ENSG00000164023	sgms2	ENSDARG00000052520	0.1155	1.0000	1.8074	0.0001
ENSG00000211450	C8H11orf31	ENSDARG00000093022	0.1155	0.0942	1.7697	0.0000
ENSG00000166145	spint1b	ENSDARG00000012467	0.1147	0.1671	-1.7872	0.0000
ENSG00000169105	chst14	ENSDARG00000043011	0.1142	0.3855	-1.7276	0.0000
ENSG00000079689	scgn	ENSDARG00000058732	0.1072	0.3076	-1.9598	0.0000
ENSG00000070729	CNGB1	ENSDARG00000042107	0.1062	0.0000	-2.1380	0.0000
ENSG00000026508	cd44a	ENSDARG00000087863	0.1043	0.8264	3.4330	0.0000
ENSG00000012211	prickle3	ENSDARG00000073996	0.1043	0.0162	-1.7898	0.0000
ENSG00000169856	onecut1	ENSDARG00000007982	0.1036	0.5104	1.7099	0.0000
ENSG00000139410	sdsl	ENSDARG00000074698	0.1031	0.7770	-2.1699	0.0000
ENSG00000182957	spata13	ENSDARG00000062837	0.1023	0.0758	1.7345	0.0000
ENSG00000008405	cry1aa	ENSDARG00000045768	0.1018	0.0000	-1.5817	0.0000
ENSG00000157851	dpysl5a	ENSDARG00000011141	0.0998	0.0000	2.0003	0.0000
ENSG00000123243	itih5	ENSDARG00000045517	0.0995	0.7807	2.2425	0.0000
ENSG00000131944	faap24	ENSDARG00000051731	0.0995	1.0000	2.2370	0.0000
ENSG00000130226	dpp6b	ENSDARG00000024744	0.0976	0.0000	-2.2886	0.0000
ENSG00000128973	cln6b	ENSDARG00000090002	0.0965	0.0000	-2.8689	0.0000
ENSG00000168418	KCNG4	ENSDARG00000051892	0.0933	0.0002	-4.3706	0.0000
ENSG00000123096	sspn	ENSDARG00000041747	0.0909	0.4636	-1.7975	0.0000
ENSG00000198691	abca4a	ENSDARG00000057169	0.0898	0.0000	-2.0313	0.0000
ENSG00000151500	thyn1	ENSDARG00000042659	0.0845	0.5069	1.9742	0.0000
ENSG00000171450	cdk5r2b	ENSDARG00000078671	0.0826	0.0000	-3.8109	0.0000
ENSG00000162374	elavl4	ENSDARG00000045639	0.0793	0.0042	1.9970	0.0000
ENSG00000187730	gabrd	ENSDARG00000059763	0.0784	0.0002	-1.5800	0.0000
ENSG00000149418	st14a	ENSDARG00000061173	0.0740	1.0000	1.5850	0.0000
ENSG00000170145	sik2a	ENSDARG00000079618	0.0735	0.0491	-2.9815	0.0000
ENSG00000037757	mri1	ENSDARG00000075754	0.0705	0.4839	2.1205	0.0000
ENSG00000143376	snx27a	ENSDARG00000033804	0.0656	0.0010	-1.9937	0.0000
ENSG00000151577	drd3	ENSDARG00000032131	0.0645	0.2244	-2.9990	0.0000
ENSG00000116703	pdca	ENSDARG00000011886	0.0618	0.0000	-3.2881	0.0000
ENSG00000185974	grk1a	ENSDARG00000058803	0.0572	0.0000	-1.9720	0.0000
ENSG00000133256	pde6b	ENSDARG00000011671	0.0570	0.0000	-2.6038	0.0000
ENSG00000168418	kcng4a	ENSDARG00000062967	0.0553	0.2306	-2.4017	0.0000
ENSG00000166912	mtmr10	ENSDARG00000010601	0.0552	0.0189	-1.6554	0.0000
ENSG00000116663	si:dkey-147f3.8	ENSDARG00000092166	0.0525	0.3084	2.3099	0.0000
ENSG00000074276	CDHR2	ENSDARG00000068586	0.0473	0.0357	-1.7167	0.0000
ENSG00000167325	rrm1	ENSDARG00000014017	0.0464	0.3182	1.9959	0.0000
ENSG00000205542	si:ch211-11c15.3	ENSDARG00000092970	0.0461	0.0097	1.6375	0.0000
ENSG00000170633	rnf34a	ENSDARG00000038300	0.0432	0.0357	-3.0133	0.0000
ENSG00000135916	itm2cb	ENSDARG00000039650	0.0426	0.0018	-1.7892	0.0000
ENSG00000167791	cabp2a	ENSDARG00000052016	0.0426	0.0003	-3.7633	0.0000
ENSG00000076685	nt5c2b	ENSDARG00000058231	0.0417	0.0739	-1.7620	0.0000
ENSG00000170775	gpr37b	ENSDARG00000033296	0.0371	0.5346	1.8388	0.0000
ENSG00000100399	chadla	ENSDARG00000055160	0.0356	1.0000	1.8480	0.0000
ENSG00000137561	ttpa	ENSDARG00000027584	0.0352	0.9378	-1.7549	0.0000
ENSG00000106078	cobl	ENSDARG00000004532	0.0350	0.3974	-1.9695	0.0000
ENSG00000145390	usp53b	ENSDARG00000076499	0.0346	0.4043	-1.6586	0.0000
ENSG00000164458	tb	ENSDARG00000039806	0.0318	0.8510	-2.7630	0.0000
ENSG00000134640	mtnr1bb	ENSDARG00000086493	0.0313	0.6495	-3.0212	0.0000
ENSG00000240771	arhgef25b	ENSDARG00000014465	0.0310	0.2042	-3.1019	0.0000
ENSG00000176393	rnpep	ENSDARG00000012506	0.0298	0.8014	1.6148	0.0000

ENSG00000186918	znf395b	ENSDARG00000024195	0.0286	0.2020	-1.9830	0.0000
ENSG00000105507	cabp5a	ENSDARG00000002576	0.0285	0.0000	-1.9938	0.0000
ENSG00000055332	eif2ak2	ENSDARG00000068729	0.0282	0.8227	1.5944	0.0000
ENSG00000126091	st3gal3b	ENSDARG00000015252	0.0276	0.3939	-2.5476	0.0000
ENSG00000149218	ENDOD1	ENSDARG00000063613	0.0242	1.0000	-1.6347	0.0000
ENSG00000127585	fbxl16	ENSDARG00000060915	0.0238	0.5044	-1.6162	0.0000
ENSG00000112116	il17a/f3	ENSDARG00000041976	0.0227	1.0000	1.7843	0.0000
ENSG00000139190	vamp1	ENSDARG00000031283	0.0221	0.1792	-2.3654	0.0000
ENSG00000172500	fibpb	ENSDARG00000087666	0.0211	0.8784	-2.0653	0.0000
ENSG00000112599	guca1b	ENSDARG00000013393	0.0195	0.0880	-2.2718	0.0000
ENSG00000182872	rbm10	ENSDARG00000077400	0.0193	0.6822	-1.8284	0.0000
ENSG00000171109	MFN1	ENSDARG00000011884	0.0171	0.3223	-1.8903	0.0000
ENSG00000143457	golph3l	ENSDARG00000036943	0.0153	0.9130	-1.9809	0.0000
ENSG00000148225	WDR31	ENSDARG00000073808	0.0140	0.6117	-2.7830	0.0000
ENSG00000137868	stra6	ENSDARG00000051874	0.0126	0.9391	3.4093	0.0000
ENSG00000071242	rps6ka2	ENSDARG00000028469	0.0059	0.8517	-3.5206	0.0000
ENSG00000170581	stat2	ENSDARG00000031647	0.0053	0.9088	2.1294	0.0000
ENSG00000095397	dfnb31a	ENSDARG00000075362	0.0041	0.8567	-1.8440	0.0000
ENSG00000178473	ucn3l	ENSDARG00000087241	0.0032	1.0000	-3.1699	0.0000
ENSG00000109047	rcvrna	ENSDARG00000052223	0.0026	0.7765	-2.0387	0.0000
ENSG00000117298	ece1	ENSDARG00000061737	0.0018	0.8898	-1.8504	0.0000
ENSG00000047617	ANO2	ENSDARG00000003210	0.0018	0.9368	-1.8480	0.0000
ENSG00000146276	gabrr1	ENSDARG00000043902	0.0013	0.9659	-4.2881	0.0000
ENSG00000213593	tmx2a	ENSDARG00000079122	0.0000	1.0000	3.3458	0.0000
ENSG00000108255	cryba1b	ENSDARG00000053875	0.0000	1.0000	2.6301	0.0000
ENSG00000129355	cdkn2d	ENSDARG00000094176	0.0000	1.0000	2.0255	0.0000
ENSG00000198756	COLGALT2	ENSDARG00000078299	0.0000	1.0000	-1.9107	0.0000
ENSG00000106686	spata6l	ENSDARG00000004874	0.0000	1.0000	-2.0369	0.0000
ENSG00000112773	fam46ab	ENSDARG00000054641	-0.0012	0.9674	-2.6337	0.0000
ENSG00000068383	inpp5l	ENSDARG00000053878	-0.0034	0.9322	-1.7407	0.0000
ENSG00000176871	wsb2	ENSDARG00000058608	-0.0041	0.9653	-1.9295	0.0000
ENSG00000113494	prlra	ENSDARG00000016570	-0.0047	0.7725	-3.9757	0.0000
ENSG00000101004	ninl	ENSDARG00000075119	-0.0058	0.8762	-1.7264	0.0000
ENSG00000073861	tbx21	ENSDARG00000004473	-0.0064	0.8209	-2.5456	0.0000
ENSG00000188486	h2afx	ENSDARG00000029406	-0.0068	0.9418	1.8965	0.0000
ENSG00000198892	SHISA4	ENSDARG00000071543	-0.0089	1.0000	2.4962	0.0000
ENSG00000114757	pex5la	ENSDARG00000022518	-0.0112	0.7333	-2.3258	0.0000
ENSG00000198515	cnga1	ENSDARG00000012125	-0.0190	0.0515	-2.0412	0.0000
ENSG00000109101	foxn1	ENSDARG00000011879	-0.0199	0.8656	-1.9837	0.0000
ENSG00000100031	ggt1a	ENSDARG00000023526	-0.0233	0.8723	-1.6560	0.0000
ENSG00000073803	MAP3K13	ENSDARG00000009493	-0.0298	0.3270	-1.7451	0.0000
ENSG00000134072	camk1b	ENSDARG00000029474	-0.0312	0.2378	-1.9491	0.0000
ENSG00000145888	glra1	ENSDARG00000012019	-0.0320	0.2873	-1.6603	0.0000
ENSG00000165119	hnrpkl	ENSDARG00000007034	-0.0330	0.7343	-1.8826	0.0000
ENSG00000125730	c3a.1	ENSDARG00000012694	-0.0375	0.8161	1.9798	0.0000
ENSG00000109819	ppargc1a	ENSDARG00000067829	-0.0393	0.1904	-1.9291	0.0000
ENSG00000069696	drd4b	ENSDARG00000035820	-0.0393	0.1315	-2.0943	0.0000
ENSG00000113240	clk4a	ENSDARG00000089372	-0.0397	0.0000	-1.6293	0.0000
ENSG00000174738	nr1d2b	ENSDARG00000009594	-0.0410	0.0000	-4.3623	0.0000
ENSG00000066027	ppp2r5a	ENSDARG00000027017	-0.0533	0.1449	-2.3015	0.0000
ENSG00000074590	nuak1b	ENSDARG00000028676	-0.0586	0.2541	2.1003	0.0000
ENSG00000128596	ccdc136a	ENSDARG00000075513	-0.0607	0.0398	-2.1448	0.0000
ENSG00000087237	cetp	ENSDARG00000030872	-0.0609	0.7951	-1.5969	0.0000
ENSG00000130589	helz2	ENSDARG00000016527	-0.0612	0.4489	2.4866	0.0000
ENSG00000166342	neto1	ENSDARG00000068483	-0.0628	0.0024	-2.6883	0.0000
ENSG00000183034	otop2	ENSDARG00000006522	-0.0642	0.4137	-1.7734	0.0000
ENSG00000183049	camk1db	ENSDARG00000017446	-0.0650	0.0021	-2.7185	0.0000
ENSG00000175287	phyhd1	ENSDARG00000029905	-0.0652	0.0481	-2.3194	0.0000

ENSG00000089639	gmip	ENSDARG00000077249	-0.0667	0.0006	-1.8475	0.0000
ENSG00000090621	pabpc4	ENSDARG00000059259	-0.0673	0.4447	1.7785	0.0000
ENSG00000102760	rgcc	ENSDARG00000035810	-0.0680	0.0684	-1.5990	0.0000
ENSG00000112242	e2f3	ENSDARG00000070463	-0.0690	0.7821	2.0034	0.0000
ENSG00000145416	march1	ENSDARG00000037487	-0.0692	0.0006	-2.2026	0.0000
ENSG00000164600	neurod6b	ENSDARG00000020794	-0.0700	0.6780	1.8353	0.0000
ENSG00000144711	iqsec1b	ENSDARG00000016551	-0.0701	0.0000	-1.5891	0.0000
ENSG00000162231	zgc:153681	ENSDARG00000086017	-0.0704	0.9122	-1.6919	0.0001
ENSG00000215845	TSTD1	ENSDARG00000071567	-0.0712	0.6755	1.6843	0.0000
ENSG00000074621	slc24a1	ENSDARG00000041431	-0.0712	0.0000	-1.9485	0.0000
ENSG00000111907	tpd52l1	ENSDARG00000042548	-0.0712	0.0019	-2.0703	0.0000
ENSG00000164122	asb5b	ENSDARG00000053222	-0.0736	0.0144	-3.0694	0.0000
ENSG00000166816	ldhd	ENSDARG00000038845	-0.0737	0.0177	-2.0209	0.0000
ENSG00000175175	ppm1e	ENSDARG00000026499	-0.0754	0.0000	-1.6854	0.0000
ENSG00000134255	cept1a	ENSDARG00000058716	-0.0794	0.5487	-2.0418	0.0000
ENSG00000151062	cacna2d4b	ENSDARG00000023886	-0.0803	0.0000	-1.7842	0.0000
ENSG00000112964	ghrb	ENSDARG00000007671	-0.0820	0.2379	-2.8168	0.0000
ENSG00000149295	drd2a	ENSDARG00000056926	-0.0825	0.6758	-2.0000	0.0000
ENSG00000008118	camk1gb	ENSDARG00000008788	-0.0830	0.0000	-2.3177	0.0000
ENSG00000007047	MARK4	ENSDARG00000024966	-0.0881	0.0000	-1.6810	0.0000
ENSG00000166317	synpo2lb	ENSDARG00000078696	-0.0881	0.3228	-2.1649	0.0000
ENSG00000068650	atp11a	ENSDARG00000008413	-0.0884	0.0000	-1.7594	0.0000
ENSG00000171385	kcnd3	ENSDARG00000056101	-0.0902	0.0052	-1.6539	0.0000
ENSG00000182132	kcnip1b	ENSDARG00000034808	-0.0922	0.0081	-2.1588	0.0000
ENSG00000137727	arhgap20	ENSDARG00000089326	-0.0929	0.0000	-1.8420	0.0000
ENSG00000134574	ddb2	ENSDARG00000041140	-0.0940	0.0014	-2.6755	0.0000
ENSG00000123700	kcnj2a	ENSDARG00000019418	-0.0941	0.2786	-2.1251	0.0000
ENSG00000062716	vmp1	ENSDARG00000012450	-0.0975	0.0028	1.6383	0.0000
ENSG00000173210	ablim3	ENSDARG00000088168	-0.1038	0.0036	-2.1207	0.0000
ENSG00000166689	plekha7a	ENSDARG00000060813	-0.1043	0.0002	-1.6360	0.0000
ENSG00000153832	fbxo36b	ENSDARG00000039152	-0.1069	1.0000	1.6521	0.0001
ENSG00000171246	nptx1l	ENSDARG00000074671	-0.1083	0.0000	-2.2175	0.0000
ENSG00000225697	slc26a6l	ENSDARG00000054127	-0.1095	0.2939	-1.7392	0.0000
ENSG00000166949	smad3b	ENSDARG00000010207	-0.1104	0.0000	-1.7507	0.0000
ENSG00000065154	oat	ENSDARG00000078425	-0.1128	0.0000	-1.9045	0.0000
ENSG00000151320	akap6	ENSDARG00000077295	-0.1147	0.0000	1.5948	0.0000
ENSG00000121621	kif18a	ENSDARG00000008022	-0.1155	0.8176	1.6335	0.0000
ENSG00000198732	smoc1	ENSDARG00000088255	-0.1200	0.3998	-1.6469	0.0000
ENSG00000144290	slc4a10b	ENSDARG00000060303	-0.1230	0.0000	-1.8997	0.0000
ENSG00000076826	CAMSAP3	ENSDARG00000091359	-0.1250	0.0001	1.6658	0.0000
ENSG00000167612	ankrd33ab	ENSDARG00000002508	-0.1252	0.0000	-5.0378	0.0000
ENSG00000055813	ccdc85al	ENSDARG00000005343	-0.1252	0.0000	-2.0189	0.0000
ENSG00000130413	stk33	ENSDARG00000054318	-0.1263	0.2952	-1.7162	0.0000
ENSG00000017483	slc38a5a	ENSDARG00000009901	-0.1265	0.0000	-2.9349	0.0000
ENSG00000167083	gngt2a	ENSDARG00000010680	-0.1272	0.0000	-1.7046	0.0000
ENSG00000012660	elovl5	ENSDARG00000004979	-0.1279	0.0065	-1.9074	0.0000
ENSG00000132915	pde6a	ENSDARG00000000380	-0.1302	0.0000	-2.7055	0.0000
ENSG00000167524	sgk494b	ENSDARG00000086169	-0.1319	0.0000	-2.5494	0.0000
ENSG00000188038	nrn1lb	ENSDARG00000061149	-0.1327	0.0000	-5.0166	0.0000
ENSG00000239474	klhl41a	ENSDARG00000068888	-0.1330	0.4049	-1.6152	0.0000
ENSG00000204291	col15a1b	ENSDARG00000061848	-0.1333	0.0000	-2.8794	0.0000
ENSG00000105516	dbpa	ENSDARG00000063014	-0.1342	0.0000	-3.5516	0.0000
ENSG00000168874	atoh8	ENSDARG00000037555	-0.1381	0.3158	-1.8480	0.0000
ENSG00000104267	ca2	ENSDARG00000014488	-0.1391	0.0000	-2.7088	0.0000
ENSG00000198780	fam169ab	ENSDARG00000004177	-0.1400	0.0000	-2.3056	0.0000
ENSG00000169181	gsg1l	ENSDARG00000037390	-0.1433	0.0138	-3.9989	0.0000
ENSG00000188886	ASTL	ENSDARG00000070011	-0.1459	0.6879	-1.7004	0.0000
ENSG00000170776	AKAP13	ENSDARG00000023868	-0.1478	0.0876	-1.6065	0.0000

ENSG00000123329	ARHGAP9	ENSDARG00000013390	-0.1512	0.0000	-2.7926	0.0000
ENSG00000154642	C10H21orf91	ENSDARG00000030803	-0.1522	0.0015	-1.9792	0.0000
ENSG00000239672	nme2a	ENSDARG00000043820	-0.1569	0.0000	-3.5327	0.0000
ENSG00000161509	grin2ca	ENSDARG00000078149	-0.1575	0.0000	-1.9852	0.0000
ENSG00000082126	MPP4	ENSDARG00000076872	-0.1576	0.0011	-1.7733	0.0000
ENSG00000101977	mcf2b	ENSDARG00000056603	-0.1598	0.0000	-2.0168	0.0000
ENSG00000119707	rbm25a	ENSDARG00000039266	-0.1620	0.0000	-2.4930	0.0000
ENSG00000164089	etnppl	ENSDARG00000035544	-0.1635	0.5259	-1.5850	0.0000
ENSG00000162494	lrrc38	ENSDARG00000075147	-0.1638	0.0858	-2.0202	0.0000
ENSG00000126368	nr1d1	ENSDARG00000033160	-0.1638	0.0000	-5.4870	0.0000
ENSG00000168748	ca7	ENSDARG00000045139	-0.1658	0.0000	-2.5917	0.0000
ENSG00000119139	tjp2a	ENSDARG00000063309	-0.1667	0.0000	-1.6943	0.0000
ENSG00000066926	fech	ENSDARG00000003462	-0.1669	0.1107	-2.0453	0.0000
ENSG00000171428	zgc:101040	ENSDARG00000005176	-0.1699	0.0175	-1.9267	0.0000
ENSG00000095777	myo3a	ENSDARG00000010186	-0.1710	0.0000	-3.2754	0.0000
ENSG00000111247	rad51ap1	ENSDARG00000045853	-0.1728	0.3955	1.6195	0.0000
ENSG00000104879	ckma	ENSDARG00000035327	-0.1741	0.0000	-2.0078	0.0000
ENSG00000023171	gramd1ba	ENSDARG00000075383	-0.1775	0.0000	-1.6501	0.0000
ENSG00000111241	fgf6a	ENSDARG00000009351	-0.1776	0.3058	1.8273	0.0000
ENSG00000163424	C5H3orf30	ENSDARG00000093783	-0.1787	0.4297	1.6444	0.0000
ENSG00000186297	gabra5	ENSDARG00000070730	-0.1791	0.0000	-2.9768	0.0000
ENSG00000107186	MPDZ	ENSDARG00000094677	-0.1811	0.0001	-1.7929	0.0000
ENSG00000009413	rev3l	ENSDARG00000058801	-0.1847	0.0114	-2.2218	0.0000
ENSG00000115350	pole4	ENSDARG00000013016	-0.1859	0.0000	-1.6957	0.0000
ENSG00000171680	plekhg5a	ENSDARG00000025902	-0.1868	0.0000	-1.9881	0.0000
ENSG00000188770	optc	ENSDARG00000014358	-0.1876	0.6488	3.1060	0.0000
ENSG00000072736	nfatc3a	ENSDARG00000076297	-0.1896	0.0000	-2.2764	0.0000
ENSG00000099998	ggt5a	ENSDARG00000052045	-0.1904	0.0000	-2.7443	0.0000
ENSG00000114857	nktr	ENSDARG00000059097	-0.1915	0.0000	-1.9210	0.0000
ENSG00000152192	pou4f1	ENSDARG00000005559	-0.1922	0.0001	1.7331	0.0000
ENSG00000156427	fgf18a	ENSDARG00000088048	-0.1926	0.5584	1.9408	0.0000
ENSG00000161647	mpp3b	ENSDARG00000062667	-0.1956	0.0000	-1.8413	0.0000
ENSG00000114805	plch1	ENSDARG00000079253	-0.1971	0.0194	-1.7117	0.0000
ENSG00000164363	slc6a18	ENSDARG00000068387	-0.1985	0.1509	-1.6562	0.0000
ENSG00000116761	cthl	ENSDARG00000032206	-0.1988	0.0162	-1.6355	0.0000
ENSG00000109158	GABRA4	ENSDARG00000013389	-0.2024	0.0088	-2.8495	0.0000
ENSG00000263761	gdf2	ENSDARG00000059173	-0.2040	0.0969	-3.5803	0.0000
ENSG00000167791	cabp2b	ENSDARG00000052277	-0.2052	0.0000	-2.1076	0.0000
ENSG00000132359	rap1gap2a	ENSDARG00000061551	-0.2057	0.0000	-1.6240	0.0000
ENSG00000108924	hlfb	ENSDARG00000061011	-0.2061	0.0000	-1.7001	0.0000
ENSG00000253958	CLDN23	ENSDARG00000089553	-0.2062	0.0312	-1.7265	0.0000
ENSG00000138161	cuzd1.1	ENSDARG00000089050	-0.2063	0.0007	-4.5755	0.0000
ENSG00000135835	si:ch211-13f8.1	ENSDARG00000093342	-0.2068	0.0000	-4.8404	0.0000
ENSG00000155307	samsn1b	ENSDARG00000078647	-0.2072	0.0000	-3.3701	0.0000
ENSG00000107372	zfand5a	ENSDARG00000018898	-0.2074	0.0000	-1.7597	0.0000
ENSG00000006453	baiap2l1b	ENSDARG00000031119	-0.2098	0.0000	-1.5875	0.0000
ENSG00000113302	IL12B	ENSDARG00000069504	-0.2115	0.5811	2.0244	0.0000
ENSG00000205268	pde7a	ENSDARG00000045305	-0.2119	0.0000	-2.0989	0.0000
ENSG00000143340	FAM163A	ENSDARG00000093021	-0.2119	0.0161	-1.9173	0.0000
ENSG00000196358	ntng2a	ENSDARG00000077367	-0.2129	0.0000	-1.9666	0.0000
ENSG00000165886	ubtd1a	ENSDARG00000007382	-0.2130	0.0000	-3.1670	0.0000
ENSG00000169184	mn1a	ENSDARG00000076529	-0.2268	0.0000	-2.5551	0.0000
ENSG00000149679	cables2b	ENSDARG00000017154	-0.2291	0.0000	-2.8182	0.0000
ENSG00000157087	atp2b2	ENSDARG00000063433	-0.2320	0.0000	-1.8263	0.0000
ENSG00000176834	vsig10	ENSDARG00000058605	-0.2324	0.0026	-1.7301	0.0000
ENSG00000104332	sfrp1b	ENSDARG00000057678	-0.2345	0.5614	1.8176	0.0000
ENSG00000102057	kcnd1	ENSDARG00000074746	-0.2355	0.0000	-1.6400	0.0000
ENSG00000171773	nxnl1	ENSDARG00000052035	-0.2382	0.0000	-2.7430	0.0000

ENSG00000182107	TMEM30B	ENSDARG00000054122	-0.2410	0.3594	-1.6415	0.0000
ENSG00000101890	gc2	ENSDARG00000018329	-0.2419	0.0000	-2.1366	0.0000
ENSG00000170049	KCNAB3	ENSDARG00000074320	-0.2421	0.0000	-1.9152	0.0000
ENSG00000008405	cry1ab	ENSDARG00000011583	-0.2432	0.0000	-2.0418	0.0000
ENSG00000145864	gabrb2	ENSDARG00000079586	-0.2439	0.0000	-1.6879	0.0000
ENSG00000168843	fstl5	ENSDARG00000040198	-0.2489	0.0000	-1.6606	0.0000
ENSG00000198822	grm3	ENSDARG00000031712	-0.2489	0.0000	-1.8498	0.0000
ENSG00000147145	LPAR4	ENSDARG00000089824	-0.2494	0.1199	-1.8898	0.0000
ENSG00000187902	shisa7a	ENSDARG00000062462	-0.2499	0.0007	-2.0051	0.0000
ENSG00000180616	SSTR2	ENSDARG00000059090	-0.2502	0.0291	-1.8400	0.0000
ENSG00000088179	ptpn4b	ENSDARG00000000183	-0.2505	0.0000	-1.7036	0.0000
ENSG00000069696	drd4a	ENSDARG00000038363	-0.2510	0.0000	-1.7723	0.0000
ENSG00000104888	slc17a7a	ENSDARG00000016480	-0.2526	0.0000	-3.2100	0.0000
ENSG00000180914	oxtr	ENSDARG00000033956	-0.2530	0.1748	-2.1155	0.0000
ENSG00000179134	SAMD4B	ENSDARG00000086735	-0.2548	0.0000	-2.6147	0.0000
ENSG00000197535	myo5ab	ENSDARG00000025218	-0.2563	0.0000	-2.6076	0.0000
ENSG00000099204	ablim1b	ENSDARG00000045064	-0.2564	0.0000	-2.8306	0.0000
ENSG00000001617	sema3fa	ENSDARG00000011163	-0.2569	0.0000	-1.6156	0.0000
ENSG00000107954	neurl1aa	ENSDARG00000069438	-0.2577	0.0000	-2.5166	0.0000
ENSG00000171208	neto2b	ENSDARG00000063293	-0.2582	0.0000	-2.9011	0.0000
ENSG00000179094	per1a	ENSDARG00000056885	-0.2607	0.0000	-5.2003	0.0000
ENSG00000135299	ankrd6b	ENSDARG00000029370	-0.2607	0.0000	-2.8687	0.0000
ENSG00000182253	synm	ENSDARG00000062350	-0.2613	0.0000	-2.6013	0.0000
ENSG00000108924	hlfa	ENSDARG00000074752	-0.2626	0.0000	-2.4783	0.0000
ENSG00000139537	ccdc65	ENSDARG00000032005	-0.2630	0.8318	3.1964	0.0000
ENSG00000187391	magi2a	ENSDARG00000021590	-0.2670	0.0000	-1.6985	0.0000
ENSG00000185149	npy2rl	ENSDARG00000089943	-0.2679	0.3866	-1.7952	0.0000
ENSG00000184678	si:ch211-113a14.22	ENSDARG00000039547	-0.2692	0.4505	-1.9696	0.0000
ENSG00000188452	cerkl	ENSDARG00000079397	-0.2692	0.0000	-2.4354	0.0000
ENSG00000147509	rgs20	ENSDARG00000038859	-0.2722	0.0000	-4.0187	0.0000
ENSG00000143858	SYT2	ENSDARG00000014169	-0.2736	0.0000	-1.7818	0.0000
ENSG00000110717	ndufs8b	ENSDARG00000057055	-0.2756	0.0000	-2.7102	0.0000
ENSG00000120694	hsph1	ENSDARG00000019874	-0.2762	0.0000	-1.9959	0.0000
ENSG00000092850	tekt2	ENSDARG00000028973	-0.2801	0.5258	-1.7655	0.0004
ENSG00000129680	map7d3	ENSDARG00000060771	-0.2804	0.0000	-1.6672	0.0000
ENSG00000120645	iqsec3b	ENSDARG00000093091	-0.2831	0.0000	-2.1396	0.0000
ENSG00000109103	unc119b	ENSDARG00000044362	-0.2860	0.0000	-2.3183	0.0000
ENSG00000105711	scn1ba	ENSDARG00000060222	-0.2881	0.0000	-2.0357	0.0000
ENSG00000154099	dnaaf1	ENSDARG00000012030	-0.2886	0.0000	-4.6277	0.0000
ENSG00000151615	pou4f2	ENSDARG00000069737	-0.2891	0.0000	1.6369	0.0000
ENSG00000174099	msrb3	ENSDARG00000045658	-0.2895	0.0000	-1.7414	0.0000
ENSG00000158985	cdc42se2	ENSDARG00000094577	-0.2896	0.0000	-2.0084	0.0000
ENSG00000082482	kcnk2a	ENSDARG00000055123	-0.2908	0.0354	-1.7105	0.0000
ENSG00000178026	LRRC75B	ENSDARG00000091128	-0.2919	0.0000	-1.6836	0.0000
ENSG00000164830	oxr1b	ENSDARG00000063310	-0.2938	0.0000	-1.9750	0.0000
ENSG00000148483	TMEM236	ENSDARG00000075715	-0.2944	0.0151	-2.8849	0.0000
ENSG00000204396	vwa7	ENSDARG00000061462	-0.2982	0.0153	2.3072	0.0000
ENSG00000135362	PRR5L	ENSDARG00000029431	-0.2991	0.0001	-1.6109	0.0000
ENSG00000144671	SLC22A14	ENSDARG00000091222	-0.2992	0.0000	-4.5161	0.0000
ENSG00000198722	unc13ba	ENSDARG00000017391	-0.2999	0.0000	-1.8532	0.0000
ENSG00000167552	tuba1c	ENSDARG00000055216	-0.3000	0.0000	1.8292	0.0000
ENSG00000152495	camk4	ENSDARG00000005372	-0.3007	0.0000	-1.9009	0.0000
ENSG00000198515	CNGA1	ENSDARG00000029898	-0.3008	0.0000	-1.7872	0.0000
ENSG00000215906	lactbl1a	ENSDARG00000089063	-0.3048	0.0000	-4.0383	0.0000
ENSG00000187288	cidec	ENSDARG00000059651	-0.3081	0.5601	1.8845	0.0000
ENSG00000256349	bbs1	ENSDARG00000075169	-0.3096	0.0000	-1.6827	0.0000
ENSG00000017427	igf1	ENSDARG00000094132	-0.3096	0.0000	-2.4183	0.0000
ENSG00000183423	lrit3a	ENSDARG00000055463	-0.3099	0.0000	-2.6172	0.0000

ENSG00000103415	hmox2a	ENSDARG00000055101	-0.3106	0.0000	-1.7530	0.0000
ENSG00000168710	ahcyl1	ENSDARG00000056331	-0.3115	0.0000	-1.8612	0.0000
ENSG00000112619	prph2a	ENSDARG00000038018	-0.3138	0.0000	-2.5207	0.0000
ENSG00000173457	ppp1r14ba	ENSDARG00000044541	-0.3178	0.0000	1.7923	0.0000
ENSG00000163075	CFAP221	ENSDARG00000087107	-0.3219	0.6177	1.6323	0.0000
ENSG00000174453	vwc2l	ENSDARG00000069134	-0.3219	0.0000	-1.5862	0.0000
ENSG00000138166	dusp5	ENSDARG00000019307	-0.3224	0.0000	-1.9042	0.0000
ENSG00000082556	oprk1	ENSDARG00000006894	-0.3234	0.0010	-2.1126	0.0000
ENSG00000101166	slmo2	ENSDARG00000009505	-0.3266	0.0000	-1.9006	0.0000
ENSG00000109103	unc119a	ENSDARG00000034453	-0.3277	0.0000	-3.0081	0.0000
ENSG00000173960	ubxn2a	ENSDARG00000087180	-0.3287	0.0000	-1.7265	0.0000
ENSG00000157764	braf	ENSDARG00000017661	-0.3299	0.0000	-1.6047	0.0000
ENSG00000118276	b4galt6	ENSDARG00000010301	-0.3326	0.0000	-1.8053	0.0000
ENSG00000154678	PDE1C	ENSDARG00000090398	-0.3339	0.0001	-2.6972	0.0000
ENSG00000156313	rpgra	ENSDARG00000056617	-0.3351	0.0000	-3.5830	0.0000
ENSG00000113319	rasgrf2b	ENSDARG00000002816	-0.3369	0.0000	-3.0996	0.0000
ENSG00000056291	npffr2a	ENSDARG00000033635	-0.3391	0.0145	-1.9169	0.0000
ENSG00000102104	rs1a	ENSDARG00000027236	-0.3420	0.0000	-2.5447	0.0000
ENSG00000159208	ciarta	ENSDARG00000058094	-0.3439	0.0000	-2.9925	0.0000
ENSG00000112706	impg1a	ENSDARG00000077187	-0.3443	0.0000	-2.5510	0.0000
ENSG00000198794	scamp5b	ENSDARG00000040274	-0.3448	0.0000	-1.6220	0.0000
ENSG00000141447	OSBPL1A	ENSDARG00000053746	-0.3466	0.0000	-3.2119	0.0000
ENSG00000157601	mxb	ENSDARG00000004953	-0.3479	0.6900	2.9475	0.0000
ENSG00000141665	FBXO15	ENSDARG00000029848	-0.3504	0.0000	-2.6693	0.0000
ENSG00000136535	tbr1b	ENSDARG00000004712	-0.3512	0.0257	1.6395	0.0000
ENSG00000124140	slc12a5b	ENSDARG00000078187	-0.3533	0.0000	-1.6092	0.0000
ENSG00000139287	tph2	ENSDARG00000057239	-0.3545	0.0072	-1.7030	0.0000
ENSG00000082438	cobll1a	ENSDARG00000089858	-0.3558	0.0000	-1.7933	0.0000
ENSG00000153936	hs2st1b	ENSDARG00000062008	-0.3583	0.0000	2.6943	0.0000
ENSG00000275572	grifin	ENSDARG00000033382	-0.3587	0.0078	-2.1592	0.0000
ENSG00000146950	SHROOM2	ENSDARG00000076416	-0.3589	0.0000	-1.6247	0.0000
ENSG00000143469	syt14b	ENSDARG00000001891	-0.3612	0.0000	-2.3052	0.0000
ENSG00000070601	frmpd1a	ENSDARG00000079074	-0.3614	0.0000	-3.3965	0.0000
ENSG00000106633	gck	ENSDARG00000068006	-0.3626	0.4709	5.6071	0.0000
ENSG00000150627	wdr17	ENSDARG00000075098	-0.3639	0.0000	-1.6901	0.0000
ENSG00000126759	cfp	ENSDARG00000094451	-0.3643	0.0760	2.8125	0.0000
ENSG00000085491	slc25a24	ENSDARG00000008568	-0.3646	0.0000	-3.2481	0.0000
ENSG00000167615	leng8	ENSDARG00000076805	-0.3678	0.0000	-1.7217	0.0000
ENSG00000116703	pdcb	ENSDARG00000017634	-0.3679	0.0000	-4.8076	0.0000
ENSG00000113318	msh3	ENSDARG00000063276	-0.3704	0.0002	-1.6683	0.0000
ENSG00000117394	slc2a1a	ENSDARG00000001437	-0.3706	0.0000	-4.5665	0.0000
ENSG00000158246	fam46bb	ENSDARG00000011797	-0.3711	0.0009	-2.3711	0.0000
ENSG00000150995	itpr1b	ENSDARG00000074149	-0.3755	0.0000	-1.8929	0.0000
ENSG00000106348	impdh1a	ENSDARG00000042336	-0.3755	0.0000	-3.4886	0.0000
ENSG00000065054	CABZ01067151.1	ENSDARG00000057669	-0.3764	0.0000	-4.9878	0.0000
ENSG00000167191	gprc5bb	ENSDARG00000075141	-0.3799	0.0000	-1.8766	0.0000
ENSG00000187079	tead1b	ENSDARG00000059483	-0.3824	0.0000	-1.7927	0.0000
ENSG00000205643	CDPF1	ENSDARG00000095705	-0.3837	0.0000	-2.0377	0.0000
ENSG00000170011	MYRIP	ENSDARG00000075706	-0.3858	0.0000	-1.7203	0.0000
ENSG00000081248	cacna1sa	ENSDARG00000029457	-0.3864	0.0000	-1.5806	0.0000
ENSG00000186862	pdzd7a	ENSDARG00000076974	-0.3871	0.0000	-1.9742	0.0000
ENSG00000001617	sema3fb	ENSDARG00000055373	-0.3899	0.0000	-1.7718	0.0000
ENSG00000164117	fbxo8	ENSDARG00000007477	-0.3899	0.0000	-1.6943	0.0000
ENSG00000077522	actn2b	ENSDARG00000071090	-0.3920	0.0000	-3.2516	0.0000
ENSG00000185052	slc24a3	ENSDARG00000006760	-0.3940	0.0000	-2.7355	0.0000
ENSG00000148600	cdhr1a	ENSDARG00000004643	-0.3946	0.0000	-1.9302	0.0000
ENSG00000174564	crfb16	ENSDARG00000075181	-0.3957	0.0006	-1.8629	0.0000
ENSG00000136297	mmd2a	ENSDARG00000087446	-0.3965	0.0000	-2.3465	0.0000

ENSG00000197321	svilb	ENSDARG00000014651	-0.3969	0.0000	-1.7474	0.0000
ENSG00000131089	arhgef9a	ENSDARG00000061746	-0.3971	0.0000	-2.4666	0.0000
ENSG00000128298	baiap2l2b	ENSDARG00000060933	-0.3974	0.0000	-3.1972	0.0000
ENSG00000145087	stxbp5l	ENSDARG00000006383	-0.3995	0.0000	-1.9119	0.0000
ENSG00000179094	per1b	ENSDARG00000012499	-0.4009	0.0000	-4.9594	0.0000
ENSG00000006025	osbpl7	ENSDARG00000012981	-0.4011	0.0000	-4.2553	0.0000
ENSG00000055118	kcnh2a	ENSDARG00000029881	-0.4022	0.0000	-1.5914	0.0000
ENSG00000213160	klhl23	ENSDARG00000078475	-0.4039	0.0000	-2.5492	0.0000
ENSG00000050438	slc4a8	ENSDARG00000015531	-0.4039	0.0000	-2.0358	0.0000
ENSG00000169891	REPS2	ENSDARG00000076768	-0.4050	0.0000	-1.6573	0.0000
ENSG00000139517	lnx2a	ENSDARG00000029177	-0.4060	0.0000	-2.4530	0.0000
ENSG00000185652	ntf3	ENSDARG00000059043	-0.4068	0.0000	-1.6764	0.0000
ENSG00000126822	PLEKHG3	ENSDARG00000058835	-0.4068	0.0000	-2.9595	0.0000
ENSG00000198894	cipca	ENSDARG00000075397	-0.4079	0.0000	-4.0015	0.0000
ENSG00000155975	vps37a	ENSDARG00000017119	-0.4098	0.0000	-1.9181	0.0000
ENSG00000070748	chata	ENSDARG00000015854	-0.4098	0.0000	-1.6882	0.0000
ENSG00000116237	icmt	ENSDARG00000020241	-0.4102	0.0000	-1.7060	0.0000
ENSG00000144057	st6gal2b	ENSDARG00000088515	-0.4120	0.0000	-2.1285	0.0000
ENSG00000148110	CU856539.1	ENSDARG00000010023	-0.4123	0.0000	-2.2843	0.0000
ENSG00000150510	FAM124A	ENSDARG00000075264	-0.4133	0.0000	-2.2297	0.0000
ENSG00000145349	camk2d1	ENSDARG00000043010	-0.4136	0.0000	-2.6312	0.0000
ENSG00000125534	ppdpfa	ENSDARG00000007682	-0.4139	0.0000	-2.7577	0.0000
ENSG00000197442	map3k5	ENSDARG00000005416	-0.4143	0.0000	-1.6638	0.0000
ENSG00000104951	IL4I1	ENSDARG00000056804	-0.4150	0.3916	3.0506	0.0000
ENSG00000148803	fuom	ENSDARG00000039422	-0.4170	0.0000	-2.0331	0.0000
ENSG00000101892	atp1b4	ENSDARG00000053262	-0.4181	0.0000	-3.0270	0.0000
ENSG00000185324	cdk10	ENSDARG00000034256	-0.4193	0.0000	-1.8021	0.0000
ENSG00000254647	insb	ENSDARG00000034610	-0.4224	0.0000	-3.3994	0.0000
ENSG00000112280	col9a1b	ENSDARG00000031483	-0.4229	0.0104	2.4459	0.0000
ENSG00000165623	ucmab	ENSDARG00000005485	-0.4236	0.0002	-1.9602	0.0000
ENSG00000179270	C20H2orf71	ENSDARG00000095802	-0.4253	0.0000	-1.5955	0.0000
ENSG00000280165	PCDH20	ENSDARG00000036424	-0.4274	0.0078	-2.4780	0.0000
ENSG00000144191	cnga3b	ENSDARG00000012297	-0.4307	0.0000	-3.8271	0.0000
ENSG00000111262	KCNA1	ENSDARG00000017108	-0.4317	0.0000	-1.6996	0.0000
ENSG00000104369	jph1b	ENSDARG00000038826	-0.4336	0.0000	-1.6521	0.0000
ENSG00000150656	CNDP1	ENSDARG00000069583	-0.4355	0.0000	2.1123	0.0000
ENSG00000250479	chchd10	ENSDARG00000010717	-0.4391	0.0000	-2.2075	0.0000
ENSG00000139835	grtp1b	ENSDARG00000030269	-0.4399	0.0000	-1.8808	0.0000
ENSG00000078549	adcyap1r1b	ENSDARG00000053724	-0.4442	0.0000	-2.6160	0.0000
ENSG00000115520	coq10b	ENSDARG00000056141	-0.4443	0.0000	-2.1554	0.0000
ENSG00000117650	nek2	ENSDARG00000005619	-0.4457	0.0031	1.7114	0.0000
ENSG00000079335	cdc14ab	ENSDARG00000057016	-0.4458	0.0000	-3.7684	0.0000
ENSG00000134802	slc43a3a	ENSDARG00000059682	-0.4484	0.0000	-1.9679	0.0000
ENSG00000244405	etv5b	ENSDARG00000044511	-0.4494	0.0000	-2.6829	0.0000
ENSG00000069966	gnb5b	ENSDARG00000055377	-0.4494	0.0000	-2.0166	0.0000
ENSG00000170264	fam161a	ENSDARG00000089742	-0.4506	0.0000	-3.3231	0.0000
ENSG00000137699	ftr30	ENSDARG00000095448	-0.4523	0.0000	-2.7292	0.0000
ENSG00000114302	prkar2ab	ENSDARG00000009477	-0.4525	0.0000	-1.6466	0.0000
ENSG00000054179	entpd2b	ENSDARG00000044795	-0.4525	0.3713	3.1513	0.0000
ENSG00000101298	snpha	ENSDARG00000078485	-0.4531	0.0000	-2.1615	0.0000
ENSG00000143375	cgnb	ENSDARG00000058690	-0.4544	0.0000	-3.1414	0.0000
ENSG00000181656	gpr88	ENSDARG00000043122	-0.4564	0.1371	-1.9758	0.0000
ENSG00000107242	pip5k1ba	ENSDARG00000044295	-0.4602	0.0000	-2.6516	0.0000
ENSG00000143125	prok1	ENSDARG00000073948	-0.4612	0.0000	-1.8569	0.0000
ENSG00000083067	trpm3	ENSDARG00000039181	-0.4617	0.0000	-1.9208	0.0000
ENSG00000163794	uts1	ENSDARG00000014927	-0.4638	0.0000	-2.3638	0.0000
ENSG00000099260	palmda	ENSDARG00000001913	-0.4639	0.0000	-1.8448	0.0000
ENSG00000070526	st6galnac1.1	ENSDARG00000043814	-0.4653	0.0000	-2.5019	0.0000

ENSG00000182324	kcnj14	ENSDARG00000075914	-0.4654	0.0000	-2.3432	0.0000
ENSG00000112706	impg1b	ENSDARG00000074839	-0.4658	0.0000	-2.8607	0.0000
ENSG00000106302	hyal4	ENSDARG00000051823	-0.4658	0.0000	-1.9335	0.0000
ENSG00000084070	smap2	ENSDARG00000061446	-0.4667	0.0000	-1.7601	0.0000
ENSG00000141741	sepw2b	ENSDARG00000089936	-0.4671	0.1821	-2.0952	0.0000
ENSG00000013293	slc7a14b	ENSDARG00000079231	-0.4695	0.1462	2.7370	0.0000
ENSG00000163793	dnajc5ga	ENSDARG00000041896	-0.4698	0.0000	-1.7829	0.0000
ENSG00000168135	KCNJ4	ENSDARG00000068110	-0.4732	0.0000	-2.1317	0.0000
ENSG00000185432	mettl7a	ENSDARG00000056726	-0.4751	0.0000	-1.6534	0.0000
ENSG00000137501	SYTL2	ENSDARG00000061956	-0.4772	0.0000	-2.9306	0.0000
ENSG00000163071	spata18	ENSDARG00000052343	-0.4774	0.0001	-2.6630	0.0000
ENSG00000008513	st3gal1l	ENSDARG00000079310	-0.4800	0.1418	-2.4060	0.0000
ENSG00000165322	arhgap12a	ENSDARG00000008548	-0.4828	0.0000	-2.4156	0.0000
ENSG00000197580	bco2a	ENSDARG00000055722	-0.4838	0.0000	-3.4987	0.0000
ENSG00000197535	myo5ab	ENSDARG00000074622	-0.4844	0.0000	-2.4699	0.0000
ENSG00000220201	zglp1	ENSDARG00000067669	-0.4854	0.1945	-1.8074	0.0001
ENSG00000122643	nt5c3a	ENSDARG00000058597	-0.4866	0.0000	-2.9803	0.0000
ENSG00000162877	pm20d1.2	ENSDARG00000062096	-0.4868	0.0000	-1.7024	0.0000
ENSG00000161958	fgf11b	ENSDARG00000043907	-0.4871	0.0000	-2.8475	0.0000
ENSG00000157131	c8a	ENSDARG00000039516	-0.4872	0.0060	-2.7137	0.0000
ENSG00000172175	malt1b	ENSDARG00000077768	-0.4877	0.0000	-1.9618	0.0000
ENSG00000104419	ndrg1a	ENSDARG00000032849	-0.4877	0.0000	-1.7257	0.0000
ENSG00000115353	tacr1a	ENSDARG00000035533	-0.4882	0.0000	-2.5126	0.0000
ENSG00000149573	mpzl2b	ENSDARG00000027345	-0.4906	0.0000	-2.4369	0.0000
ENSG00000269964	mei4	ENSDARG00000043912	-0.4923	0.0002	-2.4183	0.0000
ENSG00000183873	scn12aa	ENSDARG00000090724	-0.4934	0.0000	-2.0172	0.0000
ENSG00000173598	nudt4b	ENSDARG00000045878	-0.4944	0.0000	-1.6941	0.0000
ENSG00000175745	nr2f1a	ENSDARG00000052695	-0.4945	0.0000	-1.6618	0.0000
ENSG00000115255	reep6	ENSDARG00000087349	-0.4948	0.0000	-2.0000	0.0000
ENSG00000115041	kcnip3b	ENSDARG00000017880	-0.4959	0.0000	-2.0595	0.0000
ENSG00000004660	camkk1b	ENSDARG00000015134	-0.4973	0.0000	-7.6498	0.0000
ENSG00000185989	rasa3	ENSDARG00000063371	-0.5004	0.0000	-2.0774	0.0000
ENSG00000115461	igfbp5a	ENSDARG00000039264	-0.5005	0.0000	-1.9295	0.0000
ENSG00000123836	pfkfb2b	ENSDARG00000002037	-0.5019	0.0000	-2.6913	0.0000
ENSG00000160097	fndc5a	ENSDARG00000069739	-0.5022	0.0000	-2.9842	0.0000
ENSG00000178951	zbtb7a	ENSDARG00000039899	-0.5031	0.0000	-1.8541	0.0000
ENSG00000008441	nfixa	ENSDARG00000043226	-0.5040	0.0000	-1.7884	0.0000
ENSG00000166272	wbp1lb	ENSDARG00000029751	-0.5058	0.0000	-1.8815	0.0000
ENSG00000104879	ckmb	ENSDARG00000040565	-0.5091	0.0000	-1.6450	0.0000
ENSG00000143217	PVRL4	ENSDARG00000077419	-0.5092	0.0002	-1.8235	0.0000
ENSG00000040933	inpp4ab	ENSDARG00000070995	-0.5115	0.0000	-2.7108	0.0000
ENSG00000206538	VGLL3	ENSDARG00000038305	-0.5115	0.0747	-1.6067	0.0000
ENSG00000014216	CAPN1	ENSDARG00000055338	-0.5116	0.0000	-2.5835	0.0000
ENSG00000120457	kcnj5	ENSDARG00000061014	-0.5120	0.0000	-1.7101	0.0000
ENSG00000182492	bgna	ENSDARG00000017884	-0.5122	0.0000	-4.0193	0.0000
ENSG00000246922	ubap1la	ENSDARG00000089217	-0.5151	0.0000	-3.5217	0.0000
ENSG00000089558	kcnh4b	ENSDARG00000061990	-0.5151	0.0000	-2.9126	0.0000
ENSG00000186765	fscn2a	ENSDARG00000059574	-0.5154	0.0000	-2.5134	0.0000
ENSG00000172137	calb2b	ENSDARG00000036344	-0.5161	0.0000	-1.7068	0.0000
ENSG00000168994	pxdc1a	ENSDARG00000055177	-0.5164	0.0000	-3.8773	0.0000
ENSG00000123505	amd1	ENSDARG00000043856	-0.5186	0.0000	-3.2383	0.0000
ENSG00000128805	ARHGAP22	ENSDARG00000076434	-0.5275	0.0000	1.9202	0.0000
ENSG00000091428	rapgef4	ENSDARG00000079872	-0.5296	0.0000	-2.2179	0.0000
ENSG00000155755	tmem237a	ENSDARG00000041735	-0.5321	0.0000	-2.8129	0.0000
ENSG00000181408	UTS2R	ENSDARG00000009624	-0.5340	0.0060	-1.7970	0.0000
ENSG00000213889	ppm1nb	ENSDARG00000057032	-0.5343	0.0000	-1.9849	0.0000
ENSG00000117477	CCDC181	ENSDARG00000062021	-0.5352	0.0000	-2.2358	0.0000
ENSG00000115977	aak1a	ENSDARG00000011855	-0.5352	0.0000	-1.7310	0.0000

ENSG00000080493	slc4a4b	ENSDARG00000044808	-0.5359	0.0000	-2.3411	0.0000
ENSG00000164742	adcy1b	ENSDARG00000088634	-0.5367	0.0000	-2.8021	0.0000
ENSG00000111664	gnb3b	ENSDARG00000002696	-0.5384	0.0000	-2.3444	0.0000
ENSG00000025156	hsf2	ENSDARG00000053097	-0.5401	0.0000	-2.7703	0.0000
ENSG00000009724	masp2	ENSDARG00000007988	-0.5425	0.0594	-1.6738	0.0000
ENSG00000139835	grtp1a	ENSDARG00000029936	-0.5429	0.0000	-2.1261	0.0000
ENSG00000152270	PDE3B	ENSDARG00000062190	-0.5437	0.0000	-1.9764	0.0000
ENSG00000159423	aldh4a1	ENSDARG00000038207	-0.5460	0.0000	-2.2555	0.0000
ENSG00000170633	rnf34b	ENSDARG00000054036	-0.5479	0.0000	-2.6953	0.0000
ENSG00000176463	slco3a1	ENSDARG00000018726	-0.5483	0.0000	-1.8339	0.0000
ENSG00000074855	ano8a	ENSDARG00000076265	-0.5538	0.0000	-2.4021	0.0000
ENSG00000143850	plekha6	ENSDARG00000020328	-0.5565	0.0000	-2.3300	0.0000
ENSG00000111886	gabrr2a	ENSDARG00000052982	-0.5603	0.0000	-3.5112	0.0000
ENSG00000186566	gpatch8	ENSDARG00000059768	-0.5610	0.0000	-1.6517	0.0000
ENSG00000142675	cnksr1	ENSDARG00000032932	-0.5620	0.0000	-3.7222	0.0000
ENSG00000112541	pde10a	ENSDARG00000003449	-0.5648	0.0000	-2.3705	0.0000
ENSG00000106236	nptx2b	ENSDARG00000045164	-0.5693	0.0000	-3.4853	0.0000
ENSG00000169231	thbs3a	ENSDARG00000077641	-0.5701	0.0000	2.0513	0.0000
ENSG00000119946	CNNM1	ENSDARG00000078087	-0.5706	0.0000	-3.6016	0.0000
ENSG00000196358	ntng2b	ENSDARG00000087100	-0.5714	0.0000	-2.1289	0.0000
ENSG00000198814	GK2	ENSDARG00000053456	-0.5714	0.0000	-1.5904	0.0000
ENSG00000128283	cdc42ep1b	ENSDARG00000077792	-0.5745	0.0000	-1.6751	0.0000
ENSG00000160570	dedd1	ENSDARG00000002758	-0.5759	0.0000	-2.5056	0.0000
ENSG00000063127	slc6a16a	ENSDARG00000007129	-0.5759	0.0000	-1.7628	0.0000
ENSG00000185518	sv2ba	ENSDARG00000057427	-0.5786	0.0000	-2.3041	0.0000
ENSG00000182077	PTCHD3	ENSDARG00000010075	-0.5800	0.0000	-2.1073	0.0000
ENSG00000164070	hspa4l	ENSDARG00000053544	-0.5830	0.0000	-2.1773	0.0000
ENSG00000163472	tmem79a	ENSDARG00000071250	-0.5850	0.0929	-1.7776	0.0000
ENSG00000163026	C20H2orf44	ENSDARG00000016528	-0.5855	0.0000	-2.4777	0.0000
ENSG00000135175	pvalb9	ENSDARG00000071601	-0.5891	0.0000	-3.9461	0.0000
ENSG00000006016	crlf1b	ENSDARG00000006368	-0.5911	0.0000	-1.6925	0.0000
ENSG00000064042	limch1a	ENSDARG00000074275	-0.5913	0.0000	-1.5984	0.0000
ENSG00000138472	guca1d	ENSDARG00000044629	-0.5916	0.0000	-6.8723	0.0000
ENSG00000068724	ttc7a	ENSDARG00000074760	-0.5940	0.0000	-2.3255	0.0000
ENSG00000139508	SLC46A3	ENSDARG00000079933	-0.5969	0.0000	-1.8018	0.0000
ENSG00000177989	odf3b	ENSDARG00000042391	-0.5972	0.0000	-2.4046	0.0000
ENSG00000158079	ptpdc1b	ENSDARG00000058873	-0.5978	0.0000	-2.2375	0.0000
ENSG00000111432	fzd10	ENSDARG00000068213	-0.5991	0.0000	-2.2507	0.0000
ENSG00000125779	pank2	ENSDARG00000003311	-0.6001	0.0000	-1.7780	0.0000
ENSG00000052802	msmo1	ENSDARG00000055876	-0.6012	0.0000	-2.0554	0.0000
ENSG00000155269	gpr78a	ENSDARG00000074600	-0.6051	0.0000	-2.5454	0.0000
ENSG00000113504	slc12a7b	ENSDARG00000062058	-0.6068	0.0000	-2.3213	0.0000
ENSG00000012124	CD22	ENSDARG00000039096	-0.6068	0.0001	-1.7349	0.0000
ENSG00000212907	mt-nd4l	ENSDARG00000063916	-0.6128	0.0000	-1.7215	0.0000
ENSG00000173404	insm1b	ENSDARG00000053301	-0.6137	0.0000	1.9543	0.0000
ENSG00000185345	park2	ENSDARG00000021555	-0.6143	0.0000	-1.7272	0.0000
ENSG00000196711	FAM150A	ENSDARG00000074387	-0.6169	0.0000	-3.9982	0.0000
ENSG00000146112	ppp1r18	ENSDARG00000071251	-0.6172	0.0000	-2.2361	0.0000
ENSG00000114124	grk7a	ENSDARG00000020602	-0.6192	0.0000	-4.9327	0.0000
ENSG00000215218	ube2ql1	ENSDARG00000079276	-0.6224	0.0000	-3.7401	0.0000
ENSG00000142677	ifnlr1	ENSDARG00000087131	-0.6234	0.0043	-1.8787	0.0000
ENSG00000140463	bbs4	ENSDARG00000063522	-0.6253	0.0000	-1.8714	0.0000
ENSG00000084710	efr3ba	ENSDARG00000033516	-0.6295	0.0000	-2.6607	0.0000
ENSG00000100314	cabp7b	ENSDARG00000060846	-0.6309	0.0000	-1.6549	0.0000
ENSG00000131089	arhgef9b	ENSDARG00000078624	-0.6360	0.0000	-2.4157	0.0000
ENSG00000215906	lactbl1b	ENSDARG00000070467	-0.6361	0.0000	-3.0921	0.0000
ENSG00000186765	fscn2b	ENSDARG00000074396	-0.6362	0.0000	-2.9635	0.0000
ENSG00000136261	bzw2	ENSDARG00000035918	-0.6365	0.0000	-2.8084	0.0000

ENSG00000081181	arg2	ENSDARG00000039269	-0.6382	0.0000	-3.4197	0.0000
ENSG00000144681	stac	ENSDARG00000074004	-0.6443	0.0000	-1.7263	0.0000
ENSG00000277893	srd5a2b	ENSDARG00000039067	-0.6456	0.0005	-3.3969	0.0000
ENSG00000112619	prph2b	ENSDARG00000014840	-0.6477	0.0000	-2.9248	0.0000
ENSG00000164776	phkg1b	ENSDARG00000069498	-0.6486	0.0000	-3.5336	0.0000
ENSG00000275832	arhgap23b	ENSDARG00000026840	-0.6498	0.0000	-1.6756	0.0000
ENSG00000108960	mmd	ENSDARG00000040387	-0.6505	0.0000	-2.6586	0.0000
ENSG00000139163	etnk1	ENSDARG00000019420	-0.6506	0.0000	-2.1729	0.0000
ENSG00000127220	abhd8b	ENSDARG00000076620	-0.6531	0.0000	-2.3815	0.0000
ENSG00000091831	esr1	ENSDARG00000004111	-0.6563	0.0000	-1.8147	0.0000
ENSG00000183638	rp1l1a	ENSDARG00000089458	-0.6567	0.0000	-2.7882	0.0000
ENSG00000130035	galnt8b.1	ENSDARG00000045851	-0.6587	0.0000	-2.2764	0.0000
ENSG00000124831	lrrfip1a	ENSDARG00000030012	-0.6592	0.0000	-2.4302	0.0000
ENSG00000198420	si:ch211-210b2.4	ENSDARG00000095212	-0.6620	0.0016	-2.1584	0.0000
ENSG00000182674	kcnb2	ENSDARG00000038862	-0.6628	0.0000	-3.2101	0.0000
ENSG00000142798	hspg2	ENSDARG00000076564	-0.6644	0.0000	-1.7016	0.0000
ENSG00000186642	PDE2A	ENSDARG00000079064	-0.6652	0.0000	-1.7755	0.0000
ENSG00000156973	pde6d	ENSDARG00000074892	-0.6709	0.0000	-1.9277	0.0000
ENSG00000186326	RGS9BP	ENSDARG00000086756	-0.6712	0.0000	-2.8337	0.0000
ENSG00000155530	lrguk	ENSDARG00000069698	-0.6732	0.0001	-1.6603	0.0000
ENSG00000198888	mt-nd1	ENSDARG00000063895	-0.6735	0.0000	-1.6014	0.0000
ENSG00000171126	kcng3	ENSDARG00000061622	-0.6737	0.0000	-1.7612	0.0000
ENSG00000120049	kcnip2	ENSDARG00000075846	-0.6789	0.0000	-2.0780	0.0000
ENSG00000103313	BX664721.4	ENSDARG00000090371	-0.6828	0.0265	-1.6088	0.0000
ENSG00000189431	rassf10a	ENSDARG00000077424	-0.6895	0.0000	-1.8712	0.0000
ENSG00000070019	GUCY2C	ENSDARG00000056045	-0.6908	0.0000	-2.2363	0.0000
ENSG00000148798	inaa	ENSDARG00000011862	-0.6914	0.0000	-2.5351	0.0000
ENSG00000149305	htr3b	ENSDARG00000061749	-0.6919	0.0223	-1.7294	0.0000
ENSG00000187664	hapln4	ENSDARG00000018542	-0.6937	0.0000	-4.0060	0.0000
ENSG00000115419	glsb	ENSDARG00000040705	-0.6982	0.0000	-1.6022	0.0000
ENSG00000149527	plch2b	ENSDARG00000056805	-0.7010	0.0000	-2.5242	0.0000
ENSG00000071909	myo3b	ENSDARG00000006892	-0.7020	0.0000	-1.9989	0.0000
ENSG00000158125	xdh	ENSDARG00000055240	-0.7039	0.0000	-2.4622	0.0000
ENSG00000075415	slc25a3a	ENSDARG00000027424	-0.7044	0.0000	-1.8171	0.0000
ENSG00000105507	cabp5b	ENSDARG00000028485	-0.7056	0.0000	-2.0909	0.0000
ENSG00000111077	tns2b	ENSDARG00000068397	-0.7067	0.0000	-2.4915	0.0000
ENSG00000186862	pdzd7b	ENSDARG00000090631	-0.7081	0.0000	-2.0239	0.0000
ENSG00000198892	SHISA4	ENSDARG00000071541	-0.7097	0.0001	-2.8074	0.0000
ENSG00000143258	usp21	ENSDARG00000094280	-0.7104	0.0000	-3.4299	0.0000
ENSG00000204977	trim13	ENSDARG00000010010	-0.7120	0.0000	-2.5205	0.0000
ENSG00000006071	ABCC8	ENSDARG00000077007	-0.7179	0.0000	-5.4984	0.0000
ENSG00000118308	si:ch211-163l21.11	ENSDARG00000095855	-0.7183	0.0000	-4.7157	0.0000
ENSG00000128285	mchr1b	ENSDARG00000022525	-0.7261	0.0000	-3.0082	0.0000
ENSG00000168263	kcnv2a	ENSDARG00000076644	-0.7277	0.0000	-1.6289	0.0000
ENSG00000144834	tagln3b	ENSDARG00000058394	-0.7289	0.0000	2.0411	0.0000
ENSG00000133105	rxfp2a	ENSDARG00000019660	-0.7297	0.0000	-2.4594	0.0000
ENSG00000162889	mapkapk2b	ENSDARG00000018530	-0.7303	0.0000	-2.3505	0.0000
ENSG00000182795	C11H1orf116	ENSDARG00000070229	-0.7363	0.0000	-4.7786	0.0000
ENSG00000198886	mt-nd4	ENSDARG00000063917	-0.7371	0.0000	-1.9569	0.0000
ENSG00000198763	mt-nd2	ENSDARG00000063899	-0.7424	0.0000	-1.6922	0.0000
ENSG00000189292	fam150bb	ENSDARG00000042815	-0.7468	0.0000	-3.5010	0.0000
ENSG00000126895	avpr2aa	ENSDARG00000007436	-0.7472	0.0002	-2.1854	0.0000
ENSG00000130477	UNC13A	ENSDARG00000061829	-0.7474	0.0000	-1.6002	0.0000
ENSG00000151789	ZNF385D	ENSDARG00000086162	-0.7481	0.0000	-2.6161	0.0000
ENSG00000071626	dazap1	ENSDARG00000070846	-0.7490	0.0000	-1.9691	0.0000
ENSG00000109738	glrba	ENSDARG00000052782	-0.7491	0.0000	-2.0160	0.0000
ENSG00000163377	fam19a4b	ENSDARG00000062471	-0.7493	0.0000	-1.6766	0.0000
ENSG00000166411	idh3a	ENSDARG00000030278	-0.7502	0.0000	-1.6539	0.0000

ENSG00000138078	prepl	ENSDARG00000017853	-0.7506	0.0000	-2.1946	0.0000
ENSG00000122692	smu1b	ENSDARG00000051970	-0.7513	0.0000	-5.4697	0.0000
ENSG00000172350	abcg4b	ENSDARG00000078068	-0.7544	0.0000	-2.0274	0.0000
ENSG00000131730	ckmt2b	ENSDARG00000039929	-0.7544	0.0000	-2.8372	0.0000
ENSG00000178226	zgc:100868	ENSDARG00000004748	-0.7549	0.0230	-1.6674	0.0000
ENSG00000181264	tmem136b	ENSDARG00000035163	-0.7578	0.0000	-2.6267	0.0000
ENSG00000118308	lrmp	ENSDARG00000045574	-0.7639	0.0000	-2.0770	0.0000
ENSG00000137558	pi15b	ENSDARG00000061292	-0.7655	0.0759	-1.7655	0.0004
ENSG00000154319	fam167ab	ENSDARG00000023952	-0.7717	0.0000	-1.8869	0.0000
ENSG00000120451	snx19a	ENSDARG00000061101	-0.7723	0.0000	-2.4580	0.0000
ENSG00000044459	cntln	ENSDARG00000033345	-0.7724	0.0000	-1.8280	0.0000
ENSG00000188582	PAQR9	ENSDARG00000052059	-0.7732	0.0000	-1.5916	0.0000
ENSG00000107295	sh3gl2	ENSDARG00000023600	-0.7757	0.0000	-2.8463	0.0000
ENSG00000114771	aadac	ENSDARG00000063621	-0.7772	0.0000	-2.1713	0.0000
ENSG00000106128	ghrhrl	ENSDARG00000095788	-0.7791	0.0001	-1.5954	0.0000
ENSG00000172209	gpr22b	ENSDARG00000045383	-0.7835	0.0008	-1.5850	0.0000
ENSG00000008118	camk1ga	ENSDARG00000044526	-0.7849	0.0000	-4.3215	0.0000
ENSG00000188338	slc38a3b	ENSDARG00000091061	-0.7869	0.0000	-1.8722	0.0000
ENSG00000175164	gbgt1l1	ENSDARG00000025275	-0.7885	0.0519	-1.9260	0.0001
ENSG00000111262	kcna1a	ENSDARG00000062942	-0.7899	0.0000	-1.8066	0.0000
ENSG00000132518	gc3	ENSDARG00000026820	-0.7915	0.0000	-4.1433	0.0000
ENSG00000108381	aspa	ENSDARG00000005154	-0.7988	0.0000	-1.5824	0.0000
ENSG00000176095	ip6k1	ENSDARG00000073744	-0.7990	0.0000	-2.8255	0.0000
ENSG00000138669	prkg2	ENSDARG00000054741	-0.7996	0.0000	-7.5209	0.0000
ENSG00000167080	b4galnt2.2	ENSDARG00000070605	-0.7997	0.0000	-2.3219	0.0000
ENSG00000106086	plekha8	ENSDARG00000040698	-0.8010	0.0000	-1.9634	0.0000
ENSG00000158079	ptpdc1a	ENSDARG00000061656	-0.8021	0.0000	-2.0788	0.0000
ENSG00000124232	rbpjl	ENSDARG00000074148	-0.8032	0.0000	-1.6130	0.0000
ENSG00000131828	pdha1b	ENSDARG00000010555	-0.8071	0.0000	-2.3219	0.0000
ENSG00000167011	NAT16	ENSDARG00000086222	-0.8089	0.0000	-2.5951	0.0000
ENSG00000081800	slc13a1	ENSDARG00000045638	-0.8121	0.0000	-2.7790	0.0000
ENSG00000165406	march8	ENSDARG00000062489	-0.8170	0.0000	-2.3267	0.0000
ENSG00000126838	A2ML1	ENSDARG00000093199	-0.8207	0.0000	-2.0695	0.0000
ENSG00000151014	NOCT	ENSDARG00000078525	-0.8226	0.0000	-3.1276	0.0000
ENSG00000164116	gucy1a3	ENSDARG00000013787	-0.8246	0.0000	-2.5135	0.0000
ENSG00000167705	rilp	ENSDARG00000074660	-0.8271	0.0000	-1.6979	0.0000
ENSG00000101298	snphb	ENSDARG00000075539	-0.8346	0.0000	-2.0315	0.0000
ENSG00000273611	znhit3	ENSDARG00000023950	-0.8360	0.0000	-1.7960	0.0000
ENSG00000138821	SLC39A8	ENSDARG00000056757	-0.8395	0.0004	-2.0286	0.0000
ENSG00000105516	dbpb	ENSDARG00000057652	-0.8419	0.0000	-3.4867	0.0000
ENSG00000088836	slc4a11	ENSDARG00000075532	-0.8433	0.0000	-3.8389	0.0000
ENSG00000138675	fgf5	ENSDARG00000035377	-0.8480	0.0032	-2.1699	0.0000
ENSG00000159445	them4	ENSDARG00000061621	-0.8491	0.0000	-1.5847	0.0000
ENSG00000004838	zmynd10	ENSDARG00000002406	-0.8515	0.0014	1.6910	0.0000
ENSG00000134183	gnat2	ENSDARG00000042529	-0.8533	0.0000	-3.8543	0.0000
ENSG00000077279	dclk2a	ENSDARG00000034093	-0.8539	0.0000	-1.9422	0.0000
ENSG00000249961	ccdc79	ENSDARG00000058587	-0.8574	0.0000	-2.1793	0.0000
ENSG00000075073	tacr2	ENSDARG00000074509	-0.8586	0.0000	-2.4787	0.0000
ENSG00000112992	NNT	ENSDARG00000095765	-0.8613	0.0000	-2.0133	0.0000
ENSG00000116014	kiss1ra	ENSDARG00000002728	-0.8620	0.0000	-1.6671	0.0000
ENSG00000182533	cav3	ENSDARG00000024141	-0.8625	0.0067	-2.0995	0.0000
ENSG00000168477	tnxba	ENSDARG00000001760	-0.8639	0.0007	-1.7529	0.0000
ENSG00000101605	myom1a	ENSDARG00000061249	-0.8680	0.0000	-1.7017	0.0000
ENSG00000100503	nin	ENSDARG00000060298	-0.8710	0.0000	-3.9665	0.0000
ENSG00000172482	agxtb	ENSDARG00000018478	-0.8719	0.0000	-3.0308	0.0000
ENSG00000198786	mt-nd5	ENSDARG00000063921	-0.8728	0.0000	-1.8548	0.0000
ENSG00000110318	cep126	ENSDARG00000068398	-0.8733	0.0000	-3.7284	0.0000
ENSG00000054654	syne2a	ENSDARG00000030107	-0.8751	0.0000	-2.0794	0.0000

ENSG00000055813	ccdc85a	ENSDARG00000039497	-0.8803	0.0000	-1.8545	0.0000
ENSG00000148288	CABZ01024426.1	ENSDARG00000011283	-0.8863	0.0051	-2.1234	0.0000
ENSG00000188229	zgc:55461	ENSDARG00000041723	-0.8880	0.0000	2.0764	0.0000
ENSG00000104722	nefmb	ENSDARG00000043697	-0.8908	0.0000	-1.8669	0.0000
ENSG00000197142	acsl5	ENSDARG00000075931	-0.8948	0.0001	-2.3099	0.0000
ENSG00000158828	pink1	ENSDARG00000001929	-0.9082	0.0000	-1.6250	0.0000
ENSG00000163399	atp1a1a.2	ENSDARG00000007739	-0.9088	0.0000	-1.8031	0.0000
ENSG00000162877	pm20d1.1	ENSDARG00000037551	-0.9115	0.0000	-1.6220	0.0000
ENSG00000148399	dph7	ENSDARG00000062083	-0.9121	0.0000	-2.0549	0.0000
ENSG00000197580	bco2b	ENSDARG00000041715	-0.9125	0.0000	-1.7984	0.0000
ENSG00000117155	ssx2ipa	ENSDARG00000039521	-0.9176	0.0000	-1.6455	0.0000
ENSG00000186832	si:ch211-156l18.7	ENSDARG00000022334	-0.9220	0.0300	-1.7105	0.0003
ENSG00000081148	impg2a	ENSDARG00000019782	-0.9263	0.0000	-2.2086	0.0000
ENSG00000135437	rdh5	ENSDARG00000008306	-0.9275	0.0000	-2.3145	0.0000
ENSG00000133030	MPRIP	ENSDARG00000073998	-0.9375	0.0000	-2.8720	0.0000
ENSG00000140455	usp3	ENSDARG00000045641	-0.9403	0.0014	-1.6903	0.0000
ENSG00000171612	slc25a33	ENSDARG00000039931	-0.9425	0.0000	-2.8071	0.0000
ENSG00000104228	TRIM35	ENSDARG00000025601	-0.9475	0.0596	2.5850	0.0000
ENSG00000164776	phkg1a	ENSDARG00000030604	-0.9520	0.0000	-2.4233	0.0000
ENSG00000187942	ldlrad2	ENSDARG00000090297	-0.9534	0.0000	-3.1313	0.0000
ENSG00000159214	ccdc24	ENSDARG00000038793	-0.9537	0.0000	-1.7162	0.0000
ENSG00000275993	sik1	ENSDARG00000058606	-0.9554	0.0000	-2.7791	0.0000
ENSG00000180834	map6d1	ENSDARG00000079777	-0.9557	0.0000	-1.9061	0.0000
ENSG00000148702	habp2	ENSDARG00000057498	-0.9596	0.0000	-6.8820	0.0000
ENSG00000160973	foxh1	ENSDARG00000055630	-0.9598	0.0000	-3.1457	0.0000
ENSG00000177575	si:dkey-21h14.14	ENSDARG00000096184	-0.9668	0.0125	-1.6189	0.0002
ENSG00000162614	nexn	ENSDARG00000057317	-0.9668	0.0000	-2.2563	0.0000
ENSG00000169562	cx31.7	ENSDARG00000058064	-0.9794	0.0000	-3.3854	0.0000
ENSG00000140090	slc24a4b	ENSDARG00000067509	-0.9887	0.0000	-2.3053	0.0000
ENSG00000163399	atp1a1a.4	ENSDARG00000001870	-0.9888	0.0000	-1.7597	0.0000
ENSG00000104804	TULP2	ENSDARG00000062902	-0.9900	0.0000	-2.4345	0.0000
ENSG00000196878	LAMC3	ENSDARG00000059369	-0.9923	0.0000	-2.7920	0.0000
ENSG00000119938	ppp1r3ca	ENSDARG00000071005	-0.9932	0.0000	-2.4800	0.0000
ENSG00000111142	metap2a	ENSDARG00000069261	-1.0028	0.0000	-2.0594	0.0000
ENSG00000099204	ablim1a	ENSDARG00000060149	-1.0072	0.0000	-2.4947	0.0000
ENSG00000118520	arg1	ENSDARG00000057429	-1.0089	0.0000	-3.0663	0.0000
ENSG00000185761	ADAMTSL5	ENSDARG00000052118	-1.0091	0.0000	-1.8219	0.0000
ENSG00000007171	nos2a	ENSDARG00000026925	-1.0118	0.0000	-2.6206	0.0000
ENSG00000115756	hpcal1	ENSDARG00000022763	-1.0127	0.0000	-2.3186	0.0000
ENSG00000099814	cep170b	ENSDARG00000074636	-1.0176	0.0000	-1.7769	0.0000
ENSG00000187955	col14a1a	ENSDARG00000005762	-1.0233	0.0000	-1.7930	0.0000
ENSG00000010295	iffo1a	ENSDARG00000062976	-1.0311	0.0000	-2.3402	0.0000
ENSG00000070961	atp2b1b	ENSDARG00000007788	-1.0362	0.0000	-2.0566	0.0000
ENSG00000162522	si:ch211-194e15.5	ENSDARG00000061804	-1.0470	0.0000	-2.4148	0.0000
ENSG00000128617	opn1sw1	ENSDARG00000045677	-1.0523	0.0000	-2.2502	0.0000
ENSG00000182674	KCNB2	ENSDARG00000088842	-1.0526	0.0000	-2.6833	0.0000
ENSG00000122176	fmodb	ENSDARG00000010294	-1.0544	0.0000	-1.7549	0.0000
ENSG00000177453	NIM1K	ENSDARG00000079031	-1.0580	0.0000	-6.7467	0.0000
ENSG00000174225	arl13a	ENSDARG00000052575	-1.0600	0.0000	-1.6071	0.0000
ENSG00000125648	slc25a23b	ENSDARG00000024708	-1.0608	0.0000	-3.4500	0.0000
ENSG00000120903	chrna2a	ENSDARG00000006602	-1.0644	0.0000	-1.7993	0.0000
ENSG00000197444	OGDHL	ENSDARG00000079249	-1.0676	0.0000	-2.5653	0.0000
ENSG00000163399	atp1a1a.3	ENSDARG00000039131	-1.0742	0.0000	-1.7978	0.0000
ENSG00000157303	SUSD3	ENSDARG00000069038	-1.0744	0.0000	-3.6324	0.0000
ENSG00000129244	atp1b2b	ENSDARG00000034424	-1.0772	0.0000	-3.1957	0.0000
ENSG00000179542	slitrk4	ENSDARG00000079781	-1.0786	0.0000	-2.1375	0.0000
ENSG00000120053	got1	ENSDARG00000039093	-1.0829	0.0000	-1.5880	0.0000
ENSG00000123095	bhlhe41	ENSDARG00000041691	-1.0832	0.0000	-5.7668	0.0000

ENSG00000010379	SLC6A13	ENSDARG00000067567	-1.0833	0.0000	-1.6733	0.0000
ENSG00000115107	steap3	ENSDARG00000075641	-1.0837	0.0000	-2.1128	0.0000
ENSG00000090512	fetub	ENSDARG00000053973	-1.0887	0.0000	-6.0039	0.0000
ENSG00000255561	FDXACB1	ENSDARG00000095750	-1.0889	0.0000	-1.6463	0.0000
ENSG00000204335	sp5a	ENSDARG00000076571	-1.0926	0.0000	-1.7127	0.0000
ENSG00000132031	matn3a	ENSDARG00000069245	-1.0931	0.0000	-2.3370	0.0000
ENSG00000135750	kcnk1a	ENSDARG00000045067	-1.0983	0.0000	-2.5895	0.0000
ENSG00000174527	myo1ha	ENSDARG00000061968	-1.1009	0.0000	2.2231	0.0000
ENSG00000169994	myo7bb	ENSDARG00000077201	-1.1069	0.0000	-1.6374	0.0000
ENSG00000151790	tdo2a	ENSDARG00000071429	-1.1069	0.0012	-2.1069	0.0000
ENSG00000177150	fam210a	ENSDARG00000040186	-1.1095	0.0000	-2.9972	0.0000
ENSG00000104327	calb1	ENSDARG00000031598	-1.1095	0.0000	-1.6822	0.0000
ENSG00000007216	slc13a2	ENSDARG00000053853	-1.1100	0.0000	-2.8950	0.0000
ENSG00000147010	sh3kbp1	ENSDARG00000075853	-1.1157	0.0000	-1.8921	0.0000
ENSG00000166819	plin1	ENSDARG00000054048	-1.1355	0.0000	-1.7536	0.0000
ENSG00000126882	fam78ab	ENSDARG00000088222	-1.1391	0.0000	-1.7263	0.0000
ENSG00000164683	hey1	ENSDARG00000070538	-1.1411	0.0000	-2.0970	0.0000
ENSG00000231389	zgc:103599	ENSDARG00000031745	-1.1453	0.0000	4.6336	0.0000
ENSG00000090512	FETUB	ENSDARG00000070918	-1.1571	0.0000	-1.9549	0.0000
ENSG00000188816	hmx2	ENSDARG00000070954	-1.1946	0.0000	-1.8791	0.0000
ENSG00000246922	UBAP1L	ENSDARG00000091843	-1.1974	0.0000	-3.3793	0.0000
ENSG00000176136	mc5ra	ENSDARG00000031348	-1.2046	0.0000	-1.5899	0.0000
ENSG00000164142	fam160a1a	ENSDARG00000061021	-1.2102	0.0000	-2.3972	0.0000
ENSG00000198570	rd3	ENSDARG00000031600	-1.2109	0.0000	-1.9030	0.0000
ENSG00000170425	adora2b	ENSDARG00000002533	-1.2120	0.0000	-2.6198	0.0000
ENSG00000035664	dapk2a	ENSDARG00000061096	-1.2134	0.0000	-2.1832	0.0000
ENSG00000184678	si:dkey-108k21.9	ENSDARG00000034203	-1.2186	0.0000	-1.7905	0.0000
ENSG00000100276	rasl10a	ENSDARG00000062314	-1.2210	0.0000	-2.3134	0.0000
ENSG00000117407	artna	ENSDARG00000096252	-1.2224	0.0027	-1.6919	0.0001
ENSG00000118492	adgb	ENSDARG00000005595	-1.2224	0.0011	-1.7078	0.0000
ENSG00000106537	tspan13a	ENSDARG00000068883	-1.2280	0.0000	-2.0255	0.0000
ENSG00000174842	glmnb	ENSDARG00000010958	-1.2320	0.0000	-3.2388	0.0000
ENSG00000168427	klhl30	ENSDARG00000076094	-1.2479	0.0000	-2.9849	0.0000
ENSG00000138435	chrna1	ENSDARG00000009021	-1.2563	0.0019	-2.1043	0.0000
ENSG00000123358	nr4a1	ENSDARG00000000796	-1.2877	0.0000	-3.0787	0.0000
ENSG00000103313	zgc:194990	ENSDARG00000069407	-1.2945	0.0000	-2.5922	0.0000
ENSG00000136003	iscub	ENSDARG00000026582	-1.3006	0.0000	-2.4057	0.0000
ENSG00000064205	wisp2	ENSDARG00000077882	-1.3036	0.0000	-2.5434	0.0000
ENSG00000164542	si:dkey-222b8.4	ENSDARG00000057227	-1.3051	0.0000	-2.2133	0.0000
ENSG00000161654	lsm12b	ENSDARG00000045940	-1.3176	0.0000	-2.6708	0.0000
ENSG00000278259	MYO19	ENSDARG00000073761	-1.3206	0.0000	-3.6661	0.0000
ENSG00000138623	sema7a	ENSDARG00000078707	-1.3281	0.0000	-4.2199	0.0000
ENSG00000133055	mybpha	ENSDARG00000058799	-1.3373	0.0000	-1.6055	0.0000
ENSG00000065613	slkb	ENSDARG00000012574	-1.3377	0.0000	-2.1053	0.0000
ENSG00000117868	ESYT2	ENSDARG00000078964	-1.3425	0.0000	-2.3693	0.0000
ENSG00000173805	HAP1	ENSDARG00000074508	-1.3425	0.0000	-1.7865	0.0000
ENSG00000135175	pvalb8	ENSDARG00000037790	-1.3436	0.0000	-4.4083	0.0000
ENSG00000107551	rassf4	ENSDARG00000012460	-1.3475	0.0000	-2.0734	0.0000
ENSG00000079335	cdc14aa	ENSDARG00000013858	-1.3589	0.0000	-2.6433	0.0000
ENSG00000197467	COL13A1	ENSDARG00000091398	-1.3608	0.0000	-2.0847	0.0000
ENSG00000205186	fabp11b	ENSDARG00000002311	-1.3870	0.0000	-1.6280	0.0000
ENSG00000155886	slc24a2	ENSDARG00000042988	-1.3876	0.0000	-4.5745	0.0000
ENSG00000049192	adamts6	ENSDARG00000041982	-1.3992	0.0000	-1.9771	0.0000
ENSG00000198133	tmem229b	ENSDARG00000060569	-1.4225	0.0000	-1.7687	0.0000
ENSG00000108786	hsd17b1	ENSDARG00000027469	-1.4274	0.0000	-2.7618	0.0000
ENSG00000198276	uckl1a	ENSDARG00000001686	-1.4432	0.0000	-3.1295	0.0000
ENSG00000157873	TNFRSF14	ENSDARG00000068993	-1.4493	0.0000	3.1812	0.0000
ENSG00000115457	igfbp2a	ENSDARG00000052470	-1.4540	0.0000	-2.1203	0.0000

ENSG00000152556	pfkmb	ENSDARG00000060797	-1.4597	0.0000	-3.3537	0.0000
ENSG00000103313	btr07	ENSDARG00000094877	-1.4613	0.0000	-1.5887	0.0000
ENSG00000138379	mstna	ENSDARG00000042947	-1.4617	0.0000	-1.7824	0.0000
ENSG00000127588	gng13a	ENSDARG00000024740	-1.4664	0.0000	-6.1117	0.0000
ENSG00000183638	rp1l1b	ENSDARG00000088377	-1.4683	0.0000	-6.3911	0.0000
ENSG00000189410	sh2d5	ENSDARG00000014324	-1.4727	0.0000	-2.5621	0.0000
ENSG00000157978	ldlrap1b	ENSDARG00000039750	-1.4760	0.0000	-1.9069	0.0000
ENSG00000169064	zbbx	ENSDARG00000069723	-1.4794	0.0000	-3.6383	0.0000
ENSG00000131183	slc34a1b	ENSDARG00000054658	-1.5090	0.0009	-1.7500	0.0002
ENSG00000179933	C2H14orf119	ENSDARG00000054292	-1.5188	0.0000	2.0822	0.0000
ENSG00000180488	fam73a	ENSDARG00000020596	-1.5235	0.0000	-2.3563	0.0000
ENSG00000144191	cnga3a	ENSDARG00000070726	-1.5388	0.0000	-3.9820	0.0000
ENSG00000143537	adam15	ENSDARG00000089213	-1.5527	0.0000	-1.9501	0.0000
ENSG00000131668	barx1	ENSDARG00000007407	-1.5580	0.0000	-2.2685	0.0000
ENSG00000141642	elac1	ENSDARG00000045095	-1.5680	0.0000	-1.7843	0.0000
ENSG00000186472	pcloa	ENSDARG00000063299	-1.5784	0.0000	-2.4315	0.0000
ENSG00000163586	fabp1a	ENSDARG00000019357	-1.5809	0.0000	-3.3465	0.0000
ENSG00000185055	EFCAB10	ENSDARG00000092825	-1.5838	0.0000	-2.5146	0.0000
ENSG00000166603	mc4r	ENSDARG00000015515	-1.5850	0.0000	-0.8931	0.0013
ENSG00000106809	OGN	ENSDARG00000031489	-1.5850	0.0000	-1.2630	0.0000
ENSG00000141485	slc13a5a	ENSDARG00000077691	-1.6002	0.0000	-2.1819	0.0000
ENSG00000110436	slc1a2a	ENSDARG00000052138	-1.6023	0.0000	-5.2563	0.0000
ENSG00000167733	hsd11b1la	ENSDARG00000071377	-1.6043	0.0000	-0.6240	0.0000
ENSG00000166908	pip4k2cb	ENSDARG00000091637	-1.6046	0.0000	-3.9696	0.0000
ENSG00000151835	sacs	ENSDARG00000091042	-1.6055	0.0000	-1.1646	0.0000
ENSG00000157303	SUSD3	ENSDARG00000069607	-1.6133	0.0000	-4.9191	0.0000
ENSG00000187553	cyp26c1	ENSDARG00000056029	-1.6222	0.0000	-0.1327	0.0792
ENSG00000100593	ism2b	ENSDARG00000053859	-1.6263	0.0000	-3.4532	0.0000
ENSG00000116670	MAD2L2	ENSDARG00000042456	-1.6290	0.0000	0.2726	0.0057
ENSG00000162692	si:dkey-60b12.4	ENSDARG00000078201	-1.6292	0.0000	-1.2978	0.0000
ENSG00000158467	zgc:158222	ENSDARG00000051873	-1.6323	0.0015	-0.8667	0.0595
ENSG00000135093	usp30	ENSDARG00000056842	-1.6383	0.0000	-1.5796	0.0000
ENSG00000125744	rtn2b	ENSDARG00000057027	-1.6471	0.0000	-3.7685	0.0000
ENSG00000182223	zar1	ENSDARG00000019706	-1.6491	0.0000	-1.4647	0.0000
ENSG00000276547	pcdh2g13	ENSDARG00000078190	-1.6598	0.0000	-0.7075	0.0000
ENSG00000276547	pcdh2g6	ENSDARG00000071891	-1.6652	0.0000	-0.3093	0.0034
ENSG00000136872	aldob	ENSDARG00000053684	-1.6674	0.0000	-0.5261	0.0178
ENSG00000065618	col17a1a	ENSDARG00000069415	-1.6775	0.0000	-2.3246	0.0000
ENSG00000147872	plin2	ENSDARG00000042332	-1.6811	0.0000	0.5950	0.0000
ENSG00000186480	insig1	ENSDARG00000010658	-1.6856	0.0000	-1.6075	0.0000
ENSG00000114859	clcn2	ENSDARG00000062427	-1.6861	0.0000	-2.5854	0.0000
ENSG00000169021	uqcrfs1	ENSDARG00000007745	-1.7033	0.0000	-5.3978	0.0000
ENSG00000105559	PLEKHA4	ENSDARG00000071460	-1.7173	0.0000	-3.9662	0.0000
ENSG00000100242	SUN2	ENSDARG00000086490	-1.7186	0.0000	-1.1115	0.0000
ENSG00000162490	draxin	ENSDARG00000058256	-1.7221	0.0000	-0.4598	0.0000
ENSG00000148339	slc25a25b	ENSDARG00000035468	-1.7222	0.0000	-2.8522	0.0000
ENSG00000155850	slc26a2	ENSDARG00000011618	-1.7258	0.0000	-3.0864	0.0000
ENSG00000121316	PLBD1	ENSDARG00000076015	-1.7392	0.0000	-1.4812	0.0000
ENSG00000163362	C8H1orf106	ENSDARG00000078155	-1.7425	0.0000	-0.0251	0.8491
ENSG00000179178	TMEM125	ENSDARG00000073768	-1.7457	0.0000	-1.3754	0.0000
ENSG00000214513	noto	ENSDARG00000021201	-1.7480	0.0000	-2.7115	0.0000
ENSG00000155380	SLC16A1	ENSDARG00000016963	-1.7500	0.0000	-2.1220	0.0000
ENSG00000183255	pttg1ipb	ENSDARG00000040039	-1.7530	0.0000	-2.7703	0.0000
ENSG00000275713	zgc:171759	ENSDARG00000091728	-1.7544	0.0000	-3.0947	0.0000
ENSG00000079931	moxd1	ENSDARG00000031136	-1.7558	0.0000	-1.4186	0.0000
ENSG00000124205	edn3b	ENSDARG00000086669	-1.7749	0.0000	-2.5663	0.0000
ENSG00000115355	ccdc88aa	ENSDARG00000078440	-1.7953	0.0000	-1.0022	0.0000
ENSG00000130164	ldlra	ENSDARG00000029476	-1.8013	0.0000	-2.5187	0.0000

ENSG00000164707	slc13a4	ENSDARG00000059053	-1.8074	0.0000	-2.8074	0.0000
ENSG00000074410	ca12	ENSDARG00000045644	-1.8147	0.0000	-2.3152	0.0000
ENSG00000118729	casq2	ENSDARG00000008982	-1.8182	0.0000	-1.2081	0.0001
ENSG00000114124	grk7b	ENSDARG00000055534	-1.8391	0.0000	-2.2211	0.0000
ENSG00000173511	VEGFB	ENSDARG00000090997	-1.8425	0.0000	-1.3860	0.0000
ENSG00000129673	aanat2	ENSDARG00000079802	-1.8565	0.0000	-0.3454	0.0000
ENSG00000104549	sqlea	ENSDARG00000079946	-1.8739	0.0000	-2.3665	0.0000
ENSG00000130150	mospd2	ENSDARG00000026024	-1.8756	0.0000	-1.1735	0.0000
ENSG00000120068	hoxb8b	ENSDARG00000054025	-1.8791	0.0000	-2.0426	0.0000
ENSG00000166833	nav2b	ENSDARG00000001879	-1.8833	0.0000	-1.4482	0.0000
ENSG00000197921	her2	ENSDARG00000038205	-1.8845	0.0000	-0.8301	0.0203
ENSG00000153157	sycp2l	ENSDARG00000079190	-1.8850	0.0000	-0.6895	0.0000
ENSG00000137831	uacab	ENSDARG00000060238	-1.8905	0.0000	-0.2741	0.0000
ENSG00000183185	gabrr3a	ENSDARG00000027153	-1.8967	0.0000	-1.5917	0.0000
ENSG00000154175	abi3bpb	ENSDARG00000071095	-1.9082	0.0000	-2.3792	0.0000
ENSG00000162946	disc1	ENSDARG00000021895	-1.9210	0.0000	-1.7367	0.0000
ENSG00000185527	PDE6H	ENSDARG00000056791	-1.9298	0.0000	-3.9148	0.0000
ENSG00000173698	adgrg2b	ENSDARG00000088937	-1.9364	0.0000	-1.1506	0.0000
ENSG00000124701	apobec2a	ENSDARG00000018881	-1.9573	0.0000	-2.1637	0.0000
ENSG00000008405	cry1ba	ENSDARG00000069074	-1.9690	0.0000	-1.2293	0.0000
ENSG00000182405	pgdb4	ENSDARG00000076178	-1.9783	0.0000	-1.2914	0.0000
ENSG00000104490	ncaldb	ENSDARG00000011334	-2.0156	0.0000	-6.1935	0.0000
ENSG00000132429	popdc3	ENSDARG00000058551	-2.0211	0.0000	-2.3012	0.0000
ENSG00000160202	hspb6	ENSDARG00000077236	-2.0306	0.0000	-4.5905	0.0000
ENSG00000134121	chl1	ENSDARG00000076233	-2.0353	0.0000	-0.2025	0.0000
ENSG00000261934	pcdhga9	ENSDARG00000076707	-2.0435	0.0000	-1.2164	0.0000
ENSG00000147573	trim55a	ENSDARG00000029596	-2.0834	0.0000	-1.6428	0.0000
ENSG00000084674	apobb.1	ENSDARG00000022767	-2.0995	0.0000	-1.9683	0.0000
ENSG00000154122	ankha	ENSDARG00000071724	-2.1076	0.0000	-4.3593	0.0000
ENSG00000153201	si:zfos-979f1.2	ENSDARG00000011652	-2.1137	0.0000	-1.6650	0.0000
ENSG00000116745	rpe65a	ENSDARG00000007480	-2.1143	0.0000	-3.1501	0.0000
ENSG00000010219	dyrk4	ENSDARG00000070734	-2.1241	0.0000	-3.8547	0.0000
ENSG00000110042	dtx1	ENSDARG00000076302	-2.1299	0.0000	-2.4347	0.0000
ENSG00000166582	cenpv	ENSDARG00000092285	-2.1301	0.0000	-1.2581	0.0000
ENSG00000162267	itih1	ENSDARG00000055053	-2.1809	0.0000	0.6406	0.0000
ENSG00000196482	esrrgb	ENSDARG00000011696	-2.2092	0.0000	-7.3824	0.0000
ENSG00000128617	opn1mw2	ENSDARG00000044280	-2.2556	0.0000	-4.5166	0.0000
ENSG00000177575	si:dkey-21h14.12	ENSDARG00000094603	-2.2820	0.0000	-1.5715	0.0000
ENSG00000100652	slc10a1	ENSDARG00000030588	-2.2854	0.0000	-2.8704	0.0000
ENSG00000121207	LRAT	ENSDARG00000077652	-2.3026	0.0000	-2.8875	0.0000
ENSG00000158571	pfkfb1	ENSDARG00000037140	-2.3345	0.0000	-3.3227	0.0000
ENSG00000162490	DRAXIN	ENSDARG00000087681	-2.3745	0.0000	-0.4863	0.0000
ENSG00000174343	pkdccb	ENSDARG00000038235	-2.4150	0.0000	-3.4374	0.0000
ENSG00000175899	A2ML1	ENSDARG00000041685	-2.4170	0.0000	-1.0647	0.0000
ENSG00000112078	kctd20	ENSDARG00000059381	-2.4205	0.0000	-0.7837	0.0000
ENSG00000134996	ostf1	ENSDARG00000086835	-2.4514	0.0000	-2.8373	0.0000
ENSG00000072571	hmmr	ENSDARG00000021794	-2.4769	0.0000	-0.9241	0.0000
ENSG00000065809	si:ch211-160o17.6	ENSDARG00000059399	-2.4787	0.0000	-2.8511	0.0000
ENSG00000125898	fam110a	ENSDARG00000015293	-2.4867	0.0000	-0.3712	0.0003
ENSG00000170807	lmod2b	ENSDARG00000045864	-2.4930	0.0000	-2.2256	0.0000
ENSG00000197321	si:dkeyp-57f11.2	ENSDARG00000017036	-2.5136	0.0000	-3.8205	0.0000
ENSG00000261934	pcdhga9	ENSDARG00000071892	-2.5167	0.0000	-0.0684	0.4906
ENSG00000177453	nim1k	ENSDARG00000079988	-2.5288	0.0000	-2.2581	0.0000
ENSG00000117983	muc5.2	ENSDARG00000058556	-2.6389	0.0000	-1.0335	0.0000
ENSG00000188779	skor1a	ENSDARG00000069030	-2.6399	0.0000	-1.3410	0.0000
ENSG00000100154	ttc28	ENSDARG00000089527	-2.6991	0.0000	-2.9222	0.0000
ENSG00000186051	tal2	ENSDARG00000042041	-2.7828	0.0000	-4.2273	0.0000
ENSG00000244274	DBNDD2	ENSDARG00000074904	-2.7913	0.0000	-4.7121	0.0000

ENSG00000128626	mrps12	ENSDARG00000089095	-2.8143	0.0000	-0.3004	0.0050
ENSG00000255408	pcdh2aa15	ENSDARG00000088136	-2.8194	0.0000	-0.4639	0.0014
ENSG00000175182	fam131a	ENSDARG00000077022	-2.9061	0.0000	-2.2637	0.0000
ENSG00000137869	cyp19a1	ENSDARG00000009852	-2.9235	0.0000	-3.4247	0.0000
ENSG00000139330	kera	ENSDARG00000056938	-2.9688	0.0000	-3.7561	0.0000
ENSG00000116574	RHOU	ENSDARG00000005626	-2.9765	0.0000	-1.8827	0.0000
ENSG00000146809	asb15b	ENSDARG00000045863	-2.9954	0.0000	-1.3231	0.0000
ENSG00000124215	cdh27	ENSDARG00000055093	-3.0376	0.0000	-3.6124	0.0000
ENSG00000166535	A2ML1	ENSDARG00000041645	-3.0743	0.0000	-2.3485	0.0000
ENSG00000175899	A2ML1	ENSDARG00000041645	-3.0743	0.0000	-2.3485	0.0000
ENSG00000101247	si:ch211-93g23.2	ENSDARG00000070558	-3.2281	0.0000	-4.1756	0.0000
ENSG00000163116	STPG2	ENSDARG00000061060	-3.3827	0.0000	-1.0475	0.0000
ENSG00000167612	ankrd33aa	ENSDARG00000055638	-3.4241	0.0000	-4.2274	0.0000
ENSG00000102076	opn1lw2	ENSDARG00000044861	-3.5021	0.0000	-3.8825	0.0000
ENSG00000072274	tfr1b	ENSDARG00000077372	-3.5286	0.0000	-1.1345	0.0000
ENSG00000139329	lum	ENSDARG00000045580	-3.5850	0.0000	-2.3479	0.0000
ENSG00000175899	a2ml	ENSDARG00000056314	-3.6342	0.0000	-2.2650	0.0000
ENSG00000179142	cyp11c1	ENSDARG00000042014	-3.8363	0.0000	-2.8342	0.0000
ENSG00000261934	pcdhga9	ENSDARG00000071894	-3.8939	0.0000	-0.7488	0.0000
ENSG00000221988	zgc:77118	ENSDARG00000093176	-3.9228	0.0000	-2.0267	0.0000
ENSG00000102313	itih6	ENSDARG00000056037	-4.1243	0.0000	-4.4173	0.0000
ENSG00000095627	tdrd1	ENSDARG00000007465	-4.3112	0.0000	-6.8962	0.0000
ENSG00000159176	csrp1b	ENSDARG00000030980	-5.0624	0.0000	-1.9965	0.0000
ENSG00000102924	cbln12	ENSDARG00000068232	-7.1972	0.0000	-6.1972	0.0000
